# Discovery of All Three Types in Cartilaginous Fishes Enables Phylogenetic Resolution of the Origins and Evolution of Interferons

**DOI:** 10.3389/fimmu.2019.01558

**Published:** 2019-07-12

**Authors:** Anthony K. Redmond, Jun Zou, Christopher J. Secombes, Daniel J. Macqueen, Helen Dooley

**Affiliations:** ^1^School of Biological Sciences, University of Aberdeen, Aberdeen, United Kingdom; ^2^Centre for Genome-Enabled Biology and Medicine, University of Aberdeen, Aberdeen, United Kingdom; ^3^Smurfit Institute of Genetics, Trinity College Dublin, University of Dublin, Dublin, Ireland; ^4^Scottish Fish Immunology Research Centre, Institute of Biological and Environmental Sciences, University of Aberdeen, Aberdeen, United Kingdom; ^5^Key Laboratory of Exploration and Utilization of Aquatic Genetic Resources, Ministry of Education, Shanghai Ocean University, Shanghai, China; ^6^The Roslin Institute and Royal (Dick) School of Veterinary Studies, The University of Edinburgh, Edinburgh, United Kingdom; ^7^Department of Microbiology and Immunology, University of Maryland School of Medicine, Baltimore, MD, United States; ^8^Institute of Marine and Environmental Technology, Baltimore, MD, United States

**Keywords:** interferon, phylogenetics, evolution, antiviral immunity, cytokine, retrotransposition, jawed vertebrate, shark

## Abstract

Interferons orchestrate host antiviral responses in jawed vertebrates. They are categorized into three classes; IFN1 and IFN3 are the primary antiviral cytokine lineages, while IFN2 responds to a broader variety of pathogens. The evolutionary relationships within and between these three classes have proven difficult to resolve. Here, we reassess interferon evolution, considering key phylogenetic pitfalls including taxon sampling, alignment quality, model adequacy, and outgroup choice. We reveal that cartilaginous fishes, and hence the jawed vertebrate ancestor, possess(ed) orthologs of all three interferon classes. We show that IFN3 groups sister to IFN1, resolve the origins of the human IFN3 lineages, and find that intronless IFN3s emerged at least three times. IFN2 genes are highly conserved, except for IFN-γ-rel, which we confirm resulted from a teleost-specific duplication. Our analyses show that IFN1 phylogeny is highly sensitive to phylogenetic error. By accounting for this, we describe a new backbone IFN1 phylogeny that implies several IFN1 genes existed in the jawed vertebrate ancestor. One of these is represented by the intronless IFN1s of tetrapods, including mammalian-like repertoires of reptile IFN1s and a subset of amphibian IFN1s, in addition to newly-identified intron-containing shark IFN1 genes. IFN-f, previously only found in teleosts, likely represents another ancestral jawed vertebrate IFN1 family member, suggesting the current classification of fish IFN1s into two groups based on the number of cysteines may need revision. The providence of the remaining fish IFN1s and the coelacanth IFN1s proved difficult to resolve, but they may also be ancestral jawed vertebrate IFN1 lineages. Finally, a large group of amphibian-specific IFN1s falls sister to all other IFN1s and was likely also present in the jawed vertebrate ancestor. Our results verify that intronless IFN1s have evolved multiple times in amphibians and indicate that no one-to-one orthology exists between mammal and reptile IFN1s. Our data also imply that diversification of the multiple IFN1s present in the jawed vertebrate ancestor has occurred through a rapid birth-death process, consistent with functional maintenance over a 450-million-year host-pathogen arms race. In summary, this study reveals a new model of interferon evolution important to our understanding of jawed vertebrate antiviral immunity.

## Introduction

Antiviral immunity in jawed vertebrates is directed by interferons released by host cells in response to viral pathogens (1, 2). Interferons are members of the class II α-helical cytokines along with interleukin (IL)-10,−19,−20,−22,−24, and −26 (hereafter called the IL-10 family), and are categorized into three classes, denoted as type I [e.g., IFN-α, β, κ, etc. in human [amongst others] and chicken], II (IFN-γ), and III (IFN-λs; known as IL-28A, IL-28B and IL29 in humans) interferons (hereafter called IFN1, IFN2, and IFN3), based on their receptors, genomic location, and sequence/structural homology (2, 3). Roles beyond antiviral immunity have recently come to light for interferons, and IFN2 has been shown to contribute mainly toward defense against bacterial (especially mycobacteria), parasitic and fungal pathogens, leaving IFN1 and IFN3 as the main antiviral cytokines (1, 2, 4–7).

**Figure 1:**
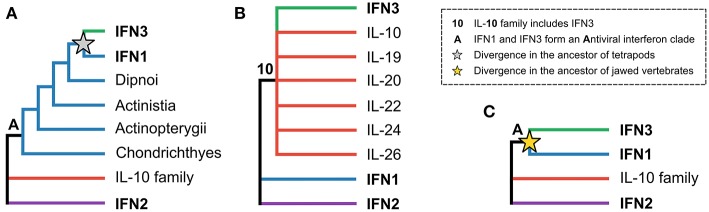
The three major hypotheses of IFN3 origins within the class II α-helical cytokine family. **(A)** Antiviral interferons form a clade, but IFN3 diverged from IFN1 in the tetrapod ancestor, in line with IFN3s only being found in tetrapods. **(B)** IFN3 is either within or closely related to the IL-10 family based on their shared structural characteristics. **(C)** Antiviral interferons form a clade but all three interferon classes existed in the jawed vertebrate ancestor.

The evolutionary relationships between the three interferon classes, as well as intra-class evolutionary histories, have received considerable attention, but have proven difficult to resolve. The origins of the IFN3 lineage are particularly contentious. While some early studies suggested that the IFN3 and IFN1 lineages diverged in tetrapods, with teleost fishes possessing IFN1/3-like molecules (Figure 1A) (8–10), other studies suggested that teleosts possessed either IFN3 (11, 12) or IFN1 orthologs (13). Later work, incorporating protein structures, showed that the teleost molecules were indeed IFN1s (14), and suggested that IFN3s likely emerged early in vertebrate evolution following whole genome duplication events (15–17). This scenario was supported by the discovery of IFN3 receptor homologs (along with those of IFN1 and IFN2) in cartilaginous fishes (18, 19). However, other structure-based studies concluded that IFN3 is either part of the IL-10 family (specifically IL-22 or IL-19) (Figure 1B) (3, 20–22), or sister to IFN1s (Figure 1C) (15). A recent study has also revived the idea that IFN3 may have emerged from within IFN1 in the tetrapod ancestor (Figure 1A) (23). Crucially, as no study has yet identified either the root of the class II α-helical cytokine family or orthologs of IFN3 genes outside tetrapods, none of these three hypotheses has been firmly rejected.

Evolutionary histories within each interferon class also remain unclear. For example, IFN2 is typically considered the most conserved interferon, however a tandem duplicate (IFN-γ-rel) has been found in some teleost fish-species (24), and phylogenetic analyses have failed to clarify whether this an ancient jawed vertebrate gene lost in other lineages (24–26), or teleost-specific (27). Multiple IFN3 genes often exist in individual species, but IFN3s are thought to be tetrapod-specific (28). However, very few studies have specifically focused on IFN3 evolution across vertebrates (28). The evolution of IFN1 genes, while better studied, also appears to be the most complicated. IFN1 genes are often present as lineage-specific clusters; for example, with the exception of IFN-κ, the IFN1 molecules of humans are evidently not directly orthologous with those of chickens (29–31). Clusters of lineage-specific IFN1s have also been observed in teleost fishes (32), classified as belonging to fish-specific group 1 or group 2 based on cysteine patterns (having two and four conserved cysteines, respectively) in the mature peptide sequence (9), and in amphibians (23). In fact, some phylogenetic analyses place all IFN1 sequences from mammals, teleosts, and amphibians into lineage-specific clades (9, 33–35), supporting a scenario where IFN1s evolve through concerted evolution (36, 37). This would imply that high-turnover, lineage-specific gene gain and loss events, and/or gene conversion are major driving forces of IFN1 evolution (37, 38). This is consistent with functional data, where individual genes appear to be specialized for defense against specific viruses. However, some studies have found phylogenetic relationships between IFN1s that are more difficult to interpret (23).

Poor resolution of interferon phylogenies hinders our ability to infer the history of evolutionary events including retro(trans)position, intron gains and losses, and changes in disulphide bridge structure. Amniote IFN1s are intronless and are classically thought to have arisen as a result of a retro(trans)position early in amniote evolution, as fish and amphibian interferons were found to contain four introns (2, 9, 19, 30, 33, 39). Recent studies have revealed that both intron-containing and intronless IFN1 genes also exist in amphibians, leading to two competing hypotheses to explain the origins of intronless amphibian IFN1 genes; (i) they arose from the same event as the amniote intronless IFN1s (23, 35), or (ii) they arose during independent retro(trans)position events (40). Intronless IFN3 genes also exist in mammals and amphibians (23), but whether they resulted from a single event or not remains to be tested. Similarly, two- and four-cysteine containing IFN1s exist in mammals and teleosts but it is thought that two-cysteine containing IFN1s emerged independently in each lineage; with intronless mammal and intron-containing teleost two-cysteine containing IFNs having lost a different cysteine pair (and hence disulphide bridge) from an ancestral four-cysteine containing IFN1 (19). Better resolution of IFN1 and IFN3 evolution could help determine both the frequency and timing of emergence of such features.

The primary amino acid sequence of interferons are short and rapidly evolving, both characteristics expected to promote phylogenetic error (41). Short alignments may have insufficient phylogenetic information to infer relationships between sequences and are more prone to stochastic errors. On the other hand, rapidly evolving sequences can be difficult to align and may induce systematic errors, resulting in long branch attraction (LBA) (42). Homoplasy (i.e., convergence due to hidden substitutions) is the best studied cause of LBA, and has previously been acknowledged as a concern when inferring immune gene phylogenies (41). Fortunately, it can often be counteracted by breaking long branches with additional taxa (43–45), applying site-heterogeneous models of evolution (42, 46), removing fast-evolving sites (47), and/or identifying the best outgroup (48–51), in addition to using outgroup-free methods (41, 52–55) to root the tree. Compositional heterogeneity, resulting from differing codon usage preferences among sequences under comparison can also lead to LBA through non-phylogenetic similarity between lineages (56, 57), and can be remedied by applying time-heterogeneous models of evolution (58, 59), or removing compositionally biased sites or sequences (56, 57, 60, 61). Other sources of systematic error have been identified (e.g., heterotachy, heteropecilly, non-independence of sites), but are either less well studied or thought to have a less important effect on tree topology (62). Attempting to account for multiple sources of error, or applying several error-attenuating strategies at once is thought to improve phylogenetic accuracy (49, 63, 64), and this has proven successful for immune genes in the past (41, 51).

Here, taking account of important phylogenetic considerations overlooked in past studies, we infer the origins and evolutionary history of interferons using a dataset that incorporates unprecedented sampling of both species and interferon diversity. Our findings offer a substantially overhauled model of interferon evolution and provide insights into the varied issues that hinder such studies, which have broader implications for immune gene phylogenetic analysis.

## Materials and Methods

### Homolog Identification and Characterization

TBLASTN (65) searches were carried out against a densely-sampled set of genomes spanning chordate phylogeny (Table S1). An e-value cut-off of 10 was used in all searches, and sequences with either >75% identity compared to the query sequence [a set of known phylogenetically diverse IFNs were used for all searches, while known sequences (Table S2) from closely related species were also applied on an *ad hoc* basis], and/or with a top BLASTP (66) hit against an interferon in the NCBI non-redundant protein database, were retained for further analysis. To increase taxon sampling of cartilaginous fishes beyond elephant shark [until recently the only cartilaginous fish species with a sequenced genome (18, 67)], transcriptome datasets for the small spotted-catshark were also analyzed from this lineage (41, 68, 69). Gene predictions were performed where a protein sequence was not already available, with the FGENESH+ webserver, using parameters for the closest related species available, and using either the blast hit or query as the homologous sequence (70). Structural homology prediction was performed through the Phyre2 protein structure prediction webserver using the “intensive” search option (71). Assessment of evolutionary conservation of sites necessary for IFN-λ3-receptor binding was achieved through visual comparison of multiple sequence alignment.

### Multiple Sequence Alignment

All multiple sequence alignments were generated using PRANK, which has been shown to improve inference of insertions and deletions compared to other alignment approaches (72). This should help avoid alignment of non-homologous sites, reducing the potential for phylogenetic error. Manual curation was also performed (e.g., positions with no homologous amino acid in other classes were removed when examining inter-class relationships). Due to the rapidly evolving nature of IFN1s a set of high-quality, known sequences (Table S2) was used to build a base alignment before adding additional sequences from transcriptome and draft genome datasets, which may be truncated and more error prone, to the IFN1 dataset. Prior to analyzing this dataset, the PRANK alignment process was bootstrapped using GUIDANCE, which identifies sites that are not consistently aligned (73, 74). Site alignments present in <93% of the GUIDANCE replicates were then removed to avoid use of unreliably aligned sites in phylogenetics (73, 74). The “—add” function of MAFFT was then used with the L-INS-i approach to add new sequences to this high-quality core alignment (75). Alignment positions present in only a single species were then removed. See Data S1 for all multiple sequence alignment files.

### Phylogenetic Analyses

All maximum likelihood phylogenetic analyses and model selection were performed in IQ-tree v1.6.7 (76). The Bayesian information criterion was used for model selection using IQ-tree's ModelFinder (77), and 1,000 ultrafast bootstrap replicates were generated to provide branch support values (78). IQ-tree was also used to detect compositionally biased sequences, using the built-in χ^2^ test (71).

Outgroup-free rooted phylogenetic analyses were performed using a relaxed clock model, that permits root inference while accommodating rate variation among different tree branches (52). We have previously applied this approach to root other fast-evolving immune gene families (41, 51, 54, 79, 80), and it appears to work consistently for such datasets, except in the face of extreme rate asymmetry (41). This analysis was performed in BEAST v1.8.3 (81) applying an uncorrelated lognormal relaxed molecular clock model (52), a Yule speciation prior (82, 83), and the best-fit amino acid substitution model (as inferred with IQ-tree). Two Markov chain Monte Carlo (MCMC) chains were run until effective samples sizes (>200) and convergence were sufficient, as assessed in Tracer v1.6 (84). Maximum clade credibility trees were generated in RootAnnotator (85).

Bayesian phylogenetic analyses incorporating outgroups were performed in PhyloBayes v4.1b (86), which also permits testing of site-heterogeneous models. Two MCMC chains were run for each analysis, until convergence was reached and effective sample sizes were sufficient for all statistics. This was assessed using the bpcomp (maxdiff <0.3) and tracecomp (effsize > 50, and rel_diff <0.3) programs within the PhyloBayes package (86).

### Site-Heterogeneous Models, Cross-Validation, and Posterior Predictive Analyses

Site-heterogeneous models typically allow for better detection of homoplasy by accommodating site-specific evolutionary constraints in phylogenomic datasets (42). Such models have been applied with the objective of generating more reliable immune gene phylogenies (87, 88), and have recently been shown to be capable of better explaining the site-specific evolutionary processes of aligned immune gene datasets (41). However, such models do not always provide a better fit for short alignments, and their relative fit cannot always be compared to standard models with the commonly applied information criteria. As such we used 10-fold cross-validation, as implemented in PhyloBayes (42, 86), to compare the relative fit of a range of site-heterogeneous mixture models to the best-fitting standard model for the IFN1 dataset [JTT+Γ (89, 90)]. The models tested included the infinite mixture model CAT (46), empirical derivations of CAT (C10/20/30/40/50/60) with limited numbers of site-categories, intended for gene family phylogenies (91), as well as an alternative site-heterogeneous model, WLSR5 (92), and a three-matrix substitution model, UL3, that loosely accommodates evolutionary process differences between structural features (91). Cross-validation relies on randomly partitioning the alignment into equal sized subsamples (10 here, as the analysis is 10-fold), before one of these subsamples is used for validation to test the model, while the rest are combined as a training set. This process is then repeated, using each of the other subsamples as the validation dataset, and then the average results are used for comparison against other models. We ran each individual chain (i.e., one chain for each of the 10 training sets for each model) for 1,000 points, using the first 100 as burn-in (i.e., 10 chains for each model tested).

In addition to assessing relative model fit, posterior predictive simulations (PPS) were also performed in PhyloBayes to determine if the model applied could adequately describe the real data for the tested statistic (42, 86). This approach consists of generating simulated data under the model in question, for comparison against the observed (i.e., real) data. Here, PPS was used to investigate the ability of models to account for homoplasy and compositional heterogeneity across lineages in the IFN1 dataset (42, 56, 57, 60). The compositional heterogeneity test was used to generate a second IFN1 dataset by identifying and removing sequences that deviate significantly from the assumption of homogeneity, measured at *Z*-scores < −2 and >2 (the default in PhyloBayes, which is slightly more inclusive than *P* < 0.05 cut-off). All PPS analyses were specifically performed under JTT+Γ, as this model should be the most susceptible to error compared to the tested mixtures, so we viewed this as more conservative. Finally, we also tested a time-heterogeneous (59), and a site- and time-heterogeneous (58) model for the IFN1 dataset, but these analyses failed to reach convergence despite running continuously for more than three months each.

### Testing Exacerbation of Potential Errors in Interferon Phylogenetics Analysis

Multiple approaches were tested to induce phylogenetic error (93) in the rapidly-evolving IFN1 family to better explain the discrepancy in the results of past studies, as well as the difficulty in inferring IFN1 evolutionary history. This included applying a more distantly related outgroup in place of the closest related outgroup to root the tree (50, 51, 94), inferring the phylogeny under less well-fitting substitution models (50, 51, 54, 93, 95), including sequences that introduce significant compositional bias in the analysis (63, 64), as well as sequence removal to lengthen target branches and increase the potential for LBA (93, 96).

## Results

### A Cartilaginous Fish IFN-λ

**Figure 2:**
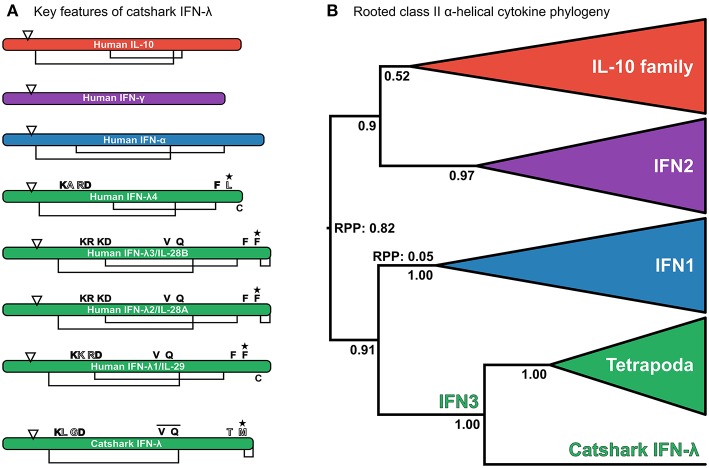
**(A)** Analysis of key residues in the catshark IFN-λ sequence compared to a set of other interferons and IL-10. Sequences are represented as cartoon bars, which are relatively scaled according to amino acid sequence length. Arrows denote the end of the signal peptide region, while disulphide bridges are shown as connected regions underneath each cartoon bar, with “C” in the C-terminal region being an unpaired Cys from the characteristic C-terminal disulphide bridge of IFN3s (22). Above the bar the most important residues for IFN-λ3 receptor binding are shown (22). Residues filled in black are conserved, whereas residues filled white are not well conserved, and gray-filled residues involve conserved replacements (e.g., K → R). The bar over the VXXQ motif of catshark IFN-λ indicates that this is not aligned perfectly to human IFN3s, while the star indicates that mutation of this residue abolishes binding in human IFN-λ3 (22). See Figure S1 for full alignment. **(B)** Relaxed clock (uncorrelated lognormal) rooted class II α-helical cytokine family phylogeny under JTT + I + τ and a Yule speciation prior. The tree is rooted at the best supported root position. Root posterior probabilities (RPP) are shown for branches with a non-negligible probability (i.e., posterior probability <0.05) of being the root. Posterior probabilities are also shown for key nodes, and clades representing individual family members, or the entire IL-10 family have been collapsed to emphasize deep relationships within the family.

Reciprocal BLAST searches of a multi-tissue small-spotted catshark transcriptome (41) revealed a putative cartilaginous fish IFN3 sequence. Characterization of this sequence by multiple sequence alignment against human interferon sequences and IL-10 (as a representative of the broader IL-10 family) support this assignment (Figure 2A and Figure S1). Additionally, the signature disulfide bridge-forming Cys pair were present at the C-terminus of this sequence (Figure 2A and Figure S1) (21, 22). Interestingly, the most important receptor binding sites of human IFN-λ are poorly conserved in this sequence (22); including Phe158, which is vital for human IFN-λ3-receptor interaction (22). However, this may not preclude antiviral functionality of catshark IFN3, considering that this residue is also not conserved in human IFN-λ4 (Figure 2A and Figure S1), which appears to be capable of binding the IFN3 receptor (97). Further, our preliminary analyses suggest that catshark IFN-λ is involved in antiviral defense (unpublished data). Submission to the Phyre2 protein structure prediction server, an approach which has previously been employed to aid orthology assignment of fast-evolving immune genes in cartilaginous fishes (98), also indicated a best-structural match of the putative catshark sequence to mammalian IFN-λs (Figure S2). Finally, phylogenetic analysis (see next section), verified this assignment; indeed, catshark IFN-λ forms a clade with tetrapod IFN3s, to the exclusion of other class II α-helical cytokines, with maximal support (posterior probability [PP] = 1.00; Figure 2B, Figure S3). The existence of a cartilaginous fish IFN3 allows us to unequivocally reject the hypothesis that IFN3s emerged by duplication from IFN1s in the tetrapod ancestor (Figure 1A) (8, 10, 23).

### Deep Relationships Within the Class II α-Helical Cytokine Family

To understand the evolutionary relationships between the interferon classes and other class II α-helical cytokines, and identify the closest outgroups to best infer within-class interferon relationships, we performed a phylogenetic analysis of the full class II α-helical cytokine family. A relaxed clock model was used to root this tree, as the deeper phylogenetic origins of the family, and thus potential outgroups, are not known. Our analysis supports a sister group relationship between IFN1 and IFN3 (PP = 0.91; Figure 2B and Figure S3), while on the other side of the tree root (root posterior probability [RPP] = 0.82; Figure 2B and Figure S3), a monophyletic IL-10 family is sister to IFN2 (PP = 0.9; Figure 2B and Figure S3). These findings reject the hypothesis that IFN3 is part of the IL-10 family (14, 21, 22) (Figure 1B), while the root placement suggests that the deepest divergence in the class II α-helical cytokines separates the main antiviral interferons from the rest of the family, consistent with the model of class II α-helical cytokine evolution proposed by Siupka et al. (15). A second root position placing IFN1 as sister to the rest of the family could not be rejected however, although this was only very weakly supported (PP = 0.05; yielding a 16:1 weighting in favor of the best root) (Figure 2B). These results concur with the conclusion that the IFN1/3s previously identified outside tetrapods are in fact true IFN1s, consistent with their structural and functional features (14). Further, by supporting a sister group relationship between IFN1 and IFN3 (Figure 1C), our findings indicate that IFN1 and IFN3 can be used as reciprocal outgroups in phylogenetic analyses, enabling outgroup-rooted IFN1 and IFN3 phylogenies without the inclusion of more distant, and potentially-biasing, outgroups like IFN2 and/or the IL-10 family (which by the same rationale can be applied as outgroups for each other).

### IFN2 Evolution Indicates That IFN-γ-Rel Is Teleost-Specific

**Figure 3:**
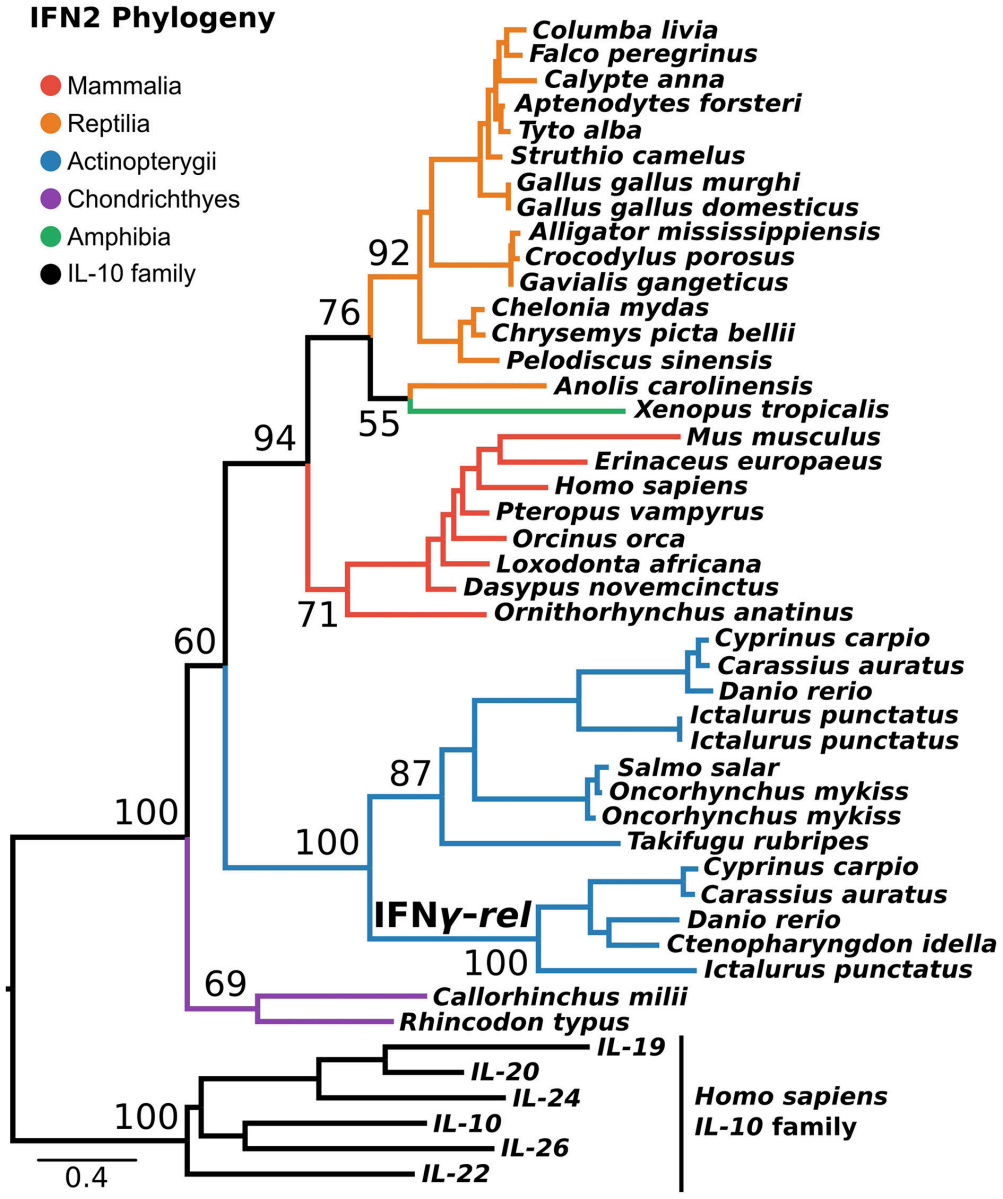
IFN2 phylogeny. Maximum likelihood consensus tree of the IFN2 genes under JTT+τ with the IL-10 family as outgroup. Ultrafast bootstrap support values are shown for key nodes.

IFN2 is the most structurally conserved of the interferon classes and is thought to have the simplest evolutionary history. Despite being present in single copy across most of vertebrate phylogeny, an additional gene, IFN-γ-rel (24), is present in tandem to IFN-γ in some teleosts. Phylogenies in some previous studies suggest IFN-γ-rel could be an ancient lineage, lost from other vertebrates (24–26). Not all phylogenies support this however (27), and it has also been suggested that IFN-γ-rel arose through duplication of IFN-γ during teleost evolution (19). Here, we tested this using a PRANK alignment of IFN-γ sequences spanning jawed vertebrate phylogeny, as well as the best-fitting substitution model, and the most closely related outgroup, the IL-10 family. This phylogenetic analysis maximally supported IFN-γ-rel as sister to teleost IFN-γ (Ultrafast Bootstrap [UFBOOT] = 100%) (Figure 3). Together with its absence outside of teleosts, this indicates that IFN-γ-rel resulted from a teleost-specific tandem gene duplication.

### Divergence of Human IFN-λs and Convergent Intron Loss in IFN3 Evolution

**Figure 4:**
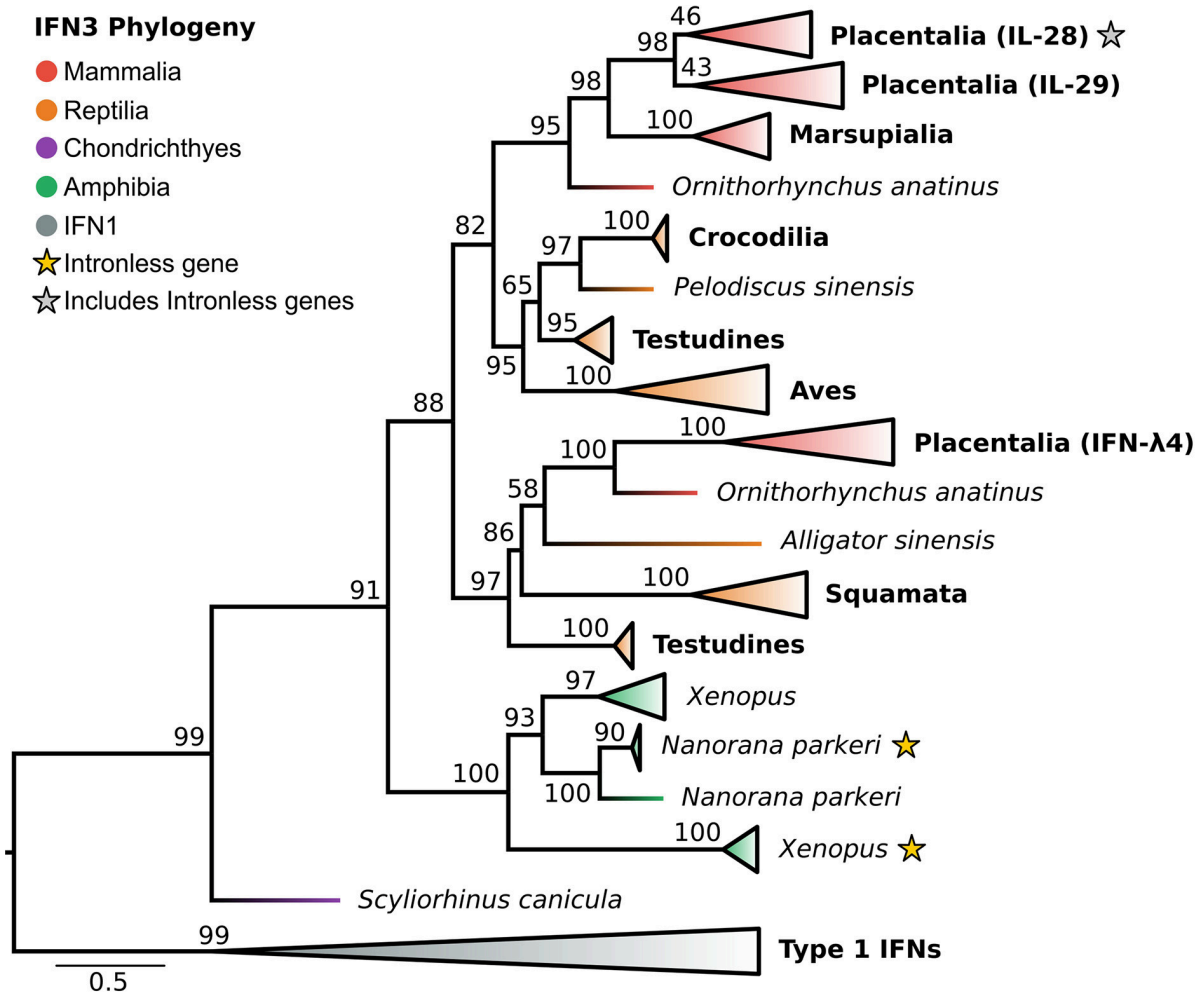
IFN3 phylogeny. Maximum likelihood consensus tree of the IFN3 genes under JTT+τ with IFN1s as outgroup. Clades are collapsed into major lineages and all ultrafast bootstrap support values are shown for non-collapsed portions of the tree.

In light of the newly discovered catshark IFN-λ, and identification of the IFN1 family as the closest outgroup, we reassessed the evolutionary history of the IFN3s (Figure 4 and Figure S4). Along with a selection of known tetrapod IFNs, BLAST analyses of genomes spanning vertebrate phylogeny revealed putative new amphibian and reptile IFN3 sequences, but consistent with previous studies failed to identify coelacanth or teleost IFN3s. Phylogenetic analysis of this dataset revealed that catshark IFN-λ falls sister to all tetrapod IFN3s (UFBOOT = 91%) (Figure 4 and Figure S4), while within tetrapods, amphibians and amniotes form separate sister clades (UFBOOT = 88%) (Figure 4 and Figure S4). Our analyses verified the presence of intronless IFN3s in amphibians (23). Strikingly however, we found that the intronless IFN3s of amphibians and mammals emerged independently, and that intronless IFN3s have evolved at least twice during amphibian evolution (UFBOOT = 100%) (Figure 4 and Figure S4), and hence at least three times throughout vertebrate evolution. Within amniotes, our results are largely consistent with those of Chen et al. (28), as we find that reptiles have at least two IFN3 lineages. In our analyses these lineages form clades with mammalian IFN-λ4 (though reptiles are not monophyletic) (UFBOOT = 97%) and mammalian IL-28/29 (UFBOOT = 82%), suggesting that they are orthologous, and that the IL-28/29 and IFN-λ4 lineages split in the amniote ancestor (Figure 4 and Figure S4). The reptile IL-28/29-like gene appears to have been duplicated in the ancestor of archelosaurians (turtles, birds, and crocodiles) (UFBOOT = 97%), while the human IL-28 and IL-29 lineages appear to have been duplicated in placental mammals (UFBOOT = 98%), with IL-28A and IL-28B later splitting during primate evolution (Figure 4 and Figure S4).

### Accounting for Phylogenetic Errors to Generate a Reliable IFN1 Tree

The evolutionary history of IFN1s has been studied intensively and many very different tree topologies generated. However, previous studies have not intentionally accounted for any of the major known sources of phylogenetic error. To help counter this we first applied two data-centric approaches designed to combat phylogenetic errors (43–45, 48–50, 94, 96). First, we applied only the closely related IFN3 as an outgroup, and increased taxon sampling by identifying new IFN1s from a dense sample of genomes across vertebrate phylogeny. This revealed hundreds of new IFN1 sequences (Table S2), which we subsampled prior to phylogenetic analyses, keeping only sequences above 100 amino acids in length (except for cartilaginous fishes and Japanese eel, where sequences of 50 or more amino acids were retained, given the paucity of data available for these species and their important evolutionary placement within jawed vertebrates and teleosts), and removing highly similar sequences within species from densely sampled lineages to reduce computational burden without negatively affecting deeper nodes in the tree.

Next, we tested the utility of site-heterogeneous phylogenetic mixture models to resolve the IFN1 phylogeny; such models have been shown to offer an improved fit to many datasets (42, 49, 95), including immune genes (41), as well as being more resistant to LBA artifacts (42) by accounting for evolutionary process variation among sites. As such we ran PhyloBayes analyses under the best-fitting standard model, JTT+Γ, as well as under a variety of site-heterogeneous mixture models (46, 91, 92, 99). Unfortunately, none of these runs reached convergence. However, this is not an uncommon occurrence in PhyloBayes analyses of difficult datasets, and can be remedied by identifying and removing error causing sequences or branches (49).

**Figure 5:**
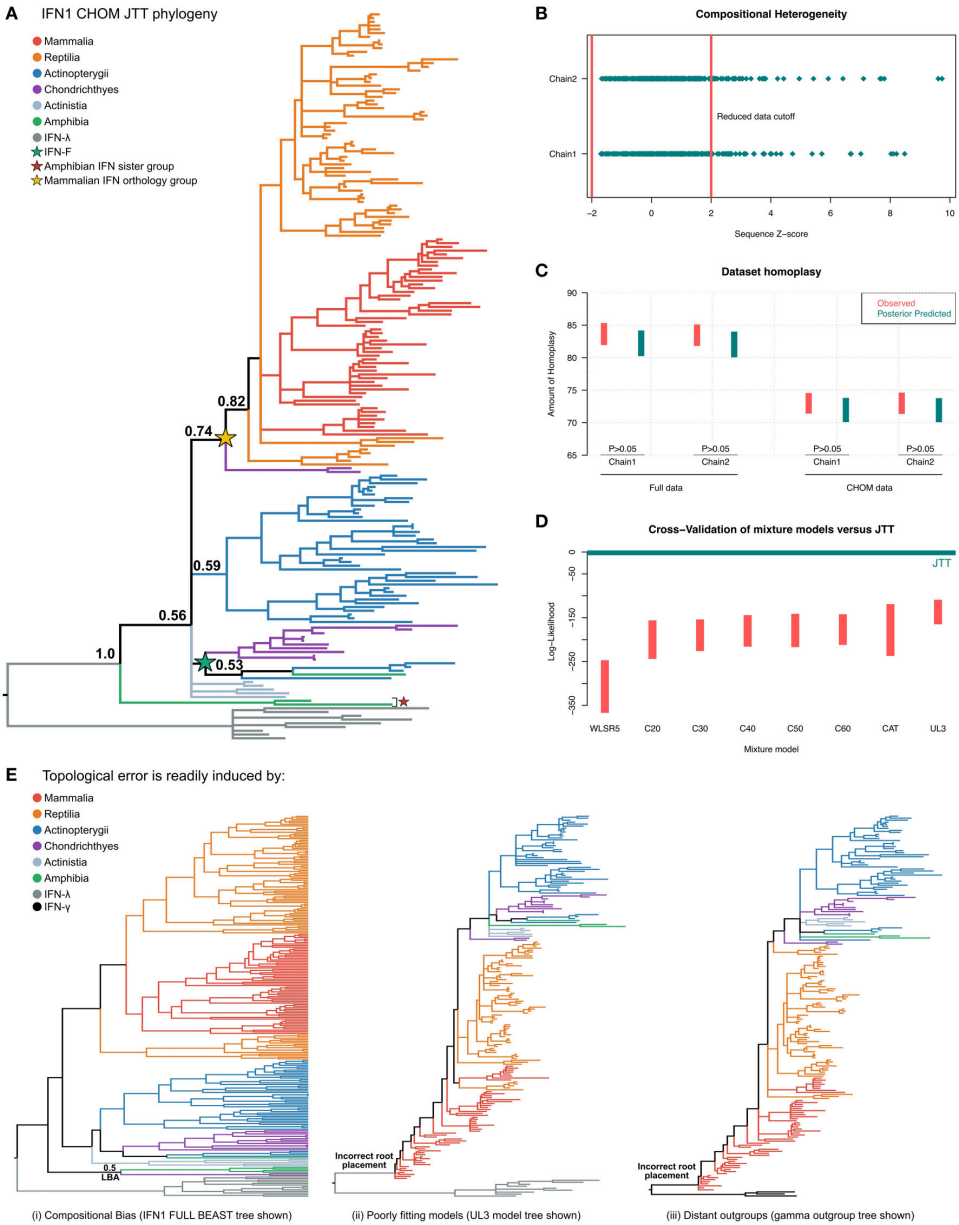
Phylogenetic investigation of IFN1 evolution. **(A)** Bayesian consensus tree of the CHOM IFN1 dataset under JTT+Γ. **(B)** Posterior predictive simulations (PPS) showed that ~50 IFN sequences introduced significant potential for compositional bias, which were removed to minimize branching artifacts (i.e., forming the CHOM dataset used for part **(A)**. **(C)** PPS also shows that JTT adequately predicts homoplasy in both the full and CHOM datasets. **(D)** Model selection via 10-fold Bayesian cross-validation indicates that the site-homogenous JTT model fits the data better than a range of site-heterogeneous mixture models. **(E)** IFN1 topology is highly sensitive to both dataset bias and methodological error: (i) the full (i.e., compositionally heterogeneous) dataset places the cartilaginous fish group otherwise identified as sister to amniote IFNs in a monophyletic group with the amphibian sequences that form the sister group to all other IFN1s (see also Figures S6, S7), while (ii) and (iii) show that less well fitting models (see also Figure S8) and distant outgroup taxa (full tree in Figure S9) result in evolutionarily irreconcilable root placement.

Interrogation of the MCMC chains for the JTT+Γ analyses revealed that effective sample sizes were sufficient, but that individual runs had become “stuck” at different log-likelihoods (the lesser of which must represent a local optimum), and at different tree topologies (i.e., PhyloBayes bpcomp “maxdiff = 1”). Trying to resolve this objectively, while also reducing systematic error, we used PPS to detect and remove sequences that deviated from the assumption of compositional heterogeneity (60). We then re-ran the phylogenetic analyses using this reduced “compositionally homogeneous” dataset (CHOM) and found that runs now converged for JTT+Γ (Figure 5A and Figure S5), as well as all the tested mixture models. Before examining the resultant tree topologies however, we sought to gain further understanding of the difference between analyzing the full and CHOM datasets, and to determine if the site-heterogeneous models might provide a better fit than JTT+Γ.

Interrogation of the compositional heterogeneity PPS results for the full dataset showed consistency between chains, with almost fully overlapping sets of sequences identified as biased in both chains, implying that sequence removal (i.e., identification of compositional bias) was not affected by lack of convergence (Figure 5B; Table S3), and therefore should be reliable.

An additional consideration is that removal of sequences may serve to lengthen branches in the phylogenetic tree, reducing the ability to detect hidden substitutions and increasing the potential of LBA artifacts. To determine if this was an issue we performed PPS analyses to test whether JTT+Γ could adequately accommodate homoplasy in both the full and CHOM datasets (42). While a greater level of homoplasy was both observed and predicted in the full dataset, this was adequately predicted by JTT+Γ (Figure 5C), implying that it should not be a major source of error (including topological inconsistency between chains), in either dataset.

Finally, using Bayesian cross-validation (42) we determined that JTT+Γ was in fact better-fitting than any of the tested mixture models (Figure 5D), perhaps due to the short alignment length, and so only this tree was used to make evolutionary inferences.

### Birth-Death Evolution of Multiple IFN1 Genes Since the Jawed Vertebrate Ancestor

Phylogenetic analyses of the CHOM dataset under JTT+Γ (Figure 5A and Figure S5) suggest a new paradigm for IFN1 evolution. The resultant tree indicates that duplication and loss events have occurred frequently since the origins of IFN1s (Figure 5A and Figure S5). This fits a rapid birth–death model of evolution (100, 101), as proposed for salmonid IFN1s (32), rather than the concerted evolution model (i.e., IFN1 expansions and contractions are confined to specific lineages) implied by many previous phylogenies. Multiple features of the tree topology support this scenario, including the presence of an amphibian lineage (red star in Figure 5A) that falls sister to all other IFN1s (PP = 0.65), intimating this lineage existed in the jawed vertebrate ancestor and has since been lost in other jawed vertebrates (Figure 5A; Figure S5). In addition, we identified a new IFN1 locus in elephant shark (Table S2), the intron-containing genes of which form the sister group to a clade (PP = 0.82) containing all amniote intronless interferons (PP = 0.74), suggesting that these genes are orthologous and that other jawed vertebrate classes have lost orthologs of this gene (Figure 5A and Figure S5). This means that the intron-containing IFN1s of teleosts and amphibians which gave rise to the idea that the retrotransposition event occurred in the amniote ancestor are in fact paralogous to this lineage, and as such are not informative on this point. Within the intronless amniote interferon clade, reptiles possess lineage-specific expansions comparable to those seen in mammals, and may retain ancient amniote IFN1s lost in mammals (Figure 5A). Elsewhere in the tree, we find that IFN-f, previously found only in teleosts, is likely also present in amphibians (PP = 0.74), and possibly cartilaginous fishes (PP = 0.53), where a lineage-specific expansion has occurred (green star in Figure 5A). Thus IFN-f appears to be an ancient jawed vertebrate IFN1 lineage, secondarily lost in amniotes. Importantly, this suggests that the ray-finned fish IFN1s may not be monophyletic and that the two groups defined by cysteine structures (9) may not have a phylogenetic basis (Figure 5A and Figure S5). Despite this, non-IFN-f ray-finned fish IFN1s group together (PP = 0.59), though, due to a polytomy, they do not appear to be identifiably orthologous to IFN1s from any other jawed vertebrate lineage (Figure 5A and Figure S5). The relationships of coelacanth IFN1s are similarly unresolved (Figure 5A and Figure S5). This result seems most consistent with both lineages representing lineage-specific expansions of ancient jawed vertebrate IFN1 genes lost in other jawed vertebrates, though the support for any scenario is low. Taken together, these results imply that several distinct IFN1 genes existed in the jawed vertebrate ancestor and have undergone rapid birth-death evolution since, meaning that ancient interferon genes are sometimes retained in only one or very few extant descendant taxa, while at the same time lineage-specific interferon expansions and contractions are common.

### Sensitivity of IFN1 Family to Phylogenetic Error

Major differences were observed between our results and those of previous studies. While we believe this is a result of improved methodology, we attempted to formally test this by performing experiments designed to exacerbate error potential in phylogenetic analyses (93). First, given that sequences displaying compositional bias contributed to non-convergence of PhyloBayes analyses, we instead built the full IFN1 phylogeny using alternative software (BEAST). This produced a similar topology to that obtained for the PhyloBayes CHOM analysis, however, the cartilaginous fish lineage that fell sister to the intronless amniote IFNs in the CHOM PhyloBayes analysis was instead placed sister to the amphibian sequences that fell sister to all other IFN1s (PP = 0.5) (Figure 5E and Figure S6). As the CHOM dataset does not deviate from the assumption of compositionally homogeneity, we considered this result to be an error induced by compositional bias. To further explore stability of this cartilaginous fish lineage in the CHOM dataset, we pruned sequences contributing to nearby branches to lengthen this branch, but this did not perturb its placement in the tree (Figure S7). Second, we examined tree topologies generated under the less well-fitting mixture models (Figure S8). Even for the second best-fitting model, UL3, this resulted in major issues with root placement (Figure 5E and Figure S8), suggesting an extremely non-parsimonious evolutionary scenario. Third, a similar outcome was observed when more distantly related IFN2 was applied as the outgroup instead of IFN3 (Figure 5E and Figure S9). Collectively, these results suggest that IFN1 phylogeny is highly sensitive to methodological and sampling errors.

### Intronless IFN1s Emerged in the Tetrapod Ancestor and Multiple Times in Amphibians

Since performing our IFN1 analyses, recent studies have identified new sequences not present in our dataset that may be relevant to IFN1 evolution. However, given the large compute time of performing all our PhyloBayes analyses (i.e., including cross-validation and PPS), it was not practical to rerun these with the addition of the new sequences (102). Instead, we decided upon a reasonable compromise; given that the best-fit model in PhyloBayes was a standard site-homogeneous model, we added the relevant sequences to our alignment and ran this under JTT+Γ in IQ-tree, with compositionally biased sequences (as identified by the χ^2^ test implemented in IQ-tree) removed.

**Figure 6:**
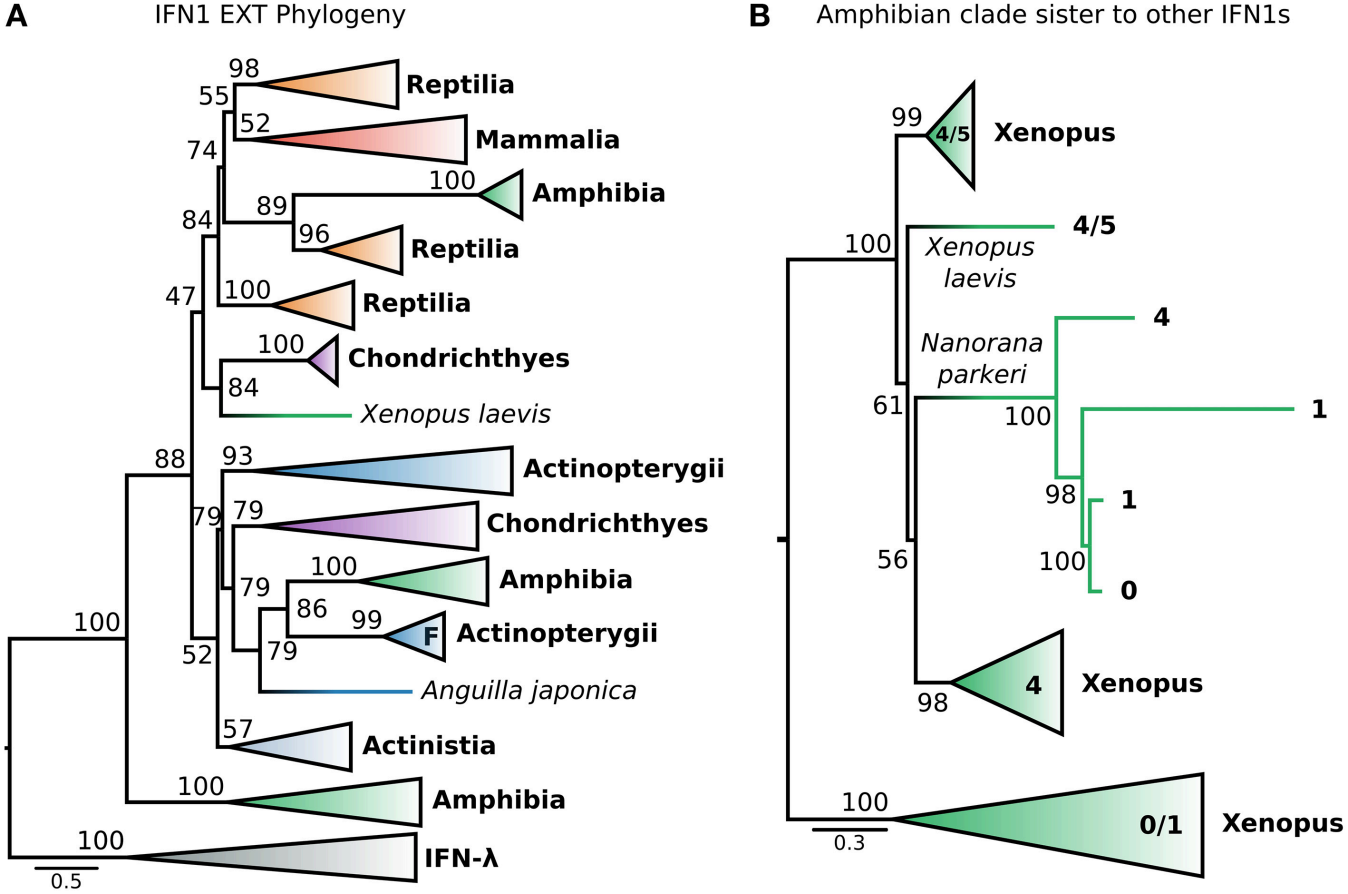
Extended IFN1 phylogeny reveals convergent evolution of intronless IFN1s. **(A)** Maximum likelihood consensus tree of the reduced, compositionally homogenous IFN1 dataset under JTT+τ, with additional compositionally homogeneous teleost and amphibian sequences from (23, 35, 40, 103). Clades are collapsed into major lineages and all ultrafast bootstrap supports in the visible tree are shown. The IFN-f lineage of salmonids is marked with an “F;” the closely related lone *Anguilla japonica* sequence is truncated, perhaps explaining its absence from the clade. **(B)** Maximum likelihood consensus tree, under JTT+τ, of the amphibian sister group of all other IFN1s showing two independent origins of intronless IFN1s within this clade.

This dataset (hereafter called EXT) included the recently identified fish IFN-h (103), as well as additional IFNs (both intron-containing and intronless) from amphibians. The EXT phylogenetic tree (Figure 6A and Figure S10) is generally consistent with that of the CHOM analysis, except that non-IFN-f ray-finned fish IFN1s, coelacanth IFN1s, and IFN-f form a weakly supported clade (UFBOOT = 52%) rather than a polytomy (i.e., PP < 0.5 in PhyloBayes analyses) (Figures 5A, 6A; Figures S5, S10). Within this clade, non-IFN-f ray-finned fish IFN1s fall sister to IFN-f (UFBOOT = 79%) with the coelacanth IFN1s being sister to both (Figure 6A and Figure S10). Because the IFN-f clade includes cartilaginous fish (UFBOOT = 79%) and amphibian (UFBOOT = 86%) sequences, this is consistent with non-IFN-f ray-finned fish IFN1s being the only surviving lineage of an interferon gene that was present in the jawed vertebrate ancestor (Figure 6A and Figure S10). A similar evolutionary scenario can thus be applied to coelacanth IFN1s, but support for this is weaker (UFBOOT = 52%) (Figure 6A and Figure S10). The newly included IFN-h falls within the clade of non IFN-f teleost IFN1s (UFBOOT = 93%), and as such does not alter the backbone IFN1 phylogeny (Figure 6A and Figure S10). Similarly, despite being placed differently in past analyses (23, 35), we find that almost all of the recently identified amphibian IFN1s (23, 35) fall into the clade of amphibian sequences (UFBOOT = 100%) that is sister to all other IFN1s (UFBOOT = 88%), in the CHOM analysis (Figures 5A, 6A; Figures S5, S10). Within this clade, the deepest split falls between intronless *Xenopus* IFN1s, and a clade containing intron-containing *Xenopus* and *Nanorana parkeri* sequences, as well as intronless *N. parkeri* sequences, confirming the recently discovered independent origins of intronless IFN1s in these species (40) (Figure 6B and Figure S11).

Strikingly, a small number of intronless amphibian IFN1s were nested within the mammal and reptile IFN1 clade, falling sister to a clade containing only reptile sequences (UFBOOT = 89%) (Figure 6A and Figure S10). This suggests that orthologs of amniote intronless IFN1s are present in amphibians and arose in the ancestor of tetrapods. Within this intronless tetrapod clade, two additional ancient reptile lineages are also present, one of which forms the sister group to all mammalian IFN1s (Figure 6A and Figure S10), while the other forms the sister to all other intronless IFN1s (i.e., both former reptile clades, and their mammalian and amphibian intronless counterparts) (UFBOOT ≥ 74%) (Figure 6A and Figure S10). This is consistent with a birth-death model of evolution, where reptiles have retained genes from three ancient intronless lineages that were present in the ancestor of tetrapods, but with amphibians and mammals retaining only one of these each, before the onset of independent lineage-specific diversifications. Intriguingly, an amphibian interferon containing a single intron falls sister to the group of cartilaginous fish IFN1s that were sister to the intronless amniote IFN1s in the CHOM analysis (UFBOOT = 84%) and together they fall sister to the intronless tetrapod IFN1s (UFBOOT = 47%). If accurate, this suggests that these cartilaginous fish genes are paralogous rather than orthologous to mammalian IFN1s, as both clades contain amphibians, further increasing the number of IFN1s likely present in the jawed vertebrate ancestor (Figure 6A and Figure S10).

### No One-to-One Orthology Relationships Between Mammal and Reptile IFN1s

**Figure 7:**
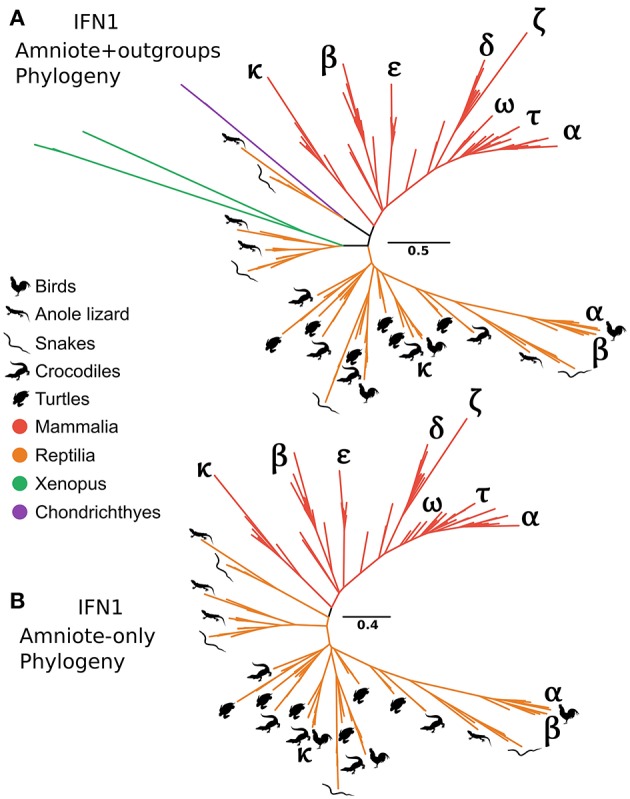
Tetrapod intronless IFN1 phylogenies. Maximum likelihood consensus tree of the tetrapod intronless IFN1s under JTT+Γ, including the sister group from the CHOM and EXT analyses **(A)**, and excluding the sister group (i.e., no outgroup sequences) as well as all amphibian sequences **(B)**.

It has long been recognized that the IFN-α and IFN-β genes of human and chicken are not orthologous (29). In contrast, the recently discovered chicken IFN-κ is purportedly an ortholog of mammalian IFN-κ (31). Interestingly, our CHOM and EXT IFN1 datasets, which greatly expanded taxon sampling in reptiles, failed to find evidence for orthology between IFN-κ genes of mammals and reptiles, but did not include the lineage containing chicken IFN-α because this was compositionally biased (Figures S5, S10; Table S3). Similarly, a lone amphibian sequence containing a single intron grouped together with the cartilaginous fish sequences that fall sister to the tetrapod intronless interferon clade. As this sequence would, more parsimoniously, be expected to group with the intronless IFN1s we performed more focused phylogenetic analyses to examine this finding. Our analyses included the cartilaginous fish and amphibian sequences that fell sister to this group in the EXT analysis (Figure 6A and Figure S10), but not more distantly related IFN1s to avoid biases introduced by distant outgroups. We also reinstated sequences, including chicken IFN-α, that were excluded from CHOM and EXT due to compositional bias. Interestingly, in this instance the amphibian sequence sister to cartilaginous fish in CHOM and EXT grouped with the intronless IFN1s of other amphibians (UFBOOT = 68%), away from the cartilaginous fish sequences (UFBOOT = 100%). This, far more parsimonious scenario, verifies the cartilaginous fish sequences as orthologs of the intronless tetrapod IFNs (Figure 7A and Figure S12). No evidence for orthology between any mammalian and reptile IFN1s was observed in this analysis. If rooted with the cartilaginous fish sequences, the results are also consistent with reptile genomes harboring ancient tetrapod intronless interferon lineages lost in mammals (Figure 7A and Figure S12). Finally, an unrooted analysis (i.e., excluding cartilaginous fish and amphibian sequences) recovered independent mammal and reptile clans, further supporting the lack of orthology between and reptile and mammalian IFNs (Figure 7B and Figure S12).

### Group 1, but Not Group 2, Ray-Finned Fish IFN1s Are Monophyletic

**Figure 8:**
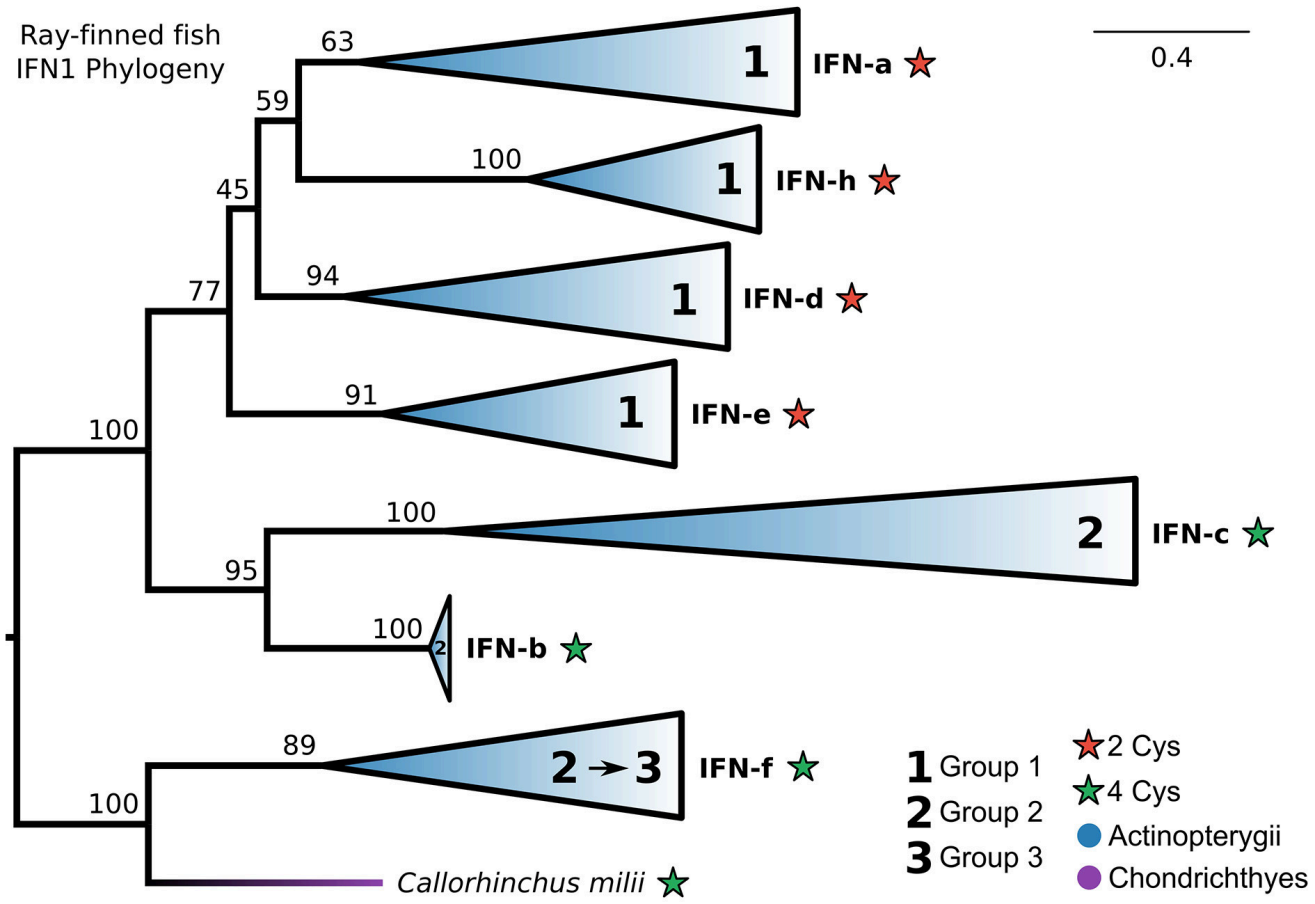
Phylogeny of ray-finned fish IFN1s. Maximum likelihood consensus tree under JTT+Γ with clades collapsed into major lineages and all ultrafast bootstrap support values are shown for non-collapsed portions of the tree. Conserved cysteine (Cys) counts in the mature peptide are shown for each clade, while clades are marked based on whether they were previously assigned to fish IFN1 group 1 or group 2. The root was placed between the IFN-f lineage, which is not specific to ray-finned fishes according to our complete IFN1 phylogenies (Figures 5A, 6A; Figures S5, S10), and all other lineages, and IFN-f has been reassigned to group 3 for this reason.

Our IFN1 phylogenies consistently showed that IFN-f is not a member of the ray-finned fish-specific IFN1s (Figures 5A, 6A; Figures S5, S10). This suggests that IFN1 classification based on conserved cysteine pairs may not have a phylogenetic basis. For example, group 2 IFNs (IFN-b, IFN-c, and IFN-f) do not form a clade despite all having two conserved cysteine pairs in the mature peptide. To better explore this, we performed a focused phylogenetic analysis (Figure 8 and Figure S13) of the remaining ray-finned fish-specific IFN1s that formed a clade in our CHOM and EXT analyses, using IFN-f as an outgroup. This placed the root between the remaining group 2 and group 1 members, in agreement with past hypotheses of fish IFN1 evolution, except for IFN-f (UFBOOT ≥ 77%) (Figure 8 and Figure S13). The group 2 members IFN-b and IFN-c, fell sister to each other (UFBOOT = 95%), while within group 1, IFN-a and IFN-h form a sister group (UFBOOT = 59%), with IFN-d (UFBOOT = 45%) and IFN-e (UFBOOT = 77%) forming successive sister groups (Figure 8 and Figure S13). Thus, our phylogenetic analyses reject the monophyly of group 2 (two pairs of conserved cysteines), due to the independent origins of IFN-f, but not of group 1 (one pair of conserved cysteines) ray-finned fish IFN1s.

## Discussion

The origins and evolutionary relationships between, and within, interferon subtypes have proven difficult to resolve. Here, with greatly increased taxon sampling and careful application of alignment and phylogenetic methodology, we overhaul our current understanding of the origins and relationships of the three IFN classes. Our findings also provide a significant step forward compared to previous work in understanding the mode and tempo of intra-class IFN evolution.

A notable study finding was our identification of a cartilaginous fish IFN3 gene, revealing that both IFN3 ligands and receptors existed in the jawed vertebrate ancestor, helping to resolve the deep relationships within the class II α-helical cytokines. We found that the four major lineages of this gene superfamily (i.e., IFN1, IFN2, IFN3, and the IL-10 family) diverged by multiple gene duplications [or genome duplication (15)] in quick succession in the ancestor of jawed vertebrates. We also revealed that the antiviral interferons, IFN1 and IFN3, are likely sister groups, with IFN2 being sister to the IL-10 family, similar to the model proposed by Siupka et al. (15). These results reject both of the other proposed hypotheses of IFN3 origins; (i) that tetrapod IFN3 genes evolved from IFN1s (8, 10, 23) and (ii) that IFN3 is a member of the IL-10 family (which is based on structural homology) (14, 21, 22). Structural similarity between IFN3 and the IL-10 family can be explained if these features were ancestral within the class II α-helical cytokines and secondarily lost in the IFN1 and IFN2 lineages. Importantly, unraveling the early evolution of class II α-helical cytokines also allowed us to objectively choose the best outgroups to test ingroup relationships for each of the IFN classes for the first time. This, along with other improvements in phylogenetic approach, made it possible for us to resolve some of the discrepancies noted in previous studies.

Our findings corroborate the conserved nature of IFN2 genes (which are not predominantly antiviral interferons) compared to IFN1 and IFN3 (24, 27, 104). By incorporating the closest outgroup, including cartilaginous fish IFN-γ (18), and better accounting for insertions and deletions at the alignment stage (72), we found strong support for teleost-specific origins of IFN-γ-rel by tandem duplication as proposed previously (19). Thus we can now reject the possibility that this represents an ancestral jawed vertebrate gene that was lost in other groups (24–26). Applying a similar approach to IFN3 evolution, we were able to delineate the evolution of the major IFN3 gene lineages found in humans for the first time; with the IL-28/29 ancestor diverging from IFN-λ4 in the amniote ancestor, and the IL-28 and IL-29 lineages splitting in the ancestor of placental mammals.

Our results confirm that inferring the evolutionary relationships between IFN1 family members is difficult. IFN1 phylogeny is highly sensitive to several confounding factors, including model inadequacy, distant outgroups, and limited taxon sampling. The short length and rapid evolution of IFN1s may also have driven stochastic errors and resulted in some weakly supported branches in our phylogenetic trees. Importantly, we observed consistency in our analyses that were designed to minimize systematic error (i.e., applying best-fit models and outgroups, and exclusion of compositionally biased sequences), both of which are factors that may be indicative of accuracy, even in the face of weak support (105, 106). By accounting for phylogenetic error, and considering consistency across our datasets, we reconstructed a strongly supported scenario of IFN1 evolution where several IFN1 genes existed in the jawed vertebrate ancestor. These genes subsequently underwent extensive lineage-specific gene duplication and loss events. Central to this finding is our unprecedented taxon sampling, which allowed us to identify ancestral jawed vertebrate genes that have become very taxonomically confined due to multiple loss events. Our data imply that while IFN1s often undergo lineage-specific expansions, they can also be lost many times in parallel, generating extreme cases of “elusive” genes (i.e., genes which are difficult to detect because of recurrent loss or biases in generating assembled genomes) (107) and hidden paralogy (i.e., where differential loss results in paralogs presenting as orthologs) (108, 109). A key example of this is the discovery of intron-containing cartilaginous fish orthologs of intronless tetrapod IFN1s, which revealed that intron-containing IFN1s of ray-finned fishes are paralogous, rather than orthologous, to the intronless tetrapod IFN1s. This means that the retrotransposition event giving rise to intronless tetrapod IFN1s may have occurred as early as in the ancestor of bony fishes (indicating loss of intronless IFN1s from ray-finned fishes and coelacanth), or as late as in the most recent common ancestor of extant tetrapods (indicating loss of intron-containing IFN1s from ray-finned fishes and coelacanth). Either way, this lineage, which is remarkably expanded in amniotes, has been lost from teleosts and coelacanth. Together these findings imply that IFN1 molecules, like some other immune genes, evolve via a rapid birth-death evolutionary process, and have done so at least since the jawed vertebrate ancestor (100, 101, 110). This is consistent with a scenario where IFN1 genes have maintained their antiviral function for over 450 million years by evolving rapidly, in terms of both substitutions and gene gain and loss, due to the host pathogen arms race with viruses.

Analyses focused on the evolution of ray-finned fish IFN1s revealed that their group 1 (one conserved cysteine pair), but not group 2 (two conserved cysteine pairs), interferons are monophyletic. Our findings suggest that group 2 should be split into two groups. The first consisting of IFN-b and IFN-c (together these form the sister group to group 1), for which we suggest the group 2 name be retained. And the second, consisting only of ray-finned fish IFN-f (although IFN-f appears to be present in at least amphibians and cartilaginous fishes also), which we propose be referred to as group 3. Interestingly, group 1 and group 2 IFN1s use different interferon receptors in zebrafish (11, 111), however zebrafish lack IFN-f, and as such it may be that IFN-f (now group 3) may have a different receptor to both group 1 and group 2. If this proved to be the case, analyses of receptor use may also help verify the assignment of amphibian and cartilaginous fish IFN-f. Importantly, although ray-finned fish group 1 and group 2 IFN1s are sister to each other, and seem to be derived from an ancestral jawed vertebrate IFN1 that has been lost in all other species, our results suggest that the ancestor of both groups possessed two conserved cysteine pairs. Based on the presence of the two conserved cysteine pairs across the IFN1 CHOM and EXT trees, our results are also consistent with the ancestral IFN1 possessing two disulphide bridges and four introns (9, 19, 32).

Similarly focusing on amniote IFN1 evolution we found that several intronless IFN1 genes existed in the tetrapod ancestor, with extensive IFN1 repertoires present in extant reptiles. In fact, as more ancestral tetrapod IFN1s appear to have been retained in reptiles, they evidently have even greater IFN1 diversity than mammals. Our analyses incorporated a greater breadth of mammals and reptiles than previous studies, including aquatic and/or semi-aquatic lineages, and had a more appropriate outgroup, but do not support one-to-one orthology of any mammalian or reptile IFN1s. This confirms non-orthology between human and chicken IFN-α and IFN-β (29, 112), while rejecting orthology of chicken and mammal IFN-κ (31).

Emergence of intronless interferons is more common in the IFN1 and IFN3 families than previously thought, consistent with intronless interferons bestowing an evolutionary advantage over those harboring introns (39). Our results suggest that both of the models (19, 23, 35, 40) put forth previously for the origins of amphibian intronless IFN1s are correct, with some emerging multiple times independently within amphibians, and others resulting from the same event that gave rise to amniote IFN1s. Strikingly, we also found that intronless amphibian IFN3s have emerged at least twice and independently from those of mammals on both occasions. Interestingly, amphibians also possess by far the most diverse set of IFN1s, including those which form part of the intronless tetrapod IFN1 group, the IFN-f group, and those in the sister group to all other IFN1s. Given this highly diverse repertoire of antiviral IFN1s and propensity for retrotransposition (or at least gross loss of introns), it is tempting to speculate a link to their morphology (e.g., permeable skin involved in terrestrial cutaneous respiration) or developmental life-history (e.g., aquatic tadpoles undergo metamorphosis to become terrestrial adults), especially as unique interferon responses have been observed between their distinctive stages of life (112, 113).

Lastly, our study indicates that a new nomenclature system is required to describe IFN1s to avoid relying on awkward (as applied here) or inaccurate descriptions. We have not attempted to formulate one here, as it is likely to be a substantial undertaking and will require input and agreement from several parties.

## Data Availability

All datasets generated and analyzed for the study are included in the manuscript and the Supplementary Files.

## Author Contributions

AR, JZ, and HD conceived the study. AR performed sequence similarity searches, designed and performed phylogenetic analyses, and drafted the manuscript and figures. JZ and HD performed IFN1 searches for cartilaginous fishes. All authors contributed to and approved study design and the final manuscript.

### Conflict of Interest Statement

The authors declare that the research was conducted in the absence of any commercial or financial relationships that could be construed as a potential conflict of interest.

## References

[1] Muller U Steinhoff U Reis LF Hemmi S Pavlovic J Zinkernagel RM. Functional role of type I and type II interferons in antiviral defense. Science. (1994) 264:1918–21. 10.1126/science.8009221 8009221

[2] Manry J Laval G Patin E Fornarino S Itan Y Fumagalli M. Evolutionary genetic dissection of human interferons. J Exp Med. (2011) 208:2747–59. 10.1084/jem.20111680 22162829 PMC3244034

[3] Pestka S Krause CD Sarkar D Walter MR Shi Y Fisher PB. Interleukin-10 and related cytokines and receptors. Annu Rev Immunol. (2004) 22:929–79. 10.1146/annurev.immunol.22.012703.104622 15032600

[4] Pestka S Krause CD Walter MR. Interferons, interferon-like cytokines, and their receptors. Immunol Rev. (2004) 202:8–32. 10.1111/j.0105-2896.2004.00204.x 15546383

[5] Zhang SY Boisson-Dupuis S Chapgier A Yang K Bustamante J Puel A. Inborn errors of interferon (IFN)-mediated immunity in humans: Insights into the respective roles of IFN-α/β, IFN-γ, and IFN-λ in host defense. Immunol Rev. (2008) 226:29–40. 10.1111/j.1600-065X.2008.00698.x 19161414

[6] Filipe-Santos O Bustamante J Chapgier A Vogt G de Beaucoudrey L Feinberg J. Inborn errors of IL-12/23- and IFN-γ-mediated immunity: molecular, cellular, and clinical features. Semin Immunol. (2006) 18:347–61. 10.1016/j.smim.2006.07.010 16997570

[7] Witte K Witte E Sabat R Wolk K. IL-28A, IL-28B, and IL-29: Promising cytokines with type I interferon-like properties. Cytokine Growth Factor Rev. (2010) 21:237–51. 10.1016/j.cytogfr.2010.04.002 20655797

[8] Fox BA Sheppard PO O'Hara PJ. The role of genomic data in the discovery, annotation and evolutionary interpretation of the interferon-lambda family. PLoS ONE. (2009) 4:e4933. 10.1371/journal.pone.0004933 19300512 PMC2654155

[9] Zou J Tafalla C Truckle J Secombes CJ. Identification of a second group of type I IFNs in fish sheds light on IFN evolution in vertebrates. J Immunol. (2007) 179:3859–71. 10.4049/jimmunol.179.6.3859 17785823

[10] Stein C Caccamo M Laird G Leptin M. Conservation and divergence of gene families encoding components of innate immune response systems in zebrafish. Genome Biol. (2007) 8:R251. 10.1186/gb-2007-8-11-r251 18039395 PMC2258186

[11] Levraud J-P Boudinot P Colin I Benmansour A Peyrieras N Herbomel P. Identification of the zebrafish IFN receptor: implications for the origin of the vertebrate IFN system. J Immunol. (2007) 178:4385–94. 10.4049/jimmunol.178.7.4385 17371995

[12] Lutfalla G Crollius HR Stange-Thomann N Jaillon O Mogensen K Monneron D. Comparative genomic analysis reveals independent expansion of a lineage-specific gene family in vertebrates: the class II cytokine receptors and their ligands in mammals and fish. BMC Genomics. (2003) 4:29. 10.1186/1471-2164-4-29 12869211 PMC179897

[13] Robertsen B. The interferon system of teleost fish. Fish Shellf Immunol. (2006) 20:172–91. 10.1016/j.fsi.2005.01.010 15939626

[14] Hamming OJ Lutfalla G Levraud J-P Hartmann R. Crystal structure of zebrafish interferons I and II reveals conservation of type I interferon structure in vertebrates. J Virol. (2011) 85:8181–7. 10.1128/jvi.00521-11 21653665 PMC3147990

[15] Siupka P Hamming OJ Frétaud M Luftalla G Levraud JP Hartmann R. The crystal structure of zebrafish IL-22 reveals an evolutionary, conserved structure highly similar to that of human IL-22. Genes Immun. (2014) 15:293–302. 10.1038/gene.2014.18 24833303

[16] Dehal P Boore JL. Two rounds of whole genome duplication in the ancestral vertebrate. PLoS Biol. (2005) 3:e314. 10.1371/journal.pbio.0030314 16128622 PMC1197285

[17] McLysaght A Hokamp K Wolfe KH. Extensive genomic duplication during early chordate evolution. Nat Genet. (2002) 31:200–4. 10.1038/ng884 12032567

[18] Venkatesh B Lee AP Ravi V Maurya AK Lian MM Swann JB. Elephant shark genome provides unique insights into gnathostome evolution. Nature. (2014) 505:174–9. 10.1038/nature12826 24402279 PMC3964593

[19] Secombes CJ Zou J. Evolution of interferons and interferon receptors. Front Immunol. (2017) 8:209. 10.3389/fimmu.2017.00209 28303139 PMC5332411

[20] Commins S Steinke JW Borish L. The extended IL-10 superfamily: IL-10, IL-19, IL-20, IL-22, IL-24, IL-26, IL-28, and IL-29. J Allergy Clin Immunol. (2008) 121:1108–11. 10.1016/j.jaci.2008.02.026 18405958

[21] Gad HH Hamming OJ Hartmann R. The structure of human interferon lambda and what it has taught us. J Interf Cytokine Res. (2010) 30:565–71. 10.1089/jir.2010.0062 20712454

[22] Gad HH Dellgren C Hamming OJ Vends S Paludan SR Hartmann R. Interferon-lambda is functionally an interferon but structurally related to the interleukin-10 family. J Biol Chem. (2009) 284:20869–75. 10.1074/jbc.M109.002923 19457860 PMC2742852

[23] Sang Y Liu Q Lee J Ma W McVey DS Blecha F. Expansion of amphibian intronless interferons revises the paradigm for interferon evolution and functional diversity. Sci Rep. (2016) 6:29072. 10.1038/srep29072 27356970 PMC4928184

[24] Igawa D Sakai M Savan R. An unexpected discovery of two interferon gamma-like genes along with interleukin (IL)-22 and−26 from teleost: IL-22 and−26 genes have been described for the first time outside mammals. Mol Immunol. (2006) 43:999–1009. 10.1016/j.molimm.2005.05.009 16005068

[25] Swain B Basu M Lenka SS Das S Jayasankar P Samanta M. Characterization and inductive expression analysis of interferon gamma-related gene in the indian major carp, Rohu (*Labeo rohita*). DNA Cell Biol. (2015) 34:367–78. 10.1089/dna.2014.2656 25756860

[26] Fu JP Chen SN Zou PF Huang B Guo Z Zeng LB. IFN-γ in turtle: conservation in sequence and signalling and role in inhibiting iridovirus replication in chinese soft-shelled turtle pelodiscus sinensis. Dev Comp Immunol. (2014) 43:87–95. 10.1016/j.dci.2013.11.001 24239708

[27] Chen SN Huang B Zhang XW Li Y Zhao LJ Li N. IFN-γ and its receptors in a reptile reveal the evolutionary conservation of type II IFNs in vertebrates. Dev Comp Immunol. (2013) 41:587–96. 10.1016/j.dci.2013.07.002 23850722

[28] Chen SN Zhang XW Li L Ruan BY Huang B Huang WS. Evolution of IFN-λ in tetrapod vertebrates and its functional characterization in green anole lizard (Anolis carolinensis). Dev Comp Immunol. (2016) 61:208–24. 10.1016/j.dci.2016.04.004 27062970

[29] Hughes AL Roberts RM. Independent origin of IFN-alpha and IFN-beta in birds and mammals. J Interferon Cytokine Res. (2000) 20:737–9. 10.1089/10799900050116444 10954917

[30] Roberts RM Liu L Guo Q Leaman D Bixby J. The evolution of the type I interferons. J Interf Cytokine Res. (1998) 18:805–16. 10.1089/jir.1998.18.805 9809615

[31] Santhakumar D Iqbal M Nair V Munir M. Chicken IFN kappa: a novel cytokine with antiviral activities. Sci Rep. (2017) 7:2719. 10.1038/s41598-017-02951-2 28578423 PMC5457445

[32] Zou J Gorgoglione B Taylor NGH Summathed T Lee P-T Panigrahi A. Salmonids have an extraordinary complex type I IFN system: characterization of the IFN Locus in Rainbow Trout *Oncorhynchus mykiss* reveals two novel IFN subgroups. J Immunol. (2014) 193:2273–86. 10.4049/jimmunol.1301796 25080482

[33] Qi Z Nie P Secombes CJ Zou J. Intron-containing type I and type III IFN coexist in amphibians: refuting the concept that a retroposition event gave rise to type I IFNs. J Immunol. (2010) 184:5038–46. 10.4049/jimmunol.0903374 20357248

[34] Xu L Yang L Liu W. Distinct evolution process among type I interferon in mammals. Protein Cell. (2013) 4:383–92. 10.1007/s13238-013-3021-1 23636688 PMC4875548

[35] Gan Z Chen SN Huang B Hou J Nie P. Intronless and intron-containing type I IFN genes coexist in amphibian Xenopus tropicalis: Insights into the origin and evolution of type I IFNs in vertebrates. Dev Comp Immunol. (2017) 67:166–76. 10.1016/j.dci.2016.10.007 27780747

[36] Nei M Rooney AP. Concerted and birth-and-death evolution of multigene families. Annu Rev Genet. (2005) 39:121–52. 10.1146/annurev.genet.39.073003.112240 16285855 PMC1464479

[37] Gillespie D Carter W. Concerted evolution of human interferon alpha genes. J Interferon Res. (1983) 3:83–8. 10.1089/jir.1983.3.83 6842040

[38] Woelk CH Frost SDW Richman DD Higley PE Kosakovsky Pond SL. Evolution of the interferon alpha gene family in eutherian mammals. Gene. (2007) 397:38–50. 10.1016/j.gene.2007.03.018 17512142 PMC2174272

[39] Krause CD. Intron loss in interferon genes follows a distinct set of stages, and may confer an evolutionary advantage. Cytokine. (2016) 83:193–205. 10.1016/j.cyto.2016.04.018 27155818

[40] Li N Nie P Hou J Laghari ZA Chen SN Gan Z. Unique composition of intronless and intron-containing type I IFNs in the Tibetan Frog *Nanorana parkeri* provides new evidence to support independent retroposition hypothesis for type I IFN genes in amphibians. J Immunol. (2018) 201:3329–42. 10.4049/jimmunol.1800553 30389775

[41] Redmond AK Macqueen DJ Dooley H. Phylotranscriptomics suggests the jawed vertebrate ancestor could generate diverse helper and regulatory T cell subsets. BMC Evol Biol. (2018) 18:169. 10.1186/s12862-018-1290-2 30442091 PMC6238376

[42] Lartillot N Brinkmann H Philippe H. Suppression of long-branch attraction artefacts in the animal phylogeny using a site-heterogeneous model. BMC Evol Biol. (2007) 7(Suppl. 1):S4. 10.1186/1471-2148-7-S1-S4 17288577 PMC1796613

[43] Zwickl DJ Hillis DM. Increased taxon sampling greatly reduces phylogenetic error. Syst Biol. (2002) 51:588–98. 10.1080/10635150290102339 12228001

[44] Pick KS Philippe H Schreiber F Erpenbeck D Jackson DJ Wrede P. Improved phylogenomic taxon sampling noticeably affects nonbilaterian relationships. Mol Biol Evol. (2010) 27:1983–7. 10.1093/molbev/msq089 20378579 PMC2922619

[45] Philippe H Delsuc F Brinkmann H Lartillot N Phylogenomics. Annu Rev Ecol Evol Syst. (2005) 36:541–62. 10.1146/annurev.ecolsys.35.112202.130205

[46] Lartillot N Philippe H. A Bayesian mixture model for across-site heterogeneities in the amino-acid replacement process. Mol Biol Evol. (2004) 21:1095–109. 10.1093/molbev/msh112 15014145

[47] Pisani D. Identifying and removing fast-evolving sites using compatibility analysis: an example from the arthropoda. Syst Biol. (2004) 53:978–89. 10.1080/10635150490888877 15764565

[48] Rota-Stabelli O Telford MJ. A multi criterion approach for the selection of optimal outgroups in phylogeny: recovering some support for Mandibulata over Myriochelata using mitogenomics. Mol Phylogenet Evol. (2008) 48:103–11. 10.1016/j.ympev.2008.03.033 18501642

[49] Pisani D Pett W Dohrmann M Feuda R Rota-Stabelli O Philippe H Genomic data do not support comb jellies as the sister group to all other animals. Proc Natl Acad Sci USA. (2015) 112:201518127 10.1073/pnas.1518127112 PMC4687580 26621703

[50] Feuda R Rota-Stabelli O Oakley TH Pisani D. The comb jelly opsins and the origins of animal phototransduction. Genome Biol Evol. (2014) 6:1964–71. 10.1093/gbe/evu154 25062921 PMC4159004

[51] Redmond AK Pettinello R Dooley H. Outgroup, alignment and modelling improvements indicate that two TNFSF13-like genes existed in the vertebrate ancestor. Immunogenetics. (2017) 69:187–92. 10.1007/s00251-016-0967-1 28070614 PMC5316386

[52] Drummond AJ Ho SYW Phillips MJ Rambaut A. Relaxed phylogenetics and dating with confidence. PLoS Biol. (2006) 4:699–710. 10.1371/journal.pbio.0040088 16683862 PMC1395354

[53] Kümmel Tria FD Landan G Dagan T Phylogenetic rooting using minimal ancestor deviation. Nat Ecol Evol. (2017) 1:193 10.1038/s41559-017-0193 29388565

[54] Redmond AK Ohta Y Criscitiello MF Macqueen DJ Flajnik MF Dooley H. Haptoglobin is a divergent MASP family member that neofunctionalized to recycle hemoglobin via CD163 in mammals. J Immunol. (2018) 201:2483–91. 10.4049/jimmunol.1800508 30194112 PMC6179929

[55] Williams TA Heaps SE Cherlin S Nye TMW Boys RJ Embley TM. New substitution models for rooting phylogenetic trees. Philos Trans R Soc Lond B Biol Sci. (2015) 370:20140336. 10.1098/rstb.2014.0336 26323766 PMC4571574

[56] Foster PG Hickey DA. Compositional bias may affect both DNA-based and protein-based phylogenetic reconstructions. J Mol Evol. (1999) 48:284–90. 10.1007/PL00006471 10093217

[57] Foster PG Jermiin LS Hickey DA. Nucleotide composition bias affects amino acid content in proteins coded by animal mitochondria. J Mol Evol. (1997) 44:282–8. 10.1007/PL00006145 9060394

[58] Blanquart S Lartillot N. A site- and time-heterogeneous model of amino acid replacement. Mol Biol Evol. (2008) 25:842–58. 10.1093/molbev/msn018 18234708

[59] Blanquart S Lartillot N. A Bayesian compound stochastic process for modeling nonstationary and nonhomogeneous sequence evolution. Mol Biol Evol. (2006) 23:2058–71. 10.1093/molbev/msl091 16931538

[60] Foster PG. Modeling compositional heterogeneity. Syst Biol. (2004) 53:485–95. 10.1080/10635150490445779 15503675

[61] Criscuolo A Gribaldo S. BMGE (Block Mapping and Gathering with Entropy): a new software for selection of phylogenetic informative regions from multiple sequence alignments. BMC Evol Biol. (2010) 10:210. 10.1186/1471-2148-10-210 20626897 PMC3017758

[62] Gouy R Baurain D Philippe H. Rooting the tree of life: the phylogenetic jury is still out. Philos Trans R Soc B Biol Sci. (2015) 370:20140329. 10.1098/rstb.2014.0329 26323760 PMC4571568

[63] Feuda R Dohrmann M Pett W Philippe H Rota-Stabelli O Lartillot N. Improved modeling of compositional heterogeneity supports sponges as sister to all other animals. Curr Biol. (2017) 27:3864–70.e4. 10.1016/j.cub.2017.11.008 29199080

[64] Feuda R Hamilton SC McInerney JO Pisani D Metazoan opsin evolution reveals a simple route to animal vision. Proc Natl Acad Sci USA. (2013) 110:7097 10.1073/pnas.1305990110 23112152 PMC3503164

[65] Gerts EM Yu YK Agarwala R Schäffer AA Altschul SF Composition-based statistics and translated nucleotide searches: improving the TBLASTN module of BLAST. BMC Biol. (2006) 4:41 10.1186/1741-7007-4-41 17156431 PMC1779365

[66] Altschul SF Madden TL Schäffer AA Zhang J Zhang Z Miller W. Gapped BLAST and PSI-BLAST: a new generation of protein database search programs. Nucleic Acids Res. (1997) 25:3389–402. 10.1093/nar/25.17.3389 9254694 PMC146917

[67] Hara Y Yamaguchi K Onimaru K Kadota M Koyanagi M Keeley SD. Shark genomes provide insights into elasmobranch evolution and the origin of vertebrates. Nat Ecol Evol. (2018) 2:1761–71. 10.1038/s41559-018-0673-5 30297745

[68] Mulley JF Hargreaves AD Hegarty MJ Heller RS Swain MT. Transcriptomic analysis of the lesser spotted catshark (Scyliorhinus canicula) pancreas, liver and brain reveals molecular level conservation of vertebrate pancreas function. BMC Genomics. (2014) 15:1074. 10.1186/1471-2164-15-1074 25480530 PMC4362833

[69] King BL Gillis JA Carlisle HR Dahn RD. A natural deletion of the HoxC cluster in elasmobranch fishes. Science. (2011) 334:1517. 10.1126/science.1210912 22174244 PMC3264428

[70] Solovyev V Statistical approaches in eukaryotic gene prediction. In: Balding DJ Bishop M Cannings C, editors. Handbook of Statistical Genetics. Hoboken, NJ: Wiley (2007). p. 1616.

[71] Kelley LA Mezulis S Yates CM Wass MN Sternberg MJE. The Phyre2 web portal for protein modeling, prediction and analysis. Nat Protoc. (2015) 10:845–58. 10.1038/nprot.2015.053 25950237 PMC5298202

[72] Löytynoja A Goldman N. Phylogeny-aware gap placement prevents errors in sequence alignment and evolutionary analysis. Science. (2008) 320:1632–5. 10.1126/science.1158395 18566285

[73] Penn O Privman E Ashkenazy H Landan G Graur D Pupko T. GUIDANCE: A web server for assessing alignment confidence scores. Nucleic Acids Res. (2010) 38:W23–8. 10.1093/nar/gkq443 20497997 PMC2896199

[74] Penn O Privman E Landan G Graur D Pupko T. An alignment confidence score capturing robustness to guide tree uncertainty. Mol Biol Evol. (2010) 27:1759–67. 10.1093/molbev/msq066 20207713 PMC2908709

[75] Katoh K Standley DM. MAFFT multiple sequence alignment software version 7: improvements in performance and usability. Mol Biol Evol. (2013) 30:772–80. 10.1093/molbev/mst010 23329690 PMC3603318

[76] Nguyen LT Schmidt HA Von Haeseler A Minh BQ. IQ-TREE: A fast and effective stochastic algorithm for estimating maximum-likelihood phylogenies. Mol Biol Evol. (2015) 32:268–74. 10.1093/molbev/msu300 25371430 PMC4271533

[77] Kalyaanamoorthy S Minh BQ Wong TKF von Haeseler A Jermiin LS. ModelFinder: fast model selection for accurate phylogenetic estimates. Nat Methods. (2017) 14:587–9. 10.1038/nmeth.4285 28481363 PMC5453245

[78] Minh BQ Nguyen MAT Von Haeseler A. Ultrafast approximation for phylogenetic bootstrap. Mol Biol Evol. (2013) 30:1188–95. 10.1093/molbev/mst024 23418397 PMC3670741

[79] Zou J Redmond AK Qi Z Dooley H Secombes CJ. The CXC chemokine receptors of fish: insights into CXCR evolution in the vertebrates. Gen Comp Endocrinol. (2015) 215:117–31. 10.1016/j.ygcen.2015.01.004 25623148

[80] Pettinello R Redmond AK Secombes CJ Macqueen DJ Dooley H. Evolutionary history of the T cell receptor complex as revealed by small-spotted catshark (Scyliorhinus canicula). Dev Comp Immunol. (2017) 74:125–35. 10.1016/j.dci.2017.04.015 28433528

[81] Drummond AJ Suchard MA Xie D Rambaut A. Bayesian phylogenetics with BEAUti and the BEAST 1.7. Mol Biol Evol. (2012) 29:1969–73. 10.1093/molbev/mss075 22367748 PMC3408070

[82] Yule U A mathematical theory of evolution, based on the conclusions of II. - a mathematical theory of evolution, based on the conclusions of Dr. J. C. Willis, F.R.S. Source Philos Trans R Soc London Ser B. (1925) 213:21–87. 10.2307/92117

[83] Gernhard T. The conditioned reconstructed process. J Theor Biol. (2008) 253:769–78. 10.1016/j.jtbi.2008.04.005 18538793

[84] Rambaut A Drummond AJ Xie D Baele G Suchard MA. Posterior summarization in Bayesian phylogenetics using tracer 1.7. Syst Biol. (2018) 67:901–4. 10.1093/sysbio/syy032 29718447 PMC6101584

[85] Calvignac-Spencer S Schulze JM Zickmann F Renard BY. Clock rooting further demonstrates that guinea 2014 EBOV is a member of the zaïre lineage. PLoS Curr. (2014) 6:1–9. 10.1371/currents.outbreaks.c0e035c86d721668a6ad7353f7f6fe86 24987574 PMC4073806

[86] Lartillot N Lepage T Blanquart S. PhyloBayes 3: a Bayesian software package for phylogenetic reconstruction and molecular dating. Bioinformatics. (2009) 25:2286–8. 10.1093/bioinformatics/btp368 19535536

[87] Li R Redmond AK Wang T Bird S Dooley H Secombes CJ. Characterisation of the TNF superfamily members CD40L and BAFF in the small-spotted catshark (Scyliorhinus canicula). Fish Shellfish Immunol. (2015) 47:381–9. 10.1016/j.fsi.2015.09.033 26386192

[88] Mukherjee K Korithoski B Kolaczkowski B. Ancient origins of vertebrate-specific innate antiviral immunity. Mol Biol Evol. (2014) 31:140–53. 10.1093/molbev/mst184 24109602 PMC3879448

[89] Jones DT Taylor WR Thornton JM. The rapid generation of mutation data matrices from protein sequences. Bioinformatics. (1992) 8:275–82. 10.1093/bioinformatics/8.3.275 1633570

[90] Yang Z. Among-site rate variation and its impact on phylogenetic analyses. Trends Ecol Evol. (1996) 11:367–72. 10.1016/0169-5347(96)10041-0 21237881

[91] Le SQ Gascuel O Lartillot N Empirical profile mixture models for phylogenetic reconstruction. Bioinformatics. (2008) 24:2317–23. 10.1093/bioinformatics/btn445 18718941

[92] Wang H-C Li K Susko E Roger AJ Jones D Taylor W. A class frequency mixture model that adjusts for site-specific amino acid frequencies and improves inference of protein phylogeny. BMC Evol Biol. (2008) 8:331. 10.1186/1471-2148-8-331 19087270 PMC2628903

[93] Campbell LI Rota-Stabelli O Edgecombe GD Marchioro T Longhorn SJ Telford MJ. MicroRNAs and phylogenomics resolve the relationships of Tardigrada and suggest that velvet worms are the sister group of Arthropoda. Proc Natl Acad Sci USA. (2011) 108:15920–4. 10.1073/pnas.1105499108 21896763 PMC3179045

[94] Li C Matthes-Rosana KA Garcia M Naylor GJP. Phylogenetics of *Chondrichthyes* and the problem of rooting phylogenies with distant outgroups. Mol Phylogenet Evol. (2012) 63:365–73. 10.1016/j.ympev.2012.01.013 22300842

[95] Domman D Horn M Embley TM Williams TA Plastid establishment did not require a chlamydial partner. Nat Commun. (2015) 6:6421 10.1038/ncomms7421 25758953 PMC4374161

[96] Philippe H Brinkmann H Lavrov DV Littlewood DTJ Manuel M Wörheide G. Resolving difficult phylogenetic questions: why more sequences are not enough. PLoS Biol. (2011) 9:e1000602. 10.1371/journal.pbio.1000602 21423652 PMC3057953

[97] Hamming OJ Terczynska-Dyla E Vieyres G Dijkman R Jørgensen SE Akhtar H. Interferon lambda 4 signals via the IFNλ receptor to regulate antiviral activity against HCV and coronaviruses. EMBO J. (2013) 32:3055–65. 10.1038/emboj.2013.232 24169568 PMC3844954

[98] Dijkstra JM T H 2 and T reg candidate genes in elephant shark. Nature. (2014) 511:E7–9. 10.1038/nature13446 25008534

[99] Le SQ Lartillot N Gascuel O. Phylogenetic mixture models for proteins. Philos Trans R Soc Lond B Biol Sci. (2008) 363:3965–76. 10.1098/rstb.2008.0180 18852096 PMC2607422

[100] Nei M Gu X Sitnikova T. Evolution by the birth-and-death process in multigene families of the vertebrate immune system. Proc Natl Acad Sci USA. (1997) 94:7799–806. 10.1073/pnas.94.15.7799 9223266 PMC33709

[101] Thomas JH. Rapid birth-death evolution specific to xenobiotic cytochrome P450 genes in vertebrates. PLoS Genet. (2007) 3:720–8. 10.1371/journal.pgen.0030067 17500592 PMC1866355

[102] Philippe H de Vienne DM Ranwez V Roure B Baurain D Delsuc F Pitfalls in supermatrix phylogenomics. Eur J Taxon. (2017) 283:1–25. 10.5852/ejt.2017.283

[103] Ding Y Ao J Huang X Chen X. Identification of two subgroups of type I IFNs in perciforme fish large yellow croaker *Larimichthys crocea* provides novel insights into function and regulation of fish type I IFNs. Front Immunol. (2016) 7:343. 10.3389/fimmu.2016.00343 27656183 PMC5013148

[104] Savan R Ravichandran S Collins JR Sakai M Young HA. Structural conservation of interferon gamma among vertebrates. Cytokine Growth Factor Rev. (2009) 20:115–24. 10.1016/j.cytogfr.2009.02.006 19268624 PMC2755191

[105] Kumar S Filipski AJ Battistuzzi FU Kosakovsky Pond SL Tamura K. Statistics and truth in phylogenomics. Mol Biol Evol. (2012) 29:457–72. 10.1093/molbev/msr202 21873298 PMC3258035

[106] Philippe H Brinkmann H Copley RR Moroz LL Nakano H Poustka AJ. Acoelomorph flatworms are deuterostomes related to Xenoturbella. Nature. (2011) 470:255–8. 10.1038/nature09676 21307940 PMC4025995

[107] Hara Y Takeuchi M Kageyama Y Tatsumi K Hibi M Kiyonari H. Madagascar ground gecko genome analysis characterizes asymmetric fates of duplicated genes. BMC Biol. (2018) 16:40. 10.1186/s12915-018-0509-4 29661185 PMC5901865

[108] Kuraku S. Palaeophylogenomics of the vertebrate ancestor - Impact of hidden paralogy on hagfish and lamprey gene phylogeny. Integr Comp Biol. (2010) 50:124–9. 10.1093/icb/icq044 21558193

[109] Siu-Ting K Torres-Sánchez M San Mauro D Wilcockson D Wilkinson M Pisani D Inadvertent paralog inclusion drives artefactual topologies and timetree estimates in phylogenomics. Mol Biol Evol. (2019) 36:1344–56. 10.1093/molbev/msz067 30903171 PMC6526904

[110] Sabbagh A Marin J Veyssière C Lecompte E Boukouvala S Poloni ES. Rapid birth-and-death evolution of the xenobiotic metabolizing NAT gene family in vertebrates with evidence of adaptive selection. BMC Evol Biol. (2013) 13:62. 10.1186/1471-2148-13-62 23497148 PMC3601968

[111] Aggad D Mazel M Boudinot P Mogensen KE Hamming OJ Hartmann R. The two groups of zebrafish virus-induced interferons signal via distinct receptors with specific and shared chains. J Immunol. (2009) 183:3924–31. 10.4049/jimmunol.0901495 19717522

[112] Wendel ES Yaparla A Melnyk MLS Koubourli DV Grayfer L. Amphibian (Xenopus laevis) tadpoles and adult frogs differ in their use of expanded repertoires of type I and type III interferon cytokines. Viruses. (2018) 10:372. 10.3390/v10070372 30018186 PMC6070924

[113] Wendel ES Yaparla A Koubourli DV Grayfer L. Amphibian (Xenopus laevis) tadpoles and adult frogs mount distinct interferon responses to the Frog Virus 3 ranavirus. Virology. (2017) 503:12–20. 10.1016/j.virol.2017.01.001 28081430

