# Supplementary material for: Discovery of All Three Types in Cartilaginous Fishes Enables Phylogenetic Resolution of the Origins and Evolution of Interferons
Source: Front Immunol. 2019 Jul 12;10:1558. doi: 10.3389/fimmu.2019.01558 (PMC6640115; doi:10.3389/fimmu.2019.01558)
Supplement: Supplementary file 1 [file Data_Sheet_1.docx]

Discovery of all three types in cartilaginous fishes enables phylogenetic resolution of the origins and evolution of interferons

Anthony K. Redmond, Jun Zou, Christopher J. Secombes, Daniel J. Macqueen, Helen Dooley

Supplementary Data S1

Multiple Sequence Alignments:

1. **Class II α-helical cytokines:**

>gaga_L

M--VCYGVTIILVGT--------LGSLLVGAFPQKKSCSLSKQFPEQFEDIMLLTNRKC-

-------------NTRLFHRKWDIAEL-SVPDRITLVEAELDLTITVLTNPTTQRLAETC

QQPLAFLTQVQEDLRDC-------LLEASHQPSGKLRHWLQKLETA--------------

------KKKETAGCLEASAILHIFQVL-NDLR---

>IFNL_xen

M-----EIPIRLAAM--------MALLVVTAHPHRRHCHMSRRSVNEHEKSPFSDGIKC-

-------------YRKMLRQKPSVCDL-QASDRLILTLERVTLAVDVLTNMTESPLAKLL

SLPLTMLLSLEDDLKIC-------RSPLSDPPSEQLMPWLHHLKHF--------------

------REKVSSECVQDAVLLSLTQLLIEDIM---

>scca_ifnL

M-----GVVLLLVATFLAP---SLGSGRVRRSPGQDGCSLSRARLGDFHRSLGSSQEPG-

-------------HRQVLVSSEQLRSL-EVPERLLLVEAEFRLFIRMMQELETSELQNLT

KKVLEVLYQMCWNLGRC-------LPETKDQTPKQLKKFLRNLKHA--------------

-----GRLGRTKQYIQTNLML----LLNEHLS---

>IFN1_coelacanth

M---TLFCLLLLVSNGT-------FC----------QDKWYNKGLQYLEAGEFPLKCLE-

-------------GSAQLDVPRKVCKVLKGERRIELVHDTLEHISKTYSN-TSTNLKKFQ

N----VIHLESEELRAC-------LQERS--GNKS--RHFKETGNF--------------

-----LQRQNYSSCVRAMILQLI----Q-------

>IFNE1_ONMY

I---SF-CLVMTICTWN-------KT----------IGTWSNNTITMLQKHE-----VS-

-------------RGPQITFPDKQYRQVKADEQIAFISHTLNAIKKLYSG-KSTGVDKFM

N----DLYRQTSELDQC-------MKTRS--VNR----HFKFLKHF--------------

-----LKREDYSASIRTAHLQRLD--TK-------

>IFNA_chicken

M---AVLLLKALATTAS-------AC----NHLRP-QDTFSHDSLQLLRDPTLPQLCPQ-

-------------HNASCSFNDTILDTSQADKT---THDILQHLFKILSS-TPAQRQSLL

N----RIHRYTQHLEQC-------LDSSS--RTRTIKKHFSCLHTF--------------

------QDNDYSACVRLAWFLHIHNLTT-------

>IFN1_sqac

M---VFLLVLLPGTLSQ-------DC----QRLQL-LDNINNQALDALREGPIPLHCKT-

-------------ERTSLRAKSLDLHQLQTPDRIQIVHQTLRHLTKIYSNLGSVKVENFR

L----LLDRQLGELEEC-------VRKPS--RPRPIHKYFRKVEKF--------------

-----LKQKRFSDCIRAARLQQILFITRR------

>IFN1_leer

M---VLVCVLLSGSLSL-------GC----ERLQL-LQVLNTDTLGKLNEGHLPRQCVT-

-------------ERRSLKTKPLNLVKLTAQDRIQIVHQTLHHLRRIYSNLSSVTVEHFR

L----LLDRQFRELEVC-------VRKPS--RARTVRKYFRKLRKF--------------

-----LKLKGFSDCTRAAHLQQLLLIMRE------

>IFN1_cami

M---AVLFAMLCVSLTL-------GC----STLRL-QKILIATTLNTLDEGHVPRHCVA-

-------------VGAEQGIASPDLRLLQNNDRILLLHKTFQHLNKIFHNMKSVQVNHFR

E----LLVTQREVVKDC-------IQDSS--MLSTIHTYFRKLKKF--------------

-----LKQQRYSACIRMARLQQILILTKG------

>IFNA_human

M---ALVALLVLSCKSSC----SVGC----D---L-----PQ-THSLLAQRISLFSCLK-

-------------DRHDFGFPQEEF---QKAETIPVLHEMIQQIFNLFSKDSSALLDKFY

T----ELYQQLNDLEAC-------VIQGT--ETPAVRKYFQRITLY--------------

-----LKEKKYSPCVRARSFSLSTNLQS-------

>IFNB_human

M---TNIALLLCFSTTAL----SMSY----N---L-LGFLQR-SSNLLWQG-RLEYCLK-

-------------DRMNFDIPEEKQ---QKEDAALTIYEMLQNIFAIFRDSSSTIVENLL

A----NVYHQINHLKT------------L--EEKHLKRYYGRILHY--------------

-----LKAKEYSHCVRVRNFYFINRLTN-------

>IFNC1_ONMY

M---ILSAFLCLAQVCSV----PMPC----Q---L-QGQLVRITHNLLRDGNFPLECLQ-

-------------ENVFVAFPATAFTISLSSSGAMAIYETLKNIDTLFGDDLPTKLENFQ

N----IVYRQIEESK-C-------MMGSS--DYLGLKTYFGNIAAV--------------

-----LKEKNFSYCVRKYSLQFIEHNST-------

>IFN3_frog

T----LQTILLLVLIPIVQ---SQNC----KWLQPKQEYLNRQTLKTFEE--NPPECQY-

-------------DSIELPNIDEIYSISQMEEMVLAVRGVLNETMRFYMHHESMAWERFQ

Q----LLYYQINQLEAC-------IPETA--ENPTISDQYQALEQI--------------

-----LQEK-NTACIQSGNLQLVGQLARQRLLQRT

>IFN4_frog

M----LSVLLLLSLTSIVH---SQSC----KWLHPKQEYLNTQILKAFNE--MPLKCEE-

-------------HPTDLPNTESTYSVSQVEAGALAVREVLNETMRFYMHHESMAWERFQ

Q----LLYYQIHQLEAC-------VSQTE--ENDSISEEFNLLETM--------------

-----VLEKDNSACIHLRNLQQVLQLSRQRLLQRP

>IFN1_frog

M---SVSVLLLITLGSSGQ---PTKG----KDVYRTQLNINREVRTLLGNAIPYSECED-

-------------NWRFFRLSEFLKNMSEVPIGLLQ--ITIHQFSVIFTNLANSAMSKMQ

T----LLYWYSTSGD-----------YSGLKELTTIRRYFRKMLKY--------------

-----LMKKGYSRCVRDKVLLLVTWHTKKHLV---

>IFN2_frog

L---SLCVLFLTISIPRGQ---SLEC----NHIYMSQHHCNKEALKHLVNKMLHPNCKD-

-------------QWKNFRFPKFLDKMKQVPKVILL--EIVHESSKLFSQLVMSTISQLL

M----VLHKSSTDWAQC-------VASSTHKVPKIIKKYFGRMEAY--------------

-----LKKKGYSHCVINNLMKFVARHTKKDLF---

>IFNF1_ONMY

M--ATLNVLLCIIVFYPAV---YAKC----SAPKVQKYYLSQQTLNDLAEERLPRGCIP-

-------------EAERLRVQRPALPIEEGEK-VWTLRLAFQLASELFQNLTLVKLRDLQ

D----LLARQNMTYSEC-------VRDMSVHLNLPIKNYFKELEDF--------------

-----LSHERFSACVRASIISQAIRNAKKH-V---

>hosa_il10

-LLCCLVLLTG-VRASPGQGTQSENSCT----------------------------LRDL

RDAFSRVKTNLLL-KESLDFKGY--------LGCQALSEMIQFYLEVM-----PQ-A--E

NQNLKT---LRLRLRRCH---RFLPCENKSKAVEQVKNAFNKLQEKGIYKAMSEFDIFIN

YIEAYMTMKI----------------------RN-

>gaga_il10

---CCQALLLL-LAACTLPAHCLEPTCL----------------------------LREL

RVKFEEIKDIQLL-SSELEFKGT--------FGCQSVSEMLRFYTDVL-----PR-A--M

QTMLLG---LKATMRRCH---RFFTCEKRSKAIKQIKETFEKMDENGIYKAMGEFDIFIN

YIEEYLLMRR----------------------RK-

>cyca_il10

MILSALVMFLL-SDSAQCRRVDCKTDCC----------------------------LKEL

RFAYREIQN-PLL-DENVNINSP--------YGCHVMNEILRFYLDIL-----PT-A--V

QKIFQD---LKRDILKCR---NYFFCQNPFE-FASIKNSYEKMKEKGVYKAMGELDMLFK

YIEQYLASKR----------------------GK-

>hosa_il26

MLRCGLLLVTL-SLAIAKHKQSSTKSCP----------------------------LKAT

-IPEDRIKNIRLL-KKKTQFM----------KNCQFQEQLLSFFMEVF-----GQLQ--L

QGFVEDFHSLRQKLSHCI---SCASSAREMKSITRMKRIFYRIGNKGIYKAISELDILLS

WIKKLLESSQ-------------------------

>xetr_il26

MLLSVLYLVSL-HIFTCDTRLIPQKTCQ----------------------------FKEL

-FPKDNITDIQFL-TEELDFMAQ--------KNCNLRNNFLSFYIKFL-----EKPP--V

QEIIQDLMVIQDMLLHCK---KCDHKETGIKSLTELKIKIRQIGNKVLWKAISEMDTLLE

WIYEYVEEML-------------------------

>gaga_il26

------------------------------------------------------------

--------------------------------------------MKVF-----SHLG--M

-EVISAFRVLQENMNACL---PCAPSTRLTSAVKNIKKTFLKLGEKGVYKAINELDILLP

WIQAYIQTIV-------------------------

>cod_il22

MLFVCFAWLLC-SQHSSARPMSK----VLRN-----------------------ADKK--

----AQNTSLRLI-SQTLN---A--------EICCLHANILDFYLLVL-----QHPT--M

PRLKTDLRRISQDLSHCN-----VTHYQDHQNAVEFREKLITMGQRGITKAIGEIDILFS

YLQ---DFCV----------------------QN-

>hosa_il22

MTLATSCLLLL-AQGGAAAPISSH--CRLDK-----------------------SNLA--

----DNNTDVRLI-GEKLGVSMS--------ERCYLMKQVLNFTLEVL-----FQPY--M

QEVVPFLARLSNRLSTCH-----IEGDDLHRNVQKLKDTVKKLGESGEIKAIGELDLLFM

SLR---NACI-------------------------

>gag_il22

GVVFCCCLLLT-SPKGTGVVSNAHQACRLRK-----------------------INLS--

----DQDTDNRLI-GQQINIREN--------NRCYMMKRITEIIVKIL-----LYPY--A

EDVAQFLASLTSELSRCK-----YSGNREHKNLEEMKSKMKELGENGKNKAIGELDLLFD

YIE---NACT-------------------------

>hosa_il19

MLQC---VLWLL-ILCSVD---NH-RCLI-S-----------------------TDMHHI

EESFDTFPNVTIL-STLTIIKPL--------DVCCVTKNLLAFYVDVFKDHQEPNP----

----KILRKISKTLRQCQEQRQCHCRQEATNATRVIHDNYDQLEHAAAIKSLGELDVFLA

WINKNHEVMS----------------------SA-

>duck_il20

MLLCLYSVCWLS-LMPAAE---NKIPCRI-S-----------------------MSVTEI

RSGFDPIRTLSIL-SHPSKVKSS--------DRCCIIHNLFNFYMDVFKHCQTEDS----

----YINRKISRKLEQCHNQNKCLCGQESTEKFKQILANYEGLNTSAAIKSLGELDILLD

WMEKS------------------------------

>hosa_il20

MAFSLLSAFYLL-WTPSTG---LKTSCVI-A-----------------------TNLQEI

RNGFDGNIDIRIL-RRTSDTKPA--------NRCCLLRHLLRLYLDVFKNYQTPDH----

----YTLRKISKDLRLCHAHMTCHCGEEAMKKYSQILSHFEKLEQAAVVKALGELDILLQ

WMEET-----------------------------E

>fugu_il20

MSLCLLFLFLLLCLTKPAR---SQTSCSV-N-----------------------VNLEEL

RKHYDDEIGVKFL-DKSLDVQDG--------QRCCFLRLVLRFYVEVFRSYTSSQP----

----QDERILSKDMHKCH----CLCEEPTQKRVDALHQAFNQLEGKAARKAVGELDIILG

WLQDS-EQKS----------------------PN-

>hosa_il24

MQMVVLPCLLLLSQVSGAQ---GQEPCQVKG-----------------------VVPQKL

WEAFDNITSARLL-QQEVNVSDA--------ESCYLVHTLLEFYLKVFKNYHTVEV----

----RTLKSFSSQLQPSQENEMFSIRDSAHRRFLLFRRAFKQLDEAALTKALGEVDILLT

WMQKFYKL---------------------------

>dare_il26

MILIPFTLCALLW--SEGH---KQEECLKRE-----------------------IRLPMI

REMLDNKPFHRILKCKELNVPDF--------KR------VLEIYDEVFEKMWDELPT---

-QFIDYFKRLKGIMQNCATEGKPTQSRCAKEKLKKFEQTLMKLQPDGKTKALSEFHSVLL

WISSGMDRRK----------------------KKI

>hosa_G

--MKYYILAFQLIVLGSLG-------CYCQD-----------------------PYVKEA

----YFNAGHSFLNWKE----ES--------DRKIMQSQIVSFYFKLFKNFKDDQSI--Q

KSV----ETIKEDMNVFNSNKK---------KRD---D-FEKLTNYSVTKAIHELIQVMA

ELSPAATGKR----------------------KRS

>gaga_G

--MTCYNL-FVLVIMSSLN-------VQLQD---------------------------DI

----DFNSSHSIVNWTE----RN--------EKRIILSQIVSMYLEMLEN--TDKS---K

PHI----KHISEELYTLKNNVK---------KVK---D-IMDLAKLPMNKAANELFSILQ

KLVDPPSFKR----------------------KRS

>Catshark-IFNgamma

MLLACFIIGMVSVLSDSAR-------CAPLN-----------------------SLDEPL

----HFNINHHFTKYKQ----TP--------ESGIVLNVILRLYLDLFAHIKPQTGV--E

SQI----SIVANSLKECLNSRT---------ETNLLSD-LKELSKIKWEKAILELDGILT

IMKETGKRRR----------------------KRN

1. **IFN2:**

>XP_007903185.1_IFNG_Callorhinchus_milii

---------------M------K-------L--CFIIGL---VTC-------------LL

---L----LG--------------S----ASC--DR----------L-Q---DEINN--L

KS-HF-------D-----T-SSHEVADG-GPLF----L---NMLEK--W--KG-SPESGI

VFHRILLWYENFFKN-I--------------K--GAPGKNK---EL---------DISNI

--G----NLISK-WIVE-DRY--K-PVELKYDL-DKLDNIQWNKQLVQRKAVLELEMLLP

RM-------------N-VN--GRRRR----------------------------------

------------------------------------------------------------

--------RS---N----MG---------GRR-R-----SRT---

>XP_020383749.1_IFN_G_like_Rhincodon_typus

---------------M------I-------LLTCLIIGM---VSH-------------VL

---SD--STG--------------C----AAP--HS----------L-D---KQLTR--L

SQ-HF-------N-----I-NHPDVGNG-GAIF----T---NILKK--Y--KQ-IPEKSI

ILDAILHLYLDLFKV-L--------------M------QHN---EVKD-------EVSIV

--A----NSLNE-CMTS-ANY--KTQKELLSDL-KKLYNIKWDDQMVQRKAIIE------

------------------------------------------------------------

------------------------------------------------------------

---------------------------------------------

>ABN80441.1_G_IFN_Mus_musculus

-M---------NATHC------I-------L--ALQLFL---MA----------------

----V--S-G--------------C----YCH--GT----------V-I---ESLES--L

NN-YF-------N-----S-SGIDVEE--KSLF----L---DIWRN--W--QK-DGDMKI

LQSQIISFYLRLFEV-L--------------K------DNQ---AISN-------NISVI

--E----SHL-----IT-TFF--SNSKARKDAF-MSIAKFEVNNPQVQRQAFNELIRVV-

H----QLL----PESS-LR--KRKRS----------------------------------

------------------------------------------------------------

--------RC-----------------------------------

>ereu_G

-M---------KYAGY------I-------L--ALQLCV---ML----------------

----S--S-S--------------S----SCM--VG----------I-I---KEIDK--L

KE-YF-------N-----A-TSSSVASG-GTLF----L---DTMKK--W--KE-ESD-KI

IQSQVISFYFKLFES-V--------------Q------DNQ----IQS-------ALNSV

--K----EEL-----VV-KFF--NGNLSKREDF-EKLANISMNDGMVQRRAISELDNVI-

H----SLL----P--C-QR--KRKRSQTQFRGRRNNEYSVSSGRSGGNQGEKSEGYVVSL

KLAMITNLNKDIEYNEVPFLYILNNITQKQLSAFDFSLPTNGESNSEQAFEGLLLSSFCG

LFFLLHSQQCTIF--------------------------------

>NP_000610.2_IFN_G_Homo_sapiens

-M---------KYTSY------I-------L--AFQLCI---VL----------------

----G--SLG--------------C----YCQ--DP----------Y-V---KEAEN--L

KK-YF-------N-----A-GHSDVADN-GTLF----L---GILKN--W--KE-ESDRKI

MQSQIVSFYFKLFKN-F--------------K------DDQ---SIQK-------SVETI

--K----EDM-----NV-KFF--NSNKKKRDDF-EKLTNYSVTDLNVQRKAIHELIQVM-

A----ELS----PAAK-TG--KRKRS----------------------------------

------------------------------------------------------------

--------QM---L---FRG-----------R-R-----ASQ---

>oror_G

-M---------KYTSY------F-------L--AFQLCV---IL----------------

----G--SSG--------------S----YCQ--AP----------F-F---KEIQN--L

KE-YF-------N-----A-SNPDVAGG-GPLF----L---EILEN--W--KD-ESDKKI

IQSQIVSFYFKLFEN-L--------------K------GNQ---IIQR-------SMDII

--K----QDM-----FQ-KFL--NGSSEKLDDF-KKLIQIPVDDLQIQRKAISELIRVM-

K----DLS----PRSN-LR--KRRRS----------------------------------

------------------------------------------------------------

--------QN---L---FRG-----------Q-R-----ASK---

>peva_G

MM---------NYTSY------I-------L--AFQLCV---IL----------------

----G--SSS--------------C----YCQ--AT----------F-L---KEIEN--L

KE-YF-------N-----A-SNSNVADG-GNLF----L---DILKN--W--RE-ESDKKI

IQSQIVSFYFKLFEN-L--------------K------DNP---IIQS-------SVQII

--K----EDL-----RV-KFF--NSNNSKLEDF-KKVIQIPVNNQTVQRKAISELFKVM-

T----DLS----PKSN-QR--KRKRS----------------------------------

------------------------------------------------------------

--------QS---L---FRG-----------W-K-----A-----

>Loaf_G

-M---------NFTSY------I-------L--AFQLCI---IL----------------

----G--SSS--------------C----YCQ--AT----------F-L---KEIQN--L

KE-YL-------N-----A-TDSDVADG-GPLF----I---DILKN--W--KE-DSDKKI

IQSQIVSFYLKIFDN-L--------------K------DNQ---VIQE-------SVKTL

--E----EDL-----FV-KFF--NSSSSKRDDF-LKVMQTPVNDRNIQRKAISELAKVM-

N----DLS----HRST-GS--KRKRR----------------------------------

------------------------------------------------------------

--------QY---S---FRG-----------R-R-----ASE---

>dano_G

-M---------NYTGY------L-------L--AFQLCI---IL----------------

----G--SSS--------------C----YCQ--AD----------L-F---KEKYK--L

RN-IF-------N-----A-SDSDVADG-G-LF----L---DILKN--W--KE-ESDKKI

IQSQIVSVYFKIFDN-L--------------K------DNQ---IIQK-------SMATI

--K----EDL-----IA-KFF--NSSSSKLNDFPQKLIRTPVNDLKVQRKAVNELFKVMN

X----DLS----PKSN-LR--KRKRS----------------------------------

------------------------------------------------------------

--------QS---T---FHG-----------R-R-----ASI---

>oran_G

-M---------NYPAA------F-------C--VLQFGI---VIC--------------L

---SG--FAH--------------G----FSF--SL----------L-S---NEIDN--L

KE-YY-------N-----A-SEPDVAED-GPLF----V---MMLKD-----AQ-QDEKKV

ILSQIISMYLDIFST-L--------------E------DNH---LVKE-------SMGKI

--R----ESM-----VQ-WNQ--THGFKKLGDL-QKLIKTSVSDAKIQRKAVHELFWVFQ

NL--HNMT----ANSS-QR--KKRRS----------------------------------

------------------------------------------------------------

--------R----------------------Q-R-----ISKY--

>ABI83735.1_IFN_G_Gallus_gallus_murghi

-M---------TCQTYN-----L-------F--VL-SVI---MIY--------------Y

---GH--TAS--------------------SLNLVQ----------L-Q---DDIDK--L

KA-DF-------N-----S-SHSDVADG-GPII----V---EKLKN--W--TE-RNEKRI

ILSQIVSMYLEMLEN-T--------------D------KSK------P-------HIKHI

--S----EELYT---LK-NNL--PDGVKKVKDI-MDLAKLPMSDLRIQRKAANELFSILQ

KLV----D-----PPS-F---KRKRS----------------------------------

------------------------------------------------------------

--------QS---Q----RR---------CNC-------------

>gaga_G

-M---------TCQTYN-----L-------F--VL-SVI---MIY--------------Y

---GH--TAS--------------------SLNLVQ----------L-Q---DDIDK--L

KA-DF-------N-----S-SHSDVADG-GPII----V---EKLKN--W--TE-RNEKRI

ILSQIVSMYLEMLEN-T--------------D------KSK------P-------HIKHI

--S----EELYT---LK-NNL--PDGVKKVKDI-MDLAKLPMNDLRIQRKAANELFSILQ

KLV----D-----PPS-F---KRKRS----------------------------------

------------------------------------------------------------

--------QS---Q----RR---------CNC-------------

>coli_G

-M---------TCQTYS-----L-------F--VL-SVV---MIC--------------F

---GR--FGN--------------------SLILAH----------L-E---NDIDQ--L

KS-DF-------N-----S-SNSDVADG-GPIF----T---GKLTD--W--TE-RNEKRI

ILSQIVSMYLEMLEK-S--------------D------KSK------A-------HVRHI

--S----EELYT---LK-NSL--PDGLKKLQDL-KDLAKLQMNDLKTQRKAVHELFSVLQ

KLV----D----TPAS-L---KRKRS----------------------------------

------------------------------------------------------------

--------QF---Q----RR---------CKC-------------

>fape_G

-M---------TCQTYS-----L-------F--VL-SVI---MIC--------------F

---GR--FGN--------------------SSILAQ----------L-E---NDIDQ--L

KA-DF-------N-----S-SNSDVADG-GPIF----V---DKLTN--W--TE-RNEKRI

ILSQIVSMYLEMLEK-T--------------D------KSK------A-------HIRHI

--S----EELYT---LK-NSL--PDGLKKMKDL-MDLTKLQMSDLKIQRKAANELFSVLQ

KL-----E----PSAP-L---KRKRS----------------------------------

------------------------------------------------------------

--------QF---Q----KK---------CKC-------------

>caan_G

-M---------TCQTYS-----L-------F--AL-SVI---MIY--------------F

---GH--FGN--------------------SLILPQ----------L-Q---KDIDQ--L

KD-DF-------N-----S-SHSDVAEG-GPIF----T---GKLKN--W--TE-RNEKRI

ILSQIVPMYLKMLEN-T--------------D------KSK------D-------HVRHI

--R----EELYT---LQ-ESL--SDGSRKMKDL-RDLEKLQMTDLKIQRKAVNELFSVLQ

KLA----E----TPAS-H---KRKRS----------------------------------

------------------------------------------------------------

--------QF---Q----RS---------CRC-W-----------

>Apfo_G

-M---------TCQTYS-----L-------F--VL-SVI---MIY--------------F

---GR--FGN--------------------SLILAQ----------L-Q---NDIDQ--L

KA-DF-------N-----S-SHSDVADG-GPIF----T---EKLIN--W--TE-RNEKRI

ILSQIVSMYLEMLEN-T--------------D------KSK------A-------HIRHI

--S----EELYT---LK-KSL--PDGLKKMKDL-MDLSKLQMSDLKIQRKAVNELFSVLQ

KLV----E----TSTS-I---KRKRS----------------------------------

------------------------------------------------------------

--------QS---Q----RR---------CKC-------------

>tyal_G

-M---------TCQTYS-----L-------F--VL-SVI---MIY--------------F

---GR--FGH--------------------TLILAQ----------L-Q---KDIDQ--L

KA-DF-------N-----S-SHSDVADG-GPIF----T---EKLIN--W--TE-RNEKRI

ILSQIVSMYLEMLEN-T--------------D------KSK------A-------HVRHI

--S----EELYT---LK-KSL--PDGLKKMKDL-MDLTNLQMTDLKIQRKAVNELFSVLQ

KLV----E----TSAS-L---KRKRS----------------------------------

------------------------------------------------------------

--------QS---Q----RR---------CKC-------------

>strca_G

-M---------TCQTYS-----L-------L--VL-SII---TIY--------------F

---GR--FGS--------------------SLILAN----------L-Q---NDIDK--L

KA-EF-------N-----S-SHSDVADG-GPIF----T---EKLKN--W--TE-RNEKRI

ILSQIVSMYLEMLEN-T--------------D------KSK------G-------HVRHI

--S----EELFT---LK-NSL--PDGLKKLKDI-MDLAKLQMSDLKIQRKAVNELFSVLQ

TLV----E----TPTS-F---KRKRS----------------------------------

------------------------------------------------------------

--------QS---Q----RR---------CKC-------------

>AFK09742.1_IFN_G_Pelodiscus_sinensis

-M---------TPQTY------F-------F--IL-LSI---SSY--------------F

---GC--VFG--------------------QSLLTQ----------I-E---DDIEK--L

KA-DF-------N-----S-NQSDVADG-GSIF----T---ERLKS--W--TE-ITEKKI

ILSQIVSLYLKMF-S-V--------------VPT----NGK------H-------HIVNI

--N----NALHT---LN-NNL--TDSFKKVKDL-MELSQLQMNDSKIQRKAVNELFPTLQ

KLLQ---D----HPAT-HN--KRKRS----------------------------------

------------------------------------------------------------

--------QS---QK---RK---------CRC-------------

>chmy_G

-M---------TPQIY------L-------F--IL-LSI---SSC--------------F

---GC--FFC--------------------QPILPK----------I-E---EDIEK--L

KA-DF-------N-----S-SQSDVADG-GSIF----T---ERLKS--W--TE-TTEKKI

ILSQIISMYLKMFEN-I--------------HPT----INK------TAIKQL--HVKNI

--R----NALYT---LN-NSL--TESFKKVNDL-MELARLPMNDLKIQRKAVNELFPTLQ

KLLL---D----HPTT-HV--KRKRS----------------------------------

------------------------------------------------------------

--------QS---QK---RK---------CRC-------------

>chpi_G

-M---------TPQIY------L-------F--IL-LSI---SSC--------------F

---GC--FFC--------------------QSILPK----------I-E---EDIEK--L

KA-DF-------N-----S-SQSDVAEG-GSIF----T---ERLKS--W--TE-ATEKKI

ILSQIVSMYLKMFDN-I--------------GPA----TSK------S-------HVKNI

--H----NALYT---LN-DSL--TESFKKVKDL-MELARLPMNDKKIQRKAVNELFPTLQ

KLLL---D----HPTT-HV--KRKRS----------------------------------

------------------------------------------------------------

--------QN---QK---RK---------CRC-------------

>almi_G

-M---------SSQAY------L-------F--IF-LSV--XLSC--------------L

---GC--LES--------------------TIIPFQ----------V-Q---SDIDK--L

KI-DF-------N-----S-SNSDVADG-GPIF----T---EKLKS--W--TE-VNERRI

LFSHIISLYLKMFEN-I--------------D------TSK------T-------HVRNV

--H----EYLLA---KK-SNL--ANDYKKINDI-MELAKLPMSDLKIQRKAINELFPLLQ

KL-----D----SPTA-RS--ERRRR----------------------------------

------------------------------------------------------------

--------QN---S----RG---------CKC-------------

>Gavga_G

-M---------TYQTY------L-------F--IF-LSI--HLSC--------------L

---GC--LES--------------------TIIPFQ----------V-Q---SDIDK--L

KI-DF-------N-----S-SNSDVADG-GPIF----T---EKLKS--W--TE-VNERRI

LFSHIISLYLKMFEN-I--------------D------TSK------A-------HIRNV

--H----EYLLA---KK-SNL--ANDYKKINDI-MELAKLPMSDLKIQRKAINELFPLLQ

KL-----D----SPTV-RS--ERRRR----------------------------------

------------------------------------------------------------

--------QN---S----RG---------CKC-------------

>Crpo_G

-M---------TYQTY------L-------F--IF-LSV--HLSC--------------L

---GC--LES--------------------TIIPFQ----------V-Q---SDIDK--L

KI-DF-------N-----S-SNSDVADG-GPIF----T---EKLKS--W--TE-VNERRI

LFSHIISLYLKMFES-I--------------D------TSK------A-------HIRNV

--H----EYLLA---KK-SNL--ANDYKKINDI-MELAKLPTSDLKIQRKAINELSPLLQ

KL-----D----SPTG-RS--ERRRR----------------------------------

------------------------------------------------------------

--------QN---P----RG---------CKC-------------

>AGU16998.1_IFN_G_Anolis_carolinensis

-M---------AWQIC------L-------I--IL-LAA---RSS--------------L

---GL--TSS--------------------TS--ER----------I-T---SAIES--L

QK-DF-------N-----A-TRSDVAEG-GPVF----T---KMLDSGLW--SQ-PNEKKI

LIAQIISKYVQMLNN-I--------------T------KTP------A-----PQYIKEL

--R----EALED---YK-KNY--NESLMKANDL-IHLAQLPMDNLRTQRKAVLEMTRVLQ

EV-----K----KEES-R---RRRRS----------------------------------

------------------------------------------------------------

--------QR---Q-NP-RG---------LKR-RMPNMG------

>ABU54059.1_IFN_G_Xenopus_tropicalis

-M----------RQYR------L-------L--SL-FVI---IYW--------------V

---GH--IHG--------------------SS--VN----------I-REASTATEE--L

RK-HF-------N-----K-INQDDDDSTGLIF----L---KLFDS--W--KE-EGEKKI

LLSQIVPVYLKMLDA-I--------------P------KIP---ELQA-------SIKNL

--K----MMLHTS--FE-DLL--KQSDQKLRGL-HELKKIQVGDVKTQHAAIKELFMILR

EL--SVME----QPKN-HVVKKRKLD----------------------------------

------------------------------------------------------------

--------FQ---Q----RN---------RKR-R-----NRLF--

>CAJ98867.1_IFN_G_1_Cyprinus_carpio

MY---------CWLNM------V------HL--ICALLL---IVS--------------L

---QG--TVGARLPQSQNDKE---Q----ML---KN----------L-R---EKIEP--L

QK-HY-------H-----T-TDKEWF-G-KSVL----L---SHLHQ--LNSKASCTCQSL

LLDRMLNITETILQD-LRGK-----------A------ENE---ETKT-------RLTDV

--M----TEVKI---LR-HKY--SEEQKVWREL-QDIHSVEVKNGTNQKGALNSFLILYD

LA--Y-------------------------------------------------------

------------------------------------------------------------

---------------------------------------------

>ACV41807.1_IFN_G_1_Carassius_auratus

MY---------CRLNM------V------YL--ICALLL---IVS--------------L

---QG--TVGARLPQSQKDKE---Q----ML---KN----------V-R---EKIES--L

QK-HY-------H-----T-TGTEWF-G-KSVL----S---SHLHQ--LNSKASCTCQSL

LLDSMLNITETIFQD-MRGK-----------A------ENE---ETKT-------SLRDV

--M----TEVKM---LR-HKY--SEEQKVWREL-QDIHSVEVNNGKIQKGALNSFLILYD

LA--Y-------------------------------------------------------

------------------------------------------------------------

---------------------------------------------

>BAD72865.1_IFN_G_1_Danio_rerio

MD---------SCLKM------V-------L--LCGLLW---IAS--------------L

---QT--TSAYRFRRSRSENP---I----LN---TN------------------IEK--L

KT-HY-------N-----T-LAKDWV-G-KSVF----V---SHLDQ--LNSKPTCTCQAV

LLEGMLSIYEDIFQD-MMNK-----------S------DNK---EVRD-------DLKKV

--I----HEVKN---LK-HKY--NEEHKLWREL-QDIHSVKAKNGTIQERALNDFLKVYY

RA--ST-----------EK--RHLHM----------------------------------

------------------------------------------------------------

--------S------------------------------------

>ACN56578.1_IFN_G_Ctenopharyngodon_idella

MD---------SWLNM------M-------L--LCGLLL---IAS--------------L

---QT--TNAFRFRRSKSEMT---H----LE---TN------------------IHS--L

QE-HY-------K-----T-RGTEWV-S-KSVF----V---PHLNQ--LNSKASCTCQAL

LLERMLNIYEELFQD-M--K-----------S------EHK---EGRK-------DLDHL

--M----DEVKK---LR-GNY--KEEHKVWKEL-QEMNSVKVKNGTIRGGALNDFLMVFD

RA--ST-----------EK---HKKV----------------------------------

------------------------------------------------------------

--------Q------------------------------------

>AAZ40504.1_IFN_G_1_Ictalurus_punctatus

MG---------SWSNV------L-------L--MCGLVM---VAL--------------L

---NG--TTG--------------H----EI---HN----------L-T---EAVHT--L

QI-HH-------G-----L-TDTKWV-G-KAVF----T---PYLGK--V--EDTCTCEKL

VLLRMLNGYMDIFSD-MLKK-----------A------KTV---ETET-------SLKEL

--Q----ESVKE---LK-NKY--NNEQAVWKQL-HEINTVKKDDSTIQGGAVNDFISVYD

KA--FV-----------VA--QHSKK----------------------------------

------------------------------------------------------------

--------TP---L----LL---------K---H-----FLQR--

>CAE82301.2_IFN_G_Takifugu_rubripes

---------------M------V-------T--MATAVVGW-CLC--------------L

---AVCHVRG--------------S----YIP--VE----------M-N---KTIQN--L

LG-HY-------T-----I-TNKELFDG-KPIF----S---K--EP--L--SGNLQAEMI

YMSAILQTYDKILNQ-MLKELPTPGPTTAQSS------GDKGTAELRS-------QLNYI

--L----KKITN---LRIQHY--NKPEQLLKML-QPLREVQFNNAVIQSKALWELIKVYR

EA--SSLP---NKLEK-RR--RRRRR----------------------------------

------------------------------------------------------------

--------QT---Q----MS---------IRG-H-----------

>AAW21707.1_IFN_G_Salmo_salar

---------------M------D-------V--LSRAVM---CFC--------------L

---MGWMTLG-----------WSNAAQ--YTS--IN----------M-K---SNIDK--L

KV-HY-------K-----I-SKDQLFNG-KPVF----P---K--DT--F--EDSERR--V

WMSVVLDVYRSIFNQ-MLNQ-----------T------GDQ---EVRE-------RLDQV

--K----GKVQE---TQ-KHYFLKRIPELRTHL-QNLWAIETSNTTVQGKALSEFITIYE

KA--SKLALKI-HLKK-DN--RRKRR----------------------------------

------------------------------------------------------------

--------QA---Q----RL---------KSS-I-----M-----

>CAR95730.1_IFN_G2_Oncorhynchus_mykiss

---------------M------D-------V--LSRAVM---CFC--------------L

---MGWMTLG-----------WSNAAQ--YTS--IN----------M-K---RNIDK--L

KV-HY-------K-----I-SKDQLFNG-NPVF----P---K--DT--F--EDSEQR--V

LMSVVLDVYLSIFSQ-MLNQ-----------T------GDQ---EVRE-------RLDQV

--K----GKVQE---TQ-KHYFLGRIPELRTHL-QNLWAIKTSDTTVQGKALSEFITIYE

KA--SKLAHEI-HLKK-DN--RRKRR----------------------------------

------------------------------------------------------------

--------QA---Q----RL---------KSTHI-----M-----

>CAE82300.1_IFN_G_Oncorhynchus_mykiss

---------------M------D-------V--LSRAVM---CFC--------------L

---MGWMTLG-----------WSNAAQ--FTS--IN----------M-K---RNIDK--L

KV-HY-------K-----I-SKDQLFNG-NPVF----P---K--DT--F--EDSEQR--V

LMSVVLDVYLSIFGQ-MLNQ-----------T------GDQ---EMIE-------SLKYV

--K----GKIQD---LQ-KHYFLGRIPELRTHL-QNLWAIETSDTTVQGKALSEFITIYE

KA--SKLALKF-HLKK-DN--RRKRR----------------------------------

------------------------------------------------------------

--------QA---Q----RL---------KSH-I-----M-----

>AAZ40506.1_IFN_G_2b_Ictalurus_punctatus

---------------M------T-------L--FWR--I---CFV--------------F

---FGMMA-------------YSEA----FLP--KN----------I-K---ESIDH--L

NN-HY-------N-----P-NPGKLYDG-HSLFLDKLT---K--QK--F--EESEQK--L

LMTIILDAYNKIFTK-MENE-----------T------QDE---TLKN-------HLHEV

--K----DQMNK---LK-EHYFSGKHADIKKYV-TELLDLKENDPRIQSKAIFELKAVYN

KA--TNLG----RMSA-EN--PRRRR----------------------------------

------------------------------------------------------------

--------QA---K----SS---------KKQ-H-----S-----

>AAZ40505.1_IFN_G_2a_Ictalurus_punctatus

---------------M------T-------L--FWR--I---CFV--------------F

---FGMMA-------------YSEA----FLP--KN----------I-K---ESIDH--L

NN-HY----VRKN-----P-NPGKLYDG-HSLFLDKLT---K--QK--F--EESEQK--L

LMTIILDAYNKIFTK-MENE-----------T------QDE---TLKN-------HLHEV

--K----DQMNK---LK-EHYFSGKHADIKKYV-TELLDLKENDPRIQSKAIFELKAVYN

KA--TNLG----RMSA-EN--PRRRR----------------------------------

------------------------------------------------------------

--------QA---K----SS---------KKQ-H-----S-----

>CAJ51088.1_IFN_G_2a_Cyprinus_carpio

MT---------AQNTM------A-------F--FWG--V---CLL--------------T

---SGWMT-------------YGEA----SVP--EN----------L-D---KSIDE--L

KA-YY-------I-----K-DDHELHNA-HPVFLRALKDL-K--VN--L--EEPEQN--L

LMSIIMDTYSRIFTR-MEND-----------S------LDE---ATKE-------RLAHV

--Q----EHLKK---LK-ENYFPGKSAELKTYA-ETLWAIKEDDPVIQRKALFELKRVYR

EA--TQL-----RNLK-NK--ERRRR----------------------------------

------------------------------------------------------------

--------QA---K----IT---------KKQ-K-----S-----

>BAD06253.1_IFN_G_Danio_rerio

MI---------AQHMM------G-------F--AWG--V---CLL--------------F

---SGWMT-------------YSEA----SVP--EN----------L-D---KSIEE--L

KA-YY-------I-----K-EDSQLHNA-HPIFLRILKDL-K--VN--L--EESEQN--L

LMSIVMDTYSRIFTR-MQND-----------S------VDE---ATKE-------RLAHV

--Q----EHLKK---LQ-ESYFPGKSAELRTYA-ETLWAIKENDPIVQRKALFELKRVYR

EA--TLL-----KNLK-NK--ERKRR----------------------------------

------------------------------------------------------------

--------QA---K----AS---------RS--K-----SLNRG-

>ACG68885.1_IFN_G_Carassius_auratus

MI---------AQNMT------I-------F--FWG--V---CLL--------------T

---SGWAT-------------YSEA----SVP--EN----------L-D---KSIDE--L

KA-YY-------I-----K-DDHEIHNA-HPVFLRVLKDL-K--VN--L--EEPEQN--L

LMSIIMDTYSRIFTR-MEND-----------S------LDE---ATKE-------RIAHV

--Q----EHLKK---LR-ENYFPGKSAELKTYA-ETLWAIKEDDPVIQRKALFELKRVYR

EA--TLL-----KNLK-NK--ERRRR----------------------------------

------------------------------------------------------------

--------QA---K----NT---------KNL-K-----S-----

>AAI04254.1_IL_10_Homo_sapiens

-M---------HSSAL------L-------C--CLVLLT---GVR--------------A

---SP--GQG--------------T----QSE--N--SC-THFPGNL-P---NMLRD--L

RD-AF-------SRVKTFFQMKDQLDNL--LLK----E---SLLED--F--KG-Y-LGCQ

ALSEMIQFYLE--EV-M--------------P------QAE---NQDP-------DIKA-

-HVNSLGENLKT---LR-LRL--RRCHRFLP------C---ENKS----KAVEQVKNAFN

KL-------------Q-EK--GIYKA----------------------------------

------------------------------------------------------------

--------MS---E----FDIFINYIEAYMTM-K-----IRN---

>AAG16755.1_IL_19_Homo_sapiens

-M---------KLQCV------S-------L--W--LLG---TIL--------------I

LCSVD--NHG--------------L----R-------RC-L-----I-S---TDMHH--I

EE-SF-------QEIKRAIQAKDTFPNV-TILS----T--LETLQI--I--KP-L-DVCC

VTKNLLAFYVD--RV-F--------------K------DHQ-----EP-------NPKIL

RKISSIANSFLY---MQ-KTL--RQCQEQRQ------CHCRQEAT----NATRVIHDNYD

QL-------------EVHA--AAIKS----------------------------------

------------------------------------------------------------

--------LG---E----LDVFLAWINKNHEV-M-----SSA---

>AAH69311.1_IL_20_Homo_sapiens

-M---------KASSL------A-------F--S--LLS---AAF--------------Y

LLWTP--STG--------------L----KTL--NLGSC-V-----I-A---TNLQE--I

RN-GF-------SEIRGSVQAKDGNIDI-RILR----R--TESLQD--T--KP-A-NRCC

LLRHLLRLYLD--RV-F--------------K------NYQ-----TP-------DHYTL

RKISSLANSFLT---IK-KDL--RLCHAHMT------CHCGEEAM----KKYSQILSHFE

KL-------------EPQA--AVVKA----------------------------------

------------------------------------------------------------

--------LG---E----LDILLQWMEE------------TE---

>AAG41401.1_IL_24_Homo_sapiens

-MNFQQ-----RLQSL-WTLARP-------F--CPPLLA---TASQMQMVVLPCLGFT-L

LLWSQ--VSG--------------A--QGQEF--HFGPCQV-----K-G---VVPQK--L

WE-AF-------WAVKDTMQAQDNITSA-RLLQ----Q---EVLQN--V--SD-A-ESCY

LVHTLLEFYLK--TV-F--------------K------NYH---NRTV-------EVRTL

KSFSTLANNFVL---IV-SQL--QPSQENEM------FSIRDSAH----RRFLLFRRAFK

QL-------------DVEA--ALTKA----------------------------------

------------------------------------------------------------

--------LG---E----VDILLTWMQKFYKL-------------

>EAW97181.1_IL_26_Homo_sapiens

-M---------LVNFI------L-------R--CGLLLV---TLS--------------L

---AI--AKH--------------K----QSS--FTKSC-Y-----P-R---GTLSQ--A

VD-ALYIKA---AWLKATI-PEDRIKNI-RLLK----K---KTKKQ--F-----M-KNCQ

FQEQLLSFFME--DV-F--------------G------QLQ---LQ---------GCKKI

RFV----EDFHS---LR-QKL--SHC---IS------CASSAREM----KSITRMKRIFY

RI-------------G-NK--GIYKA----------------------------------

------------------------------------------------------------

--------IS---E----LDILLSWIKKL--L-E-----SSQ---

>AAH69308.1_IL_22_Homo_sapiens

-M----AALQKSVSSF------LMGTLAT-S--CLLLLA--------------------L

---LV--QGG--------------A----AAP--ISSHC-R-----LDK---SNFQQPYI

TNRTF-------MLAKEAS-LADNNTDV-RLIG----E---KLFHG--V--SM-S-ERCY

LMKQVLNFTLE--EVLF--------------P------QSD---RFQP-------YMQEV

--V----PFLAR---LS-NRL--S------T------CHIEGDDLHIQ-RNVQKLKDTVK

KL-------------G-ES--GEIKA----------------------------------

------------------------------------------------------------

--------IG---E----LDLL------FMSL-R-----NAC--I

1. **IFN3:**

>scca_L

------MAF--APSG-----------VVLLLVATFLA--PSLGSGRV-----------RR

SCSLSRYARLPPSV---FKLFGDFHRSLGSSQEPGHRQ---VLVSSEQLR--S------L

EVPERLLL-VEAEFRLFIRMMQELEEP--ILQNLTKKVLEVLYQMCWNLGRCLPETQKDQ

---TKYPK-------QLKKFLRNLKHAGR---LGRTKQYIQTNLMLLLN-EHLSCVAS--

-GEDC-

>oran_L4

-----M-----EARL-----------WSLVATGLWLVLLEVSCSS---FMD-------GK

KCYLAHYGSLDPQVLRDVKDLQNRY-----------------------------------

-ACGRLLL-LERELAHAQAVLRNLSGL--DLGRNATRPLQLLAAICEDLASC---VPPSA

---RQRE-MGH----RARRQLRSKAKAKKEVTPRCLEAAVVLNLFRLLTR-DLRQAT-YL

--KPCA

>oror_L4

-----M-----GPSG-----------AAAVAVGLW-VLVTVGVAADPEVVE-------PP

RRSLSHYRSLDPRALLAIKALRDRYEEETLSWRPRNCS---FRR-RRDPP--R------P

SSRALLRQ-VARGLADAQDVLSSLPSP--ELFPGVGPTLELLAAAGRDVAACLELVRPGS

---RSKS-LRR----PRRRP-----QTRTADSPRCHEATVIFNLLRLLAW-DLRLVA-HS

--GPCL

>Bota_L4a

-----M-----GRSG-----------TAAAAIGLW-VL----------------------

-------------------------EEETLSWGPRKGS---IRP-KRNPP--R------L

SSCAMLRR-VAHDLADAQAVLSSLPSP--ELFPGVGQTLELLAAAGRDVAACLELARPGS

---WRRS-PRR----PGRRP-----KTRRAESPRCHEATVIFHLLHLLAW-DLRLVA-HA

--GPCL

>Bota_L4b

-----M-----GQSG-----------TAAAVVGLW-VLVTVGVAANPNVTE-------PQ

RCLLSHYRSLDPRALLAVKALRDHYEEETLSWGPQNCS---IRP-KRNPP--R------P

SSCAMLRR-MARDLADAQAVLSSLPSP--ELFPGVGQTLELLAAAGRDVAACLELARPGS

---WRRS-PRR----PGRRP-----KTRRAESPRCHEATVIFHLLRLLAW-DLRLVA-HA

--GPCL

>Ovai_L4

-----M-----EGAD-----------TADLA----------------------------R

YCLLSHYRSLDPRALLAVKARRDHYEEETLSWGPQNCS---IRL-KRNPP--R------P

SSCAMLRR-VARDLADAQAVLSSLPSP--ELFPGVGQTLELLAAAGRDVAACLELARPGS

---WSRS-PRR----PGRRP-----KTRRADSPRCHEAAVIFHLLRLLAW-DLRLVA-RA

--GPCL

>New|XP_013845957.1_IFN_L-4_Sus_scrofa

-----M-----GPRG-----------TAAVAMGLW-VFVTAVFALDPDVVV-------PG

RCVLSHYRSLDPQALVAVKALRDHYEEETLSWRPRNCS---FRL-RRDPP--P------P

SSCARLRL-VARGLADAQAVLSSLPSP--ELFPGVGPTLELLAAARRDVAACLELVQPGS

---GRKS-LRP----PRRR--------HRADSPRCHEATVIFNLLRLLAW-DLRLVA-HS

--GPCL

>New|XP_023616326.1_IFN_L-4_Myotis_lucifugus

-----M-----RPNG-----------AAAVSVGLW-VLVTVNVVADPGVTE-------PR

RCSLSHYRWLDPRALKAVKALKDHYEEEMLSWRPRNCS---FRP-RRVPP--L------P

WSCARLRL-VARGLSDAQAVLSSLPSP--ELFPGVAPTLELLASARRDVVACLELVRPGS

---SRKS-PRA----PRRRP-----QRRRAVSPERVAATIIFNLLRLLTW-DLKLAA-LS

--GPCL

>New|XP_010598693.1_IFN_L-4-like_Loxodonta_africana

-----M-----GLNV-----------GAAVAVGLW-VLVTVGVAADPVVQV-------PR

PCLLSHYRSLDLKVLAAVKGLRDRYEEETLTWRPRTCS---FRL-RRVPP--K------P

ASCARLRV-VARGLADAQAVLSSLRSL--ELLPGTSPALELLAAAGRDVGACLEPVRPGW

---SRKS-LRP----PRRRH-----KARRAESPQCHEARVIFNLLRLLTW-DLRLVA-LS

--GPCV

>New|XP_010598629.2_IFN_L-4-like_Loxodonta_africana

MGRAKM-----GSNV-----------GAAVAVGLW-VLVTVGVAAEPVVQE-------PR

PCLLSHYRSLDPGALAAVKALRDRYEEEALRWGPRNCS---FRLRRREPP--R------P

ASCARLRV-VARGLADAQAVLSSLRSL--ELLPGTRRTLELLAAAGRDVGACLELVRPGW

---SRKS-LRP----PRRRH-----KARRAESPQCREPTVIFNLLRLLTW-DLRLVA-LS

--GPCV

>New|NP_001263183.2_IFN_L-4_Homo_sapiens

-----M-----RPSV-----------WAAVAAGLW-VLCTVIAAA-------------PR

RCLLSHYRSLEPRTLAAAKALRDRYEEEALSWGQRNCS---FRP-RRDPP--R------P

SSCARLRH-VARGIADAQAVLSGLHRS--ELLPGAGPILELLAAAGRDVAACLELARPGS

---SRKV-PGA----QKRRH-----KPRRADSPRCRKASVVFNLLRLLTW-ELRLAA-HS

--GPCL

>New|XP_009230852.2_IFN_L-4_Pongo_abelii

-----M-----RPSV-----------WAAVAAGLW-VLCTVVAAA-------------PQ

RCLLSHYRSLEPRTLAAVKALRDRYEEEALSWGPRNCS---FRP-RRDPP--R------P

SSCARLRH-VARGIADAQAVLSGLHRP--ELLPGTVPILELLAAAGRDVAACLELARPGS

---SRKV-PGA----QRRRH-----KPRRADSPRCREASVVFNLLRLLTW-ELRLAA-HS

--GPCL

>New|XP_023616327.1_IFN_L-4-like_Myotis_lucifugus

-----M-----RPNG-----------AAAVSVGLW-VLVTVNVVAEPGVTE-------PR

RCSLSHYRWLDPRALEAVKALRDHYEEEMLSWRPRNCS---FPP-TEGSP--S------P

QSCARLRL-VARGLSDAQAVLSSLPSP--ELFPGVAPTLELLASARRDVVACLELVRPGT

---GDAD-PTS----HGRGS-----TSLGTSSPE--------------------------

---PSL

>ereu_L4a

-----M-----GALGTEMGVLGARSRAVVAAAGLW-VLVAVDAVAQPGQQP-------PR

HCLLSHYSSLDPTVLLEVKALRDHYEEETLSWRPQNCS---FRP-RRMPP--P------P

SSCARLLL-VARDIVDTQAVISSLRSL--GPVPGAGPTLDLLVAAGRDLAACLELVRPGS

---SRRL-LGP----RRRSQ-----KTKRQDSPRCHEAKVIFSLLRLLTW-DLRLVA-QL

--GPCL

>ereu_L4b

-----M-----EAAGTEMGVLGARSRAVVAAAGLW-VLVAVDAVAQPGQQP-------PR

HCLLSHYSSLDPTVLLEVKALRDHYEEETLSWRPQNCS---FRP-RRMPP--P------P

SSCARLLL-VARDIVDTQAVISSLRSL--GPVPGAGPTLDLLVAAGRDLAACLELVRPGS

---SRRL-LGP----RRRSQ-----KTKRQDSPRCHEAKVIFSLLRLLTW-DLRLVA-QL

--GPCL

>ptva_L4

-----M-----GPRG-----------AVAVTVGLW-VLAT--VAADHGEAK-------PG

RCLLSHYRSLDPRALEAVKALRDRYVSDAQT----------------------------P

ASWAR-------------------------------------------------------

------------------------------------RATVIFNLLRLLAW-DLRLVA-HS

--GPCL

>New|XP_023616336.1_IFN_L-4-like_Myotis_lucifugus

-------------------------------MGLW-VLVTVNMVADPGVTE-------PR

RCSLSHYHWLDPRALEAVKALRDHYEEEMLSWRPRNCS---FRP-RRVPP--L------P

W---------VRPAAQLVRPSSSRKSR--GPQEASPKAETVSGAAARSPGSLNEEIAASA

---PLRF-L-----------------PRFQDSPRCHEATIIFNLLRLLTW-DLKLAA-LS

--GPCL

>New|XP_008171423.1_IFN_L-3-like_Chrysemys_picta_bellii

-----M--------S-----------RTLLVSALLLALVDAFAEGAPNEM----------

--------------------LRD---------PAANCS---ARIFRRKLD--K------L

SECQNLLV-LEKKVDVTIRVIQNLSDP--ELVRNASKPLEILASIQEDLRSCIQLKGLGG

---DPAA-QQP----VLARWLRNAYAGKSEGSARCLEEAMIFNLFRLLN--DLQNVA-HK

FNESCH

>New|XP_024066418.1_IFN_L-3-like_Terrapene_mexicana_triunguis

-----M--------V-----------RTLLVSAFLLVLVEAFAEGAPNEM----------

--------------------LQD---------PAVDCS---AGIFRRKLD--E------L

SECQNLLV-LEKKVDVTIRVIQNLSEP--ELVRNASKPLEILASIQEDLRSCIQRKGLDG

---DVAA-QRP----ILAKWLRKAHAGKSEGSARCLEEAVIFNLFRLLN--DLQNVA-HK

FNESCH

>New|XP_001517931.2_IFN_L-3_Ornithorhynchus_anatinus

-----MRQT--G--H-----------LLILAVTLAAVSTGAQAADPP-----------KK

QCYLGKFKSLSPQELEAFKKAKDAFEESML-LTDRTCR---VQIFHRSWSVKQ------L

QVKERLLF-LEAELKLTVEVLGKMSKS--NLEGYLGRPLWTLRYISQELQRCIPQAQE-S

----RHSS-------RLTHWLHKLQEAREK----------------------LALV----

-----L

>oror_L1

-------------------------------MGLGLARACPVPTT--------T----RK

GFDIGVFKSLLPNELVAFKKAKDG---NSL-AKDWNCS---SRLFPRTRDLKQ------L

QGXEHPTA-LEAELALTLKVLGMVANS--SLDHILDQTLHTLHHIHSKLQACVQ-AQP-T

AGP-GPRS-------HFEYQLHWLQEAPKKEFQDGLKASVTFILFHLLT-WDLNFI----

------

>New|XP_004271427.1_IFN_L-3-like_Orcinus_orca

MKL-DVAPG--C--R-----------LLLVLLTVAPSRTGAVPVPSP----LGALPG-AR

GCHMAQFKSLSPQELQAFKRAKDAFEESLL-QKDWNCS---SRLFPRTRDLRQ------L

QVWERPVA-LEAELALTRNVLEATANS--SLDHILDQPLHTLHHIHSKLQACVP-ARP-T

AGP-RPRD-------RLHHWLHRLQEAPKKESRDCLEASVVFNLFRLLT-RDLKCVAS--

-GDQCV

>New|XP_007180050.1_IFN_L-3-like_X1_Balaenoptera_acutorostrata_scammoni

-----MAPG--C--T-----------LVLVLMTAAPSRTGAAPVPSP----LGALPG-AR

GCHMAQFKSLSPQELQAFKRAKDAFEESLL-QKDWDCS---SRLFPRTRDLRQ------L

QVWERPVA-LEAELALTLNVLEAMANS--SLDHILDQPLHTLHHIHSKLQACVP-AQP-T

AGP-RPRG-------RLHHWLHRLQDAPKKESRDCLEASVMFNLFRLLT-RDLKCVAS--

-GDQCV

>New|XP_006195414.1_IFN_L-3_X2_Camelus_ferus

-----MTSG--C--L-----------LGLLLMTAALTRTGAVPVPSP----LRALPG-TR

GCHLAQFKSLSPQELQAFKRAKDAFEESLL-QKDWNCS---SRIFPRTRDLRQ------L

QVWERPVA-LKAEVALTLAVLGTMANS--SLGSTLDQPLHTLGHIHSQLQACVP-AQP-T

AGP-RPRD-------RLHHWLHRLQEAPKK-SQDCLEASVMFNLFRLLT-RDLKCVAS--

-GHLCV

>Bota_l3

-----MAPG--C--T-----------LVLVLMTVALSRTGAVPVPSA----PRALPP-AR

GCHVAQFKSLSPQELQAFKTARDAFEDSFL-PKDWDCS---THLFPRTRDLKH------L

QVWERPVA-LEAELALTLTVLEAMANS--SLGHSLEQPLLTLQNIHSKLQACVP-AQP-T

ASS-RPRG-------RLHHWLHRLQEA-RKESQDCLEASVMFNLLRLLT-RDLKCVAS--

-GDQCV

>New|XP_014955839.1_IFN_L-3_Ovis_aries

-----MAPG--C--T-----------LVLVLMTAALSRTGAVPVPSA----PRALPP-AR

GCHMAQFKSLSPQELQAFKTVRDAFEDSRL-QKDWDCG---GLLFPRTXDLKH------L

QVWERPVA-LEAELALTLTVLEAMANS--SLGRSLEQPLLTLQHVHSKLQACVP-AQP-T

AGP-RPRG-------RLHHWLHRLQEAQKKESQDCLEASVMFNLFRLLT-RDLKCVAS--

-GDQCV

>susc_L3

--M-ALGGS--L--V-----------LVLVLMTVAPPRTGAVPVPEA----LRALPG-AR

GCHLAQFKSLSPQALQAFKRAKDAFEESLL-E-DWNCS---SRIFPRSRDLKQ------L

QVWERPVA-LEAEVALTLSVLGSLANS--SLHSSLDQPLHTLRHIHAQLQACVP-AQP-M

AGP-RPRG-------RLHHWLHRLQEAQKKEPQSCLEASVMFNLFRLLT-RDLKCVAS--

-GDLCV

>New|XP_007524040.1_IFN_L-3-like_Erinaceus_europaeus

-----MALG--P--R-----------LLLLLLSMVLTSTGAAPAPKS----HRGPPD-AR

GCHLDQFKSLSPQELQSFKRAKDALEDWLL-LKDWSCS---SRLFPRTQDLRQ------L

QVWERPVA-LEAELAVSLKVLGALAES--PMGRVLEQPLRTLHHAHSQLQACVP-AQP-T

VGP-RPRG-------RLQHWLHRLQEAPKKESQGCLEASVTFNLFRLLT-WDLSCVAS--

-GDLCV

>New|XP_016044015.1_IFN_L-3-like_Erinaceus_europaeus

-----MALG--P--R-----------LLLLLLSMVLTSTGAAPAPKS----HRGPPD-AR

GCHLDQFKSLSPQELQSFKRAKDALEDWLL-LKDWSCS---SRLFPRTQDLRQ------L

QVWERPVA-LEAELAVSLKVLGALAES--PMGRVLEQPLRTLRHAHSQLQACVP-AQP-T

VGP-RPRG-------RLQHWLHRLQEVPKK-SQDCLEASVTFNLFRLLT-WDLSCVAS--

-GDLCV

>New|XP_023395605.1_IFN_L-3-like_Loxodonta_africana

MTEALM------------------------IVATMLTMTGAVPTPTP----SGALLD-TR

GCHMARFQSLSPRELQAFKRAKDTFEESLL-LKDRSCS---SRLFPRTWDLKQ------L

QV-----S-WESQGHP---CPHLVLLI--LLGAVLDQPLHTLHHIHSKLRACVS-AQP-T

SGP-RPQG-------HLHRWLHRLQEATKKESQSCLEASVMFNLFRLLT-RDLKCVAS--

-GDQCA

>loaf_L3b

-----M------------------------LMSTMLTTTGAVPICIP----SGTPLN-AR

GCRIDQFKSLSPRELQAFQRAKDAFEESLL-LKDGSCS---SLLFPRTWDLRQ------L

QVWERPMA-LEAELALTVKVLRTMSDS--ALGAVLDQPLHTLHHIHSKLRACVS-AQP-T

SGP-RPQS-------RLHRWLHRLQKATKKESQSCLEASVMFNLFRLLT-RDLKCVAS--

-GDQCA

>loaf_L3d

-----M--G--C--L-----------LVLMLMATMLTMTETVPTPTP----SRAPLD-AR

GCHIAQFKSLSPQELQAFKRAKDAFEETLL-LKNGSCS---SRLFPRTWDLKQ------L

QVWERPVA-LEAELALTLKVLETVVDS--ALRAILDQPLRTLHHIHSKLRACVS-PQG-T

QGP-PPQ-----------------------ESRTCLEASVTFNLFRLLT-RDLKCVAS--

-RDQCA

>New|XP_023616322.1_IFN_L-3-like_Myotis_lucifugus

MTLLGWSV---E--A-----------LVLMLMTTVLTSTGAVPVTTP----LATLLG-AR

GCDMAQFKSLPPQEMKAFRRAKAALEESLL-LRNRSCS---SRPLPRTRDLRQ------L

QVWERPVA-LEAELALTLKVLGPVANS--TLGDILDQPLRTLRHIHSKLQACVP-AQS-T

AGP-RPRG-------RLRQWLHRLQDAETKESRGCLEASVASNLFRLLV-RDLRCVAD--

-GDLCV

>New|XP_006096810.1_IFN_L-3-like_X2_Myotis_lucifugus

MKL-GLAVC--C--A-----------LVLMLMTTVLTSTGAVPVITP----LATLLG-AR

GCDMAQFKSLSPQEMKAFRRAKAALEESLL-LRNRSCS---SHPLPRTRDLRQ------L

QVWERPVA-LEAELALTLKVLGTVANS--TLGDILDQPLRTLRHIHSKLRACVP-AQS-T

AGP-RPRG-------RLRQWLHRLQEAEKKESRGCLEASVTLNLFRLLV-RDLRCVAD--

-GDLCV

>New|XP_023616345.1_IFN_L-3-like_X1_Myotis_lucifugus

MTLTGLAVC--C--A-----------LVLMLMTTVLTSTGAVPVITP----LATLLG-AR

GCDMAQFKSLSPQEMKAFRRAKAALEESLL-LRNRSCS---SHPLPRTRDLRQ------L

QVWERPVA-LEAELALTLKVLGTVANS--TLGDILDQPLRTLRHIHSKLRACVP-AQS-T

AGP-RPRG-------RLRQWLHRLQEAEKKESRGCLEASVTLNLFRLLV-RDLRCVAD--

-GDLCV

>New|XP_006761534.1_IFN_L-3_Myotis_davidii

MKLLAVCCT--L--V-----------LVLMLMASVLTRTGAAPAPTP----PRTHLG-AR

GCHMGQFKSLSPQEMKAFKRVKDALEESLL-LKNWSCG---SRPLPRTRDLRQ------L

QVWERPVA-LEAELALTLKVLGTLKNS--TLGDILDQPLHTLHHIHSKLQACAP-AQA-T

AGP-RPRG-------LLRQWLHRLWEAGKKESQGCLEASVTLNLFRLLT-RDLRCVAA--

-GDLCA

>New|ELK31984.1_IL-28B_Myotis_davidii

MKLLAVCCT--L--V-----------LVLMLMASVLTRTGAAPAPTP----PRTHLG-AR

GCHMGQFKSLSPQEMKAFKRVKDALEESLL-LKNWSCG---SRPLPRTRDLRQ------L

QVWERPVA-LEAELALTLKVLGTLKNS--TLGDILDQPLHTLHHIHSKLQACAP-AQA-T

AGP-RPRG-------LLRQWLHRLWEAGKKESQGCLEASVTLNLFRLLT-RDLRCVAA--

-GDLCA

>New|ENSPVAP00000017072

MEL-GAASA--C--T-----------LLLMLMTTGLTSTGAVPVPTP----L----A-TR

GCHMSQFKSLSPQELKAFKRAKDALEESLL-LKNCSCS---SRPFPRTRDLKQ------L

QVWERPMA-LEAELALTLKVLETVANS--TLGDILDQPLSMLRHIHARLQACVP-AQP-T

AGP-RSQG-------HLRHWLHRIQEAEKKESQGCLEASVTFNLFRLLI-LDLKCVAS--

-GNLCV

>New|ENSPVAP00000002134

MEL-GAASA--C--T-----------LLLMLMTTGLTSTGAVPVPTP----L----A-TR

GCHMSQFKSLSPQELKAFRRAKDALEESLL-LKNWSCS---SRPFPRTRDLKQ------L

QVWERPMA-LEAELALTLKVLETVANS--TLGDILDQPLSMLRHIHARLQACVP-AQP-T

AGP-RSQG-------HLRHWLHRIQEAEKKESQGCLEASVTFNLFRLLT-LDLKCVAS--

-GDLCV

>New|XP_023616323.1_IFN_L-3_Myotis_lucifugus

MAY-------------------------LLLLLWGLTLPSSSPTAG-------VNLQ-LS

SCGLTSAFALQ--------------EESLL-LRNRSCS---SRPLPRTRDLRQ------L

QVWERPVA-LEAELALTLKVLGTVANS--TLGDILDQPLRTLRHIHSKLQACVP-AQS-T

AGP-RPRG-------RLRQWLHRLQEAEKKESRGCLEASVTLNLFRLLV-RDLRCVAD--

-GDLCV

>New|ELK36696.1_IL-28B_Myotis_davidii

LKLPYLAVC--C--A-----------LVLMLMTTVLTSTGAVPVTTP----LATLLG-AR

GCDMAQFKSLPPQEMEAFRRAKDALEESLL-LRNRSCS---SRPLPRTRDLRQ------L

QVWERPVA-LEAELALTLKVLGTVANS--TLGDILDQRLHTLRYIHSKLQACVP-SSV-H

SRP-QARG-------HLCQWLHCLQETRKKESHGYLKASVTFNLFHHLT-HDLKYVSG--

-GDLCV

>hosa_il28a

-----MTGD--C--T-----------PVLVLMAAVLTVTGAVPVARL----HGALPD-AR

GCHIAQFKSLSPQELQAFKRAKDALEESLL-LKDCRCH---SRLFPRTWDLRQ------L

QVRERPMA-LEAELALTLKVLEATADTDPALVDVLDQPLHTLHHILSQFRACIQ-PQP-T

AGP-RTRG-------RLHHWLYRLQEAPKKESPGCLEASVTFNLFRLLT-RDLNCVAS--

-GDLCV

>hosa_il28b

MKL-DMTGD--C--M-----------PVLVLMAAVLTVTGAVPVARL----RGALPD-AR

GCHIAQFKSLSPQELQAFKRAKDALEESLL-LKDCKCR---SRLFPRTWDLRQ------L

QVRERPVA-LEAELALTLKVLEATADTDPALGDVLDQPLHTLHHILSQLRACIQ-PQP-T

AGP-RTRG-------RLHHWLHRLQEAPKKESPGCLEASVTFNLFRLLT-RDLNCVAS--

-GDLCV

>hosa_il29

-----MAAA--W--T-----------VVLVTLVLGLAVAGPVPTSKP----TTT----GK

GCHIGRFKSLSPQELASFKKARDALEESLK-LKNWSCS---SPVFPGNWDLRL------L

QVRERPVA-LEAELALTLKVLEAAAGP--ALEDVLDQPLHTLHHILSQLQACIQ-PQP-T

AGP-RPRG-------RLHHWLHRLQEAPKKESAGCLEASVTFNLFRLLT-RDLKYVAD--

-GNLCL

>New|XP_004481519.1_IFN_L-1_Dasypus_novemcinctus

-----MAAA--W--V-----------LVLVAVMLGLARAGPVPTSRP----TSA----RK

GCHIDRFKALSPRELEGFKKAKDAVEESLS-LKNWSCS---SRLFPRTWDLRQ------L

QSWERPVA-LEAELALTLQVLEATTDS--ALGAVLDQPLHTLRRVRSELQACVA-AGP-A

AGL-RPQG-------RLRHWLRRLHEAPRKESPSCLEASVTFNLFRLLL-WDLKCVAG--

-GDLCA

>dano_L3

-----------------------------MELKQGLAEAGRASRRRE----PGA----R-

GCHXALFKSLSPQELQTFKKAGDAIEESLL-LKDWSHN---SHLFPSTWGLRE------P

QRWERPVA-LEAELALTLQVLEATTDS--ALGAVLDQPLHTLRRLRSGRPGCVA-AGP-A

AGL-RPQG-------RLRHWLRRLHEAPKKESPSCLEASVTFNLFRLLL-WDLKCVAG--

-GDLCA

>Susc_L3

-----MATA--W--I-----------VVLATVMLDLARAGPVPTFKP----TTT----RK

GCHMGQFQSLSPQELKGFKKAKDALEESLS-LKNWSCS---SPLFPRTRDLRQ------L

QVWERLVA-LEAELDLTLKVLRAAADS--SLGVTLDQPLRTLHHIHVELQACIR-AQP-T

AGS-RLQG-------RLNHWLHRLQEATKKESQGCLEASVTFNLFHLLV-RDLRSVTS--

-GDLHI

>New|XP_006175047.1_IFN_L-1_Camelus_ferus

-----MAAA--W--V-----------LVLVTAMLALARAGPVPTSKP----TMT----EK

GCHMGWFQPLSPRELEGFKKAKDALEESLL-LKNWSCS---SRLFPRTRDLTQ------L

QVWERPVA-LEAELDLTLRVLGAAADS--SLGVVLDQPLRTLRHIHSKLQACVP-AQP-T

AGP-RPSG-------RLHHWLHRLQEATRKESQGCLEASVTFNLFRLLV-RDLRSVAS--

-GDLPT

>Calu_L3b

-----MATA--R--V-----------LVLVTVVLGLTRAGPVPTSKP----TTT----RR

GCHMDRFQSLSPRELEAFKKTKDALEESLS-WKNWSCS---SRLFPRSRDLRL------L

QAWERPVA-LEAELDLTLKVLENMTDS--SLGVTLDQPLRTLHHIHSELQACVP-AQP-T

ADP-RPHG-------RLHHWLHRLQKA-PKESQGCLEASITFNLFRLLT-RDLKCVAS--

-RDLCV

>Calu_L3a

-----MAIA--W--V-----------LVLVTAGLSLARAGPVPTSKP----TMA----WR

GCDIGRFKSLSPRELEAFKKAKDALEYSL---KNWSCN---SRLFPRNRDLRQ------L

QVWERPVA-LEAELALTLKVLETMADR--SLGDILDQPLHTLRHIHSQLQACVS-AQP-P

AGP-QPRG-------RLHPWLHRLHEASKKESQGCLEASVLFNLFRLLK-KDLECVAV--

-GDLCV

>New|XP_019674541.2_IFN_L-1_X1_Felis_catus

MELLVGSCT--W--V-----------LVLVAAGLSLASAGPVPTSKP----TTA----WT

DCDFGRFKSLSPRELEAFKEARDALENSL---KSWSCT---TRPFPRNRDLRQ------L

QVWERPVA-LEAELALTLKVLGAMADA--SQGDILDQPLHTLRHMHSELQACVS-AQP-T

AGP-QPQG-------RLHHWLHRLQEASRKESQGCLEASVLFNLFRLLK-KDLECVAS--

-GDLCV

>New|ENSPVAP00000014607

-----MAVA---------------WCLVLVLVAAGLGLAG----ASP-------------

-------------------------PTSM---PTSACN---ISLLPTEWKXXX------X

XVWERPMA-LEAELALTRKILETKADS--SLGDILDQPLHTLRHIHSELQACVL-PQP-T

ASP-RPRG-------RLHHWLHRLQKAPKKMSQDCLESTVTFNLFRLLT-RDLKCVAR--

-EDLCL

>ereu_L1

-----MATT--W--F-----------LMLLTLGLGLATTSLVPTSKP----ITT----KR

GCYIHRFRSLPPRELEAFKKAKDALEERLM-RNRWNCN---TSLFPKDWNLRL------L

QVWERPVA-LEAELTLVLKVLENLTES--PLGPVLDQPLSTLSQIHSQLQACLP-AQP-K

AGI-RPRG-------RLHHWLHRLQEAPKKETADCILASVTFHLFHLLL-QDLKCVAS--

-GDQCV

>New|XP_014443201.1_IFN_L-3_Tupaia_chinensis

------------------------------------------------------------

---MSRFKSLSPRELEAFKKAKDALEKSLS-LKKYSCK---FRLFPRDWHPRH------L

QMWERLMA-LEAELALTLRVLGSVADS--ALGDVLDQPLHVLRHIHSLLQACVS-VGH-S

A---QPQG-------HLPSWLLQLQEVEKKKSPGCLKAAATFNLFRLLN-QNLKCVAS--

-EDLCV

>New|XP_023395584.1_LOC100660429_Loxodonta_africana

---LVSPSP--L--L-----------IIYPHHLSHITC----------------------

-----------------------------L-PS--------------LYSLPV-------

PVWERPVA-LKAELAMTLKVLGTVANS--ALGAILDQPLHTLHHIHSKLWACVP-VNP-T

AGP-RPWG-------RLHRRLHRLQEATKKESRRCLQASVMFNIFRLLT-RDLKCVAS--

-GDQCA

>Modo_L3

------MEL--P--P-----------LLVCSLWTMGLSMSLTPAP-------SSLPP-EK

SCQIPQFKSLSPREMEAFTMAKDTYEKSML-QKKRKCS---SKFFHRGWDFRQ------L

QLADRPIV-LEAELKLTLEVLKGIQNP--ELQDVLIQPLQTLSHIYREIQSCVT-PLP-S

RGH-RLPG-------RPNNWLHRLTEA-GKESQSCLEASVMLTLPRLPD-TDLRFFLS--

-HNPSP

>New|XP_023357129.1_IFN_L-2-like_Sarcophilus_harrisii

----MVPQA--L--P-----------LLLSALMSGANSKSLAPTP-------SSLSP-ER

SCQISQFKSLSPRELETFKVAKDAYEKTML-QMERKCS---SRFFHRNWELRQ------L

PVSVRPVV-LQAELKLTLEVLKAVTKP--ELDSVLAQPLQTLSHIHQEIQSCVT-SQT-S

KEH-RLPG-------RLNNWLNKL-RG-SQLPRSIHHAQ------SLPT-PDPRCSLW--

-GPLLK

>fape_L3

-----MLCL--G--F-----------TPLLALVLGASLGAAFPQDAL-----------KK

SCSLSKYQFPVPHELKAVQKMKMHFEAIMH-QSNRRCN---TRLFHRKWNPAE------L

SVHDRMML-VEAELDLATTMLGLSAAP--KFAETRQQPLAFLTQAREDLRGCLAMEAP-S

---HQPSG-------KLRHWLQKLQVAKDTETTSCLEASTILHLFQVLN--DLRCTAL--

-REQCT

>apfo_L3

-----MLCL--S--F-----------TPLLALVLGASLGAAFPQDAL-----------KK

SCSLSKYQFLVPHELKAVQKMKEQFEDIML-LSDRKCN---TRLFHRKWNTAE------L

SVPDRVML-VEAELDLTTAMLGLPAAP--RFAETRQRPLAFLTQAREDLRGCMAMEAP-S

---HQPSG-------KLRHWLQKLQTAKKTETVGCLEATTILHLFQVLN--DLRCAAL--

-REQCT

>New|XP_015144667.1_IFN_L-3_Gallus_gallus

-----MVCY--G--V-----------TIILVGTLGSLLVGAFPQVTP-----------KK

SCSLSKYQFPAPLELKAVWRMKEQFEDIML-LTNRKCN---TRLFHRKWDIAE------L

SVPDRITL-VEAELDLTITVLTNPTTQ--RLAETCQQPLAFLTQVQEDLRDCLALEAP-S

---HQPSG-------KLRHWLQKLKTAKKKETAGCLEASAILHIFQVLN--DLRCAAQ--

-REDCT

>New|XP_005501745.1_IFN_L-3_Columba_livia

-----MLCL--S--F-----------TSLLVLVLGVSLGAAFPQGAL-----------KK

SCSLSKYKFIMRHEMKAARKMTEHFENIKL-QSDRKCN---TRLFHRKWKIAD------L

SVPDRVIL-VEAELNLTIAMLELPTVP--SFDEARQRPLAFLTQVREDLRGCMTMEAT-S

---HQPSG-------RLRHWLQNLQTAKNTETTGCLEASAILNIFQVLE--DLRCTAR--

-REQCT

>New|EOA99678.1_IL-28B_Anas_platyrhynchos

---------------------------------LGPLLAGAFPQAAL-----------KK

SCRLSQYGSPAYSELAEVLKFKKYY-ENIT-SKDSKCS---TRLFNRKWTPNE------L

SVPDRLLL-VEAELDLTIAVLAHPTVD--KLAEKSQQPLAFFTQAREDLRGCVAAEAP-S

---HQPSG-------KLRHWLQKLETAKKTETASCLEYSTIVHLFQVLH--DLGCVA---

------

>New|XP_008503116.1_IFN_L-2-like_Calypte_anna

-----MLLL--R--L-----------LPLLATTLGAPLL----QETP-----------GQ

RCGLSRYRFLPPQELRAVKRMKEQFEDIML-LSDHRCH---TKLFYRKWNPAE------L

SVPDRVML-AEAELDLAISMLELPAAP--TFAETRQRPLDFLAQAQEDLRSCMATEAP--

---HQPSR-------RLRNWLQKLQTAKETETTSCLEASVILYIFKVLN--DLQCAAL--

-GEQCS

>New|XP_025042667.1_IFN_L-3-like_Pelodiscus_sinensis

------------------------------------------------------------

--------MVSP------------------------------------------------

QVNDRVIL-VEEELNFTIHILENVEDA--TLSENLMRPLENLRHIREDLGNCIRDHLH-S

---HQRSE-------RLTRWLQKFHQAKKTKTPGCLEESVIFNLFRLFS-------SL--

-K--CM

>gavga_L

-----------------------------------------MPQQG-------------R

KC---------------------------------------TNIFSRAWEVAE------L

SVQDRVIL-VQAELNFTIEVLKNIEDP--NLSEQLPRPLEFLTHIGEDLKSCTFNHHH-S

---HKKSE-------KLSSWLQKFHEAKNKETRECLEESAILSIFRLLN-KDLACAAL--

-KDDCI

>crpo_L

-----------------------------------------MPQQG-------------R

KC---------------------------------------TNIFNRAWEVAE------L

SVQDRVIL-VQAELDFTIDMLKNIEDP--NLSEQLPRPLEFLTHIREDLKSCTFNHHH-S

---HKKSE-------KLSSWLQNFHEAKHKETRQCLEESAILSIFRLLN-KDLACAAL--

-KDDCI

>New|XP_025058026.1_IFN_L-3-like_Alligator_sinensis

-----MLQGN-TQSI----------RLGLFVMTVCAVFTETMPQQG-------------R

KC---------------------------------------TNIFNRPWEVAE------L

SVRDRVIL-VQAELDFTIDTLKNIEDP--SLSEQLPRPLEFLTHIREDLKSCTFDHHH-S

---HKKSE-------KLSSWLQKFHEAKSKETRECLEESVILSIFRLLN-KDLACAAL--

-KDDCI

>New|KYO33567.1_IFN_L-3_Alligator_mississippiensis

MSDEEWGEGN-TQSI----------RLGLFVMTVCAVFTETMPRQG-------------R

KC---------------------------------------TNIFNRAWEVAE------L

SVRDRVIL-VQAELDFTIDTLKNIEDP--SLSEQLPRPLEFLTHIREDLKSCTFDHHH-S

---HKKSE-------KLSSWLQKFHEAKSKETRKCLEESVILSIFRLLN-KDLACAAL--

-KDDCI

>New|XP_025042673.1_IFN_L-3-like_Pelodiscus_sinensis

MPSGSMMGV--G--Y-----------RVLLVLSLWVMTAEAFPKGAL-----------RT

KCHLAKYKSLPPRELEAFKKTKDKFEDILL-LSDRKCN---TRIFHRNWEVKE------L

SVHDRVIL-VEKELDFTINTLEDVEDP--SLSKLLPRPLEILSQIREDLRRCTR-QTP-S

---HSHSK-------RLNSWLQNLQASKEMESPACLEASVILNLFRLLN-EDLRCAAY--

-TELCV

>New|XP_023967605.1_IFN_L-3-like_Chrysemys_picta_bellii

MVDRKMVNV--G--D-----------KLLFALAIWTMTTEAFPKGTE-----------RT

KCHLAKYKSLPPQEPEAFKKAKDKFEDMML-SSNRKCN---TRIFHRNWEVKE------L

SVHDRVIL-VENELDFTINVLENVEDP--SLSKLLSRPLEILTQIRGDLRDCTR-QTH-S

---HRHSR-------RLNNWLQNFHKSKETETLGCLEASMVFNLFRLLN-QDLKCAAY--

-MESCT

>New|XP_025049184.1_IFN_L-3_Alligator_sinensis

-------------------------------LT-------PTP----------------R

T-----------------------------------------PHAPAHLPLCP------P

QGWAQLVL-VEAELDRTVRVLQNLSAP--QLAGKAARPLALLAGIRDELRRCIPLKAPAP

RGAFQAPT-------GLRKRLQRIWAKSSQASRECLEKAAVLNLFRLLY-KLVIPVAY--

----TS

>New|ANQ43304.1_type_III_IFN_6_Xenopus_tropicalis

------MEIPIRLAA-----------MMALLVTV---------TAHP----------HRR

HCHMSRYRSVSPSDIRAVRRLHNEHEKSP--------FSDGIKCYRKMLR--QKPSVCDL

QASDRLILTLER-VTLAVDVLTNMTES--PLAKLLSLPLTMLLSLEDDLKIC-----RKS

---PLYSD---PPSEQLMPWLHHLKHFREKVSSECVQDAVLLSLTQLLI-EDIMCWAN--

-NE---

>New|ANQ43335.1_type_III_IFN_1_Xenopus_laevis

------MEIPIRLAA-----------MMVLLVTV---------TAHP----------HRR

HCHTSRYRSLSPSDIRAVRLLHNEHEKSS--------TNDGIKCQKRMFR--QKPSVCEL

KASDRLILTLER-VTLAVDVLTNMSES--PLSEFISQPLEFFRSLEDDLKHC-----RKS

---PLNSD---PPSQQLMPWLNHLKHFREKVSSQCVQDAVLLSLIQLLI-EDVMCWAN--

-KE---

>New|ANQ43303.1_type_III_IFN_5_Xenopus_tropicalis

------MEIPIRLAA-----------MMVLLVTV---------TAHP----------HRR

HCHMSRYRSVSPSDIRAVRRLHNEHEKISF--DGIKCQ---KKMFR------QKPSVCDL

KASDRIILTLER-VTMAVDVLTNITES--PLSEFVSQPLEFFRSLEDDLKHC-----RKS

---PLYSD---PPSQQLMPWLNHLKHFRERVSSQCVQDAMLLSLTQLLI-EDVMCWAN--

-KE---

>New|ANQ43300.1_type_III_IFN_2_Xenopus_tropicalis

------MEIPIRLAA-----------MMVLLVTV---------TAHP----------HRR

HCHMSRYRSVSPSDIRAVRRLHNEHEKSP--------FSDGIKCQKKLFR--QKASVCDL

KASDRLILTLER-VTMAVDVLTNITDS--PLSEFVSKPLEFFHSLEDDLKHCRYRLQRKS

---PLYSD---PPSQQLMPWLNHLKHFRERVSSQCVQDAVLLSLTQLLI-EDVMCWAN--

-KE---

>New|ANQ43301.1_type_III_IFN_3_Xenopus_tropicalis

------MEIPIRLAA-----------MMVLLVTV---------TAHP----------HRK

HCHMSRYRSVSPSDIRAVRRLHNEHEKSP--------FSNGIKCQKKLFR--QKPSVCDL

KASDRLILTLER-VTMAVRVLTNMTES--PLSEFVSQPLEFFHSLEDDLKHC-----RKS

---PLYSD---PPSQQLMPWLNHLKHFRERVSSQCVQDAVLLSLTQLLI-EDVMCWAN--

-KE---

>New|ANQ43299.1_type_III_IFN_1_Xenopus_tropicalis

------MEIPIRLAA-----------MMVLLVTV---------TAHP----------HRR

HCHMSRYRSVSPSDIRAVRRLHNEHEKSP--------FSDGIKCQKKLFR--QKPSVCDL

KASDRLILTLER-VTMAVDVLTNMTES--PLSEFVTQPLEFFHSLEDDLKHC--------

------------------------------VSSQCVQDAVLLSLTQLLI-EDVMCWAN--

-KE---

>New|ANQ43340.1_type_III_IFN_6_Xenopus_laevis

------MAIPIRLAA-----------MMVLLVTV---------TAHP----------HRR

HCHMSRYRSLSPSDIRAVRLLHNEHEKSP--------FSDGIKCQKKMFR--HKPSVCDL

KASDKQILTLER-VTLAVDVLTNMTES--SLSEFVSQPLEFFRSLEDDLKHC--------

------------------------------VSVQCVQDAVLLSLIQLLI-EDVMCWAN--

-KE---

>New|ANQ43339.1_type_III_IFN_5_Xenopus_laevis

------MAIPIRLAA-----------MMVLLVTV---------TAHP----------HRR

HCHMSRYRSLSPSDIRAVRLLHNEHEKSP--------FSDGIKCQKKMFR--HKPSVCDL

KASDRHILTLER-VTLAVDVLTNMTES--PLSEFVSQPLEFFRSLEDDLKHC-----RKS

---PFYSD---SPSQQLMPWLNHLKHFRERVSAQCVGDAVLLSLIQLLI-EDVMCWAN--

-KE---

>New|ANQ43337.1_type_III_IFN_3_Xenopus_laevis

------MAIPIRLAA-----------MMVLLVTV---------TAHP----------HRR

HCHMSRYRSLSPSDIRAVRLLHNEHEKSP--------FSDGIKCQKKMFR--HKPSVCDL

KASDKQILTLER-VTLAVDVLTNMTES--PLSEFVSQPLEFFRSLEDDLKHC-----RKS

---PYYSD---SPSQKLMPWLNHLKHFRERVSAQCVQDAVLLSLIQLLI-EDVMCWAN--

-KE---

>New|ANQ43336.1_type_III_IFN_2_Xenopus_laevis

------MAIPIRLAA-----------MMVLLVTV---------TAHP----------HRR

HCHMSRYRSLSPSDIRAVRLLHNEHEKSP--------FSDGIKCQKKMFR--HKPSVCDL

KASDKQILTLER-VTLAVDVLTNMTES--PLSEFVSQPLEFFLSLEDDLKHCVYNFQRKS

---PLYSD---SPSQKLMPWLNHLKHFRERVSSQCVKDAVLLSLIQLLI-EDVMCWAN--

-KE---

>New|ANQ43343.1_type_III_IFN_9_Xenopus_laevis

------MAIPIRLAV-----------MMVLLVTV---------TAHP----------HRR

HCHMSRYRSLSPSDIRAVRHLHNEHEKSP--------FSDGIKCYRKMIR--QKPSVCDL

QESDRLILTLER-VSLAVDVLQNVTES--PLTTLVSQPLTMFLSLEDDLKFC-----RKS

---PKYSD---PPSPKLMPWLNHLKNFRERVPTECVQDAVLLSLIQLRI-EDVMCWAN--

-SE---

>New|ANQ43341.1_type_III_IFN_7_Xenopus_laevis

------------------------------------------------------------

-----------------------------------------------MLR--QKPSVCDL

QESDRLILTLER-VSLAVDVLQNVTES--PLTTLVSQPLTMFLSLEDDLKFC-----RKS

---PKYSD---PPSPKLMPWLNHLKNFRERVPTECVQDAVFLSLIQLRI-EDVMCWAN--

-SE---

>New|ANQ43338.1_type_III_IFN_4_Xenopus_laevis

------MAIPIRLAA-----------MMVLLVTV---------TAHP----------HRR

HCHMSRYRFLSPSDIRAVRLLHNEHEKSP--------FSDGIKCYRKMLR--QKPSVCDL

QESDRLILTLER-VSLAVDVLQNVTES--PLNTLVSQPLTMFLSLEDDMKFC-----RKS

---PKYSD---PPSPKLMPWLNHLKNFRERVPIECVQDAVLLSLIQLRI-EDVMCWAN--

-SE---

>New|ANQ43342.1_type_III_IFN_8_Xenopus_laevis

------MAIPIRLAA-----------MMVLLVTV---------TAHP----------HRR

HCHMSRYRSLSPSDIRAIRLLHNEHEKSP--------FSDGIKCQKKMFR--HKPSVCDL

KASDKQILTLER-VTLAVDVLTNMTES--PLSEFVSQPLEFFRSLEDDLKHC-----RKS

---PLYSD---SPSQKLMPWLNHLKHFRERVSAQCVQDAVIRPNLGLFQ-QDLAKSKN--

-LAESN

>napa_LR1a

------MDI--RLMV-----------LSIILVAV---------SGRL----------HKR

LCPMSRYLSVASSDITTLKQLQHNHEKNM--------SSNAMRCYRRMLR--HKPSVCDL

TQRDRLILTLER-VSLTVDVLTNMSMS--AQPDTIKQSLMVFLKLRDDLLVC-----RGS

---PEYSE---PTSPELKLWLHHLQRFKETASPDCVQEAVILSLIPLRV-EDVTCWAL--

-NQ---

>napa_LR1b

------MDI--RLMV-----------LSIILVAV---------SGRL----------HKR

LCPMSRYLSVASSDITTLKQLQHDHEKNM--------SINAMRCYRRMLR--HKPSVCDL

TQRDRLILTLER-VSLTVEVLTNMSMS--AQPDTVKQSLMVFLKLRDDLMVC-----KGS

---PEYSE---PTSPELKLWLHHLQRFKETASPDCVQEAVILSLIPLRL-EDVTCWAL--

-NQ---

>napa_LR1c

------MDI--RLMV-----------LSIILVAV---------SGRL----------HKR

LCPMSRYLSVASSDLTTLKQLQHDQEKNM--------STNAMRCYRRMLR--HKPSVCDL

TQRDRLILTLER-VSLTVDVLTNMSVS--AQPDTIKQSLMVFLKLRDDLKVC-----RGS

---PEYSE---PTSPELKLWLHHLQRFKETASPDCVQEAVILSLIPLRV-EDVTCWAL--

-NQ---

>napa_LR1d

------MDI--RLIV-----------LSIILVAV---------SGRL----------HKR

LCPMSRYLSVATSDITTLKQLQHDHEKNM--------STNAMRCYRRMLR--HKPSVCDL

TQRDRLILTLER-VSLTVDVLTNMSMS--AQPDTVKESLMVFLKLRDDLKVC-----RGS

---PEYSE---PTSPELKLWLHHLQRFKESASPDCVQEAVILSLIPLRV-EDVTCWAL--

-NQ---

>napa_L5i

------MDI--RLLV-----------LTLLIAVV---------CGRP----------HRR

LCPLSRYLSLPSSDMAILRQLQHGHENAM--------SINAMSCYRRMLR--HKPSACDL

TASDSLMLTLEQ-VSLVVEVLSDMSTY--AVSDPVTQALMLFLKMKDDLMTC-----RES

---EEYSG---PASSELKNWLHHLKHFQEVASPECVQDAVMLNLIPLR--EDVICWAL--

-SQ---

>New|ANQ43302.1_type_III_IFN_4_Xenopus_tropicalis

MATPEAMEIPIRLAA-----------MMVLLVTV---------TAHP----------HRR

HCHMSRYGSVSPSDIRAVRRLHNEH-----------------------------------

------------------------------------------------------------

------------------------------VSSQCVQDPVLLSLTQLLI-EDVMCWAN--

-KE---

>New|ANQ43305.1_type_III_IFN_1_Xenopus_tropicalis

------MYL--TAAV-----------VFTLLPTL---------GGHPT---------HRE

RCPLSGYETVLPSDIIAAKELQN--QTRM--------S---LKCHQGLRL--TSSPECDI

KGNGRLMLTLHR-ISLTVRVLKNMTKS--KL---VLRNLELFGALQETL-LC-----NTS

---HSYKV---PNHPVLESCAGLLATYTKETPAECIEQDVLLSLVWLLI-EDVRFLFD--

-GDQIE

>New|ANQ43345.1_type_III_IFN_2_Xenopus_laevis

------MYL--TAAL-----------FFALLTTL---------GGHPT---------HRE

RCPLSSYQTVLPSDITAARQLQN--QTRV--------S---LKCHQGLRL--SSFPECDI

KGNGRLRLTLHR-VSLAVRLLKNMTKT--KL---VLRNLELFGALHETL-LC-----NTS

---HSDKV---PTHPVLESCTDHLVTYTKDTSAGCIEQDILMSLVWLLI-EDLRFLVD--

-EDQRG

>New|ANQ43344.1_type_III_IFN_1_Xenopus_laevis

------MYL--TAAV-----------FFALLTTL---------GGHPT---------HRE

RCPLSSYQTVLPSDITAARQLQN--QTRV--------S---LKCHQGLRL--SSFPECDI

KGNGRLRLTLHR-VSLAVRLLKNMTKS--KL---VLRNLELFGALHETL-LC-----NTS

---HSDKV---PTHPVLESCTDHLVTYTKDTSAGCIEQDILMSLVWLLI-EDMRFLVD--

-EDQLG

>New|XP_008139537.1_IFN_L-3-like_Eptesicus_fuscus

-----MATS-------------------L-GAGLG--------QSRP-------------

-CPYFGFQSVLPWELEAFKKANESLEDLFL-LNDWSCI---SCLFPRTKVLRQ------L

QMWEHPLA-LGAKLT----VEGPWADL--SLGGGGVSWINLFTG--------A------T

ATL-------------------ELQKAPKKESQDCXEASVTFNLFCLLT-WHLKCVAS--

-EDLPL

>New|XP_016853758.1_Anolis_carolinensis

-----MIAV--AAKL-----------CLLASVALLISLENHDVDGAP-----------RR

TCYLPK----TPENRAAFRTIRNKYEDLRFDGPWQNCS---EKYLQPKMD--N------L

TKWEKMGA-MELQLNLFIPVLQNQSET--TFAKATSEVLEFLLPYREDLKVCMQH-RPSH

---HQESGQGF----KDGLQQFNTSNT--EQSPKCLEAAVGMNILSLLQ--EIKGISLHH

HHQPRH

>New|XP_015676150.1_Protobothrops_mucrosquamatus

MERNRLSAS--GPRK-----------WLLIPLALMILLKVPGVQSLP-----------VG

SCHFPQ----IAEHSDAFRQLRNRYEDERFKGPWKNCT---RQHLRGKMH--H------L

SKWEKLEA-VEAELALVVPVLQNLSEP--PFSKQASKILEFLQPLKDALAPCIRH-KPPH

---RQKSSQEF----QEAIQKFNASSV--QQSPKCLEAAVGLNIVRLLE--E-DIAQLQH

HFRHRH

>Gaga_1

------A----VGIL-----------LLLLLTAS---------ACN--------------

----------------------------D-------------------------------

--SLQLLRDMD--------TIL----D--TLQHLFKILSSIHRYTQH-LEQC------SS

---DT-------------------RSRTRLKHFSCLHTFLARAWFLHIH-N-L-------

------

>IFNA_human

------A----LTFA-----------LLLVLCSV---------GCD--------------

----------------------------R-------------------------------

--TLMLLAQME--------EF-----G--NIQQIFNLFSTLYQQLND-LEAC------TP

---LM-------------------KEDSILKYFQRITLYLIMRSFSLST-N-L-------

------

>IFN1_cami

------A----VHYQ-----------LFAML--L---------GCS--------------

----------------------------T-------------------------------

--TLNTLDEMS--------PLQ----N--NFQHLNKIFHKLVTQREV-VKDC-----SAS

---DS-------------------MRSALSTYFRKLKSF-TRAHLQQI----LTK-----

-GN---

>Xetr_1

------S----VSVL-----------LLITLPTK---------GKD--------------

----------------------------E-------------------------------

--VRTLLGNME--------FLK----N--MIHQFSVIFTDMSKMQTL-LYWY------SG

---DY-------------------SGLKELYEFRKMLKYLMEKVLLLVT-W-H-------

------

>New|IFN4_cami

---MSWI----VLPV-----------VLLYLCSP---------GTSG----HG-----P-

-----SLESL---------------NAAV-------------------------------

--TLQVLEEIR--------LFR----K--SSKFLNQVYQVIYELQNC-MTSS-----DLP

---DV-------------------HRSTIKARFANLEGFLTRKILQEVS-R-NHRK----

------

1. **IFN1 FULL:**

>oran_L3

MR---GHLLTG------------------AQAADPP--K------KQCYL--GKF-KSLS

PQELEAFKKAKDML-------------------------------LTD----R------T

ERLL--FLEA-----E-L----KLLG---------KM-SKSNLEGYLGRPLWTLRYIS-Q

ELQRCI----------------AQES-R-HSS--------------R------LTHWLHK

LQE-AREK------------------------------------------------

>gaga_L

MV---GVTIVG------------------AFPQVTP--K------KSCSL--SKY-QFPA

PLELKAVWRMKEML-------------------------------LTN----R------K

DRIT--LVEA-----E-L----DLLT---------NP-TTQRLAETCQQPLAFLTQVQ-E

DLRDCL----------------EAPSHQ-PSG--------------K------LRHWLQK

LET-AKKKET-------------AGCLLRC-AAQRED--CT---------------

>caan_L2

ML---RLLPL----------------------QETP--G------QRCGL--SRY-RFLP

PQELRAVKRMKEML-------------------------------LSD----H------R

DRVM--LAEA-----E-L----DLLE---------LP-AAPTFAETRQRPLDFLAQAQ-E

DLRSCM----------------EAP-HQ-PSR--------------R------LRNWLQK

LQT-AKETET-------------TSCLLQC-AALGEQ--CS---------------

>pesi_L3

TM---GYRVAE------------------AFPKGAL--R------TKCHL--AKY-KSLP

PRELEAFKKTKDLL-------------------------------LSD----R------K

DRVI--LVEK-----E-L----DFLE---------DV-EDPSLSKLLPRPLEILSQIR-E

DLRRCT----------------QTPSHS-HSK--------------R------LNSWLQN

LQA-SKETET-------------PACLLRC-AAYTEL--CV---------------

>gavga_L

------------------------------------------------------------

----------------------------------------------------R------K

DRVI--LVQA-----E-L----NFLK---------NI-EDPNLSEQLPRPLEFLTHIG-E

DLKSCT----------------HHHSHK-KSE--------------K------LSSWLQK

FHE-AKNKET-------------RECLLAC-AALKDD--CIS-------P------

>hosa_il28a

MT---CTPVTG------------------AVPVARLHDA------RGCHI--AQF-KSLS

PQELQAFKRAKDLL-------------------------------LKD----C------R

ERPM--ALEA-----E-L----ALLE---------AT-ADTALVDVLDQPLHTLHHIL-S

QFRAC-----------------PQPT-R-TRG--------------R------LHHWLYR

LQE-APKKES-------------PGCLLNC-VASGDL--CV---------------

>hosa_il28b

MT---CMPVTG------------------AVPVARLRDA------RGCHI--AQF-KSLS

PQELQAFKRAKDLL-------------------------------LKD----C------K

ERPV--ALEA-----E-L----ALLE---------AT-ADTALGDVLDQPLHTLHHIL-S

QLRAC-----------------PQPT-R-TRG--------------R------LHHWLHR

LQE-APKKES-------------PGCLLNC-VASGDL--CV---------------

>hosa_il29

MA---WTVVAG------------------PVPTSKPTTG------KGCHI--GRF-KSLS

PQELASFKKARDLK-------------------------------LKN----W------S

ERPV--ALEA-----E-L----ALLE---------AA-AGPALEDVLDQPLHTLHHIL-S

QLQAC-----------------PQPT-R-PRG--------------R------LHHWLHR

LQE-APKKES-------------AGCLLKY-VADGNL--CLH-------P--EST-

>xetr_L1

-------MEVT------------------AHP-----HR------RHCHM--SRY-RSVS

PSDIRAVRRLHNPF-------------------------------SDG----I------K

DRLI--LTLE-----R-V----TLLT---------NM-TESPLAKLLSLPLTMLLSLE-D

DLKICR----------------PLYSDP-PSE--------------Q------LMPWLHH

LKH-FREKVS-------------SECVIMC-WANNE--------------------

>napa_LR1a

---------VS------------------GRL-----HK------RLCPM--SRY-LSVA

SSDITTLKQLQHMS-------------------------------SNA----M------R

DRLI--LTLE-----R-V----SLLT---------NM-SMSAQPDTIKQSLMVFLKLR-D

DLLVCR----------------PEYSEP-TSP--------------E------LKLWLHH

LQR-FKETAS-------------PDCVVTC-WALNQ--------------------

>chpi_L2

---------VD------------------AFAEGAP------------------------

------------LR-------------------------------DPA----A------N

QNLL--VLEK-----K-V----DVIQ---------NL-SDPELVRNASKPLEILASIQ-E

DLRSCP----------------QQPV---------------------------LARWLRN

AYA-GKSEGS-------------ARCLLQN-VAHNES--CH---------------

>oran_L4

---------LE------------------VSCSSFM-DG------KKCYL--AHY-GSLD

PQVLRDVKDLQN------------------------------------------------

GRLL--LLER-----E-L----AHLR---------NL-SGLDLGRNATRPLQLLAAIC-E

DLASC----------------------S-ARQ--------------R------EMSWLRS

KAK-AKKEVT-------------PRCLLRQ-ATYLKP--CD---------------

>scca_L

-------MATF------------------LAPSLSP-GQ------DGCSL--SRY-ARLP

PSVFKLFGDFHRQE-----PG---------------------------------------

ERLL--LVEA-----E-F----RLMQ---------EL-ETSILQNLTKKVLEVLYQMC-W

NLGRCL----------------TQKDQK-YPK--------------Q------LKKFLRN

LKH-AGRLGR-------------T---LSC-VASGED--C----------------

>Xetr_3

MFKRNTVTTLL------------------VLIPIVQ--S------QNCKWLQPKQ-EYLN

RQTLKTFEEMNPPE-------------------------------DYD----E------S

SQME--EMVL-----A-L----NEYM---------KH-HESMGCKQQAERFQQLLYYQIN

QLEACI----------------TAENPV-FNQ--------------T------ISDQYQA

LEQ-ILQEKN-------------TACTARR-QRLLQR--TA---------------

>Xetr_4

--------MLL------------------SLTSIVH--S------QSCKWLHPKQ-EYLN

TQILKAFNEMMPLK-------------------------------ETE----E------I

SQVE--AGAL-----A-L----NEYM---------KH-HESMGCKQQAERFQQLLYYQIH

QLEACV----------------TEENDL-LKE--------------S------ISEEFNL

LET-MVLEKN-------------SACVLRR-QRLLQR--PQ---------------

>Napa_1

---------------------------------MVS--A------QTCKWLHRNQ-QEWT

RQILHNFNQMVPAE-------------------------------ETG----Q------P

TQAE--SAAI-----A-A----NQYR---------RN-QDRMDYHLPAEKLQELLNYQEQ

HLSDCI----------------GAENQL-FIQ--------------G------ISQRFNT

LQR-ILEEQD-------------TACAMRN-QKSHD--------------------

>Napa_2

---------------------------------MVS--A------QTCKWLHRNQ-EAWT

RQILHNFNQMVPAE-------------------------------KTG----Q------P

TQAE--SAAI-----A------DEHI------------------------------YQLA

RPSRCC----------------TAEGKY-VSY--------------T------VNQGREA

VKP-VLA--------------------VSR-YILLVK--PQL--------------

>IFN1_coela

--------MTL------KM--LLAFC---LLL----LVSNGT-FCQDCKKWVKQ-----Y

NKGLQYLEAMGGEFPLKCLAQ-----------------------LDVP----RKVVLRHS

KGER--RIEL-----V-H----DTLEHISKTYS-NN--TSTKWDEENLKKFQNVIHLESE

ELRACL--------------QERVSNKN-TQWRK----------KMT------LSRHFKE

TGN-FLQRQNYSSCAWETVRAITRMILQLI-Q------------------------

>IFN2_coela

--------MAL------KT--FVALC---LLL----FVPIGI-FCQECEELNSQQ-RLRI

RESLQELEGVGGKFPSQCLAQ-----------------------FNLH----KKVLLKHS

KGER--RITL-----V-Y----EILQQINRIYR-KN--PSATWDQNKLERFQNVLHSQTE

ELWKCL--------------EKKMSNMN-SQWNN----------AMK------LSKNFKE

MEK-FLKHQNYSSCAWELVRTITRRVLQQV-ER---------K-------A-----

>IFN3_coela

--------MAL------KC--LWTMF---LLL----LDFPVA-FPEQCNWVHLHQ-KY-S

TSKLPLLDEMGAGFSESCMIE-----------------------IVKD----KKLTLKFP

NSDH--MIETDIMPTV-C----EILNFIGNIYN-KNL-QLVPWDKKKIEHFQTVLYHEVE

EIKKCL--------------PGEKTNAN-SHSNS----------NMK------LQDYFST

LEN-FLEQKEYSPCAWEVVRAHIRTLLQFT-DRLTTV--ITKN-------E-----

>IFN4_coela

--------MAL------KC--LWTMF---LLL----LDFPVA-FPEQCNWVHLHQ-KY-S

ISKLQLLDEMGAGFPPHCINE-----------------------KGVD----EKVTLKFP

KQDH--MIQMEIMPTV-C----EVLNFTGNIYN-KNL-QFVPWNKRKVQHFQTVLHHEVE

ELKKCL--------------PEEKTNAN-SHSNS----------NMK------LQDYFSK

LEN-FLEQKEYSLCAWEIVRVHIRKLLQLT-DRLTTA--IRKT-------E-----

>IFN5_coela

--------MAQ------KF--QIISL---ILF----IVSQVR-ADDQCTWSTAQQ-MHLN

KRNLNLTDDMGKFSPAECTTE-----------------------IQEV----QKMRLRFP

ENE-------DVIFIV-Y----TTLRHISKIYS-KNL-QPVSWNKTVLHEFQAAVHSQVE

ELEKCL--------------MEKMVDHY-LERKV----------ELK------LRNYFKL

LEK-MLAEKENNQCAWRFIRAQVRKFLYRI-DQLTAW--IGKM-------KNQSS-

>IFN6_cami

--------MAV------HY--QCGLS----LF----AMLCVS-LTLGCSTLRLQK--ILI

ATTLNTLDEMGGHVPRHCVAVGAELRIA--S-------------PDLR----L--LLQPL

QNND--RILL-----L-H----KTFQHLNKIFH-KNM-KSVTWDLTQVNHFRELLVTQRD

VVKDCI--------------QDSAS--D-SMLSA----------LST------IHTYFRK

LKK-FLKQQRYSACAWEVIRMETRARLQQI-LILTAR--MTKG-------N-----

>IFN1_cami

--------MAV------HY--QCGLS----LF----AMLCVS-LTLGCSTLRLQK--ILI

ATTLNTLDEMGGHVPRHCVAVGAEQGIA--S-------------PDLR----L--LLQPL

QNND--RILL-----L-H----KTFQHLNKIFH-KNM-KSVTWDLTQVNHFRELLVTQRE

VVKDCI--------------QDSAS--D-SMLSA----------LST------IHTYFRK

LKK-FLKQQRYSACAWEVIRMETRARLQQI-LILTAR--MTKG-------N-----

>IFN1_sqac

--------MVF------PS--VWRLW---ILL----VLLPGT-LSQDCQRLQLLD--NIN

NQALDALREMGGPIPLHCKTERTSLRAK--S-------------LDLH----Q--LSKRL

QTPD--RIQI-----V-H----QTLRHLTKIYS-MNL-GSVTWPRDKVENFRLLLDRQLG

ELEECV--------------RKPVP--E-SRPRR----------NAP------IHKYFRK

VEK-FLKQKRFSDCAWEIIRAETRARLQQI-LFITAK--IRRR-------S-----

>IFN2_sqac

--------MVF------PS--VWRLW---ILL----VLLPGT-LSQDCQRLQLLD--NIN

NQALDALREMGGPIPLQCKTERTSLRTK--S-------------LDLH----Q--LSKRL

QTPD--RIQI-----V-H----QTLRHLTKIYS-MNL-GSATWPRDKVENFRLLLDRQLG

ELEECV--------------RKPVP--E-SRPRR----------NAS------IHKYFRK

VEK-FLKQKRFSDCAWEIIRAETRARLQQI-LFITAK--IRRR-------S-----

>IFN1_leer

--------MVL------GS--VCRVW---IVC----VLLSGS-LSLGCERLQLLQ--VLN

TDTLGKLNEMGGHLPRQCVTERRSLKTK--P-------------LNLV----K--LSMGV

TAQD--RIQI-----V-H----QTLHHLRRIYS-MNL-SSVTWVQATVEHFRLLLDRQFR

ELEVCV--------------RKPST--G-SRARK----------NAT------VRKYFRK

LRK-FLKLKGFSDCAWEITRAETRAHLQQL-LLIMAT--ISRE-------Q-----

>Leer_25

------------------------------------------------------------

------------------------------------------------------------

------TLLV-----V-D----QMLRQFRKIYS-MNL-ASVTWLQDKVENFRLLLDRQIR

ELENCV--------------RNTGS--E-TRPRR----------SAA------VHNYFRK

LGK-FLKRK-----------------------------------------------

>Leer_37

------------------------------------------------------------

------------------------------------------------------------

-TQE--RIHT-----A-H----QTLQQINNVYS-MNL-DSITWAQHKVENLRLLLDRQLR

TLEECV--------------KKPGS--K-STSKR----------NTR------ISNYFRK

LRK-FLKRNRFSDCAWEITRTE----------------------------------

>Scca_1

-------------------------------------VISGE-IVWGFNEILFYS--S--

---------QGGSFPRHCIKHRHALKTK--P-------------LNLV----K--LSKGL

EKED--QIQI-----L-H----QTLRHISKIYS-MNL-GSVTWDRDTVENLRLLLDRQLS

ELE---------------------------------------------------------

--------------------------------------------------------

>Leer_3

------------------------------------------------------------

------------------------------------------------------------

-DMD--TAVV-----V-Y----QVVSQFKEIYH-MDR-TSVTWPQDVMKRFGIKLDAQSS

ILENCV--------------RNAGS--D-AQTQK----------KAT------IQEYFKK

LSE-FLNRERFSACAWE---------------------------------------

>Leer_4

------------------------------------------------------------

------------------------------------------------------------

-APD--LAGI-----V-Y----QVVTQYNRIYN-MDR-TSVTWPQIIMTSLGFHLDVQTT

VLEDCL--------------THRGA--M-AQRQN----------RET------IHDYFRG

LSE-FLNRERFSACAWEAIREEMILWYQQV-FKFF---------------------

>Leer_35

------------------------------------------------------------

------------------------------------------------------------

-SPD--TVGV-----D-Y----KVVSQFLGIFH-KNR-TLVTWAKKQTKKVELKLNKLST

WLKDCM--------------KNTGA--K-AQPQK----------RAA------LGMYFKG

LRK-FLKQKRFSACAWEGIREQIISFYQEM-FKRDSV-------------------

>IFN2_cami

--------MPL------RC--VWKLC---LCL----ALLATQ-TPSLACNLPLHN--LMC

QRSLNLLLWMRDSIPFHCVREMGST---------VDLR------LNLR----N--VTGPL

QPGD--RMQV-----Y-L----QTLHHLNEIYS-NNV-TS-TWDQEKILGFRFVLDEQQM

EMEKCA--------------KEPAS--D-AMVHT----------TSA------IRTYFTK

LGR-FLRQKRFSACAWEVIRAKTSRRLQEM-LTLAMK--EAKT-------R-----

>IFNA_human

MALTFALLVAL------------------LVLSCKSSCS------VGCDLPQTHL-GS--

RRTLMLLAQMRRISLFSCLKDRHDFGPQEEF--------------G-N----Q------F

QKAE--TIPV-----L-H----EMIQQIFNLFS-TKD-SSAAWDETLLDKFYTELYQQLN

DLEACVIQGVGVTE--------TPLMKEDSIL--------------A------VRKYFQR

ITL-YLKEKKYSPCAWEVVRAEIMRSFSLS-TNLQES--LRSKE------------

>IFNA_horse

MALPVSLLMAL------------------VVLSCHSICS------LGCDLPHTHL-GN--

TRVLMLLGQMRRISPFSCLKDRNDFGPQEVF-------------DG-N----Q------F

RKPQ--AISA-----V-H----ETIQQIFHLFS-TDG-SSAAWDESLLDKLYTGLYQQLT

ELEACLSQEVGVEE--------TPLMNEDSLL--------------A------VRRYFQR

IAL-YLQEKKYSPCAWEIVRAEIMRSFSSS-TNLPQS-------------------

>IFNA_pig

MAPTSAFLTAL------------------VLLSCNAICS------LGCDLPQTHL-AH--

TRALRLLAQMRRISPFSCLDHRRDFGPHEAF-------------GG-N----Q------V

QKAQ--AMAL-----V-H----EMLQQTFQLFS-TEG-SAAAWNESLLHQFCTGLDQQLR

DLEACVMQEAGLEG--------TPLLEEDSIL--------------A------VRKYFHR

LTL-YLQEKSYSPCAWEIVRAEVMRSFSSS-RNLQDR--LRKKE------------

>Oror_10

MAPTVSLLLAL------------------VLLSCHSNCS------LGCDLPQTHL-AN--

TRALMLLQQMRRISPFSCLKDRNDFGPQEAF-------------GG-N----Q------F

QKAQ--AIAV-----V-H----EMIQQTFQLFS-TEG-SAAAWDETLLDKFCTALYQQLT

DLQACLMQEAGLEG--------TPLLKEDSIL--------------A------VRKYFHR

ITV-YLQEKKYSPCAWEIVRAEVMRSFSSS-TNL----------------------

>Loaf_11

MAFSFLLLIAL------------------VVLSCNSTCS------LGCDLPQSHL-AN--

RRTMMLLGQMRRISPFSCLKDRNDFGPQEEL-------------DG-N----K------F

QKAQ--AISV-----H-H----EMIQQTFNLFS-LQA-SSAAWDKTLLDKLYTGLYQQLN

DLEVCLMQEMGVEE--------APVINEDSML--------------A------VRKYFQR

ITV-YLTEKKYSPCAWETVRAEVMSSFSAS-TNWKER--LRSKEGDLAP-------

>IFNA_cow

MAPAWSFRLAL------------------LLLSCNAICS------LGCHLPHTHL-AN--

RRVLMLLGQLRRVSPSSCLQDRNDFAPQEAL-------------GG-S----Q------L

QKAQ--AISV-----L-H----EVTQHTFQLFS-TEG-SATMWDESLLDKLRDALDQQLT

DLQFCLRQEEELQG--------APLLKEDSSL--------------A------VRKYFHR

LTL-YLQEKRHSPCAWEVVRAQVMRAFSSS-TNLQES--FRRKD------------

>Ptva_8

MALLFSFLMAM------------------VVLSCQSICS------LGCDLPQTHL-VN--

RRALMLLGQMRRISPFSCLKDREDFGLQGAF-------------GG-N----Q------F

QEAQ--AIAV-----F-H----EMTQQTFLLFC-TEV-LSAAWDETLLGRFCNGLYQQLD

HLEACQTQELGAEE--------TPLLDEDSTL--------------A------VRKYFQR

INL-YLQEKKHSPCAWEIVRAEIMRSYSLS-THLKEK--SRSKD------------

>Ereu_6

MAPSSLFLKAL------------------LVLSCSYIFG------LGCDLPQSHP-VN--

RRPLLLLGQMRRLPPFSCLKDRHDFAPQEVF-------------DG-Q----Q------F

QKAH--ALSV-----L-H----EMLQQIFHLFS-TKH-SSADWDEGLLNSFCAELHQQLN

VLEGCQTQEVRVEQ--------TPRMK-DSIL--------------A------MKRYFQR

ITM-YLREKKYSPCAWEIVRVEIIRAFSLS-TKLQEK--LRSKD------------

>IFNA_mouse

MARLCAFLVML------------------IVMSYWSTCS------LGCDLPHTYL-RN--

KRALKVLAQMRRLPFLSCLKDRQDFGPLEKV-------------DN-Q----Q------I

QKAQ--AIPV-----L-R----DLTQQTLNLFT-SKA-SSAAWNTTLLDSFCNDLHQQLN

DLQTCLMQQVGVQE--------PPLTQEDALL--------------A------VRKYFHR

ITV-YLREKKHSPCAWEVVRAEVWRALSSS-VNLLPR--LSEKE------------

>IFNA_sheep

MAFVLSLLMAL------------------VLVSYGPGGS------LGCDLSQNHL-VG--

SQNLRLLGQMRRLSLRFCLQDRKDFAPQEMV-------------EG-G----Q------L

QEAQ--AISV-----L-H----EMLQQSFNLFH-TEH-SSAAWDTTLLEHVRTGLHQQLD

DLDACLGEVTGEED--------SALGRTGPTL--------------A------MKTYFQG

IHV-YLKEKGYSDCAWEIVRLEIMRSLSSS-TSLHKR--LRMMDGDLSSP------

>IFNO_cow

MAFVLSLLMAL------------------VLVSYGPGGS------LGCDLSPNHL-VG--

RQNLRLLGQMRRLSPRFCLQDRKDFAPQEMV-------------EV-S----Q------F

QEAQ--AISV-----L-H----EMLQQSFNLFH-KER-SSAAWDTTLLEQLLTGLHQQLD

DLDACLGLLTGEED--------SALGRTGPTL--------------A------MKRYFQG

IHV-YLQEKGYSDCAWEIVRLEIMRSLSSS-TSLQER--LRMMDGDLKSP------

>IFND_human

MAFVLSLLMAL------------------VLVSYGPGGS------LGCDLSQNHL-VG--

RKNLRLLDEMRRLSPHFCLQDRKDFAPQEMV-------------EG-G----Q------L

QEAQ--AISV-----L-H----EMLQQSFNLFH-TEH-SSAAWDTTLLEPCRTGLHQQLD

NLDACLGQVMGEED--------SALGRTGPTL--------------A------LKRYFQG

IHV-YLKEKGYSDCAWETVRLEIMRSFSSL-ISLQER--LRMMDGDLSSP------

>Oror_9

MAFVLPLLTAL------------------VVFSYGPGGS------LGCDLSQNHR-IS--

RKNFMLLGQMRRISPRFCLKDRKDFGPQDMV-------------DG-S----Q------L

PKAQ--ATSV-----L-H----EMLQQVFCLFH-TER-STATWDTSLLDKLRTGLHQQLE

DLDACLVQAMGDEE--------TALGVTGPTL--------------A------VKRYFQG

IHL-YLKEKKYSDCAWEIVRVEIMRSLSSS-TNLQER--LRIMNGDLGSP------

>IFNT_cow

MAFVLSLLMAL------------------VLVSYGPGRS------LGCYLSEDHL-GA--

RENLRLLARMNRLSPHPCLQDRKDFGPQEMV-------------EG-S----Q------L

QKDQ--AISV-----L-H----EMLQQCFNLFH-IEH-SSAAWNTTLLEQLCTGLQQQLE

DLDACLGPVMGEKD--------SDMGRMGPIL--------------T------VKRYFQD

IHV-YLKEKEYSDCAWEIIRVEMMRALSSS-TTLQKR--LRKMGGDLNSL------

>IFNT_sheep

MAFVLSLLMAL------------------VLVSYGPGGS------LGCYLSQRLL-DA--

RENLRLLDRMNRLSPHSCLQDRKDFGPQEMV-------------EG-D----Q------L

QKDQ--AFPV-----L-Y----EMLQQSFNLFY-TEH-SSAAWDTTLLEQLCTGLQQQLD

HLDTCRGQVMGEKD--------SELGNMDPIV--------------T------VKKYFQG

IHD-YLQEKGYSDCAWEIVRVEMMRALTSS-TTLQKR--LTKMGGDLNSP------

>IFNT_goat

MAFVLSLLMAL------------------VLVSYGPGGS------LGCYLSRRLL-DA--

RENLRLLDRMNRLSPHSCQQDRKDFGPQEMV-------------EG-D----Q------L

QKDQ--ASCV-----L-Y----EMLQQSFNLFY-TEH-SSAAWDTTLLDQLCTGLQQQLD

HLDTCRGQVMGEKD--------SELGNMDPIV--------------T------VKKYFQG

IYY-YLQEKGYSDCAWETVRVEMMRALTAS-TTLQKR--LTKTGGDLNSP------

>Loaf_4

MALLLSLLTAL------------------VVFSCGPAPS------LGCDLPQNHV-AS--

EKTVDLLDQMQRCPTFFCLDDRKDFRPQEMV-------------DG-S----Q------L

QKAQ--AIAF-----L-H----EMLQQIFDLFR-TMD-SFAAWNTTLLNQLLNGLPEQQE

DLETCFMQAMEEGK--------SALPIEGPAL--------------A------VKEYFEG

IRF-YLKEKEYSDCAWEFVRVEIRRSFSSS-TALQER--LRRKDGDMSSS------

>Loaf_5

MAFLLFLLTAL------------------VVFGCGPAPS------LGCDLSKKHL-TS--

KKTFVVLDQMRRLSPFSCLKERKDFRPQEMV-------------DG-S----Q------L

QKAQ--VISV-----L-H----EMLQQIFNLFH-TKD-SSAAWNTTLLDQLHSGLYLQLE

DLEACLVQAMEEEE--------SVLAIESSAL--------------A------VKRYFQG

IHS-YLKEKEYSDCAWEIVRVEIKRSFSSS-TNLQER--LRRKHGDMGSS------

>Dano_12

MALQLSLLMAL------------------VVFSCGPVPS------LSCDLPQSQL-VD--

RKTFVLLGQMGRISPFSCLKDRADFRPQEMV-------------DG-S----Q------V

QKSQ--AKFV-----L-H----EMFQQIFNLYH-TEG-SSAAWNMTLLDQLLSTLHEQLE

DLEACLLQEMGEEE--------TLLGIEGPVL--------------A------MRRYFQG

IRL-YLQEKKHSDCAWEVVRMELRRAFSSS-PNLKER--L----------------

>IFNO_human

MALLFPLLAAL------------------VMTSYSPVGS------LGCDLPQNHL-LS--

RNTLVLLHQMRRISPFLCLKDRRDFRPQEMV-------------KG-S----Q------L

QKAH--VMSV-----L-H----EMLQQIFSLFH-TER-SSAAWNMTLLDQLHTGLHQQLQ

HLETCLLQVVGEGE--------SAGAISSPAL--------------T------LRRYFQG

IRV-YLKEKKYSDCAWEVVRMEIMKSLFLS-TNMQER--LRSKDRDLGSS------

>Dano_2

MAFPVSSLVVL------------------MMIFSSPIGS------FSCGLPQSLV-RK--

QETFTVLSQMGTISLLSCLKDRTDFRPQEMM-------------DG-S----Q------V

QKTQ--AMSV-----L-H----EMLQQIFHLFH-TEG-SSAAWNTTLLDQLRSGLHRQLE

DLETCLLQEMG-ED--------SVLAMEGPTL--------------A------VRRYFQR

IRV-YLQKKKHSDCAWEVVRVEIRRCFLFI-NVLTRE--LRK--------------

>Ptva_4

MAPLLSLITAM------------------LVFSYGPSGS------LSCDLSQNHQ-VN--

KESIVLLHQMQRISSFRCRKDRKNFGPQEMV-------------DG-S----Q------V

QEAQ--AISV-----L-H----EMLQETSNVFG-SEH-SSAAWNTTVLHGLLSRLHWQLE

DLGTCLVLQMKEAE--------SALGMEAPTL--------------A------VKRYFQG

IRL-YLKEKQYSDCAWEIVRVEIKRAFSLS-TNLREM--LRNQDGDLRSP------

>Ptva_16

MAFLVSSLMAL------------------VVIFSSPISS------MICDLPQSLL-GK--

QETSTALNQMQRISSFLCQKDRKDFRPRKMV-------------DG-S----Q------V

QKAQ--AISV-----L-H----EMLQQTFDVFG-TKQ-SSAAWNTTLLHGLLSGLHRQLE

DLGTCLVPEMKEVE--------SVLGTEDPTL--------------A------MKRYFQG

IHL-YLEEKQYSDCAWEVVRVEIRRYLFVV-NKFTRK--EI---------------

>IFNO_horse

MAFSVSSLMAL------------------VVISSSPVSS------MSCDLPASLL-RK--

QETLRVLHQMETISPPSCLKHRTDFRPQEQL-------------DG-R----Q------F

PEAQ--ATSV-----L-Q----EMLQQIVSLFH-TER-SSAAWNTTLLDRLLAGLHQQLE

DLNTCLDEQTGEEE--------SALGTVGPTL--------------A------VKRYFRR

IRL-YLTEKKYSDCAWEIVRVDIMRSFSSS-ANLQGR--LGMKDGDLGSP------

>ereu_1

MALLLSLLMPL------------------VVLSCGPSGS------LGCELPQSHL-DS--

IQNLRLLGQMRRLSPLSCLKDRRDFRPWKQV-------------DG-S----Q------L

QKAR--VMSV-----H-H----EMVQQAFQLLL-SER-ASAAWDKTLLDQVRTGLHQRLE

HLDSCLVQLVTEED--------SSQSYGRASL--------------E------MKRYFQR

IRL-YLKEKKYSGCAWEVVRVEIMRSFSLS-TTLQER--VM--DEDMGLP------

>Ereu_9

MAFSVSSLVVL------------------VMILSSPIYS------TSCNLPLSLL-EN--

QETVRALDQVGTVSLLSCLNYRTNFKAKEQL-------------DG------Q------F

QKAQ--AMSI-----V-H----ETVQQVFHLFF-LVN-VSAAWDKTLLDLVRTGLHQQLE

YLDSCLVQLGTEED--------SALSYGSASL--------------E------MKRYFRR

IRL-YLKEKKYSVCAWEVVRVEIKRSLSSS-AMLQNS--L----------------

>Dano_10

MAPPVSVLKTL------------------LMLCSIPAC-------LGCDLPLIY--GH--

QEPFMLLHQMGRLSILSCLKDRTDFQPQELM-------------DG-I----Q------L

DKMH--ATTL-----L-H----EVVQQIFNLFS-TSG-SLATWDDTLLDRFLIGLHQQLD

NLETCLGKEKEEDQ--------THLGSENSRL--------------A------VKRYFQG

ISQ-YLTEKQDSPCAWEVVRVEIRKCFLFI-NKLQGK--LRK--------------

>Modo_6

MTSWSLLPVAL------------------ALLCSSTLCS------LDCDLTLGL------

QEDFSLLNQMSTSSLVPCLKDGINFNPKEAM-------------DR-S----Q------L

QKEN--ATVI-----V-L----EMVQQIFTLFS-QNT-TPATWNQTQVIQLLIRLDQQLE

QLERCLGQNVKWEE--------FSLRSEKTRF--------------A------LKSYFQG

ISQ-YLQGKEYSPCAWEIVRVEIRRLFLFM-SKLARK--LRD--------------

>Dano_1

MDHM-YLLLAG------------------LMLCSSLDCS------LGCPLPRSQL-ES--

KEIFTLLRQMNRIPSHSCLNDRVDFKPWKAE-------------TV-T----Q------I

PKTQ--ATCF-----S-Y----EMFQQIFNLFQ-KEN-SRAAWDNSLLDELLSRLDHNLE

QV-----EQMKVE----------NLPCADLGT--------------L------VRDYLQG

TDG-YLNEKKYSSCAWEVVRGEPEMCFPLI--------------------------

>Oror_2

MAQI-YLLVAG------------------VLLCSIPAYS------LGWNLPRSHQ-EN--

KDVFQHLEQLQRIPSQWCLKDRTDFKPWKRE-------------NI-T----P------I

QVTQ--GTCH-----H-H----LMLQQIFNLFT-TED-SRAAWNNTLLDKLLSSLHLRLH

RL-----EQMKKD----------NLDCRDLGR--------------A------AREYFHG

IHV-YLKAKEYSPCAWEVVRVEIKRCLSLM--------------------------

>Ptva_17

MAQCSSWLAAG------------------EMLSFILICS------LGGDVPWIHL-EN--

RKIVSLLRELEVIPSHFCLKDRTDFKPWERG-------------SI-T----E------I

QKTQ--RTCF-----H-H----LILQQIFSLLN-AED-SHAAWNRTLLYQLLSRLHHSLE

EL-----DQTNEG----------NLVCPDLGI--------------L------VWNYFQG

IHN-YLKQKKYSTCAWEVVRVEITARLFLM--------------------------

>Ereu_8

MFQ--FLLMTG------------------VMLSSILACS------YGQD----HP-EK--

RVILMLLTQLKNTPSLSCLKDRTDFHPWSRG-------------EI-T----Q------I

HMAQ--GPCF-----Q-K----LMLQQVFRLFN-TEA-SRAAWNNSLLDRLLSSLYDSLE

QL-----EQMEV-----------SLACPSVGT--------------D------ALKYFQR

IKI-YLKAKKYSACAWEIVRSEIEARFFLI-LGTLRR--LGQRTESLGPP-LRAA-

>Loaf_1

MALPISVLMAL------------------VMFCCRPACF------WCCDLPLSH---N--

QETFTLLNQMERISLLSCLKDRTDFRPQILM-------------DM-N----Q------L

EKTQ--AAVL-----L-Y----EMLQQTFNLFS-RSD-SLEAWDETFLDKFLLGLYQQLN

DLEICFEKERKVEQ--------IPLGTEN-----------------S------VKSYFQG

IGL-YLKEKEHSLCVGGCQSGN-QKMLSLH--------------------------

>IFNE_human

MIIKHFFGTVL------------------VLLASTTIFS------LDLKLIIFQQ-RQVN

QESLKLLNKLQTLSIQQCLPHRKNFLPQKSL-------------SP-Q----Q------Y

QKGH--TLAI-----L-H----EMLQQIFSLFR-ANI-SLDGWEENHTEKFLIQLHQQLE

YLEALL--EAEKLS--------GTLGSDNLRL--------------Q------VKMYFRR

IHD-YLENQDYSTCAWAIVQVEISRCLFFV-FSLTEK--LSK--------------

>IFNE_cow

MINKAFFEIVL------------------VLLASSTVCS------QELKLVLCQQ-RRVN

QESLKLLNKLQTSSVQQCLPHRKHFLPQKSV-------------NP-H----Q------Y

QKGQ--VLAI-----L-H----EMLQQIFSLFR-AIV-SLDGWEESHTEKFLVELHQQLE

YLEALL--QAKQKS--------DTLGSENLRL--------------Q------VKMYFQR

IHD-YLESQDYSSCAWTIVQVEINRCLFLV-FRLTRK--LSE--------------

>Oror_7

MINKPFFDIVL------------------VLLASSSVCS------RELKLVLFQQ-KRVN

RESLKLLNKLQTSSIQQCLPHRKNFLPQKSM-------------NP-H----Q------Y

QKGQ--ALTI-----L-H----EMLQQIFNLFR-AII-SLNGWEESHMEKLLIELHQQLK

YLEALR--QAEQKR--------DTLGSENLRL--------------Q------VKIYFQR

IRD-YLENQDYSTCAWTIVQVEINRCLFFV-FQLTGK--LSKQ---------ET--

>Ptva_15

MISKYFFEVVL------------------VLLASSTVFS------LELKLVLFQQ-RRVN

RENLKLLNKLQTPSIHQCLPHRKNFLPQKSL-------------NP-H----L------Y

QKGC--ALAI-----L-H----ETLQQIFSLFG-ANI-SLDGWEESHMEKFLIELHQQLE

YLETLQ--QAEQKS--------GILGSENLRL--------------Q------VKMYFQR

IRD-YLETQEYSRCAWTIVQVEINRCLFFV-FQLTGK--LSKQ---------DP--

>Dano_16

MINKHFFEIVL------------------VLLASSTLFS------LELKLVLFQQ-RQVN

RESLKLLNKLHT-SIQQCLPHRKNFLPQESM-------------NP-Y----Q------Y

QKGH--AVAI-----L-H----EMLQQIFNLFR-EKL-SLAIWEESQVEKFLIELHQQLE

HLEALQ--EPELKS--------DTLDSETFRL--------------Q------VKTYFRR

IRD-YLENQEYSSCAWTIVHVEINRCLFLF-TDSQES-------------------

>Loaf_2

MINKYFFETVV------------------VLLSSSMIFS------LELKLVHFQQ-R-MN

RESLKLLNTLWSSSIQQCLPHRANFAPQKSM-------------NP-H----Q------Y

HKGH--AVAI-----L-H----EMLQQIFNLFR-TNL-VLGSWEERHMEKFLIELYGQLE

HLEALL--EAEQKS--------GSLGTENLRL--------------Q------VKMYFQR

IHN-YLENQKYSSCAWTIVRVEIIRCLFFV-FRLTGK--LSQY---------DP--

>Ereu_10

MISKHIF--VL------------------ILLASSPIFS------LELKLFLVQL-RRLN

RESLKLLNIRQTSSIQRCLPHRKNFLPLKSP-------------SP-H----W------Y

QTEH--ALAI-----L-H----EMLQQIFNLFR-VNI-SLDDCEESYMEKFLMELHQQLE

LLEAFL--EAEQNS--------NTLSSENLRM--------------Q------VKMYFQR

IHN-YLGKQGYSNCAWTIVRVEINRCLLFM-LRLTTK--LSKQ---------DF--

>IFNE_mouse

MVHRQLPETVL------------------LLLVSSTIFS------LEPKRIPFQL-W-MN

RESLQLLKPLPSSSVQQCLAHRKNFLPQQPV-------------SP-H----Q------Y

QEGQ--VLAV-----V-H----EILQQIFTLLQ-THG-TMGIWEENHIEKVLAALHRQLE

YVESLL--NAAQKS--------GGSSAQNLRL--------------Q------IKAYFRR

IHD-YLENQRYSSCAWIIVQTEIHRCMFFV-FRFTTW--LSR--------------

>Oran_1

MTNRSSLPFVL------------------WLLLPTTIMA------QGYPKLYSHQ-WLSN

WQSLHLLDEMGGQFPLHCLKEKTNFKPAEMM-------------HP-H----Q------F

QQEN--ATEA-----I-H----DLLQNIFNIFG-RNH-SQTGWDEATVEKFLHGVHKEMM

RLELFE--EMGWEN--------STLRGDV-SL--------------H------IKSYFKG

MMD-YLKGRDYSSCAWEVTRMEAKRCFLVM-YRLTRK--LKK--------------

>Oran_7

MTNAGLIQIVL------------------VLLVSTSTVS------LSCSLLHT----VCM

EQSLKRLDRMQGKSLLSCLKDRKDFQPQELV-------------EA-G----P------F

KEGN--RAVA-----V-H----ELLQQIFTIFS-QNL-SQTGWDQSEVENFLHGLHRQLE

ELEVCQ--GTDTRW--------ASVGSDILRL--------------R------LKSYFRS

ISL-YLRDKDYSSCAWEIVRAQIRRC---I-FQFMRR--LRN--------------

>IFNB_human

MTNKCLLQIAL------------------LLCFSTTALS------MSYNLLGFLQ-RSSN

FQCQKLLWQL-NGRLEYCLKDRMNFDPEEIK-------------QL-Q----Q------F

QKED--AALT-----I-Y----EMLQNIFAIFR-QDS-SSTGWNETIVENLLANVYHQIN

HLKTVL--EELEKE--------DFTRGKMSSL--------------H------LKRYYGR

ILH-YLKAKEYSHCAWTIVRVEILRNFYFI-NRLTGY--LRN--------------

>IFNB_pig

MANKCILQIAL------------------LMCFSTTALS------MSYDVLRYQQ-RSSN

LACQKLLGQL-PGTPQYCLEDRMNFEPEEIM-------------QP-P----Q------F

QKED--AVLI-----I-H----EMLQQIFGILR-RNF-SSTGWNETVIKTILVELDGQMD

DLETIL--EEMEEE--------NFPRGDMTIL--------------H------LKKYYLS

ILQ-YLKSKEYRSCAWTVVQVEILRNFSFL-NRLTDY--LRN--------------

>Oror_6

MNHRCILQTAL------------------LLCFSTTALS------MSYRLLQFQQ-RSSN

LACQKLLQRL-PGMPQHCLEDRMDFKPEEIK-------------QP-Q----Q------F

RKED--AVLV-----T-Y----EMLQQIFGILR-RNF-SSTGWTETITENLLVEVYGQMD

RLETIL--EEMEKE--------NF-TSVVTIL--------------H------LKKYYLQ

IMQ-YLKSKEYSNCAWTVVRVEILRNFSFL-NRLTDY--LHN--------------

>Dano_11

MANRCAFQIAL------------------LLSFSTMALC------ISYNVLRFQQ-SSSN

LICQKLLKKL-NGSAEYCLQDRMDFKPEEIK-------------QP-Q----Q------F

QKEE--AALL-----I-Y----EMLQQIFGIFQ-RKF-SSTGWNETIVENLCVELYQQMD

RLETIL--EELEEE--------SFTWGDMTIL--------------H------LKNYYLR

ITQ-YLKAKEYSSCAWTVVRVEILRNFSFI-NRLTEY--LQN--------------

>Ptva_12

MTNRCILQFAL------------------LLCFSTTALS------MSYNWLRFQQ-RSSN

LACLKLLWQL-NGTPQYCHKDRMDFKPAEIK-------------QP-Q----Q------F

QKED--TVLI-----I-H----EMLRQIFDIFQ-RNF-SSTGWNETIIMNLYVTLSGQMD

RLETAM--EEMEEE--------NFTWESMTVL--------------H------LKNYYFR

IMR-YLETKLYSRCAWTVVKAEILRNFFFL-NGLTEY--LQN--------------

>Loaf_13

MTTRCILQVAL------------------LLSISTTALA------RSYKLLQFQQ-RSSN

LACQKLLWKL-NGAPESCLEDRMDFKPEEIK-------------QP-G----Q------L

QKED--AALV-----I-Y----EMLLQIFDIFL-GNF-SHTGWDETVIENLLAELSQQRD

RLVTIL--EEMEEE--------NPTSRNMTIL--------------H------LKNYYLG

IGQ-YLEAKDYSSCAWTVVQVEILRNFSFI-SGLTDY--LQN--------------

>IFNB_horse

MTYRWILPMAL------------------LLCFSTTALS------VNYDLLRSQL-RSSN

SACLMLLRQL-NGAPQRCPEDTMNFQPEEIE-------------QA-Q----Q------F

QKED--AALV-----I-Y----EMLQHTWRIFR-RNF-ASTGWNETIVKNLLVEVHLQMD

RLETNL--EEMEEE--------SSTWGNTTIL--------------R------LKKYYGR

ISQ-YLKAKKYSHCAWTVVQAEMLRNLAFL-NGLTDY--LQN--------------

>IFNB_cow

MTYRCLLQMVL------------------LLCFSTTALS------RSYSLLRFQQ-RQSL

KECQKLLGQL-PSTSQHCLEARMDFQPEEMK-------------QE-Q----Q------F

QKED--AILV-----M-Y----EVLQHIFGILT-RDF-SSTGWSETIIEDLLKELYWQMN

RLQPIQ--KEMQKQ--------NSTTEDTIVP--------------H------LGKYYFN

LMQ-YLESKEYDRCAWTVVQVQILTNVSFL-MRLTGY--VRD--------------

>IFNB_mouse

MNNRWILHAAF------------------LLCFSTTALS------INYKQLQLQE-RTNI

RKCQELLEQL-NGKIN--LTYRADFKPMEM---------------T-E----K------M

QKSY--TAFA-----I-Q----EMLQNVFLVFR-NNF-SSTGWNETIVVRLLDELHQQTV

FLKTVL--EEKQEE--------RLTWESSTAL--------------H------LKSYYWR

VQR-YLKLMKYNSYAWMVVRAEIFRNFLII-RRLTRN--FQN--------------

>Ereu_12

MANRYIFQIAL------------------LLCI-TTALA------ESYTLDQSQQ-KSSI

LVCQDLLNQL-NGSATDCLKQRMNSKPEEIK-------------NP-Q----L------L

QKED--LVLV-----T-Y----ELFQQIFGIFS-RNF-SRTSWNETIVEKLLMELYQQKN

QLKTTV--EEIKET--------NDIWGNKHIL--------------N------LKKYYFS

LMR-YLKANKYSSCAWIIIKTEIIRNFVYL-DKLISY--FSN--------------

>Modo_7

MVYRGILYLAL------------------LLLFSPSISS------KGYDSLRFHQ-RRTN

QRSLMFLNKMIGKLHPECLQERMDFQPREIV-------------QP-R----Q------C

QREN--ATMI-----I-H----EMLQQTLILFS-SKN-ACPDVNDTIIEPFLSGIYQQML

HLE----EEMDQAN--------SSWESLESIL--------------R------LKNYYQG

ITN-YLKNKEYSSCACKIVQVETRRNFSFL-YKLTEY--LKN--------------

>IFNK_human

MIQKCLWLEIL------------------MGIFIAGTLS------LDCNLLNVHL-RRVT

WQNLRHLSSMSNSFPVECLRENIAFEPQEFL-------------QY-T----Q------P

MKRD--IKKA-----F-Y----EMSLQAFNIFS-QHT-FKY-WKERHLKQIQIGLDQQAE

YLNQCLEEDKNEED--------MKEMKENEML--------------E------LRRYFHR

IDN-FLKEKKYSDCAWEIVRVEIRRCLYYF-YKFTAL--FRRK-------------

>IFNK_dog

VIRKCLWPACL------------------VGLLITGVLS------LDCNLLHFHL-RKVT

WQNLRLLSSMSNSFPVECLREIKAFEPQEIL-------------SH-T----Q------P

VKRY--IVEA-----F-Y----EMSIQAFNIFS-QYT-FKSTWENDYLKQIQIGLDQQLQ

YAERCLEEEEKEDD--------SKEMEEDGIL--------------E------LRRYFNR

IDN-FLKEKKYSHCAWEIIRVEIRRCFYYY-FKFAPL--LRKK-------------

>Ptva_13

MIRKCLWPACL------------------MGLLITGILS------LDCNLLN-----RVT

WQNLKLLSSMRNSFPKDCLRENKAFEPQEIL-------------YS-T----Q------L

LKRD--IKEA-----F-Y----EISLQAFDIFS-QYT-FQSTWKKKYLKRIQIGLDRQLQ

YLEQCLEEEEKNED--------MKEMEEDESL--------------E------LKRYFHR

ISS-FLKDKKYSHCAWEIVRVEIRRCFYY----FTAL--LRKK-------------

>Dano_13

MIQKCLWPACL------------------MDLFITGILS------LDCDLLNVHL-SRVT

WQNLRVLRSMSNSFPLKCLRETEAFEPQEIL-------------SN-T----Q------P

VRRD--IKEV-----F-Y----EMSTQAFNIFS-QYT-FNSTWEEKHLKQIQIGLDRQIE

YVEQCLEDEEKNED--------MKQMEEDEML--------------E------LRRYFNR

INK-FLKDKKHSHCAWEIVLVEIRRCFCY--FKFTAL--L----------------

>Oror_5

VIRKCMWPVCL------------------MGLFVTGILS------LDCNLLNVHL-RRVT

WKNLSLLRRMSKSFPIECLRESKAFEPQEIL-------------SH-T----Q------P

LTRD--IKEA-----F-Y----EMSRQAFHIFI-QDT-FKSTWEEKHLRQVQIGLDQQLQ

YLEQCLEEEE-NED--------MREVAEDERL--------------E------LRRYFNR

IDR-FLKDKKYSHCAWEIVRVEIRRCFYF--FKFTAL--LRRK-------------

>Loaf_16

VIRKYFWPICL------------------VGLFLTSVLS------QSCDLLYVHL-NRVT

WQNLKLLSHMSNPFPVECLKEKKAFEPQEIL-------------SH-T----Q------P

VKRH--IEEA-----F-Y----EISSQVFNIFS-QHA-CKSAWDEKHLKQIQIGLHQQVE

YLERCLEEEEKSED--------MKQMEE-KIL--------------K------LRRYFNR

LGN-FLKDKKYSQCAWEIVLVEIRRCVFYY-FKFTTL--LRKK-------------

>Ereu_11

LIRKCLWSSCL------------------VYLFLTGIHS------LDCSFLNIQL-RRVT

GQNARLMSSMKGPLRQECLKDINNFEPEEIF-------------LC-N----Q------S

KKWN--IKVN-----F-Y----EIYANAFRIFS-QYT-VKYSWEEECMQQILMELNLQLE

SLEQCLKEEK----------------ENDEIL--------------K------LKRYFFR

IQS-YLRDKKYSDCAWKIVFVEIGRCFYHS-LKLTRL--SRKK-------------

>Modo_1

-IQQCLIPAYL------------------LFLLVTSISS------LDCDSLYFYL-RNVS

LDGMILLRKMSGEFPLQCLKTRMDFESQELL-------------PP-N----Q------S

LT-D--VKIA-----I-Y----EMSQQIFNIFS-QNY-LPLTWEEENVQKIQIKLDHQSE

YLQRCLEEDEKKEV--------LEKEDEDEIL--------------E------LRKYFKR

IGS-FLKEKEYSHCAWEIVRLEMRRCFYT--LKLISQ--LK---------------

>IFNK_mouse

MTPKFLWLVAL------------------VALYIPPIQS------LNC----VYL-DDSI

LENVKLLGSTMTGFPLRCLKDITDFKPKEIL-------------PY-I----Q------H

MKRE--INAV-----S-Y----RISSLALTIFN-LKG-SIPPVTEEHWERIRSGLFKQVR

QAQECFMDEEKE----------------REHL--------------E------LGKYFFR

IKK-FLINKKYSFCAWKIVTVEIRRCFIIF-SKSRKL--LKMK-------------

>IFN1_anoli

-----LLPIAL------------------TMVLITEVSS------QDCGQLLARL-RQAN

KANLELLNSKMNSTPQQCIEGVFSFSLKNKL-------------TNRD----V------S

EEEN--AKVA-----I-Q----EVLQQTGHIFR-QNC-TEMLWDEDSLRAFHAGLDQQSE

NLKSCL----------------SASIQL-TSL--------------R------VKRYFRS

LND-FLKEKEYNRCAWEIIQIQVKQCFLWI-EKLIQE--IQSK---------MAH-

>Opha_2

-----FLQICL------------------VMF-FTNVSS------QHCDQLHSRL-QEDN

KGNLELLGSHMRATPLECIGDIADFS-EENV------------MSMNE----A------S

HEED--AKIA-----I-Q----EMLQQTDLIFK-KVH-AELFWDETSLRTFHTGLDQQIK

RLETCQ----------------NASLQL-TRL--------------R------VKRYFQG

LND-FLKDKQYSSCAWEIVQIQLRECFLLI-HQLIQR--IPTQ---------IKY-

>Vibe_2

------------------------------MF-FTKISS------QHCDQLHTRL-LEAN

KGNLELLGSHMRATPLQCIGDIVDFS-EEHL------------ISVDE----A------S

HEED--AKRA-----V-R----EMLQQTDLSFK-QAH-AELFWDENSLRQFHTGLDEQIK

KLETCQ----------------SASLQL-TRL--------------R------VKRYFQG

LNH-FLKEKKYSLCAWEIVQIQLRECFLLI-HQLIQR--IPIQ---------IMY-

>Pybi_2

-----LLQICL------------------AMF-FTKISS------QHCDQLHRRL-HKAS

KGNLKLLGSNIRATPLQCIGDIIDFS-EENL------------MSMDG----A------S

HEEN--AKIT-----I-Q----EMLQQIDLIFK-QVH-AELFWDENSLRQFHTGLYQQIK

ELEICQ----------------NASLQL-TRL--------------R------VKRYFQR

LSD-YLKDKKYSLCAWEIVQIQLRECFLLI-NELIQR--IPT-----------LY-

>Opha_6

-----CLFICL------------------GVF-FTEISP------QDCNQIRSRL-HEAN

LRNMNLPMRNMGSTPQQCIRDIIDFSLEENL------------TNMIN----E------L

QGET--AKVA-----I-K----ELLQQIDLIFK-ESH-SELAWDENSLREFHIGLHQEIK

NTKACW----------------NTSLQF-TRL--------------R------VKRYFQR

LRD-FLKNKEYNLCAWKIMQIQIRECFEWI-NHLNQR--IPSE---------T---

>Thsi_3

-----CLFICL------------------GI--FTEISS------QDCNQFRSRL-HEAN

LGNLNLLTRNMGSTPQQCIRDIIDFSLEENV------------MNMVN----E------L

QGEN--AKVA-----I-K----ELLQQIDLIFK-ESQ-SELAWDENSLREFHIGLDQEIK

KTAACW----------------NTSLKL-TRL--------------R------VKRYFQR

LRD-FLRNKDYNLCAWKIIQIQIRECFQWI-NQLNQR--IPNE---------T---

>Vibe_6

-----CLFICL------------------GIF-FTEISS------QDCNQLRSRL-HEAN

LGNLNLLTRNIGSTPQQCIRDIIDSSFEENL------------MNMVN----K------L

QGEN--AKVA-----I-K----ELLQQIDLIFK-ESH-SELVWDENSLREFHIELDQEIK

KAETCW----------------NTSLQF-TRL--------------R------VKRYFQR

LRH-FLKNKEYNLCAWKIIQIQVRECFEWI-NQLNQR--IPSE---------T---

>Opha_7

-----CLYICL------------------GIIFFGDISC------QNCNQLQRKL-LKAN

KDNSNLLSSNIRPTPLQCMRSFVELSLKKIM------------IDMND----E------C

QVDI--AKTA-----V-K----EILQQIDVIFR-QNH-TELVWHEGSLRDFHIGLDQQIK

MLETCG----------------NASLQL-TRL--------------R------IKRYFQR

LSD-FLKNKKYSLCAWEIVQIQIEACFQLI-NHYIQR--IRSK--------TMKK-

>Vibe_5

-----CLYICI------------------WIISFGGISS------QNCNQLQAKL-LKAN

QVNFNLLSSNIRPTPLQCMKDIINFSF-KIL------------IDMND----E------S

QVDI--AKTA-----V-K----EILQQIDIIFQ-QSR-TELVWHKVFLKDFRIGLDQQIK

MLETCE----------------NSSLQL-TRL--------------R------VKRYFQR

LHD-FLKNKNYNLCAWKIVQIQMKECFELI-NHYIQK--IPSK---------IVD-

>Thsi_5

-----CLYLCI------------------GII-FGEISC------ENCNQLQRKL-LKAN

QVNFKLLSSNIKSTPLQCYSDITDLSVEKIL------------IDMND----E------S

QVGI--AKTT-----I-E----EILQQIDLIFR-QNH-PKSIWHGDSLRDFHIGLDLQIK

MLETCG----------------NASLQL-PRL--------------R------VKRHFQK

VRD-FLKKKKYSLCAWKIVQIEMKQFLERI-NYYIQR--IPSK----------ID-

>IFN5_anoli

MAQQCLLAFCL------------------LMSF-REILS------QDCNDLRHEL-NGAN

KANLELLNVKMGSTPLQCVDDVINFSSKESL-------------PSIY----D------F

EEEN--ATVA-----I-D----EILQQISYLFN-QNH-TKLSWDENSIATFKLGVDNEIK

KLTPCL----------------SDSIDE-LRD--------------K------VRKYFER

INN-LLKEKEYNLCAWEIVQMEVRQCLIVV-DQLISR--IPKK---------KAV-

>Chmy_15

-----LPRVCL------------------VLLFFTEISS------RLCTMLHFQQ-KKMN

RESLEHL-KKMSGNPSQCINERAASKP-QDV-------------AQLP----V------S

QKEK--A--------------------IFSIFS-KNL-TQSAW---------NGLL----

------------------------------------------------------------

--------------------------------------------------------

>chpi_1

-------------------------------------------------MLHFQE-NKGN

KESVELL-KKMSENLSQFINEMKAFKP-QDV-------------VQLQ----L------S

QKEN--ADVA-----I-Q----EILQEIFTIFS-KNL-TQTAWDRSSIARFQNGLYQQIQ

PLEVCL----------------GA------------------------------------

--------------------------------------------------------

>Chpi_6

------------------------------ML-----------------MDVFQE-----

--------QTSSEKP-----------P-RDA-------------F-IP----V------I

KRAN--YE-W-----L-H----EILQQIFNIFS-KNL-TQSAWDGTSIVRFQNGLYQQIQ

RLEACL----------------RANLQL-TSR--------------R------VKKYFQG

IDA-FLKEKQYSLCAWEIIRMEISRSFVLI-DKLTRS--LSN--------------

>Almi_3

-----LLHICL------------------VLLFSTEISS------QHCDLLSFQQ-KKLN

KDSLELL-EKMGGNPFQCFSERTDFKS-QDV-------------LKLQ----L------S

QKEN--AKLA-----I-Q----NILQEIFTVFS-KNL-TQTAWDEISIITFQNKLHQQIE

RLEACL----------------GFKLVL-TKL--------------K------IKRYFQG

IYN-FLEEKQYSLCAWEIIRMEMTRGFLLV-DQLTKS--I----------------

>Gavga_4

-----LLHICL------------------ILLFSTEISS------QHCDLLSFQQ-KKLN

KDSLELL-EKMGGNPFQCFNEGIDFKS-QDV-------------LKLQ----S------S

QKEN--AKLA-----I-Q----NILQEIFTVFS-KNL-TQTAWDTISIITFQNKLHQQIE

RLEACL----------------GSKLVL-TKL--------------K------VKRFFQG

IYN-FLEEKQYSQCAWEIIHMEITRCFLFV-DQLTKS--L----------------

>Crpo_3

-MKMMLLHICL------------------ILLFSTEISS------QHCDLLSFQQ-KKLN

KDSLELL-EKMGGNPFQCFNEGIDFKS-QDV-------------LKRQ----S------S

QKEN--VKLA-----I-Q----NILQEIFTVFS-KNL-TQTAWDTISIITFQNKLHQQIE

RLEACL----------------GFKLVL-TKL--------------K------VRRYFQG

IYN-FLEEKQYSLCAWEIIRMEMTRCFLFV-DQLTKS--L----------------

>Gavga_8

FLSSFLLHVCL------------------VLLFSTEISS------LHCGLLNFQQ-KKLN

KDSLELL-DNMGGNPSQCSNERIDFKP-QDV-------------LMLR----S------S

LKEN--AKMA-----I-Q----EILQEVFTVFS-KNL-TQTAWDEASIVVFQNGLHWQTE

RLEACL----------------DLKALL-TRL--------------K------LKRYFQG

IRN-FLEGK-----------------------------------------------

>Crpo_4

FLSSFLLHVCL------------------VLLFSTEISS------LHCGLLNFQQ-KKLN

KDSLELL-DNMGGNPSQCSSERTDFKP-QDI-------------LMLR----S------S

LKEN--AKMA-----I-Q----EILQEVFTVFS-KNL-TQTAWDEASIVAFQNGLHWQTE

RLEACL----------------DLKVLL-TRL--------------K------LKRYFQG

IRN-FLEGKQYSLCAWEIIRLEMPRCFLLL-DLLTKW--LKI--------------

>Gavga_6

---------------------------------------------------------KIE

Q-SLELL-DKIGRKPSQ-SNEKIDFKP-QDV-------------LVLG----S------T

LKEN--AQMA-----I-Q----EALQGVFTVFG-KNL-MQTAWDETFIVMFQNGLHWQIK

KLEACL----------------GLKVLL-TRV--------------K------LKNYFQG

ICN-FLEGKQYSLCAWEIILLEMHRYFLLL-DQLTKW--LK---------------

>Chmy_2

-----LLHICL------------------VLLFSIEISS------LDCNMLHFQQ-NKMN

MESLELL-SKMGGQPLQCLNENRNFRF-QKA-------------LRPR----E------S

QEKN--AKVV-----I-Q----EILQQIFNIFS-KNL-TQAAWDRSSVETLQKGLHQQTE

QLETCL----------------YS-LLF-PML--------------K------LKKYFQR

IRD-FLKEKQYSLCAWETIRLEMGRCFFFV-DQLIIR--LQN--------------

>Chpi_17

-----LLHICL------------------IMLFSTEISS------LDCTILHFQQ-NKMN

MESLELL-SKMGGQPLQCLNENRNFRL-QKA-------------LRPR----E------S

QEKN--AKMV-----I-Q----EILQQIFNIFS-KNL-TQAAWDRSSVETLQNGLHQQTE

KLETCL----------------HLYLLF-PML--------------K------LKKYFQR

IRD-FLKEKQYSLCAWETIRLEMGRCLLFV-DQLIKR--L----------------

>Chmy_1

-----FLHICL------------------VLLFSTKTSS------VDSNMLHFQQ-NKVN

QASLQLL-EKMGGQPVQCLNENSNFIS-QNV-------------LSSR----E------F

QKES--VIVA-----L-Q----EILQQIFNIFS-KSQ-LQTAWDRSSMDAFQNGLHHQIE

LLKTWF----------------NEY--F-TIL--------------K------VKKYFHV

IDN-FLKEKQYSLCASEIIREEMRRCFLFI-DQLTKR--LKN--------------

>Chpi_2

-----FLHICL------------------VLLF-TENSS------VDCNMLHFQQ-NKVN

QASLQLL-EKMGGQPVQCLNENSNFIS-QNV-------------LSSR----E------F

QKEN--AMVA-----I-Q----EILQQIFNIFS-KSH-IQTAWDRSSIVAFQNGLHQQIE

LLKTWF----------------DGY--F-TRL--------------K------VKKYFHV

VDN-FLKEKQYSLCAWEIIREEMRKCFLIM-DQLTKR--LKN--------------

>Pesi_2

-----FLHICL------------------VLHISTKISF------VDCNMFLFQQ-NKVN

QDSLKLL-EKMGGQPVQCLNEKSNFIS-QNL-------------FSST----E------F

QKEN--AMMV-----I-Q----EILQQSFTIFR-KIQ-IQTDWDRSSIAAFQNGLYQQIE

LLKTWF----------------DGY--L-TRL--------------K------VKKYFHV

IDT-FLEKRQYSRCACEIIREEMRRCFLFI-DQLTKR--LKN--------------

>Chmy_7

----------------------------------------------------------MN

NENLEHL-EKMGGNPFQCLNEGTAFKP-RDI-------------LKLR----L------S

HQEN--AKVA-----I-Q----QTLQELFHIFN-NNL-TQAAWNGTSIKEFQNGLHQQIE

KLEMCL----------------SYYLLL-TGL--------------K------LKRYFQT

IQD-FPKEKQYNRCIQEIICVEISRCFLIL-NILTKR--LENE-------------

>Chpi_8

----------------------------------------------------------MN

SESLEHL-EKMGGNPFQCLNERTAFKP-RDI-------------LKIR----L------S

QQEN--AKVA-----I-Q----QILQELFHIFN-NNL-TQAAWNGTSIKEFQNGLHQQIE

KLETCL----------------SAYLLL-TSL--------------K------LKRYFQT

IDD-FLKEKQYSQCAWEIIRVEISRCFPIL-NILTKR--LQDE--E------LKY-

>Pesi_3

----------------------------------------------------------MN

SKSLEHL-EKMGGPPFQCLNERSAFKA-TDI-------------LKVR----L------A

QQEN--AKAA-----I-Q----QILQELFQIFS-KNL-THAAWDGTSIKEFQNGIHQQIE

KLEVCL----------------SAYLLH-TSL--------------K------LRRYFQT

VRH-FLKEKQYSRCAWEIIRLEVSRCFLVL-NILTKR--IEN--------------

>Chmy_4

-----LLQISL------------------VLLCTTKIST------LDCNTLPLLH-NKVI

QGNLHLL-NKMGQQPEQCQSEKMHFKF-EQF-------------LKLR------------

QKEN--AKVE-----I-Q----EILQQTFYVFT-KNL-TLAAWDGRALERFQNRLNQQIE

HLEACL----------------TEY--I-IRL--------------K------LKKYFQK

IDN-FLKDKQYSLCSWEIIRLEMRRCLQFI-DKVIRR--LRN--------------

>Chpi_3

-----LLQISL------------------VLLCTTKIST------LDCNILPLLH-NKVI

QGNLHLL-NKMGQQPEQCQSEKIHFKF-EQF-------------LKLR------------

QKKN--AKVE-----I-Q----EILQLIFYIFT-KNL-TLAAWDGRSLERFQNGLNQQIE

HLEACL----------------TEF--I-IRL--------------K------LKKYFQK

IDN-FLKDKQYSLCSWEIIRLEMRRCLQFI-DKVIGR--LRN--------------

>Pesi_1

-----LLQISF------------------MLLCTTNISA------LDCNILPLLH-NKMI

QGHLHVL-NKMGQQPEQCQSEKMHFQF-EKF-------------LKLR------------

KKEN--AKVA-----I-H----EILLQIFYIFT-KHL-TLVAWDGRSLERFQNGVNQQIE

HLDACL----------------TEY--I-IRL--------------K------LKKHFQK

LDN-FLKDKHYSLCSWEIIRLEIRRYLHFI-VKVTRR--LRN--------------

>Coli_2

-----LIQIGL------------------IVLCITIISS------HQCNHLPLQQ-RKAI

ENSLQLL-DKMGEKPQRCLREKMSFKF-KQV-------------LKPT------------

QKEA--VEVA-----I-E----EIFQHIFYIFS-KNL-TLAAWDGTALEKFQNGLYHQIE

QLEACV------------------Y--V-NRL--------------K------LKKYFQK

IDC-FLKDKQHNLCSWEISRAEMRRCLQLI-DKVIRK--LYKV-----------H-

>Apfo_3

-----LTQIGL------------------ILLCTTTISS------LQCNHLHLQQ-RKVI

ENSLQLL-DKMGEKPQQCLKEKMSFRF-EQV-------------LKPR------------

QKET--VKVV-----I-E----EIFQHIFYIFS-KNL-TLAAWDGTALEQFQNGLYQQIE

QLEACV------------------Y--V-NRL--------------K------LKKYFQK

IDC-FLKDKKHNLCSWEISRAEMRRCLQLI-DKVIRK--LNN--------------

>Fape_1

-----LIQIGL------------------ILLCTTTISS------LQCNHLPLQQ-GKVV

ENSLKLL-DKMGKKPQQCLREKMSFRF-EQV-------------LKPR------------

QKEN--VEVV-----V-E----EIFQHIFYIFS-KNL-TLAAWDGTALEQFQNGLHQQIE

QLEACV------------------Y--V-NRL--------------K------LKKYFQK

LDC-FLKDKKHDLCSWEISRAELRRCLQLI-DKVIRK--LNN--------------

>Tyal_2

-----LIQIGL------------------ILLCTTTISS------LQCSHLPLQQ-RKVI

KNSLQLL-DKMGKKPRQCLREKMSFRF-KQV-------------LNPR------------

QKET--VKVA-----I-E----EIFQNIFYIFS-KNL-TLAAWDGTALEQFQNGLYQQIE

QLEACV------------------Y--V-NRL--------------K------LKKYFQK

IDC-FLKDKQHNLCSWEISRAEMRRCLQLT-DKVIRK--LNN--------------

>Gaga_3

-----FIQIGF------------------ILLCTITISS------LTCNHLPLQQ-RRVI

ESSLQLL-DKMGRRPQQCLREKMSFRF-EQV-------------LKPR------------

QKET--VKVA-----I-E----EILQHIFYIFS-KNL-TLAAWDGAALEQFQNGLYQQIE

KLEACI------------------Y--V-NRL--------------K------LKKYFQK

IDS-FLKEKQHNLCSWEISRAEMRRCLQLI-DKVIRK--LYK--------------

>Stca_2

-----LLQIGL------------------ILSCTTNISS------LHCNHLSLQQ-SKVI

ESSLQLL-DKMGEKPQRCLRERMSFRF-EQV-------------LKPR------------

QKET--VKMA-----I-E----EILQHIFHIFS-KNL-TLAAWDGQALEQFQNGLYQQIE

QVEACV------------------Y--A-SRL--------------K------LKKYFQK

IDY-FLKDKQHSMCSWEISRAEMRRCLQFV-DKVIKR--LNN--------------

>Caan_3

-----LIPTGL------------------ILLCTTTISC------LWCNHLPLQQ-RKVI

QNSLQLL-DKMGNKPQQCLKEKMFFSF-EQV-------------LKPR------------

QKES--VKVA-----I-E----EIFQHIFYIFS-RNL-TLAAWDGAALEQFQNGLYQQIE

QLEVCV------------------S--V-NRL--------------K------LKKYFQK

IDC-FLRDKQHNLCSWEISRAEMRKCLQMI-DNVIWK--LNS--------------

>Almi_5

--------MFF------------------ILLCTMQIST------PDCNIPSLQQ-SKAI

QSSLHLL-DKIGQAPLQCRREHVLFKF-HNI-------------LKLS------------

QKDN--VKVA-----V-Q----ETLQSIFYMFS-KNL-TLAAWDGRSLESFQNGLYQQIE

QLEACS----------------IKY--A-NRL--------------K------LKKYFQR

IDN-FLKGKQYSLCSWEIIREEVRKCLQLI-EKGLEG--LENK---------IKND

>Gavga_2

--------MFF------------------ILLCTMQIST------LDCNIPSLQQ-SKAI

QSSLHLL-DKIGQAPLQCRREHVPFNF-RNI-------------LKLR------------

QKDN--VKVA-----V-Q----EMLQSIFYMFS-KNL-TLAAWDGRSLESFQNGLYQQIE

QLEACS----------------LKY--D-NRL--------------K------LKKYFQR

IDN-FLKDKQYSLCSWEIIREEVRTCLQLI-ETVTKA-------------------

>Crpo_7

MEVSGLLQMFF------------------ILLCTMQIST------LDCNIPPLQQ-SKAI

QSSLHLL-DKIGQAPLQCRHEHVPFNF-RNI-------------LKLR------------

QKDN--VKVA-----V-Q----EMLQSIFYTFS-KNL-TLAAWDGRSLESFQNGLYQQIE

KLEACS----------------LKY--D-NRL--------------K------LKKYFQR

IDN-FLKDKQYSLCSWEIIREEVRTCLQLI-ETVTKA-------------------

>Chmy_5

-------------------------------------MS------LDCNLLRHQQ-SKFN

WYSLQLL-QNMGGKPLECLEDKTAFQF-EKI-------------LKPK----F------L

QQ----AQMS-----V-H----EILEQLFGIFS-RNL-SQTGWERRKVERFLNGLALQTE

RLEECL----------------HT-------L--------------R------LKKYFQR

IQD-FLKEKKYSTCAWEIVREEGQRCFQYI-HKLTVR--MKN--------------

>Chpi_4

-----LWQICL------------------VLLFSAGVMS------LDCNLLRHQQ-SKFN

GYSLQLL-QNMGGNPLKCLEDKTAFQF-EKV-------------LKPK----F------Q

QH----AKMA-----I-H----EILQQLFGIFS-RNL-TQTGWERTKVGSFLNGLTLQTE

RLETCL----------------PT-------L--------------R------LKKYFQR

IQD-FLNEKKYSTCAWEIVREEGQRCFQYI-DKLTVR--MKK--------------

>Pesi_4

-----LWQLCQ------------------LLLFSAGVMS------LDCNLLHHQQ-SKFN

RYSLQLL-QKTGRSPLECLGDLTAFQF-EKV-------------LKHK----F------P

QH----AQMA-----A-H----EILQQLFGIFS-RNL-LQTRWEKGDVELFRNGLHLQTK

HLEKCL----------------ST-------L--------------R------LKRYFQR

IKD-FLEKKKYSTCAWETVRLEAQRCFLYM-DKLTVM--MKN--------------

>Almi_1

MMKNKLLHICL------------------VLLFSTEITS------MQCDMIHFQQ-KRLN

KDSLELL-EKMGGSPFQCSNEN--------------------------------------

--------------------------QIQDYPG-KNL-TQTTWDWSSIVTFQNGLHRQIQ

LLEACL----------------VATQLH-TRL--------------K------LNRYFQR

IHN-FLEEKHYNLCAWEIIRIEMPNCFLFV-DQLTKS--LKN--------------

>Almi_2

-----LLKFFL------------------VLLLFKVSSS------LHCNSLASNQ-NKVN

KDGLDFL-DKMRRNSPQCLSERLDLKT-KDI-------------FKIE----L------S

QKDN--AKAA-----I-Q----ELLKAIFYVLS-NNL-TQTTWQESSIEKFKNGLHWQIE

NLETCL----------------DASPLV-TRL--------------K------LKRYFQA

IDN-FLKEKQYSQCAWEIISVELSRCFQFI-DKLTIK--LSTS-------------

>Crpo_5

-----LLKFFL------------------VLLFFKVSSS------LHCSSLASNQ-NKVN

KDGLDFL-DKMRRNSPQCLSERLDLKT-KDI-------------FKIE----P------S

QKHN--AKAA-----I-Q----ELLKAIFYVFS-KNV-TQMTWQESSIEKFKNGLHWQIE

NLETCL----------------DASPLV-TRL--------------K------LKRYFQA

IDN-FLKEKQYSQCAWVIISVELSRCFQFI-DKLTKK--L----------------

>Gavga_7

-------------------------------------SS------LHCSSLASNQ-NKVN

KDGLDFL-DKMRRNSPQCLSERLDLKT-KDI-------------FKIE----L------S

QKHN--AKAA-----I-Q----ELLKAIFYVFS-KNL-TQTTWQESSIEKFKNGLHWQIE

NLETCL----------------DAGPLV-TRL--------------K------LKRYFQA

IDN-FLKEKQYSQCAWEIISVELSRCFQFI-DKLTKK--LRYC-------------

>Chmy_6

------------------------------------------------------------

----------------------------MAI-------------LKPR------------

EKVN--IVVT-----I-H----KILHETFNLFS-KNL--HAAWNTTCIEKFQNGLHWQIE

QLETCL----------------GANLQS-T-L--------------N------VKKYFQR

IKD-FLKEKHYSHCSWEQY-----FCLLSF-KKKSKN-------------------

>Almi_4

MEEPTFLHVCL------------------VLVFSIKISS------PDCS--RLQR-IKVN

-HSLYLL-CRMGGQPLSCLNDRTDFRI-REI-------------FIIR------------

KKEN--ALMI-----I-H----ELLHHIFQLFS-KNL-PQGAWNPSCIEKFQNGLHWQIE

QLEKCF----------------GGNLQN-NIL--------------K------AKKYFQR

ISH-FLNEKNYSRCSWETARMEMRRCFLFL-DHLLKN--LRN--------------

>Gavga_3

MEEPAFLHVCL------------------VLVFSIKISS------PDCS--RLRQ-IKVN

-QSLHLL-CRMGGEPLSCLNDRPHFRI-RQI-------------FTAR------------

NKEN--ALMI-----I-H----ELLHHIFQLFS-KNL-PQGLWNPSCIEKFQNGLHWQIE

QLQTCF----------------GGDLQN-NIL--------------K------VKKYFQR

ISH-FLNEKNYSRCSWETARMEMRICFLFL-DHLLKK--FRN--------------

>Crpo_1

MEEPTFLHVCL------------------VLVFSIKISS------PDCS--RLQQ-IKVN

-QSLHLL-CRMGGEPLSCLNDRTDFRI-REI-------------FTAR------------

KKED--ALMI-----I-H----ELLHHIFQLFS-KNL-PQGPWNPSCIEKFQNGLHWQIE

QLETCF----------------GGDLQN-NIL--------------K------VKKYFQR

ISH-FLNEKNYSRCSWETARMEMRRCFLFL-DHLLKK--FRK--------------

>Coli_1

-----LLQVCI------------------TVALYIKISH------PVC---LFQG-IKMN

YHNMNIL-CTIGGYSQQCLSETTDFRF-MEI-------------TKVT------------

QK-D--VTMI-----I-Y----EFLQQIFQLFS-KNL-PVGAWNTSKIEKFQNGLHQQIE

ELEICL----------------SEILKS-TTF--------------S------VKKYFQR

ITS-FLKDKQYSHCSWEAVQMELRTCLIIF-DSLLKK--QAT--------------

>Fape_3

-----LLQVCV------------------TLALYIQISH------PVC---LFQG-FKMN

YHNINFL-CKMGGCSQQCLSETTDFRF-MEI-------------TNIT------------

QK-N--VKMI-----I-Y----EFLQQIFQLFS-KNL-PVGAWNISKTEIFQNGIHQQIE

ELEICL----------------SETLKS-TTF--------------S------VKKYFQR

ITN-FLKDKQYSHCSWEAVQMELRTSLIIF-DSLMKN--HTS--------------

>Tyal_1

TDKTTLLRVCV------------------TLALYIKISH------PVC---LFQG-IKVN

YNNMNFL-WTMGGYSQQCLSEATDFRF-MEI-------------TKVT------------

QK-N--VTMI-----I-Y----EFLQQTFQLFS-KNL-PAGAWNTSKIQKFQNGIHQQIE

ELEVCL----------------LEILKS-TTF--------------S------VKKYFQR

ITD-FLKDKKYSHCSWEAVQMELRSCLIIF-DSLLKK--HTS--------------

>Apfo_2

-----LLRVCI------------------TLALYVKISH------PAC---LFQG-IRMN

YHNMNLL-CKMGGYSQQCPSETTDFRF-MEI-------------TKIT------------

QK-N--VTVI-----T-C----KFLQQIFQLFS-KNL-PVGAWNTSNIEKFQNGIHHQIE

ELETCL----------------SESLRS-TTL--------------S------MKKYFQR

ITN-FLKDKQXSHCSWEAVRMELRTCFIIF-DI-----------------------

>Gaga_4

-----LLRVCI------------------TLALYIKISH------HIC---LFQG-IKMN

YQNMNFL-HKMGGYSQQCLNETTDFRF-REI-------------TKIT------------

QK-N--TTVI-----I-Y----EFLQQTFQLLS-KNL-PKGVWNTSNIEKFQNRIHQQIE

ELQICL----------------LESLKS-TTL--------------S------MKKYFQR

ITS-FLNDRQYRHCSWDTVQTELRSCLIIF-DSVMKT--TH---------------

>Stca_3

-----LLQVCI------------------TLALYTKISH------PVC---LFQG-SKVN

YQNMNFL-CKMGASPQQCLRERTDFKF-MEI-------------TKVR------------

QR-N--AIVM-----I-H----ELLRQIFHLFS-KNL-PESVWNASCIEKFQNGIHQQIE

ELETCL----------------VESLNS-TTL--------------R------VKKYFRR

ITS-FLENKQYSHCSWEAVRMEVRTCFIFI-DCLMRK--HMA--------------

>Caan_2

-----LLPVCI------------------TLALYIKILH------TVC---LFEG-IKAN

HFNMNSL-FKIGGYSQQCLHETTDFRF-KEV-------------TKVT------------

QN-N--MTII-----I-Y----EFLQQTFQLFS-KNL-PVGTWNKSQTEKLKNSIHQQIE

KIEICL----------------SESLTS-TTF--------------S------MKKYFRR

ITN-FLKDKQYSHCSWEAVQMELRTLSLIQ-EQRGKK--TL---------------

>Almi_11

----------L------------------VNLYHAR---------------KQER-MKIN

K-------EKRSPHDISRKYKKIDFKP-QDV-------------LILH----S------T

LKEN--AKMA-----I-Q----EILQGVFTVFS-KNL-MQTAWDETSIVMFQNGLHWQIK

RLEACS----------------GWEVLL-TRL--------------K------LKSYFQG

I-------------------------------------------------------

>Almi_7

-----LWKICL------------------VALLSAHVAA------LDCSNFRDLQ-KVLN

RNSMQLL-GQVAGAPEECLEDRPTFRF-DKV-------------LRSK------------

APHN--AWMA-----T-Y----EILQKLFSLFK-RTL-PETAWDTRSVERLLNAVHLQIK

RLETCP------------------Y----KAT--------------G------LKKYFRK

IDD-FLRAKNYSKCAWEVVRIEAKTWFYYL-DKLKNR--LN---------------

>Gavga_1

-----LWQICL------------------AALLLAHVTA------LDCSNLKDLQ-EVLN

RNSVQLL-GQVAGAPEECLEDRPTFRF-EKV-------------LRSK------------

APHN--AWMA-----T-Y----EILQQLFSLFK-RNL-PETAWDTRSMERFLNAVHVQIK

RLETCP------------------Y----KAM--------------K------LKKYFRN

IHD-FLQEKNYSKCAWEVVRIEAKTWFYYL-DKFRNR--L----------------

>Crpo_2

-----LWQICL------------------AALLLARVTA------LDCSNFKDLQ-EVLN

RNSMQLL-GQVAGAPEECLEDRPTFRF-EKV-------------LRSK------------

APHN--AWMA-----S-Y----EILQQLFSLFK-GNL-PETAWDMHSVERFLNAVHVQIK

RLETCL------------------Y----KAM--------------K------LKKYFRN

IHN-FLREKNYSKCAWEVVRIEAKTWFYYL-DKFGNR--L----------------

>Chmy_14

-----MAYQVP------------------TQP---------------------GM-TSIS

KSAFSAL-QK----PSSC------------------------------------------

------SK-----------------------------------NGSSIKVFQNGLHQQIE

KLETCL----------------SACLLL-TSL--------------K------LKRYFQT

IDD-FLKEKQYSRCAG---RSSVRKYPDVF-SFLTY--------------------

>Chpi_15

-----LADESP------------------VTVLSVH---------------ASGQ-SSSG

RRLLAPL-MPLRSSPGSAW-----------------------------------------

RHQP--SKI---------------LQERFHIFN--NL-TQAPWNGTSIKEFQNGLHQQIE

KLETCL----------------SAYLLL-TSL--------------K------LKRYFQT

IDD-FLKEKQYSLYAWEIIRAEISRCLLIL-DIL----------------------

>Opha_5

-----TLKVCL------------------VLVLFAKSSV------FRCSIFHAQW-KQLI

QNNLPHL-CRTNEEPLQCIYELTDFNF-LEV-------------LEVT------------

NRDS--ADI------I-Y----ELLQQISYLLS-NA----HAWNSTCFENLKNGLHQQIK

NLETCL----------------NVNSYL-LTL--------------K------VKRYFQR

MNN-FLTVKQHSSCSWEMIHSEIKGCMLFI-THLLKK--L----------------

>Thsi_2

-----ILKVCL------------------VLVLLAKFLV------FRCSIFHAQW-KQLI

QNNLPHL-CRTNEEPLQCIYELTDFGF-LEV-------------LEVS------------

NRDN--AVI------I-Y----ELLQQISCLLS-NAP-SDHAWNSTCFENLKNALYEQMK

NLQACL----------------NAKSYL-LTL--------------K------VKRYFQR

MNN-FLTL------------------------------------------------

>Pybi_1

-----TLKICL------------------VLVLFAKLSV------FQCSIFHAQW-KQMI

QNNLHQL-CRANEEPLKCLPEVTDFRF-LEV-------------LKVS------------

NRGN--AII------I-Y----EILQQISCLLS-KGH-SYNVWNSTCFENLQNALHQQMK

TLATCL----------------NATSYF-LTL--------------K------VKKYFQR

MND-FLTVKQYSSCSWEMIHSEIKGCMLFI-IHLLNK--LKQ--------------

>Vibe_8

-----ILKFCL------------------VLVLFAKFSV------FRCSVFQAQW-MQLT

QNNLPHL-CR-NEIPPQCIYDLTDFRF-VEL-------------LQVN------------

NRDN--AVI------I-R----EFLNQIFYLLS-YAH-RYKVWNSTCFENVKIVLHQVIN

NLERCL----------------NANSYL-PKI--------------K------VKRYFKR

MIN-FLTLKQHSSCSWKIIHLEIEKCMMFI-TRLLEK--LKQ--------------

>IFN1_ONMY

--------------MYTMQ--SW-SC---IFL----IICSMQSVCHCCDWIRHHY-GHLS

AEYLPLLDQMGG----DITKQ----------N--AP----VLFPTSLYRHIDD------A

EFED--KVIF-----L-K----ETIYQITKLFDG--NMKSATWDKKNLDDFLNILERQLE

NLNSCV---------------------S-PAMKP------ERRLKR----------YFKK

LSK-VLRKMNYSAQAWELIGKETKRNLQRL-DILAAQ--MY---------------

>IFNA1_SASA

--------------MYTVQ--SW-TC---ICL----IICSMQSVCHCCDWIRHHY-GHLS

SEYLSLLDQMGG----DITKQ----------D--AP----VFFPTSLYRHIDD------A

EVED--QVRF-----L-K----ETIYQITKLFDG--NMKSVTWDKKKLDDFLNILERQLE

NLKSCV---------------------S-PAMKP------EKRLKR----------YFKK

LKN-VLRKMNYSAQAWELIRKETKRHLQRL-DILAAQ--MY---------------

>Eslu_3

-----------------MQ--SW-IF---LFL----ILCRTQSSCSCCDWIRDHY-GTLS

REYLSLLDEMGG----NITKQ----------D--VP----VFFPESLYRLMED------A

QYEV--QVRF-----L-N----ETIHEIIKLFDE--NMDAVTWEEKKLDDFLILLHRQFQ

KLKSCV---------------------S-PAKKA------EGRLES----------FFKK

LKK-VLKEMNYSAQAWELIRKETKYVLEKL-YLLVAT--MHR--------------

>IFN1_DARE

---------------------MW-TY---IFV----IYVILQSQSSACEWLG-RY-RIIT

TESLNLLKNMGG----KYA-D-------------LE----TPFPSRLYTLMDK------S

KVED--QVKF-----L-V----LTLDHIIHLMDAREHMNSVNWDQNTVEDFLNILHRKSS

DLKECV---------------------A-RYAKPAHKESYEIRIKR----------HFRT

LKK-ILKKKQYSAEAWEQIRRVVKSHLQRM-DIIASN--ARVN-------P-----

>IFN_CAAU

---------------MKTQ--MW-TY---MFV----MFLTLQGQCSACEWLG-RY-RMIS

NESLSLLKEMGG----KYP-E----------G--TK----VSFPGRLYNMIDN------A

KVED--QVKF-----L-V----LTLDHIIRLMDAREHMNSVQWNLQTVEHFLTVLNRQSS

DLKECV---------------------A-RY-QPSHKESYEKKINR----------HFKI

LKK-NLKKKEYSAQAWEQIRRAVKHHLQRM-DIIASN--RR---------------

>IFN_CTID

---------------MKTQ--MW-TY---MFV----MFLTLQGQCSACEWLG-RY-RMIS

NESLSLLKEMGG----KYP-E----------G--TK----VSFPGRLYNMIDN------A

KVED--QVKF-----L-V----LTLDHIIRLMDAREHMNSVQWNLQTVEHFLTVLNRQSS

DLKECV---------------------A-RY-QPSHKESYEKKINR----------HFKI

LKK-NLKKKEYSAQAWEQIRRAVKHHLQRM-DIIASN--RR---------------

>IFN_CYCA

--------------MNQTQ--MW-TC---IFV----IFLTLQSQCSACRWLG-RY-GTVS

ADSLNLLREMSG----QYP-E----------N--VK----MHFPGTLYNLIDK------A

EVED--QVRF-----L-V----LTLDHIINLMDASEHMNSAKWNLKKVEYFLEDLQRQSS

ELKECV---------------------A-QYQKPLQKESYEIRIKR----------HFRT

LKK-ILKKEKYSAQAWEQIRRAVRSHLQRM-DIIANN--AKKR-------V-----

>IFN2_ICPU

--------------MDIKL--SW-IC---LFL----LFFTVQERSEACNWMISQY-RAKN

DYCLSLLNEMGG----EIVPM----------T--GN----TSFPRRAYHEIEK------A

QAED--QVRF-----L-A----VATNEIIILFSAVSHVDDVKWDSRTLDNFLNILSRQLS

ELRNCT---------------------S-TYAERARRSSTEKKLRK----------HFKD

LRK-YLKNSNYSADSLEQIRSVVQRHLWRM-DTIAAI--VKQK-------LLRTN-

>IFN4_ICPU

--------------MDIKQ--SW-IC---LFL----LFFIVQERSEACNWMISQY-RAKN

NFCVSLLKEMGG----EIVQV--------------N----RPFPHKAYSEIDK------A

KAED--QVRF-----L-A----QATEQIISVFN-VSHVDEVKWDRSALDEFLNILNRQLT

ELTKCT---------------------S-TYAERAGHSPTERKLRK----------HFKK

LKK-FLNEANYSADSLERIRNVVQHHLWRM-DIIAAN--VKQK-------LLRTN-

>Anja_5

-------------------------------F----IVCSAQDFCDGCYWIQHGF-RRVS

GESLSLLSE---------------------------------------------------

MVDD--KVKF-----V-H----ASIDQIIKLFDE--NLDAVTWNRLKLEHFLIVLDRQSR

ELQKCV---------------------S-RCSII------RK------------------

--------------------------------------------------------

>IFND1_ONMY

----------MHR-T--KS--LL-IC---LFL----TLCN-G-LSVGCRWMDHKF-IQHS

ETLMNVLNIMGG----EFTTD----------S--VD----VPFPEDLYEQAEY------L

PTDD--TIWF-----I-L----QTLDKIAELFD-GEL-NSV-WDEKKVEIFLNVLTSQSD

GLQSCV---------------------R-AQKKN----------SKN------LQMYFKR

LNNHVLKRMAYSAHAWELVRKEVRTHLRRL-VLLGSA--TENR-------I-----

>IFND1_SASA

----------MHR-T--KS--LL-IC---LFL----TMCD-G-FSMGCRWMDHKF-IQHS

ETLMNLLNIMGG----EFTTD----------S--VD----VPFPEDLYKQAEY------L

PTDD--TIWF-----I-L----QTLDKIAELFD-GEL-DSV-WNEKKVEIFLSVLNSQSD

GLQSCV---------------------T-AQKKN----------SKN------LQMYFKR

LHNQVLKRMAYSAHAWELVRKEVRTHLMRL-VLLGSA--TENS-------I-----

>Eslu_2

--------------------------------------------------M-------HS

ASSLGFLDIMGE----DIPKD----------S--VK----IFFPEDLYKQADC------S

PADD--QIWF-----I-L----QTLDEITKLFS-DKC-YSV-WGEKTVDNFLGVLSSQVD

GLQSCI---------------------T-SQKKR----------SKN------LHKYFKR

LNNDILKSMEYSPHAWEMVRKEVRTHLKRL-TLLGSA--PDNK-------LVQQ--

>IFN1_GAAC

--------------M--LCRMFS-VF---VCL----SLYSSA-SSLSCRWVDHKF-SHLS

RTSMDLLDTLAH----NSTNS----------TEDSENN----FPNNLYSQASK------A

SAED--KLRF-----S-V----QILEEMAALFE-EDH-SNASWEEQTVDHFLIVVTKQAD

SLHSCS---------------------H-GHKRK----------NKK------LQMYFKR

LSHRVLEQMGHSAESWELIRKEMKAHLMKT-DQLVLSLL-SN--------------

>IFN_SPAU

--------------M--LNRIFF-VC---LSL----SLYSAG-SSLSCRWMDHKF-RQHS

KNSLALLDTMAN----NSTNT----------TEDAEVEDTVAFPNLLYRQASK------A

SAED--QLAF-----T-V----QILDETAALFE-EDH-SSASWEENTVENFVNVVTQQAD

ELRSCI---------------------G-SHKKK----------NKK------LHMYFQR

LSSHVLKRMGHSAEAWELIRGEVKVHLMRA-NQLVTSATRTN--------------

>IFN_DILA

--------------M--LNRIFF-VC---LSL----SLYSAG-SSLSCRWMDHKF-RQHS

KNSLALLDTMAN----NSTNT----------TEDAEVEDTVAFPNLLYRQASK------A

SAED--QLAF-----T-V----QILDETAALFE-EDH-SSASWEENTVEDFVNVVTQQAD

NLRSCI---------------------G-SHKT-----------NKK------LQMYFMK

LSSHVLKKMGHSAEAWELIRKEIKTHLMRA-DQLVSSLLTTN--------------

>IFN1_OPFA

--------------M--LSRTFL-VC---LFL----SLCSAG-SSLSCRWLDHKF-RQHS

ENSLDLLDTMVN----NSTNT----------TEDAGVKDTVAFPNELYSQASK------A

AAED--KLGF-----T-V----QVLEETAALFE-EDH-SSASWEENTVENFVNVVTQQAD

GLRSCI---------------------G-SHKKK----------NKK------LHMYFKR

LSSHVLEQMGHSAEAWELIRNEIQTHLMRA-DLLVSSLLTTN--------------

>IFN_EPCO

--------------M--LSRILL-VL---LSL----SLYSVG-SSLSCRWVDHKF-RQHS

EESLALLDTMAS----NSTNS----------TEDAEVDDTVSFPNHLYSQASK------A

SAED--KVAF-----T-V----QILEEMVALLE-GGY-SSASWEENTEENFLSVVSRQAV

GLRSCT---------------------V-HHK-E----------SKK------LHMYFKR

LSSHVLEQMGHSAEAWELIRREMKSHLKRV-DQL----LLSN--------------

>IFN_HYSE

--------------M--LSRILL-VL---LSL----SLYSAG-SSLSCRWVDHKF-RQHS

EDSLALLDTMAN----NSTNT----------TEDAEVDDTVSFPNRLYSQASK------A

SAED--KVAF-----T-V----QILEEMVALLE-GGY-SSASWEENTEENFLSVVSRQAV

GLRSCI---------------------G-SHR-E----------SKR------LHMYFKR

LSSHVLEQMGHSAEAWELIRREMKTHLKRV-DQL----LLSN--------------

>IFN_PAOL

--------------M--LNRIFF-VC---LCL----CLYSAG-SAMSCRWMDHKF-RQYS

KNSLDLLDMMAH----NSINT----------TEDVEVEETVALPEHLYSQVSN------A

SAED--RLGF-----T-V----QVLKEVAALFE-EDS-SFASWEEKQMDDFLNIVTQQAD

GLRSCI---------------------V-SHSHK----------NKK------LHMYFKR

LSRHVLKQMDYSVESWELIRKEIKNHLMRS-DVLISSLLTIN--------------

>IFN2_ORNI

--------------M--MNRILF-AC---LFL----GLFTVG-SSLSCKWMDDKF-KQHN

EETLNLLDTMGN----NSTNT----------T---EVEDTVAFPNHLYRQASK------A

SAED--KLAF-----T-V----QVLEEVAALFE-EDH-SSASWEDSTVRNFLNIVNKQAE

ELHSCI---------------------G-SHSHK----------KKK------TEMYFKR

LSDDVLKKKGHSAEAWEVIRKETKAHLMRL--TLIKSRGTTQS-------L-----

>IFN_ORLA

--------------M--LHRLVF-AC---ALV----SLAGAG-FSLRCRWLDHKF-KQFS

DTSLDLLEKMVN----NATNS----------TEGDATEDIVDFPHHLYRQASK------E

SAEN--QVAF-----T-V----QVLKEVSALFE-EDS-SSASWQQITVEKFLGVVNRQAD

ELHSCV-----------------------SESKK----------NRK------LRMYFKR

LLDHILKKQGYSAEAWETIRKETKAHLLRA-QRLLSPLISSK--------------

>IFN1_ORNI

--------------M--ISRIFI-AC---LFL----GMYSTG-SSLSCKWI-----VKHP

GNTLALLCIMFN----VITNT----------TKDAEIEHNVAFPNRLYRQTSK------A

TAED--KLAF-----T-V----QILKELLALFE-EDH-SSASWEENTVENFLNIVDKQTE

ELHSCI---------------------G-SHSNT----------QKR------REKYFKR

LLNKILKKNGYSAEAWEKIRNITQAHLRQC--EFLISLRTAH--------------

>IFN_TARU

--------------M--LP--LL-VC---LSL----CVYSQG-SPLGCRWLDDKF-RQYS

HKSLELLDTMVN----NSTNS----------S--VEPEEMVIFPQELYRQTFN------A

SAED--KLAL-----A-A----QIMNETVALLM-EDH-SGASWDEKQVENLVNVLTQQAD

NLQACM---------------------V-SPGHK----------SEE------VERYFNR

LSNHILKKMDYSAAAWELIREEIETLLMQT-HLLVSTLLSTP--------------

>IFN_TENI

--------------M--LT--VL-LC---LSL----CVCSQG-SPLGCRWLEEKF-TQYS

SLSLSLLDNMKS----NSTNS----------S--LEAEDTAIFPEVLYRQTFN------A

SAED--RLAF-----A-A----QILNETAALFE-EDY-SGASWEEKSVENFVNILTQQAD

NLGSCV---------------------A-SPGQS----------SKE------LHKYFTR

ISTHILRKTDHSAGAWELVREKIRSLLMRA-HLL----LTTH--------------

>IFN4_DARE

--------------M--KV--FA-AA---QFC----VLLSVG-FSLGCRWVKHRL-QHHH

GVSLDLLRKMGE----KVHDD----------N-----EDLNPIPYDLINNHRM------A

EPEK--QIQF-----V-I----QALVEITALFD-DAL---VPWDAKKMDDFLNIMHEEID

GLRSCG---------------------S-YKMKR----------NKK------LHLYFNR

-----LRRMTDGGRSWEMVRKRVIS-LMNQ-LHSFSF--HTH--------V-----

>IFNa1_gaac

--------------M--TSWTSM-LV---LLT----LLCSAGTPGLCCDWL-QHY-GHLS

NVSLTLVQTMGN----QLTDE----------E--SP----VSFPYRLYERIMN------D

KEDN--QLVF-----I-R----DSLELMAKLYR-HDNRSSVTWDANKMERFLMIIHRQIH

GLNLCV---------------------S-T--Q----------ITRR------LRRYYRR

LEKKTLYSTGGSPASWELIRKESKLHLDQL-NQLWGFMV-----------------

>IFNE1_ONMY

--------------MGSIS--FW-MC---LVM----TICTWN-KTIGCTWMKSMF-QVFS

NNTITMLQKMGH----EVSRG-------------PQ----ITFPDKQYRQVNN------F

KADE--QIAF-----I-S----HTLNAIKKLYSSGKY-ESTAWDQKGVDKFMNDLYRQTS

ELDQCV---------------------K---AMKTRLSKSVNRVNKK------MSLHFKF

LKH-FLKREDYSASGWEDIRTVVLAHLQRL-DTTLSS--K----------------

>Eslu_4

--------------MYSIR--LC-MS---LVL----MICSCN-ETMGCTWMRSMF-KSFI

SKSITVLQEKD-------DGE-------------PL----ISLPNKLYRQFDD------L

KADD--QIVF-----I-S----RTLKAIMHLYSSGKY-ES-TLETERIDTFIHYLSRQTM

ELDQCI---------------------K---AMNPTLSKSVKRANKK------MNSHFKF

LKN-YLKGEEFNGKAWIEIKRVVLAHLRRI-V--LT--------------------

>Opha_1

MISGCFQHIVL------------------FLLLSSGIRS------LDCNHI-VKQ-QSRT

VNIMKLLESMG---PWQCFNKIQDFAP-NDTG---------------------------S

IKED--ARAT-----F-G----LMLEQINRMFW-QNF-TKAEWNVTVTEHLQTSLDQQLV

QWEKCV----AEGK------KATK---DRIKL--------------K------LRKYFLR

LDT-FLKDEEYSSCAWEAVRHEIMGIQVFL-DQLLRT--LQR--------------

>Thsi_6

MISGCFQYIVL------------------LLLLSSGIRS------LNCNHI-VKQ-KDET

MNTIKLLESMG---PWQCFNQIQDFAP-NDTG---------------------------S

IKED--ARAT-----V-G----LMLEQINRIFS-QNF-TKAEWNMTITEHFQISLDQQLV

QWEKCV----AEGK------KATK---DRTKL--------------K------LRKYFLR

LDR-FLKDEEYSSCAWEAVRHEIMGIQFFL-DQLLRT--LQH--------------

>Vibe_7

MISGCFQYVVL------------------LFLLSSGVRS------LDCNHI-VKQ-KGAT

VNIMKLLEAMG---PLECFHKIPDFAP-NNIG---------------------------S

IKED--AKAT-----I-G----LMLEQISRIFS-QNF-TQTEWNMTVAEHFQIALDQQIV

QWEKCV----TEGN------KATK---ERTKL--------------K------LRSYFLR

LDT-FLKDEEYSSCAWEAVRQEIKGIPLFL-DRLLRT--LQS--------------

>Pybi_3

MVLRCLQHIVL------------------LLLFSSGIVS------LDCNHI-VKQ-KGAT

ADIRKLLEAMG---SLECFHKIPDFNP-KNIG---------------------------S

IKED--ARAT-----V-G----LMLEHIQRIFW-HNF-TKAEWNMTVTELFQIVLDQQVV

QWETCV----TVGE------KATK---DRTKL--------------K------LKKYFLR

LDT-FLKDEEYSPCAWDVVRQEVLGIHFFL-DQLLRT--LQN--------------

>IFNA_chick

--------MAL------------------TLLLKALATA------SACNHLRPQD-ATFS

HDSLQLLRDMAPTLPQLCPQHNASFNDT--I-------------LDTS----N------T

RQAD--KTT--------H----DILQHLFKILS-SPS-TPAHWNDSQRQSLLNRIHRYTQ

HLEQCL----------------DSSDTR-SRL--------------T------IKKHFSC

LHT-F-QDNDYSACAWEHVRLQARAWFLHI-HNLTGN--TR---------------

>IFNA_turke

--------MAL------------------TLLMKALAAA------AACNHLRPQD-ATFS

RDSLQLLRDMAPSPPQPCPQHNASFNDT--V-------------LDTN----N------T

QQAD--KTT--------H----NILQHLFKILS-GPT-TPAHWIDSQRQSLLNQIQRYAQ

HLEQCL----------------ADSHTR-SRL--------------T------INKHFSC

LHA-FLHDNDYSACAWDHVRLRARAWLLHI-HDLVRN--TR---------------

>IFNA_goose

--------MPL------------------LLLLTPPADA------FSCSPLRLHD-SAFP

WDSLQLLCNMAPSPTQPCPQQHASFPDT--L-------------LDTN----D------T

QQAS--HAT--------L----HLLQHLFDTLS-SPS-TPAHWLHTARHDLLNQLQHHIH

HLERCF----------------PADATR-FHL--------------G------INKYFGC

IQH-FLQNHTYSPCAWDHVRLEAHACFQRI-HRLTRT--MR---------------

>IFNA_duck

--------MPL------------------LLLLTPPANA------FSCSPLRLHD-SAFA

WDSLQLLRNMAPSPTQPCPQQHASFPDT--L-------------LDTN----D------T

QQAA--HTA--------L----HLLQHLFDTLS-SPS-TPAHWLHTARHDLLNQLQHHIH

HLERCF----------------PADAAR-LHL--------------S------INKYFGC

IQH-FLQNHTYSPCAWDHVRLEAHACFQRI-HRLTRT--MR---------------

>Gaga_2

--MTAHS-ILL------------------LLLLPALTTT------FSCNHLRHQD-ANFS

WKSLQLLQNTAPPPPQPCPQQDVPFPET--L-------------LKSK----D------K

KQAA--ITT--------L----RILQHLFNMLS-SPH-TPKHWIDRTRHSLLNQIQHYIH

HLEQCF----------------VNQGTR-SQL--------------S------INKYFRS

IHN-FLQHNNYSACTWDHVRLQARDCFRHV-DTLIQW--MKSR------PLARLN-

>Caan_1

MAAPGHGAPML------------------LLFFTPLAIA------TMCHHLQPRE-DTFS

WDSLQLLQDMAPSPPHPCQQYQMLFPQA--F-------------LHTK----H------P

RHAA--AAA--------L----SILQNLFSILS-STR-TPRHWDAQAQHGLLNHLGHYIY

HLEKCM----------------ESNRTA-SIL--------------S------INRYFMG

IQN-FLHAHHYGACAWDHVRLQAHICFQHM-DTL----------------------

>Apfo_1

------------------------------------------------------------

----------------------------------------------------H------P

QQAA--DIA--------L----RILQHLFTTLS-SPS-TPHHWDAQARHRLLNNLQHYKH

NLEQCV----------------PANGML-FQL--------------N------ISRYFRH

IQD-FLHTHNHSACAWDHVRLEAGACFQHL-HNLTR--------------------

>Apfo_4

------------------------------------------------------------

----------------------------------------------------H------P

QQAA--DIA--------L----RILQHLFTTLS-SPS-TPHHWDAQARHRLLNNLQHYKH

NLEQCV----------------LANRML-FKL--------------N------ISRYFRH

IQD-FLHTHNHSACAWDHVRLEARICFQRV-DTLIR--------------------

>Fape_2

MTAPAHGATPL------------------LLLLTALATA------LACHHLRPGA-ATXX

XXP------MPPRPTQPCQQHQARFPDT--L-------------LHTS----H------P

QQAA--ATA--------L----RILQHLFHTLS-SPS-TPQHWDTGARHHLLNHLQHYIQ

RLEQCP----------------TANRTL-LKL--------------S------INRYFTH

IQD-FLHTHNYSACAWDHVRLEARACFQRL-DTLIRQ--MKS--------------

>Tyal_4

-----HSALAL------------------LFLLPALATA------LTCHHLH--------

----------------------------------------------------H------P

QQAT---TT--------L----HILQHLFITLS-SPS-TPHHWDTQAQHHLLNNLQHYIH

HLKQCL----------------PGNGML-FEL--------------S------INKYFGY

IQD-FLHTRNHSPXTWDHVHLKAHTCFQCL-HNLTCI--MCN--------------

>Tyal_3

------------------------------------------------------------

-----------------------AFPDT--L-------------LHTH----R------P

QQAA--HTA--------L----RILQHLFHTLS-SPS-TPQHWDHDARHRLLNNLQHHIH

RLEQCL----------------AGSATL-DQL--------------T------ISKHFSD

IQH-FLRTHNHSACAWDRVCLQACAWFLQV-GTLIRQ--MTSQDA----PLTSAST

>Stca_1

MAAPAHSTPVL------------------LLLLPALSAA------LRCANLRTQQ-STFN

WDSLQLLHAMAPSPPQPCAQHDPPFPDT--L-------------LAIQ----S------P

QQAT--AAI--------L----RVLQHLFTTLS-NEN-TPAHWDSQAHQQLLNQLHDQIQ

LLQQCL----------------PRADAD-VKL--------------T------INGYFRR

IQD-FLRTNHHSPCAWDKVGLEARASFQRI-HNLTR--------------------

>IFN4_cami

--------MSLP-----------------VLLLCSPT-SGHG---SDC--M---Q-RRYW

RDLLESLN------PVACRGE--DVRE-PVML--------------------N------P

VKKD--KAAV-----T-L----QVLEEILRLFR-K---STVPWTNSK---FLNQVYQVIY

ELQNCM------TS--------S-----DLPVRS------------T------IKARFAN

LEG-FLNEKSI--CAWEIVHLETRKILQ----EVSRN--HARK-------------

>IFN5_cami

--------MSLP-----------------VLLLYSPT-SGHG---SDC--V---Q-RRYW

RDLLESLN------PEECRNE--DINP-APIS--------------------K------L

AKQN--KAAV-----I-V----QVLEEVLKLFS-K---PDAPWSNSKALTFLNKVSQILS

DLQSCM------QS--------P-----DSRVRS------------T------VIERFAK

LER-FLKAKSV--CAWEIVHAETRKIFQ----QVEQR--HARK-------------

>IFN9_anoli

--------MA-----------------------GQQ--S-------------SQ------

----------------ECLED--DFGPRKIL---------------------K-------

SQED--AKMA-----I-G----LILQQIQIVFQ-LNF-TQAQWSGKVTDLLSRALDQQHM

QWRRC-----A-----------TA---EAAKLR-------------S------LKRYFRK

LHT-FLRGRQYSFCAWKMVRYELLVIYPIL-NELMR---LEK--------------

>IFNB4_ONMY

--------MAVLK---------W-LSICLTLF----CQGTA--VSKPCRWTQFRL-GKLN

DVSIDLLSDMGGIFPLMCAEE----------N--VE----QMFPEDLY----K------N

TEGE--DVSV-----V-A---LEAMRYVDQLYN-NSL-TSVTWNKIKLNMFQNVIYRQVQ

NLELCV--------VGGVWE-------S-S----------------GDGGSVTLKTYFNK

LNT-VLKEKEHSACAWEIVRKEI---RENLKKFI-------DS-------RVKP--

>IFNB1_SASA

--------MAVLK---------W-LSICLTLF----CQGTV--ASKPCRWTQFRL-GKLN

DVSIGLLSDMGGLFPLMCAEE----------N--VE----QMFPEDLY----K------N

TEGE--DVSV-----V-A---LEAMRYVEQLYN-NSL-TSATWSKTKLNMFQNVIYRQVQ

NLELCV--------VGGVWE-------S-S----------------GDGWSVTLKTYFNK

LNT-VLKEKEYSACAWEIVRKEI---RENLKKFI-------DS-------RVKL--

>IFNC1_ONMY

--------MILQTV--------W-MS---AFL----CLAQV--CSVP---MPCQLQGQLV

RITHNLLRDMGGNFPLECLQE----------N--VF----VAFPATAF----T------I

SGES--QLSS-----SGAMAIYETLKNIDTLFG-ADD-LPTKWDQQKLENFQNIVYRQIE

ESK-CM--------MGSVDT-------S-DYLIR----------TEG------LKTYFGN

IAA-VLKEKNFSYCAWEVVRKEL---LYSL-QFILEH--NSDS-------LLRT--

>IFNC1_SASA

--------MALQTI--------W-MS---AFL----CVAHV--CSMP---MPCQLQGQLV

RITHNLLRDMGGNFPLECLQE----------N--VF----MAFPATAF----A------S

SGAP--QLGS-----SGAKAIYETLKNIDILFE-ADD-LPTQWDQQKLKNFQNIVYRQIE

ESK-CM--------MGSVDT-------S-DYLIR----------TEG------LNTYFGN

IAA-VLKEKNFSYCAWEVVRKEL---LYTL-QFILEH--NSDS-------LLRT--

>Eslu_1

--------MALTTI--------W-VS---ALL----CFVHV--WSMP---MTCHIQETLM

ESAKDLLRDMGGHFPLECLQG----------K--VN----ITFPAPAF----A------T

SSTP--TLSG-----SGAKAIYETLKNIELLFG-AED-LPTKWDQQKLDNFQNIIYTQID

HSK-CV--------SGSVET-------S-DYPVR----------AAA------LKTYFGN

IEA-ALKEKKFSYCAWEVVRKQV---LETL-IFILTK--NSDC-------LLRT--

>Anja_11

--------MAF-----------WFIF---AFL------AQA--WSMP---QTCQLDGNLI

RTSHNLLKDAGGHFPLQCIKE----------N--VL----IMFPSSAF----E------S

NGTI--EQET-----GVRMAIYETLRSLSLVLE-DGD-LPTKWDEKIMDDFQNIVYRQVD

----------------S-------------------------------------------

-------KCSFSFCAWEIVRKEL---VRTL-HFILDH--RSDM-------LCRL--

>clha_a13

--------MVLQSF--------W-VT---IIL----CFVHV--STVP---ITCGLQRRLV

EKSHSLLESMSGLFPVECLEH----------N--LP----IAFPSSAF----M------T

SEAA--E-SA-----GAEKVAYETLKLIDTLFA-NDS-MPTSWNN--LEDFQEIIYRQIE

ESE-CI--------M-TQSK-------D-DFPTR----------NAA------LKTYFDK

IAT-ILKEKESSDCAWEVVRKEI---LYTL-KFIL-Q--SSNY-------LI----

>IFN3_DARE

--------MDLHRV--------W-LC---TFF----CFVQV--WSLP---TNCNLQKNLM

KRTYTLLET-AGLFPMQCLDD----------R--VS----IPFPQNVF----E------Y

NE-D--QVTG-----V-EKAVYQTLQNIDALFE-NFG-DPDQWDAEKLDDFRGIVYRQIK

YST-CI--------MNKTEV-------A-DFPSR----------EAS------LKVYFET

ISS-TLKEKNSSYCAWEIVRSEI---LRTL-EFILKN--NSDI-------ML----

>IFN2_DARE

--------MEFWQV--------F-LC---PAL----FFAHI--TSKP---TNCFMRRKHV

KTAYSLLESMGGLFPRECLKE----------N--VR----ITFPKYAL----Q------S

NNSN--QKTG-----V-AKAVYKIMDHIDFLFA-NDS-YPEAWNKRKVDNFQNIVYRLTK

ENQ-CI--------MRMRAT-------V-DFPAR----------DDA------LKSYFNK

LAT-LLRNKDNSFCAWEVVRHEL---LGVL----------SDI-------IL----

>Anja_10

------------------------------------------------------------

------------------------------------------------------------

-------QSD-----IIRTVIYETLYSINSLFE-NDD-FPTDWDEIKLQDFQNIIYRQVD

KST-CV--------SS-------------------------------------LSPEM--

--------------------------------------------------------

>Leoc_1

---------------------------------------------MP---TRCAFREHLI

EVSLNLLKDMGGHFPRECIKD----------N--VL----ITFPANVL----F------S

SFAQ--TQNE-----NIQPVVYETLRSVNTLFQ-SEG-RPSTWDQRKLEDFQSVVFRQVS

DFKKCA--------LRRRET------------------------SSANNSTVQLKTYFKK

MGR-FLEEKNYSSC------------------------------------------

>Leoc_2

--------MALEGS--------W-LC---VVF----CLSQA--WAMP---TRCAFREHLI

EVSLNLLKDMGDGFPKECLSD----------N--VV----IAFPAEAY------------

EFAE--TQKE-----DFEIAIYKTLNSTDALFE-NEG-RPTSWGQRAVDEFQNLVFRQVQ

DFNTCV--------PGDVET-------TGN--------------SSAAYRITLLKTYFQK

MEN-VLQEKNYSSCAWEIIRKEL---LGIL-QVILDK--NAEI-------VV----

>Scma_1

--------MTP-------------LSVLLLQL----CSFQV--VAMP----TCKLQANVV

LKTHNLLRDLGAAFPVHCLQY----------N--VN----ISFPDSAF----P------D

ATAH--PQCR-----RALWVVYESLRGMQLILE-QND-SPVTWDEGILDNFQNLQHRLLE

DGS-CL--------ST---K-------GPDV----------------------FSSYFSN

VTD-VLQQQDSSVCGWMALRRDV---LSVLKTALREH--NSCF-------T-----

>anja_f

----------------------------------------G--KRLP---KDCQQEAMRI

NISHDERQKL-----LDLKEE----------N--IS------------------------

-------------------TLQQVFNLTWRIFR-KNM-TAVNWNMSKLKSFRKLLKRQSR

TFSTCA--------RL---T-------SSSRPAK----------NDG------KRVYFRK

LSR-FLSTEKYSRCAWEIVRAEI---LTLLHVPRIAH--NSGH-------KENAP-

>IFN1_frog

--------MS--------------VS---VLL----LIT------LGSSGQPTKG-KDVY

RTQLNIN------------REVRTLLGN--M-------------GAIP----Y------S

ECEDNWRLQI-------------TIHQFSVIFT-DNL-A----NSVEMSKMQTLLYWY--

----------------------STSLKE-LTLKE----------TKK------IRRYFRK

MLK-YLMKKGYSRCAWASVRDEMEKVLLLV-TWHTDI--LLKK-------HLRGHV

>IFNF2_ONMY

--------MATLN----VS--FV-VH---LLC----VIV------LKCSDQKEQM-YNLS

QTRQTLNDLAMERRPRGCIPEAEMIMVQ--R-------------PTLS----K------E

EVEKVWTLRL-------------AFQLASELFQ-QNL-TLVKWNSIKLRDLQDLLARQ--

----------------------YMTVRD-MRLRQ----------NLP------IKNYFKQ

LDD-FLSRESFSLCSWEVVRTEMGSIL-------RDF--YKKS-------KMRKHV

>IFNF1_ONMY

--------MATLN----VS--FV-VH---LLC----IIV------FKCSAPKVQK-YYLS

QTHQTLNDLAEERLPRGCIPEAERLRVQ--R-------------PALP----I------E

EGEKVWTLRL-------------AFQLASELFQ-QNL-TLVKWNSVKLRDLQDLLARQ--

----------------------NMTVRD-MSVHL----------NLP------IKNYFKE

LED-FLSHERFSACSWELVRAEMGSII-------SQA--IRNA-------K--KHV

>eslu_f

--------MAAHN----VT--FV-VH---LLF----ALA------LTCCD---ET-YYIF

KTRQVVNDLAMGRKPV-CVQEAARIRVH--R-------------PTLS----L------E

VGERFWTLSL-------------VFHLACELFQ-RNL-TLVKWNVNQLRELQELLARQ--

----------------------NRTVKD-IRLGQ----------SLP------NKKYFKQ

LDD-FLSRETFSLCAWEVVRFEMGRIL-------RDF--HKKS-------N-SKKT

>IFN3_cami

MAHSCVWSLCL-------------------LM----VMSG-----LA---LSARLHHFFR

RKTMDTLNEMA---PISCRDH-------NLL------------PLDLT----R--LWGGM

EPGD---RKL-----IAH----RALRRIRAIYS-GTL-HNATWDRERTVFFRTLLDAQLE

DLKESL----G--------KRPSS----KSK-N-------------S------VRKYFRK

LRS-FLRKEGYSTCSWAVVLDGTRRILQ----QLTE---MN---------------

>Eslu_6

--------MS--------------------------------------------------

-------------------ED---------------------------------------

------VAVV-----A-L----KTFGYVEQLF-DSKL-TPSTWNNGTFHLFKNCIFRQIQ

GLQEC------------------------------------------------V--NLRP

I-------------------------------------------------------

>Anja_7

---------V--------------------------------------------------

-------------------EE---------------------------------------

------RIIF-----I-H----EVINNIKDLYIKGKY-DTVTWDPKKLQMFQLNLHRQAS

ELKEC------------------------------------------------VRPNFAP

PQN-AGHDKEYR--------------------------------------------

>Opha_4

MAATSMGLLCL------------------VLLLAVPA-LG-----LHCNLLKWQQ-QRLN

QQSVELLKGMK---SPVCL--RKEVTP-QIL---------------------R------I

QRPRA-AKAI-----L------EMLHGFLHLFK-EDH---VAWDATLRKRFLPSFSAPV-

----CI----------------SI------------------------------------

---------------------QVVLDLQWF-T------------------------

>Thsi_4

MPSKRMGLLCL------------------VLLLAAPT-WG-----SNCNLLKLHQ-RRLN

RQSVELLRRVK---PAECL--RKVFSP-KIL---------------------G------I

REPRE-AKAV-----L------EVLQGFLHILK-DEH---VAWKATLQKRFLPMLHAQIQ

RIQGCL----------------GE-GRKEEKL--------------Q------LKKYFRS

IGN-FLEENGLDSCTREFVRHEIQLDFIYL-DRLTER--ME---------------

>Vibe_4

----MVGLLSL------------------VLLLALPA-SG-----RNCDLLKLQQ-QRFN

RQSLELLKGMK---PPECL--WKAFSP-KIL---------------------E------I

RQPRA-AKAV-----L------DILHGFLLLLR-DDH---VAWKATLRKRFLEKLHAQAE

RIQRCL----------------GE-GKKEEKL--------------Q------LKKYFHS

IGN-FLKEKGLDKCTQEFTRHEMQVDFIYV-DRLTER--MK---------I-----

>Pybi_6

MSATPTELLCL------------------VLLLSAQV-TA-----LNCNFLKLQQ-QRFN

RHSVELLKGMS---PQECL--RKTSNP-TVL---------------------E------I

HQPQI-AKTI-----L------EMLHGFLNILS-DDC---NAWEAKLRNR----------

---KCV----------------SA------------------------------------

--------------TVQFPLTELRCPFTYF-QVEAEE--ND---------------

1. **IFN1 CHOM:**

>oran_L3

MR---GHLLTG------------------AQAADPP--K------KQCYL--GKF-KSLS

PQELEAFKKAKDML-------------------------------LTD----R------T

ERLL--FLEA-----E-L----KLLG---------KM-SKSNLEGYLGRPLWTLRYIS-Q

ELQRCI----------------AQES-R-HSS--------------R------LTHWLHK

LQE-AREK------------------------------------------------

>gaga_L

MV---GVTIVG------------------AFPQVTP--K------KSCSL--SKY-QFPA

PLELKAVWRMKEML-------------------------------LTN----R------K

DRIT--LVEA-----E-L----DLLT---------NP-TTQRLAETCQQPLAFLTQVQ-E

DLRDCL----------------EAPSHQ-PSG--------------K------LRHWLQK

LET-AKKKET-------------AGCLLRC-AAQRED--CT---------------

>pesi_L3

TM---GYRVAE------------------AFPKGAL--R------TKCHL--AKY-KSLP

PRELEAFKKTKDLL-------------------------------LSD----R------K

DRVI--LVEK-----E-L----DFLE---------DV-EDPSLSKLLPRPLEILSQIR-E

DLRRCT----------------QTPSHS-HSK--------------R------LNSWLQN

LQA-SKETET-------------PACLLRC-AAYTEL--CV---------------

>gavga_L

------------------------------------------------------------

----------------------------------------------------R------K

DRVI--LVQA-----E-L----NFLK---------NI-EDPNLSEQLPRPLEFLTHIG-E

DLKSCT----------------HHHSHK-KSE--------------K------LSSWLQK

FHE-AKNKET-------------RECLLAC-AALKDD--CIS-------P------

>xetr_L1

-------MEVT------------------AHP-----HR------RHCHM--SRY-RSVS

PSDIRAVRRLHNPF-------------------------------SDG----I------K

DRLI--LTLE-----R-V----TLLT---------NM-TESPLAKLLSLPLTMLLSLE-D

DLKICR----------------PLYSDP-PSE--------------Q------LMPWLHH

LKH-FREKVS-------------SECVIMC-WANNE--------------------

>napa_LR1a

---------VS------------------GRL-----HK------RLCPM--SRY-LSVA

SSDITTLKQLQHMS-------------------------------SNA----M------R

DRLI--LTLE-----R-V----SLLT---------NM-SMSAQPDTIKQSLMVFLKLR-D

DLLVCR----------------PEYSEP-TSP--------------E------LKLWLHH

LQR-FKETAS-------------PDCVVTC-WALNQ--------------------

>chpi_L2

---------VD------------------AFAEGAP------------------------

------------LR-------------------------------DPA----A------N

QNLL--VLEK-----K-V----DVIQ---------NL-SDPELVRNASKPLEILASIQ-E

DLRSCP----------------QQPV---------------------------LARWLRN

AYA-GKSEGS-------------ARCLLQN-VAHNES--CH---------------

>oran_L4

---------LE------------------VSCSSFM-DG------KKCYL--AHY-GSLD

PQVLRDVKDLQN------------------------------------------------

GRLL--LLER-----E-L----AHLR---------NL-SGLDLGRNATRPLQLLAAIC-E

DLASC----------------------S-ARQ--------------R------EMSWLRS

KAK-AKKEVT-------------PRCLLRQ-ATYLKP--CD---------------

>scca_L

-------MATF------------------LAPSLSP-GQ------DGCSL--SRY-ARLP

PSVFKLFGDFHRQE-----PG---------------------------------------

ERLL--LVEA-----E-F----RLMQ---------EL-ETSILQNLTKKVLEVLYQMC-W

NLGRCL----------------TQKDQK-YPK--------------Q------LKKFLRN

LKH-AGRLGR-------------T---LSC-VASGED--C----------------

>Xetr_4

--------MLL------------------SLTSIVH--S------QSCKWLHPKQ-EYLN

TQILKAFNEMMPLK-------------------------------ETE----E------I

SQVE--AGAL-----A-L----NEYM---------KH-HESMGCKQQAERFQQLLYYQIH

QLEACV----------------TEENDL-LKE--------------S------ISEEFNL

LET-MVLEKN-------------SACVLRR-QRLLQR--PQ---------------

>Napa_2

---------------------------------MVS--A------QTCKWLHRNQ-EAWT

RQILHNFNQMVPAE-------------------------------KTG----Q------P

TQAE--SAAI-----A------DEHI------------------------------YQLA

RPSRCC----------------TAEGKY-VSY--------------T------VNQGREA

VKP-VLA--------------------VSR-YILLVK--PQL--------------

>IFN1_coela

--------MTL------KM--LLAFC---LLL----LVSNGT-FCQDCKKWVKQ-----Y

NKGLQYLEAMGGEFPLKCLAQ-----------------------LDVP----RKVVLRHS

KGER--RIEL-----V-H----DTLEHISKTYS-NN--TSTKWDEENLKKFQNVIHLESE

ELRACL--------------QERVSNKN-TQWRK----------KMT------LSRHFKE

TGN-FLQRQNYSSCAWETVRAITRMILQLI-Q------------------------

>IFN2_coela

--------MAL------KT--FVALC---LLL----FVPIGI-FCQECEELNSQQ-RLRI

RESLQELEGVGGKFPSQCLAQ-----------------------FNLH----KKVLLKHS

KGER--RITL-----V-Y----EILQQINRIYR-KN--PSATWDQNKLERFQNVLHSQTE

ELWKCL--------------EKKMSNMN-SQWNN----------AMK------LSKNFKE

MEK-FLKHQNYSSCAWELVRTITRRVLQQV-ER---------K-------A-----

>IFN3_coela

--------MAL------KC--LWTMF---LLL----LDFPVA-FPEQCNWVHLHQ-KY-S

TSKLPLLDEMGAGFSESCMIE-----------------------IVKD----KKLTLKFP

NSDH--MIETDIMPTV-C----EILNFIGNIYN-KNL-QLVPWDKKKIEHFQTVLYHEVE

EIKKCL--------------PGEKTNAN-SHSNS----------NMK------LQDYFST

LEN-FLEQKEYSPCAWEVVRAHIRTLLQFT-DRLTTV--ITKN-------E-----

>IFN4_coela

--------MAL------KC--LWTMF---LLL----LDFPVA-FPEQCNWVHLHQ-KY-S

ISKLQLLDEMGAGFPPHCINE-----------------------KGVD----EKVTLKFP

KQDH--MIQMEIMPTV-C----EVLNFTGNIYN-KNL-QFVPWNKRKVQHFQTVLHHEVE

ELKKCL--------------PEEKTNAN-SHSNS----------NMK------LQDYFSK

LEN-FLEQKEYSLCAWEIVRVHIRKLLQLT-DRLTTA--IRKT-------E-----

>IFN5_coela

--------MAQ------KF--QIISL---ILF----IVSQVR-ADDQCTWSTAQQ-MHLN

KRNLNLTDDMGKFSPAECTTE-----------------------IQEV----QKMRLRFP

ENE-------DVIFIV-Y----TTLRHISKIYS-KNL-QPVSWNKTVLHEFQAAVHSQVE

ELEKCL--------------MEKMVDHY-LERKV----------ELK------LRNYFKL

LEK-MLAEKENNQCAWRFIRAQVRKFLYRI-DQLTAW--IGKM-------KNQSS-

>IFN6_cami

--------MAV------HY--QCGLS----LF----AMLCVS-LTLGCSTLRLQK--ILI

ATTLNTLDEMGGHVPRHCVAVGAELRIA--S-------------PDLR----L--LLQPL

QNND--RILL-----L-H----KTFQHLNKIFH-KNM-KSVTWDLTQVNHFRELLVTQRD

VVKDCI--------------QDSAS--D-SMLSA----------LST------IHTYFRK

LKK-FLKQQRYSACAWEVIRMETRARLQQI-LILTAR--MTKG-------N-----

>IFN1_cami

--------MAV------HY--QCGLS----LF----AMLCVS-LTLGCSTLRLQK--ILI

ATTLNTLDEMGGHVPRHCVAVGAEQGIA--S-------------PDLR----L--LLQPL

QNND--RILL-----L-H----KTFQHLNKIFH-KNM-KSVTWDLTQVNHFRELLVTQRE

VVKDCI--------------QDSAS--D-SMLSA----------LST------IHTYFRK

LKK-FLKQQRYSACAWEVIRMETRARLQQI-LILTAR--MTKG-------N-----

>IFN1_sqac

--------MVF------PS--VWRLW---ILL----VLLPGT-LSQDCQRLQLLD--NIN

NQALDALREMGGPIPLHCKTERTSLRAK--S-------------LDLH----Q--LSKRL

QTPD--RIQI-----V-H----QTLRHLTKIYS-MNL-GSVTWPRDKVENFRLLLDRQLG

ELEECV--------------RKPVP--E-SRPRR----------NAP------IHKYFRK

VEK-FLKQKRFSDCAWEIIRAETRARLQQI-LFITAK--IRRR-------S-----

>IFN2_sqac

--------MVF------PS--VWRLW---ILL----VLLPGT-LSQDCQRLQLLD--NIN

NQALDALREMGGPIPLQCKTERTSLRTK--S-------------LDLH----Q--LSKRL

QTPD--RIQI-----V-H----QTLRHLTKIYS-MNL-GSATWPRDKVENFRLLLDRQLG

ELEECV--------------RKPVP--E-SRPRR----------NAS------IHKYFRK

VEK-FLKQKRFSDCAWEIIRAETRARLQQI-LFITAK--IRRR-------S-----

>Leer_25

------------------------------------------------------------

------------------------------------------------------------

------TLLV-----V-D----QMLRQFRKIYS-MNL-ASVTWLQDKVENFRLLLDRQIR

ELENCV--------------RNTGS--E-TRPRR----------SAA------VHNYFRK

LGK-FLKRK-----------------------------------------------

>Leer_37

------------------------------------------------------------

------------------------------------------------------------

-TQE--RIHT-----A-H----QTLQQINNVYS-MNL-DSITWAQHKVENLRLLLDRQLR

TLEECV--------------KKPGS--K-STSKR----------NTR------ISNYFRK

LRK-FLKRNRFSDCAWEITRTE----------------------------------

>Scca_1

-------------------------------------VISGE-IVWGFNEILFYS--S--

---------QGGSFPRHCIKHRHALKTK--P-------------LNLV----K--LSKGL

EKED--QIQI-----L-H----QTLRHISKIYS-MNL-GSVTWDRDTVENLRLLLDRQLS

ELE---------------------------------------------------------

--------------------------------------------------------

>Leer_3

------------------------------------------------------------

------------------------------------------------------------

-DMD--TAVV-----V-Y----QVVSQFKEIYH-MDR-TSVTWPQDVMKRFGIKLDAQSS

ILENCV--------------RNAGS--D-AQTQK----------KAT------IQEYFKK

LSE-FLNRERFSACAWE---------------------------------------

>Leer_4

------------------------------------------------------------

------------------------------------------------------------

-APD--LAGI-----V-Y----QVVTQYNRIYN-MDR-TSVTWPQIIMTSLGFHLDVQTT

VLEDCL--------------THRGA--M-AQRQN----------RET------IHDYFRG

LSE-FLNRERFSACAWEAIREEMILWYQQV-FKFF---------------------

>IFN2_cami

--------MPL------RC--VWKLC---LCL----ALLATQ-TPSLACNLPLHN--LMC

QRSLNLLLWMRDSIPFHCVREMGST---------VDLR------LNLR----N--VTGPL

QPGD--RMQV-----Y-L----QTLHHLNEIYS-NNV-TS-TWDQEKILGFRFVLDEQQM

EMEKCA--------------KEPAS--D-AMVHT----------TSA------IRTYFTK

LGR-FLRQKRFSACAWEVIRAKTSRRLQEM-LTLAMK--EAKT-------R-----

>IFNA_human

MALTFALLVAL------------------LVLSCKSSCS------VGCDLPQTHL-GS--

RRTLMLLAQMRRISLFSCLKDRHDFGPQEEF--------------G-N----Q------F

QKAE--TIPV-----L-H----EMIQQIFNLFS-TKD-SSAAWDETLLDKFYTELYQQLN

DLEACVIQGVGVTE--------TPLMKEDSIL--------------A------VRKYFQR

ITL-YLKEKKYSPCAWEVVRAEIMRSFSLS-TNLQES--LRSKE------------

>IFNA_horse

MALPVSLLMAL------------------VVLSCHSICS------LGCDLPHTHL-GN--

TRVLMLLGQMRRISPFSCLKDRNDFGPQEVF-------------DG-N----Q------F

RKPQ--AISA-----V-H----ETIQQIFHLFS-TDG-SSAAWDESLLDKLYTGLYQQLT

ELEACLSQEVGVEE--------TPLMNEDSLL--------------A------VRRYFQR

IAL-YLQEKKYSPCAWEIVRAEIMRSFSSS-TNLPQS-------------------

>IFNA_pig

MAPTSAFLTAL------------------VLLSCNAICS------LGCDLPQTHL-AH--

TRALRLLAQMRRISPFSCLDHRRDFGPHEAF-------------GG-N----Q------V

QKAQ--AMAL-----V-H----EMLQQTFQLFS-TEG-SAAAWNESLLHQFCTGLDQQLR

DLEACVMQEAGLEG--------TPLLEEDSIL--------------A------VRKYFHR

LTL-YLQEKSYSPCAWEIVRAEVMRSFSSS-RNLQDR--LRKKE------------

>Oror_10

MAPTVSLLLAL------------------VLLSCHSNCS------LGCDLPQTHL-AN--

TRALMLLQQMRRISPFSCLKDRNDFGPQEAF-------------GG-N----Q------F

QKAQ--AIAV-----V-H----EMIQQTFQLFS-TEG-SAAAWDETLLDKFCTALYQQLT

DLQACLMQEAGLEG--------TPLLKEDSIL--------------A------VRKYFHR

ITV-YLQEKKYSPCAWEIVRAEVMRSFSSS-TNL----------------------

>Loaf_11

MAFSFLLLIAL------------------VVLSCNSTCS------LGCDLPQSHL-AN--

RRTMMLLGQMRRISPFSCLKDRNDFGPQEEL-------------DG-N----K------F

QKAQ--AISV-----H-H----EMIQQTFNLFS-LQA-SSAAWDKTLLDKLYTGLYQQLN

DLEVCLMQEMGVEE--------APVINEDSML--------------A------VRKYFQR

ITV-YLTEKKYSPCAWETVRAEVMSSFSAS-TNWKER--LRSKEGDLAP-------

>Ptva_8

MALLFSFLMAM------------------VVLSCQSICS------LGCDLPQTHL-VN--

RRALMLLGQMRRISPFSCLKDREDFGLQGAF-------------GG-N----Q------F

QEAQ--AIAV-----F-H----EMTQQTFLLFC-TEV-LSAAWDETLLGRFCNGLYQQLD

HLEACQTQELGAEE--------TPLLDEDSTL--------------A------VRKYFQR

INL-YLQEKKHSPCAWEIVRAEIMRSYSLS-THLKEK--SRSKD------------

>Ereu_6

MAPSSLFLKAL------------------LVLSCSYIFG------LGCDLPQSHP-VN--

RRPLLLLGQMRRLPPFSCLKDRHDFAPQEVF-------------DG-Q----Q------F

QKAH--ALSV-----L-H----EMLQQIFHLFS-TKH-SSADWDEGLLNSFCAELHQQLN

VLEGCQTQEVRVEQ--------TPRMK-DSIL--------------A------MKRYFQR

ITM-YLREKKYSPCAWEIVRVEIIRAFSLS-TKLQEK--LRSKD------------

>IFNA_mouse

MARLCAFLVML------------------IVMSYWSTCS------LGCDLPHTYL-RN--

KRALKVLAQMRRLPFLSCLKDRQDFGPLEKV-------------DN-Q----Q------I

QKAQ--AIPV-----L-R----DLTQQTLNLFT-SKA-SSAAWNTTLLDSFCNDLHQQLN

DLQTCLMQQVGVQE--------PPLTQEDALL--------------A------VRKYFHR

ITV-YLREKKHSPCAWEVVRAEVWRALSSS-VNLLPR--LSEKE------------

>Oror_9

MAFVLPLLTAL------------------VVFSYGPGGS------LGCDLSQNHR-IS--

RKNFMLLGQMRRISPRFCLKDRKDFGPQDMV-------------DG-S----Q------L

PKAQ--ATSV-----L-H----EMLQQVFCLFH-TER-STATWDTSLLDKLRTGLHQQLE

DLDACLVQAMGDEE--------TALGVTGPTL--------------A------VKRYFQG

IHL-YLKEKKYSDCAWEIVRVEIMRSLSSS-TNLQER--LRIMNGDLGSP------

>IFNT_cow

MAFVLSLLMAL------------------VLVSYGPGRS------LGCYLSEDHL-GA--

RENLRLLARMNRLSPHPCLQDRKDFGPQEMV-------------EG-S----Q------L

QKDQ--AISV-----L-H----EMLQQCFNLFH-IEH-SSAAWNTTLLEQLCTGLQQQLE

DLDACLGPVMGEKD--------SDMGRMGPIL--------------T------VKRYFQD

IHV-YLKEKEYSDCAWEIIRVEMMRALSSS-TTLQKR--LRKMGGDLNSL------

>Loaf_4

MALLLSLLTAL------------------VVFSCGPAPS------LGCDLPQNHV-AS--

EKTVDLLDQMQRCPTFFCLDDRKDFRPQEMV-------------DG-S----Q------L

QKAQ--AIAF-----L-H----EMLQQIFDLFR-TMD-SFAAWNTTLLNQLLNGLPEQQE

DLETCFMQAMEEGK--------SALPIEGPAL--------------A------VKEYFEG

IRF-YLKEKEYSDCAWEFVRVEIRRSFSSS-TALQER--LRRKDGDMSSS------

>Loaf_5

MAFLLFLLTAL------------------VVFGCGPAPS------LGCDLSKKHL-TS--

KKTFVVLDQMRRLSPFSCLKERKDFRPQEMV-------------DG-S----Q------L

QKAQ--VISV-----L-H----EMLQQIFNLFH-TKD-SSAAWNTTLLDQLHSGLYLQLE

DLEACLVQAMEEEE--------SVLAIESSAL--------------A------VKRYFQG

IHS-YLKEKEYSDCAWEIVRVEIKRSFSSS-TNLQER--LRRKHGDMGSS------

>Dano_12

MALQLSLLMAL------------------VVFSCGPVPS------LSCDLPQSQL-VD--

RKTFVLLGQMGRISPFSCLKDRADFRPQEMV-------------DG-S----Q------V

QKSQ--AKFV-----L-H----EMFQQIFNLYH-TEG-SSAAWNMTLLDQLLSTLHEQLE

DLEACLLQEMGEEE--------TLLGIEGPVL--------------A------MRRYFQG

IRL-YLQEKKHSDCAWEVVRMELRRAFSSS-PNLKER--L----------------

>IFNO_human

MALLFPLLAAL------------------VMTSYSPVGS------LGCDLPQNHL-LS--

RNTLVLLHQMRRISPFLCLKDRRDFRPQEMV-------------KG-S----Q------L

QKAH--VMSV-----L-H----EMLQQIFSLFH-TER-SSAAWNMTLLDQLHTGLHQQLQ

HLETCLLQVVGEGE--------SAGAISSPAL--------------T------LRRYFQG

IRV-YLKEKKYSDCAWEVVRMEIMKSLFLS-TNMQER--LRSKDRDLGSS------

>Dano_2

MAFPVSSLVVL------------------MMIFSSPIGS------FSCGLPQSLV-RK--

QETFTVLSQMGTISLLSCLKDRTDFRPQEMM-------------DG-S----Q------V

QKTQ--AMSV-----L-H----EMLQQIFHLFH-TEG-SSAAWNTTLLDQLRSGLHRQLE

DLETCLLQEMG-ED--------SVLAMEGPTL--------------A------VRRYFQR

IRV-YLQKKKHSDCAWEVVRVEIRRCFLFI-NVLTRE--LRK--------------

>Ptva_4

MAPLLSLITAM------------------LVFSYGPSGS------LSCDLSQNHQ-VN--

KESIVLLHQMQRISSFRCRKDRKNFGPQEMV-------------DG-S----Q------V

QEAQ--AISV-----L-H----EMLQETSNVFG-SEH-SSAAWNTTVLHGLLSRLHWQLE

DLGTCLVLQMKEAE--------SALGMEAPTL--------------A------VKRYFQG

IRL-YLKEKQYSDCAWEIVRVEIKRAFSLS-TNLREM--LRNQDGDLRSP------

>Ptva_16

MAFLVSSLMAL------------------VVIFSSPISS------MICDLPQSLL-GK--

QETSTALNQMQRISSFLCQKDRKDFRPRKMV-------------DG-S----Q------V

QKAQ--AISV-----L-H----EMLQQTFDVFG-TKQ-SSAAWNTTLLHGLLSGLHRQLE

DLGTCLVPEMKEVE--------SVLGTEDPTL--------------A------MKRYFQG

IHL-YLEEKQYSDCAWEVVRVEIRRYLFVV-NKFTRK--EI---------------

>Dano_10

MAPPVSVLKTL------------------LMLCSIPAC-------LGCDLPLIY--GH--

QEPFMLLHQMGRLSILSCLKDRTDFQPQELM-------------DG-I----Q------L

DKMH--ATTL-----L-H----EVVQQIFNLFS-TSG-SLATWDDTLLDRFLIGLHQQLD

NLETCLGKEKEEDQ--------THLGSENSRL--------------A------VKRYFQG

ISQ-YLTEKQDSPCAWEVVRVEIRKCFLFI-NKLQGK--LRK--------------

>Modo_6

MTSWSLLPVAL------------------ALLCSSTLCS------LDCDLTLGL------

QEDFSLLNQMSTSSLVPCLKDGINFNPKEAM-------------DR-S----Q------L

QKEN--ATVI-----V-L----EMVQQIFTLFS-QNT-TPATWNQTQVIQLLIRLDQQLE

QLERCLGQNVKWEE--------FSLRSEKTRF--------------A------LKSYFQG

ISQ-YLQGKEYSPCAWEIVRVEIRRLFLFM-SKLARK--LRD--------------

>Dano_1

MDHM-YLLLAG------------------LMLCSSLDCS------LGCPLPRSQL-ES--

KEIFTLLRQMNRIPSHSCLNDRVDFKPWKAE-------------TV-T----Q------I

PKTQ--ATCF-----S-Y----EMFQQIFNLFQ-KEN-SRAAWDNSLLDELLSRLDHNLE

QV-----EQMKVE----------NLPCADLGT--------------L------VRDYLQG

TDG-YLNEKKYSSCAWEVVRGEPEMCFPLI--------------------------

>Oror_2

MAQI-YLLVAG------------------VLLCSIPAYS------LGWNLPRSHQ-EN--

KDVFQHLEQLQRIPSQWCLKDRTDFKPWKRE-------------NI-T----P------I

QVTQ--GTCH-----H-H----LMLQQIFNLFT-TED-SRAAWNNTLLDKLLSSLHLRLH

RL-----EQMKKD----------NLDCRDLGR--------------A------AREYFHG

IHV-YLKAKEYSPCAWEVVRVEIKRCLSLM--------------------------

>Ptva_17

MAQCSSWLAAG------------------EMLSFILICS------LGGDVPWIHL-EN--

RKIVSLLRELEVIPSHFCLKDRTDFKPWERG-------------SI-T----E------I

QKTQ--RTCF-----H-H----LILQQIFSLLN-AED-SHAAWNRTLLYQLLSRLHHSLE

EL-----DQTNEG----------NLVCPDLGI--------------L------VWNYFQG

IHN-YLKQKKYSTCAWEVVRVEITARLFLM--------------------------

>Ereu_8

MFQ--FLLMTG------------------VMLSSILACS------YGQD----HP-EK--

RVILMLLTQLKNTPSLSCLKDRTDFHPWSRG-------------EI-T----Q------I

HMAQ--GPCF-----Q-K----LMLQQVFRLFN-TEA-SRAAWNNSLLDRLLSSLYDSLE

QL-----EQMEV-----------SLACPSVGT--------------D------ALKYFQR

IKI-YLKAKKYSACAWEIVRSEIEARFFLI-LGTLRR--LGQRTESLGPP-LRAA-

>Loaf_1

MALPISVLMAL------------------VMFCCRPACF------WCCDLPLSH---N--

QETFTLLNQMERISLLSCLKDRTDFRPQILM-------------DM-N----Q------L

EKTQ--AAVL-----L-Y----EMLQQTFNLFS-RSD-SLEAWDETFLDKFLLGLYQQLN

DLEICFEKERKVEQ--------IPLGTEN-----------------S------VKSYFQG

IGL-YLKEKEHSLCVGGCQSGN-QKMLSLH--------------------------

>IFNE_human

MIIKHFFGTVL------------------VLLASTTIFS------LDLKLIIFQQ-RQVN

QESLKLLNKLQTLSIQQCLPHRKNFLPQKSL-------------SP-Q----Q------Y

QKGH--TLAI-----L-H----EMLQQIFSLFR-ANI-SLDGWEENHTEKFLIQLHQQLE

YLEALL--EAEKLS--------GTLGSDNLRL--------------Q------VKMYFRR

IHD-YLENQDYSTCAWAIVQVEISRCLFFV-FSLTEK--LSK--------------

>IFNE_cow

MINKAFFEIVL------------------VLLASSTVCS------QELKLVLCQQ-RRVN

QESLKLLNKLQTSSVQQCLPHRKHFLPQKSV-------------NP-H----Q------Y

QKGQ--VLAI-----L-H----EMLQQIFSLFR-AIV-SLDGWEESHTEKFLVELHQQLE

YLEALL--QAKQKS--------DTLGSENLRL--------------Q------VKMYFQR

IHD-YLESQDYSSCAWTIVQVEINRCLFLV-FRLTRK--LSE--------------

>Oror_7

MINKPFFDIVL------------------VLLASSSVCS------RELKLVLFQQ-KRVN

RESLKLLNKLQTSSIQQCLPHRKNFLPQKSM-------------NP-H----Q------Y

QKGQ--ALTI-----L-H----EMLQQIFNLFR-AII-SLNGWEESHMEKLLIELHQQLK

YLEALR--QAEQKR--------DTLGSENLRL--------------Q------VKIYFQR

IRD-YLENQDYSTCAWTIVQVEINRCLFFV-FQLTGK--LSKQ---------ET--

>Ptva_15

MISKYFFEVVL------------------VLLASSTVFS------LELKLVLFQQ-RRVN

RENLKLLNKLQTPSIHQCLPHRKNFLPQKSL-------------NP-H----L------Y

QKGC--ALAI-----L-H----ETLQQIFSLFG-ANI-SLDGWEESHMEKFLIELHQQLE

YLETLQ--QAEQKS--------GILGSENLRL--------------Q------VKMYFQR

IRD-YLETQEYSRCAWTIVQVEINRCLFFV-FQLTGK--LSKQ---------DP--

>Dano_16

MINKHFFEIVL------------------VLLASSTLFS------LELKLVLFQQ-RQVN

RESLKLLNKLHT-SIQQCLPHRKNFLPQESM-------------NP-Y----Q------Y

QKGH--AVAI-----L-H----EMLQQIFNLFR-EKL-SLAIWEESQVEKFLIELHQQLE

HLEALQ--EPELKS--------DTLDSETFRL--------------Q------VKTYFRR

IRD-YLENQEYSSCAWTIVHVEINRCLFLF-TDSQES-------------------

>Loaf_2

MINKYFFETVV------------------VLLSSSMIFS------LELKLVHFQQ-R-MN

RESLKLLNTLWSSSIQQCLPHRANFAPQKSM-------------NP-H----Q------Y

HKGH--AVAI-----L-H----EMLQQIFNLFR-TNL-VLGSWEERHMEKFLIELYGQLE

HLEALL--EAEQKS--------GSLGTENLRL--------------Q------VKMYFQR

IHN-YLENQKYSSCAWTIVRVEIIRCLFFV-FRLTGK--LSQY---------DP--

>Ereu_10

MISKHIF--VL------------------ILLASSPIFS------LELKLFLVQL-RRLN

RESLKLLNIRQTSSIQRCLPHRKNFLPLKSP-------------SP-H----W------Y

QTEH--ALAI-----L-H----EMLQQIFNLFR-VNI-SLDDCEESYMEKFLMELHQQLE

LLEAFL--EAEQNS--------NTLSSENLRM--------------Q------VKMYFQR

IHN-YLGKQGYSNCAWTIVRVEINRCLLFM-LRLTTK--LSKQ---------DF--

>IFNE_mouse

MVHRQLPETVL------------------LLLVSSTIFS------LEPKRIPFQL-W-MN

RESLQLLKPLPSSSVQQCLAHRKNFLPQQPV-------------SP-H----Q------Y

QEGQ--VLAV-----V-H----EILQQIFTLLQ-THG-TMGIWEENHIEKVLAALHRQLE

YVESLL--NAAQKS--------GGSSAQNLRL--------------Q------IKAYFRR

IHD-YLENQRYSSCAWIIVQTEIHRCMFFV-FRFTTW--LSR--------------

>Oran_1

MTNRSSLPFVL------------------WLLLPTTIMA------QGYPKLYSHQ-WLSN

WQSLHLLDEMGGQFPLHCLKEKTNFKPAEMM-------------HP-H----Q------F

QQEN--ATEA-----I-H----DLLQNIFNIFG-RNH-SQTGWDEATVEKFLHGVHKEMM

RLELFE--EMGWEN--------STLRGDV-SL--------------H------IKSYFKG

MMD-YLKGRDYSSCAWEVTRMEAKRCFLVM-YRLTRK--LKK--------------

>Oran_7

MTNAGLIQIVL------------------VLLVSTSTVS------LSCSLLHT----VCM

EQSLKRLDRMQGKSLLSCLKDRKDFQPQELV-------------EA-G----P------F

KEGN--RAVA-----V-H----ELLQQIFTIFS-QNL-SQTGWDQSEVENFLHGLHRQLE

ELEVCQ--GTDTRW--------ASVGSDILRL--------------R------LKSYFRS

ISL-YLRDKDYSSCAWEIVRAQIRRC---I-FQFMRR--LRN--------------

>IFNB_human

MTNKCLLQIAL------------------LLCFSTTALS------MSYNLLGFLQ-RSSN

FQCQKLLWQL-NGRLEYCLKDRMNFDPEEIK-------------QL-Q----Q------F

QKED--AALT-----I-Y----EMLQNIFAIFR-QDS-SSTGWNETIVENLLANVYHQIN

HLKTVL--EELEKE--------DFTRGKMSSL--------------H------LKRYYGR

ILH-YLKAKEYSHCAWTIVRVEILRNFYFI-NRLTGY--LRN--------------

>IFNB_pig

MANKCILQIAL------------------LMCFSTTALS------MSYDVLRYQQ-RSSN

LACQKLLGQL-PGTPQYCLEDRMNFEPEEIM-------------QP-P----Q------F

QKED--AVLI-----I-H----EMLQQIFGILR-RNF-SSTGWNETVIKTILVELDGQMD

DLETIL--EEMEEE--------NFPRGDMTIL--------------H------LKKYYLS

ILQ-YLKSKEYRSCAWTVVQVEILRNFSFL-NRLTDY--LRN--------------

>Oror_6

MNHRCILQTAL------------------LLCFSTTALS------MSYRLLQFQQ-RSSN

LACQKLLQRL-PGMPQHCLEDRMDFKPEEIK-------------QP-Q----Q------F

RKED--AVLV-----T-Y----EMLQQIFGILR-RNF-SSTGWTETITENLLVEVYGQMD

RLETIL--EEMEKE--------NF-TSVVTIL--------------H------LKKYYLQ

IMQ-YLKSKEYSNCAWTVVRVEILRNFSFL-NRLTDY--LHN--------------

>Dano_11

MANRCAFQIAL------------------LLSFSTMALC------ISYNVLRFQQ-SSSN

LICQKLLKKL-NGSAEYCLQDRMDFKPEEIK-------------QP-Q----Q------F

QKEE--AALL-----I-Y----EMLQQIFGIFQ-RKF-SSTGWNETIVENLCVELYQQMD

RLETIL--EELEEE--------SFTWGDMTIL--------------H------LKNYYLR

ITQ-YLKAKEYSSCAWTVVRVEILRNFSFI-NRLTEY--LQN--------------

>Ptva_12

MTNRCILQFAL------------------LLCFSTTALS------MSYNWLRFQQ-RSSN

LACLKLLWQL-NGTPQYCHKDRMDFKPAEIK-------------QP-Q----Q------F

QKED--TVLI-----I-H----EMLRQIFDIFQ-RNF-SSTGWNETIIMNLYVTLSGQMD

RLETAM--EEMEEE--------NFTWESMTVL--------------H------LKNYYFR

IMR-YLETKLYSRCAWTVVKAEILRNFFFL-NGLTEY--LQN--------------

>Loaf_13

MTTRCILQVAL------------------LLSISTTALA------RSYKLLQFQQ-RSSN

LACQKLLWKL-NGAPESCLEDRMDFKPEEIK-------------QP-G----Q------L

QKED--AALV-----I-Y----EMLLQIFDIFL-GNF-SHTGWDETVIENLLAELSQQRD

RLVTIL--EEMEEE--------NPTSRNMTIL--------------H------LKNYYLG

IGQ-YLEAKDYSSCAWTVVQVEILRNFSFI-SGLTDY--LQN--------------

>IFNB_horse

MTYRWILPMAL------------------LLCFSTTALS------VNYDLLRSQL-RSSN

SACLMLLRQL-NGAPQRCPEDTMNFQPEEIE-------------QA-Q----Q------F

QKED--AALV-----I-Y----EMLQHTWRIFR-RNF-ASTGWNETIVKNLLVEVHLQMD

RLETNL--EEMEEE--------SSTWGNTTIL--------------R------LKKYYGR

ISQ-YLKAKKYSHCAWTVVQAEMLRNLAFL-NGLTDY--LQN--------------

>IFNB_cow

MTYRCLLQMVL------------------LLCFSTTALS------RSYSLLRFQQ-RQSL

KECQKLLGQL-PSTSQHCLEARMDFQPEEMK-------------QE-Q----Q------F

QKED--AILV-----M-Y----EVLQHIFGILT-RDF-SSTGWSETIIEDLLKELYWQMN

RLQPIQ--KEMQKQ--------NSTTEDTIVP--------------H------LGKYYFN

LMQ-YLESKEYDRCAWTVVQVQILTNVSFL-MRLTGY--VRD--------------

>IFNB_mouse

MNNRWILHAAF------------------LLCFSTTALS------INYKQLQLQE-RTNI

RKCQELLEQL-NGKIN--LTYRADFKPMEM---------------T-E----K------M

QKSY--TAFA-----I-Q----EMLQNVFLVFR-NNF-SSTGWNETIVVRLLDELHQQTV

FLKTVL--EEKQEE--------RLTWESSTAL--------------H------LKSYYWR

VQR-YLKLMKYNSYAWMVVRAEIFRNFLII-RRLTRN--FQN--------------

>Ereu_12

MANRYIFQIAL------------------LLCI-TTALA------ESYTLDQSQQ-KSSI

LVCQDLLNQL-NGSATDCLKQRMNSKPEEIK-------------NP-Q----L------L

QKED--LVLV-----T-Y----ELFQQIFGIFS-RNF-SRTSWNETIVEKLLMELYQQKN

QLKTTV--EEIKET--------NDIWGNKHIL--------------N------LKKYYFS

LMR-YLKANKYSSCAWIIIKTEIIRNFVYL-DKLISY--FSN--------------

>Modo_7

MVYRGILYLAL------------------LLLFSPSISS------KGYDSLRFHQ-RRTN

QRSLMFLNKMIGKLHPECLQERMDFQPREIV-------------QP-R----Q------C

QREN--ATMI-----I-H----EMLQQTLILFS-SKN-ACPDVNDTIIEPFLSGIYQQML

HLE----EEMDQAN--------SSWESLESIL--------------R------LKNYYQG

ITN-YLKNKEYSSCACKIVQVETRRNFSFL-YKLTEY--LKN--------------

>IFNK_human

MIQKCLWLEIL------------------MGIFIAGTLS------LDCNLLNVHL-RRVT

WQNLRHLSSMSNSFPVECLRENIAFEPQEFL-------------QY-T----Q------P

MKRD--IKKA-----F-Y----EMSLQAFNIFS-QHT-FKY-WKERHLKQIQIGLDQQAE

YLNQCLEEDKNEED--------MKEMKENEML--------------E------LRRYFHR

IDN-FLKEKKYSDCAWEIVRVEIRRCLYYF-YKFTAL--FRRK-------------

>IFNK_dog

VIRKCLWPACL------------------VGLLITGVLS------LDCNLLHFHL-RKVT

WQNLRLLSSMSNSFPVECLREIKAFEPQEIL-------------SH-T----Q------P

VKRY--IVEA-----F-Y----EMSIQAFNIFS-QYT-FKSTWENDYLKQIQIGLDQQLQ

YAERCLEEEEKEDD--------SKEMEEDGIL--------------E------LRRYFNR

IDN-FLKEKKYSHCAWEIIRVEIRRCFYYY-FKFAPL--LRKK-------------

>Ptva_13

MIRKCLWPACL------------------MGLLITGILS------LDCNLLN-----RVT

WQNLKLLSSMRNSFPKDCLRENKAFEPQEIL-------------YS-T----Q------L

LKRD--IKEA-----F-Y----EISLQAFDIFS-QYT-FQSTWKKKYLKRIQIGLDRQLQ

YLEQCLEEEEKNED--------MKEMEEDESL--------------E------LKRYFHR

ISS-FLKDKKYSHCAWEIVRVEIRRCFYY----FTAL--LRKK-------------

>Dano_13

MIQKCLWPACL------------------MDLFITGILS------LDCDLLNVHL-SRVT

WQNLRVLRSMSNSFPLKCLRETEAFEPQEIL-------------SN-T----Q------P

VRRD--IKEV-----F-Y----EMSTQAFNIFS-QYT-FNSTWEEKHLKQIQIGLDRQIE

YVEQCLEDEEKNED--------MKQMEEDEML--------------E------LRRYFNR

INK-FLKDKKHSHCAWEIVLVEIRRCFCY--FKFTAL--L----------------

>Oror_5

VIRKCMWPVCL------------------MGLFVTGILS------LDCNLLNVHL-RRVT

WKNLSLLRRMSKSFPIECLRESKAFEPQEIL-------------SH-T----Q------P

LTRD--IKEA-----F-Y----EMSRQAFHIFI-QDT-FKSTWEEKHLRQVQIGLDQQLQ

YLEQCLEEEE-NED--------MREVAEDERL--------------E------LRRYFNR

IDR-FLKDKKYSHCAWEIVRVEIRRCFYF--FKFTAL--LRRK-------------

>Loaf_16

VIRKYFWPICL------------------VGLFLTSVLS------QSCDLLYVHL-NRVT

WQNLKLLSHMSNPFPVECLKEKKAFEPQEIL-------------SH-T----Q------P

VKRH--IEEA-----F-Y----EISSQVFNIFS-QHA-CKSAWDEKHLKQIQIGLHQQVE

YLERCLEEEEKSED--------MKQMEE-KIL--------------K------LRRYFNR

LGN-FLKDKKYSQCAWEIVLVEIRRCVFYY-FKFTTL--LRKK-------------

>Ereu_11

LIRKCLWSSCL------------------VYLFLTGIHS------LDCSFLNIQL-RRVT

GQNARLMSSMKGPLRQECLKDINNFEPEEIF-------------LC-N----Q------S

KKWN--IKVN-----F-Y----EIYANAFRIFS-QYT-VKYSWEEECMQQILMELNLQLE

SLEQCLKEEK----------------ENDEIL--------------K------LKRYFFR

IQS-YLRDKKYSDCAWKIVFVEIGRCFYHS-LKLTRL--SRKK-------------

>IFNK_mouse

MTPKFLWLVAL------------------VALYIPPIQS------LNC----VYL-DDSI

LENVKLLGSTMTGFPLRCLKDITDFKPKEIL-------------PY-I----Q------H

MKRE--INAV-----S-Y----RISSLALTIFN-LKG-SIPPVTEEHWERIRSGLFKQVR

QAQECFMDEEKE----------------REHL--------------E------LGKYFFR

IKK-FLINKKYSFCAWKIVTVEIRRCFIIF-SKSRKL--LKMK-------------

>IFN1_anoli

-----LLPIAL------------------TMVLITEVSS------QDCGQLLARL-RQAN

KANLELLNSKMNSTPQQCIEGVFSFSLKNKL-------------TNRD----V------S

EEEN--AKVA-----I-Q----EVLQQTGHIFR-QNC-TEMLWDEDSLRAFHAGLDQQSE

NLKSCL----------------SASIQL-TSL--------------R------VKRYFRS

LND-FLKEKEYNRCAWEIIQIQVKQCFLWI-EKLIQE--IQSK---------MAH-

>Opha_2

-----FLQICL------------------VMF-FTNVSS------QHCDQLHSRL-QEDN

KGNLELLGSHMRATPLECIGDIADFS-EENV------------MSMNE----A------S

HEED--AKIA-----I-Q----EMLQQTDLIFK-KVH-AELFWDETSLRTFHTGLDQQIK

RLETCQ----------------NASLQL-TRL--------------R------VKRYFQG

LND-FLKDKQYSSCAWEIVQIQLRECFLLI-HQLIQR--IPTQ---------IKY-

>Vibe_2

------------------------------MF-FTKISS------QHCDQLHTRL-LEAN

KGNLELLGSHMRATPLQCIGDIVDFS-EEHL------------ISVDE----A------S

HEED--AKRA-----V-R----EMLQQTDLSFK-QAH-AELFWDENSLRQFHTGLDEQIK

KLETCQ----------------SASLQL-TRL--------------R------VKRYFQG

LNH-FLKEKKYSLCAWEIVQIQLRECFLLI-HQLIQR--IPIQ---------IMY-

>Pybi_2

-----LLQICL------------------AMF-FTKISS------QHCDQLHRRL-HKAS

KGNLKLLGSNIRATPLQCIGDIIDFS-EENL------------MSMDG----A------S

HEEN--AKIT-----I-Q----EMLQQIDLIFK-QVH-AELFWDENSLRQFHTGLYQQIK

ELEICQ----------------NASLQL-TRL--------------R------VKRYFQR

LSD-YLKDKKYSLCAWEIVQIQLRECFLLI-NELIQR--IPT-----------LY-

>Opha_6

-----CLFICL------------------GVF-FTEISP------QDCNQIRSRL-HEAN

LRNMNLPMRNMGSTPQQCIRDIIDFSLEENL------------TNMIN----E------L

QGET--AKVA-----I-K----ELLQQIDLIFK-ESH-SELAWDENSLREFHIGLHQEIK

NTKACW----------------NTSLQF-TRL--------------R------VKRYFQR

LRD-FLKNKEYNLCAWKIMQIQIRECFEWI-NHLNQR--IPSE---------T---

>Thsi_3

-----CLFICL------------------GI--FTEISS------QDCNQFRSRL-HEAN

LGNLNLLTRNMGSTPQQCIRDIIDFSLEENV------------MNMVN----E------L

QGEN--AKVA-----I-K----ELLQQIDLIFK-ESQ-SELAWDENSLREFHIGLDQEIK

KTAACW----------------NTSLKL-TRL--------------R------VKRYFQR

LRD-FLRNKDYNLCAWKIIQIQIRECFQWI-NQLNQR--IPNE---------T---

>Vibe_6

-----CLFICL------------------GIF-FTEISS------QDCNQLRSRL-HEAN

LGNLNLLTRNIGSTPQQCIRDIIDSSFEENL------------MNMVN----K------L

QGEN--AKVA-----I-K----ELLQQIDLIFK-ESH-SELVWDENSLREFHIELDQEIK

KAETCW----------------NTSLQF-TRL--------------R------VKRYFQR

LRH-FLKNKEYNLCAWKIIQIQVRECFEWI-NQLNQR--IPSE---------T---

>Opha_7

-----CLYICL------------------GIIFFGDISC------QNCNQLQRKL-LKAN

KDNSNLLSSNIRPTPLQCMRSFVELSLKKIM------------IDMND----E------C

QVDI--AKTA-----V-K----EILQQIDVIFR-QNH-TELVWHEGSLRDFHIGLDQQIK

MLETCG----------------NASLQL-TRL--------------R------IKRYFQR

LSD-FLKNKKYSLCAWEIVQIQIEACFQLI-NHYIQR--IRSK--------TMKK-

>IFN5_anoli

MAQQCLLAFCL------------------LMSF-REILS------QDCNDLRHEL-NGAN

KANLELLNVKMGSTPLQCVDDVINFSSKESL-------------PSIY----D------F

EEEN--ATVA-----I-D----EILQQISYLFN-QNH-TKLSWDENSIATFKLGVDNEIK

KLTPCL----------------SDSIDE-LRD--------------K------VRKYFER

INN-LLKEKEYNLCAWEIVQMEVRQCLIVV-DQLISR--IPKK---------KAV-

>Chmy_15

-----LPRVCL------------------VLLFFTEISS------RLCTMLHFQQ-KKMN

RESLEHL-KKMSGNPSQCINERAASKP-QDV-------------AQLP----V------S

QKEK--A--------------------IFSIFS-KNL-TQSAW---------NGLL----

------------------------------------------------------------

--------------------------------------------------------

>chpi_1

-------------------------------------------------MLHFQE-NKGN

KESVELL-KKMSENLSQFINEMKAFKP-QDV-------------VQLQ----L------S

QKEN--ADVA-----I-Q----EILQEIFTIFS-KNL-TQTAWDRSSIARFQNGLYQQIQ

PLEVCL----------------GA------------------------------------

--------------------------------------------------------

>Chpi_6

------------------------------ML-----------------MDVFQE-----

--------QTSSEKP-----------P-RDA-------------F-IP----V------I

KRAN--YE-W-----L-H----EILQQIFNIFS-KNL-TQSAWDGTSIVRFQNGLYQQIQ

RLEACL----------------RANLQL-TSR--------------R------VKKYFQG

IDA-FLKEKQYSLCAWEIIRMEISRSFVLI-DKLTRS--LSN--------------

>Almi_3

-----LLHICL------------------VLLFSTEISS------QHCDLLSFQQ-KKLN

KDSLELL-EKMGGNPFQCFSERTDFKS-QDV-------------LKLQ----L------S

QKEN--AKLA-----I-Q----NILQEIFTVFS-KNL-TQTAWDEISIITFQNKLHQQIE

RLEACL----------------GFKLVL-TKL--------------K------IKRYFQG

IYN-FLEEKQYSLCAWEIIRMEMTRGFLLV-DQLTKS--I----------------

>Crpo_3

-MKMMLLHICL------------------ILLFSTEISS------QHCDLLSFQQ-KKLN

KDSLELL-EKMGGNPFQCFNEGIDFKS-QDV-------------LKRQ----S------S

QKEN--VKLA-----I-Q----NILQEIFTVFS-KNL-TQTAWDTISIITFQNKLHQQIE

RLEACL----------------GFKLVL-TKL--------------K------VRRYFQG

IYN-FLEEKQYSLCAWEIIRMEMTRCFLFV-DQLTKS--L----------------

>Gavga_8

FLSSFLLHVCL------------------VLLFSTEISS------LHCGLLNFQQ-KKLN

KDSLELL-DNMGGNPSQCSNERIDFKP-QDV-------------LMLR----S------S

LKEN--AKMA-----I-Q----EILQEVFTVFS-KNL-TQTAWDEASIVVFQNGLHWQTE

RLEACL----------------DLKALL-TRL--------------K------LKRYFQG

IRN-FLEGK-----------------------------------------------

>Crpo_4

FLSSFLLHVCL------------------VLLFSTEISS------LHCGLLNFQQ-KKLN

KDSLELL-DNMGGNPSQCSSERTDFKP-QDI-------------LMLR----S------S

LKEN--AKMA-----I-Q----EILQEVFTVFS-KNL-TQTAWDEASIVAFQNGLHWQTE

RLEACL----------------DLKVLL-TRL--------------K------LKRYFQG

IRN-FLEGKQYSLCAWEIIRLEMPRCFLLL-DLLTKW--LKI--------------

>Gavga_6

---------------------------------------------------------KIE

Q-SLELL-DKIGRKPSQ-SNEKIDFKP-QDV-------------LVLG----S------T

LKEN--AQMA-----I-Q----EALQGVFTVFG-KNL-MQTAWDETFIVMFQNGLHWQIK

KLEACL----------------GLKVLL-TRV--------------K------LKNYFQG

ICN-FLEGKQYSLCAWEIILLEMHRYFLLL-DQLTKW--LK---------------

>Chmy_2

-----LLHICL------------------VLLFSIEISS------LDCNMLHFQQ-NKMN

MESLELL-SKMGGQPLQCLNENRNFRF-QKA-------------LRPR----E------S

QEKN--AKVV-----I-Q----EILQQIFNIFS-KNL-TQAAWDRSSVETLQKGLHQQTE

QLETCL----------------YS-LLF-PML--------------K------LKKYFQR

IRD-FLKEKQYSLCAWETIRLEMGRCFFFV-DQLIIR--LQN--------------

>Chpi_17

-----LLHICL------------------IMLFSTEISS------LDCTILHFQQ-NKMN

MESLELL-SKMGGQPLQCLNENRNFRL-QKA-------------LRPR----E------S

QEKN--AKMV-----I-Q----EILQQIFNIFS-KNL-TQAAWDRSSVETLQNGLHQQTE

KLETCL----------------HLYLLF-PML--------------K------LKKYFQR

IRD-FLKEKQYSLCAWETIRLEMGRCLLFV-DQLIKR--L----------------

>Chmy_1

-----FLHICL------------------VLLFSTKTSS------VDSNMLHFQQ-NKVN

QASLQLL-EKMGGQPVQCLNENSNFIS-QNV-------------LSSR----E------F

QKES--VIVA-----L-Q----EILQQIFNIFS-KSQ-LQTAWDRSSMDAFQNGLHHQIE

LLKTWF----------------NEY--F-TIL--------------K------VKKYFHV

IDN-FLKEKQYSLCASEIIREEMRRCFLFI-DQLTKR--LKN--------------

>Chpi_2

-----FLHICL------------------VLLF-TENSS------VDCNMLHFQQ-NKVN

QASLQLL-EKMGGQPVQCLNENSNFIS-QNV-------------LSSR----E------F

QKEN--AMVA-----I-Q----EILQQIFNIFS-KSH-IQTAWDRSSIVAFQNGLHQQIE

LLKTWF----------------DGY--F-TRL--------------K------VKKYFHV

VDN-FLKEKQYSLCAWEIIREEMRKCFLIM-DQLTKR--LKN--------------

>Pesi_2

-----FLHICL------------------VLHISTKISF------VDCNMFLFQQ-NKVN

QDSLKLL-EKMGGQPVQCLNEKSNFIS-QNL-------------FSST----E------F

QKEN--AMMV-----I-Q----EILQQSFTIFR-KIQ-IQTDWDRSSIAAFQNGLYQQIE

LLKTWF----------------DGY--L-TRL--------------K------VKKYFHV

IDT-FLEKRQYSRCACEIIREEMRRCFLFI-DQLTKR--LKN--------------

>Chpi_8

----------------------------------------------------------MN

SESLEHL-EKMGGNPFQCLNERTAFKP-RDI-------------LKIR----L------S

QQEN--AKVA-----I-Q----QILQELFHIFN-NNL-TQAAWNGTSIKEFQNGLHQQIE

KLETCL----------------SAYLLL-TSL--------------K------LKRYFQT

IDD-FLKEKQYSQCAWEIIRVEISRCFPIL-NILTKR--LQDE--E------LKY-

>Pesi_3

----------------------------------------------------------MN

SKSLEHL-EKMGGPPFQCLNERSAFKA-TDI-------------LKVR----L------A

QQEN--AKAA-----I-Q----QILQELFQIFS-KNL-THAAWDGTSIKEFQNGIHQQIE

KLEVCL----------------SAYLLH-TSL--------------K------LRRYFQT

VRH-FLKEKQYSRCAWEIIRLEVSRCFLVL-NILTKR--IEN--------------

>Chmy_4

-----LLQISL------------------VLLCTTKIST------LDCNTLPLLH-NKVI

QGNLHLL-NKMGQQPEQCQSEKMHFKF-EQF-------------LKLR------------

QKEN--AKVE-----I-Q----EILQQTFYVFT-KNL-TLAAWDGRALERFQNRLNQQIE

HLEACL----------------TEY--I-IRL--------------K------LKKYFQK

IDN-FLKDKQYSLCSWEIIRLEMRRCLQFI-DKVIRR--LRN--------------

>Pesi_1

-----LLQISF------------------MLLCTTNISA------LDCNILPLLH-NKMI

QGHLHVL-NKMGQQPEQCQSEKMHFQF-EKF-------------LKLR------------

KKEN--AKVA-----I-H----EILLQIFYIFT-KHL-TLVAWDGRSLERFQNGVNQQIE

HLDACL----------------TEY--I-IRL--------------K------LKKHFQK

LDN-FLKDKHYSLCSWEIIRLEIRRYLHFI-VKVTRR--LRN--------------

>Coli_2

-----LIQIGL------------------IVLCITIISS------HQCNHLPLQQ-RKAI

ENSLQLL-DKMGEKPQRCLREKMSFKF-KQV-------------LKPT------------

QKEA--VEVA-----I-E----EIFQHIFYIFS-KNL-TLAAWDGTALEKFQNGLYHQIE

QLEACV------------------Y--V-NRL--------------K------LKKYFQK

IDC-FLKDKQHNLCSWEISRAEMRRCLQLI-DKVIRK--LYKV-----------H-

>Apfo_3

-----LTQIGL------------------ILLCTTTISS------LQCNHLHLQQ-RKVI

ENSLQLL-DKMGEKPQQCLKEKMSFRF-EQV-------------LKPR------------

QKET--VKVV-----I-E----EIFQHIFYIFS-KNL-TLAAWDGTALEQFQNGLYQQIE

QLEACV------------------Y--V-NRL--------------K------LKKYFQK

IDC-FLKDKKHNLCSWEISRAEMRRCLQLI-DKVIRK--LNN--------------

>Fape_1

-----LIQIGL------------------ILLCTTTISS------LQCNHLPLQQ-GKVV

ENSLKLL-DKMGKKPQQCLREKMSFRF-EQV-------------LKPR------------

QKEN--VEVV-----V-E----EIFQHIFYIFS-KNL-TLAAWDGTALEQFQNGLHQQIE

QLEACV------------------Y--V-NRL--------------K------LKKYFQK

LDC-FLKDKKHDLCSWEISRAELRRCLQLI-DKVIRK--LNN--------------

>Tyal_2

-----LIQIGL------------------ILLCTTTISS------LQCSHLPLQQ-RKVI

KNSLQLL-DKMGKKPRQCLREKMSFRF-KQV-------------LNPR------------

QKET--VKVA-----I-E----EIFQNIFYIFS-KNL-TLAAWDGTALEQFQNGLYQQIE

QLEACV------------------Y--V-NRL--------------K------LKKYFQK

IDC-FLKDKQHNLCSWEISRAEMRRCLQLT-DKVIRK--LNN--------------

>Gaga_3

-----FIQIGF------------------ILLCTITISS------LTCNHLPLQQ-RRVI

ESSLQLL-DKMGRRPQQCLREKMSFRF-EQV-------------LKPR------------

QKET--VKVA-----I-E----EILQHIFYIFS-KNL-TLAAWDGAALEQFQNGLYQQIE

KLEACI------------------Y--V-NRL--------------K------LKKYFQK

IDS-FLKEKQHNLCSWEISRAEMRRCLQLI-DKVIRK--LYK--------------

>Stca_2

-----LLQIGL------------------ILSCTTNISS------LHCNHLSLQQ-SKVI

ESSLQLL-DKMGEKPQRCLRERMSFRF-EQV-------------LKPR------------

QKET--VKMA-----I-E----EILQHIFHIFS-KNL-TLAAWDGQALEQFQNGLYQQIE

QVEACV------------------Y--A-SRL--------------K------LKKYFQK

IDY-FLKDKQHSMCSWEISRAEMRRCLQFV-DKVIKR--LNN--------------

>Caan_3

-----LIPTGL------------------ILLCTTTISC------LWCNHLPLQQ-RKVI

QNSLQLL-DKMGNKPQQCLKEKMFFSF-EQV-------------LKPR------------

QKES--VKVA-----I-E----EIFQHIFYIFS-RNL-TLAAWDGAALEQFQNGLYQQIE

QLEVCV------------------S--V-NRL--------------K------LKKYFQK

IDC-FLRDKQHNLCSWEISRAEMRKCLQMI-DNVIWK--LNS--------------

>Almi_5

--------MFF------------------ILLCTMQIST------PDCNIPSLQQ-SKAI

QSSLHLL-DKIGQAPLQCRREHVLFKF-HNI-------------LKLS------------

QKDN--VKVA-----V-Q----ETLQSIFYMFS-KNL-TLAAWDGRSLESFQNGLYQQIE

QLEACS----------------IKY--A-NRL--------------K------LKKYFQR

IDN-FLKGKQYSLCSWEIIREEVRKCLQLI-EKGLEG--LENK---------IKND

>Gavga_2

--------MFF------------------ILLCTMQIST------LDCNIPSLQQ-SKAI

QSSLHLL-DKIGQAPLQCRREHVPFNF-RNI-------------LKLR------------

QKDN--VKVA-----V-Q----EMLQSIFYMFS-KNL-TLAAWDGRSLESFQNGLYQQIE

QLEACS----------------LKY--D-NRL--------------K------LKKYFQR

IDN-FLKDKQYSLCSWEIIREEVRTCLQLI-ETVTKA-------------------

>Crpo_7

MEVSGLLQMFF------------------ILLCTMQIST------LDCNIPPLQQ-SKAI

QSSLHLL-DKIGQAPLQCRHEHVPFNF-RNI-------------LKLR------------

QKDN--VKVA-----V-Q----EMLQSIFYTFS-KNL-TLAAWDGRSLESFQNGLYQQIE

KLEACS----------------LKY--D-NRL--------------K------LKKYFQR

IDN-FLKDKQYSLCSWEIIREEVRTCLQLI-ETVTKA-------------------

>Chmy_5

-------------------------------------MS------LDCNLLRHQQ-SKFN

WYSLQLL-QNMGGKPLECLEDKTAFQF-EKI-------------LKPK----F------L

QQ----AQMS-----V-H----EILEQLFGIFS-RNL-SQTGWERRKVERFLNGLALQTE

RLEECL----------------HT-------L--------------R------LKKYFQR

IQD-FLKEKKYSTCAWEIVREEGQRCFQYI-HKLTVR--MKN--------------

>Chpi_4

-----LWQICL------------------VLLFSAGVMS------LDCNLLRHQQ-SKFN

GYSLQLL-QNMGGNPLKCLEDKTAFQF-EKV-------------LKPK----F------Q

QH----AKMA-----I-H----EILQQLFGIFS-RNL-TQTGWERTKVGSFLNGLTLQTE

RLETCL----------------PT-------L--------------R------LKKYFQR

IQD-FLNEKKYSTCAWEIVREEGQRCFQYI-DKLTVR--MKK--------------

>Pesi_4

-----LWQLCQ------------------LLLFSAGVMS------LDCNLLHHQQ-SKFN

RYSLQLL-QKTGRSPLECLGDLTAFQF-EKV-------------LKHK----F------P

QH----AQMA-----A-H----EILQQLFGIFS-RNL-LQTRWEKGDVELFRNGLHLQTK

HLEKCL----------------ST-------L--------------R------LKRYFQR

IKD-FLEKKKYSTCAWETVRLEAQRCFLYM-DKLTVM--MKN--------------

>Almi_1

MMKNKLLHICL------------------VLLFSTEITS------MQCDMIHFQQ-KRLN

KDSLELL-EKMGGSPFQCSNEN--------------------------------------

--------------------------QIQDYPG-KNL-TQTTWDWSSIVTFQNGLHRQIQ

LLEACL----------------VATQLH-TRL--------------K------LNRYFQR

IHN-FLEEKHYNLCAWEIIRIEMPNCFLFV-DQLTKS--LKN--------------

>Almi_2

-----LLKFFL------------------VLLLFKVSSS------LHCNSLASNQ-NKVN

KDGLDFL-DKMRRNSPQCLSERLDLKT-KDI-------------FKIE----L------S

QKDN--AKAA-----I-Q----ELLKAIFYVLS-NNL-TQTTWQESSIEKFKNGLHWQIE

NLETCL----------------DASPLV-TRL--------------K------LKRYFQA

IDN-FLKEKQYSQCAWEIISVELSRCFQFI-DKLTIK--LSTS-------------

>Crpo_5

-----LLKFFL------------------VLLFFKVSSS------LHCSSLASNQ-NKVN

KDGLDFL-DKMRRNSPQCLSERLDLKT-KDI-------------FKIE----P------S

QKHN--AKAA-----I-Q----ELLKAIFYVFS-KNV-TQMTWQESSIEKFKNGLHWQIE

NLETCL----------------DASPLV-TRL--------------K------LKRYFQA

IDN-FLKEKQYSQCAWVIISVELSRCFQFI-DKLTKK--L----------------

>Gavga_7

-------------------------------------SS------LHCSSLASNQ-NKVN

KDGLDFL-DKMRRNSPQCLSERLDLKT-KDI-------------FKIE----L------S

QKHN--AKAA-----I-Q----ELLKAIFYVFS-KNL-TQTTWQESSIEKFKNGLHWQIE

NLETCL----------------DAGPLV-TRL--------------K------LKRYFQA

IDN-FLKEKQYSQCAWEIISVELSRCFQFI-DKLTKK--LRYC-------------

>Chmy_6

------------------------------------------------------------

----------------------------MAI-------------LKPR------------

EKVN--IVVT-----I-H----KILHETFNLFS-KNL--HAAWNTTCIEKFQNGLHWQIE

QLETCL----------------GANLQS-T-L--------------N------VKKYFQR

IKD-FLKEKHYSHCSWEQY-----FCLLSF-KKKSKN-------------------

>Almi_4

MEEPTFLHVCL------------------VLVFSIKISS------PDCS--RLQR-IKVN

-HSLYLL-CRMGGQPLSCLNDRTDFRI-REI-------------FIIR------------

KKEN--ALMI-----I-H----ELLHHIFQLFS-KNL-PQGAWNPSCIEKFQNGLHWQIE

QLEKCF----------------GGNLQN-NIL--------------K------AKKYFQR

ISH-FLNEKNYSRCSWETARMEMRRCFLFL-DHLLKN--LRN--------------

>Gavga_3

MEEPAFLHVCL------------------VLVFSIKISS------PDCS--RLRQ-IKVN

-QSLHLL-CRMGGEPLSCLNDRPHFRI-RQI-------------FTAR------------

NKEN--ALMI-----I-H----ELLHHIFQLFS-KNL-PQGLWNPSCIEKFQNGLHWQIE

QLQTCF----------------GGDLQN-NIL--------------K------VKKYFQR

ISH-FLNEKNYSRCSWETARMEMRICFLFL-DHLLKK--FRN--------------

>Crpo_1

MEEPTFLHVCL------------------VLVFSIKISS------PDCS--RLQQ-IKVN

-QSLHLL-CRMGGEPLSCLNDRTDFRI-REI-------------FTAR------------

KKED--ALMI-----I-H----ELLHHIFQLFS-KNL-PQGPWNPSCIEKFQNGLHWQIE

QLETCF----------------GGDLQN-NIL--------------K------VKKYFQR

ISH-FLNEKNYSRCSWETARMEMRRCFLFL-DHLLKK--FRK--------------

>Tyal_1

TDKTTLLRVCV------------------TLALYIKISH------PVC---LFQG-IKVN

YNNMNFL-WTMGGYSQQCLSEATDFRF-MEI-------------TKVT------------

QK-N--VTMI-----I-Y----EFLQQTFQLFS-KNL-PAGAWNTSKIQKFQNGIHQQIE

ELEVCL----------------LEILKS-TTF--------------S------VKKYFQR

ITD-FLKDKKYSHCSWEAVQMELRSCLIIF-DSLLKK--HTS--------------

>Apfo_2

-----LLRVCI------------------TLALYVKISH------PAC---LFQG-IRMN

YHNMNLL-CKMGGYSQQCPSETTDFRF-MEI-------------TKIT------------

QK-N--VTVI-----T-C----KFLQQIFQLFS-KNL-PVGAWNTSNIEKFQNGIHHQIE

ELETCL----------------SESLRS-TTL--------------S------MKKYFQR

ITN-FLKDKQXSHCSWEAVRMELRTCFIIF-DI-----------------------

>Stca_3

-----LLQVCI------------------TLALYTKISH------PVC---LFQG-SKVN

YQNMNFL-CKMGASPQQCLRERTDFKF-MEI-------------TKVR------------

QR-N--AIVM-----I-H----ELLRQIFHLFS-KNL-PESVWNASCIEKFQNGIHQQIE

ELETCL----------------VESLNS-TTL--------------R------VKKYFRR

ITS-FLENKQYSHCSWEAVRMEVRTCFIFI-DCLMRK--HMA--------------

>Almi_11

----------L------------------VNLYHAR---------------KQER-MKIN

K-------EKRSPHDISRKYKKIDFKP-QDV-------------LILH----S------T

LKEN--AKMA-----I-Q----EILQGVFTVFS-KNL-MQTAWDETSIVMFQNGLHWQIK

RLEACS----------------GWEVLL-TRL--------------K------LKSYFQG

I-------------------------------------------------------

>Almi_7

-----LWKICL------------------VALLSAHVAA------LDCSNFRDLQ-KVLN

RNSMQLL-GQVAGAPEECLEDRPTFRF-DKV-------------LRSK------------

APHN--AWMA-----T-Y----EILQKLFSLFK-RTL-PETAWDTRSVERLLNAVHLQIK

RLETCP------------------Y----KAT--------------G------LKKYFRK

IDD-FLRAKNYSKCAWEVVRIEAKTWFYYL-DKLKNR--LN---------------

>Gavga_1

-----LWQICL------------------AALLLAHVTA------LDCSNLKDLQ-EVLN

RNSVQLL-GQVAGAPEECLEDRPTFRF-EKV-------------LRSK------------

APHN--AWMA-----T-Y----EILQQLFSLFK-RNL-PETAWDTRSMERFLNAVHVQIK

RLETCP------------------Y----KAM--------------K------LKKYFRN

IHD-FLQEKNYSKCAWEVVRIEAKTWFYYL-DKFRNR--L----------------

>Crpo_2

-----LWQICL------------------AALLLARVTA------LDCSNFKDLQ-EVLN

RNSMQLL-GQVAGAPEECLEDRPTFRF-EKV-------------LRSK------------

APHN--AWMA-----S-Y----EILQQLFSLFK-GNL-PETAWDMHSVERFLNAVHVQIK

RLETCL------------------Y----KAM--------------K------LKKYFRN

IHN-FLREKNYSKCAWEVVRIEAKTWFYYL-DKFGNR--L----------------

>Chmy_14

-----MAYQVP------------------TQP---------------------GM-TSIS

KSAFSAL-QK----PSSC------------------------------------------

------SK-----------------------------------NGSSIKVFQNGLHQQIE

KLETCL----------------SACLLL-TSL--------------K------LKRYFQT

IDD-FLKEKQYSRCAG---RSSVRKYPDVF-SFLTY--------------------

>Chpi_15

-----LADESP------------------VTVLSVH---------------ASGQ-SSSG

RRLLAPL-MPLRSSPGSAW-----------------------------------------

RHQP--SKI---------------LQERFHIFN--NL-TQAPWNGTSIKEFQNGLHQQIE

KLETCL----------------SAYLLL-TSL--------------K------LKRYFQT

IDD-FLKEKQYSLYAWEIIRAEISRCLLIL-DIL----------------------

>Opha_5

-----TLKVCL------------------VLVLFAKSSV------FRCSIFHAQW-KQLI

QNNLPHL-CRTNEEPLQCIYELTDFNF-LEV-------------LEVT------------

NRDS--ADI------I-Y----ELLQQISYLLS-NA----HAWNSTCFENLKNGLHQQIK

NLETCL----------------NVNSYL-LTL--------------K------VKRYFQR

MNN-FLTVKQHSSCSWEMIHSEIKGCMLFI-THLLKK--L----------------

>Thsi_2

-----ILKVCL------------------VLVLLAKFLV------FRCSIFHAQW-KQLI

QNNLPHL-CRTNEEPLQCIYELTDFGF-LEV-------------LEVS------------

NRDN--AVI------I-Y----ELLQQISCLLS-NAP-SDHAWNSTCFENLKNALYEQMK

NLQACL----------------NAKSYL-LTL--------------K------VKRYFQR

MNN-FLTL------------------------------------------------

>Pybi_1

-----TLKICL------------------VLVLFAKLSV------FQCSIFHAQW-KQMI

QNNLHQL-CRANEEPLKCLPEVTDFRF-LEV-------------LKVS------------

NRGN--AII------I-Y----EILQQISCLLS-KGH-SYNVWNSTCFENLQNALHQQMK

TLATCL----------------NATSYF-LTL--------------K------VKKYFQR

MND-FLTVKQYSSCSWEMIHSEIKGCMLFI-IHLLNK--LKQ--------------

>Vibe_8

-----ILKFCL------------------VLVLFAKFSV------FRCSVFQAQW-MQLT

QNNLPHL-CR-NEIPPQCIYDLTDFRF-VEL-------------LQVN------------

NRDN--AVI------I-R----EFLNQIFYLLS-YAH-RYKVWNSTCFENVKIVLHQVIN

NLERCL----------------NANSYL-PKI--------------K------VKRYFKR

MIN-FLTLKQHSSCSWKIIHLEIEKCMMFI-TRLLEK--LKQ--------------

>IFN1_ONMY

--------------MYTMQ--SW-SC---IFL----IICSMQSVCHCCDWIRHHY-GHLS

AEYLPLLDQMGG----DITKQ----------N--AP----VLFPTSLYRHIDD------A

EFED--KVIF-----L-K----ETIYQITKLFDG--NMKSATWDKKNLDDFLNILERQLE

NLNSCV---------------------S-PAMKP------ERRLKR----------YFKK

LSK-VLRKMNYSAQAWELIGKETKRNLQRL-DILAAQ--MY---------------

>IFNA1_SASA

--------------MYTVQ--SW-TC---ICL----IICSMQSVCHCCDWIRHHY-GHLS

SEYLSLLDQMGG----DITKQ----------D--AP----VFFPTSLYRHIDD------A

EVED--QVRF-----L-K----ETIYQITKLFDG--NMKSVTWDKKKLDDFLNILERQLE

NLKSCV---------------------S-PAMKP------EKRLKR----------YFKK

LKN-VLRKMNYSAQAWELIRKETKRHLQRL-DILAAQ--MY---------------

>Eslu_3

-----------------MQ--SW-IF---LFL----ILCRTQSSCSCCDWIRDHY-GTLS

REYLSLLDEMGG----NITKQ----------D--VP----VFFPESLYRLMED------A

QYEV--QVRF-----L-N----ETIHEIIKLFDE--NMDAVTWEEKKLDDFLILLHRQFQ

KLKSCV---------------------S-PAKKA------EGRLES----------FFKK

LKK-VLKEMNYSAQAWELIRKETKYVLEKL-YLLVAT--MHR--------------

>IFN1_DARE

---------------------MW-TY---IFV----IYVILQSQSSACEWLG-RY-RIIT

TESLNLLKNMGG----KYA-D-------------LE----TPFPSRLYTLMDK------S

KVED--QVKF-----L-V----LTLDHIIHLMDAREHMNSVNWDQNTVEDFLNILHRKSS

DLKECV---------------------A-RYAKPAHKESYEIRIKR----------HFRT

LKK-ILKKKQYSAEAWEQIRRVVKSHLQRM-DIIASN--ARVN-------P-----

>IFN_CAAU

---------------MKTQ--MW-TY---MFV----MFLTLQGQCSACEWLG-RY-RMIS

NESLSLLKEMGG----KYP-E----------G--TK----VSFPGRLYNMIDN------A

KVED--QVKF-----L-V----LTLDHIIRLMDAREHMNSVQWNLQTVEHFLTVLNRQSS

DLKECV---------------------A-RY-QPSHKESYEKKINR----------HFKI

LKK-NLKKKEYSAQAWEQIRRAVKHHLQRM-DIIASN--RR---------------

>IFN_CTID

---------------MKTQ--MW-TY---MFV----MFLTLQGQCSACEWLG-RY-RMIS

NESLSLLKEMGG----KYP-E----------G--TK----VSFPGRLYNMIDN------A

KVED--QVKF-----L-V----LTLDHIIRLMDAREHMNSVQWNLQTVEHFLTVLNRQSS

DLKECV---------------------A-RY-QPSHKESYEKKINR----------HFKI

LKK-NLKKKEYSAQAWEQIRRAVKHHLQRM-DIIASN--RR---------------

>IFN_CYCA

--------------MNQTQ--MW-TC---IFV----IFLTLQSQCSACRWLG-RY-GTVS

ADSLNLLREMSG----QYP-E----------N--VK----MHFPGTLYNLIDK------A

EVED--QVRF-----L-V----LTLDHIINLMDASEHMNSAKWNLKKVEYFLEDLQRQSS

ELKECV---------------------A-QYQKPLQKESYEIRIKR----------HFRT

LKK-ILKKEKYSAQAWEQIRRAVRSHLQRM-DIIANN--AKKR-------V-----

>IFN2_ICPU

--------------MDIKL--SW-IC---LFL----LFFTVQERSEACNWMISQY-RAKN

DYCLSLLNEMGG----EIVPM----------T--GN----TSFPRRAYHEIEK------A

QAED--QVRF-----L-A----VATNEIIILFSAVSHVDDVKWDSRTLDNFLNILSRQLS

ELRNCT---------------------S-TYAERARRSSTEKKLRK----------HFKD

LRK-YLKNSNYSADSLEQIRSVVQRHLWRM-DTIAAI--VKQK-------LLRTN-

>IFN4_ICPU

--------------MDIKQ--SW-IC---LFL----LFFIVQERSEACNWMISQY-RAKN

NFCVSLLKEMGG----EIVQV--------------N----RPFPHKAYSEIDK------A

KAED--QVRF-----L-A----QATEQIISVFN-VSHVDEVKWDRSALDEFLNILNRQLT

ELTKCT---------------------S-TYAERAGHSPTERKLRK----------HFKK

LKK-FLNEANYSADSLERIRNVVQHHLWRM-DIIAAN--VKQK-------LLRTN-

>Anja_5

-------------------------------F----IVCSAQDFCDGCYWIQHGF-RRVS

GESLSLLSE---------------------------------------------------

MVDD--KVKF-----V-H----ASIDQIIKLFDE--NLDAVTWNRLKLEHFLIVLDRQSR

ELQKCV---------------------S-RCSII------RK------------------

--------------------------------------------------------

>IFND1_ONMY

----------MHR-T--KS--LL-IC---LFL----TLCN-G-LSVGCRWMDHKF-IQHS

ETLMNVLNIMGG----EFTTD----------S--VD----VPFPEDLYEQAEY------L

PTDD--TIWF-----I-L----QTLDKIAELFD-GEL-NSV-WDEKKVEIFLNVLTSQSD

GLQSCV---------------------R-AQKKN----------SKN------LQMYFKR

LNNHVLKRMAYSAHAWELVRKEVRTHLRRL-VLLGSA--TENR-------I-----

>IFND1_SASA

----------MHR-T--KS--LL-IC---LFL----TMCD-G-FSMGCRWMDHKF-IQHS

ETLMNLLNIMGG----EFTTD----------S--VD----VPFPEDLYKQAEY------L

PTDD--TIWF-----I-L----QTLDKIAELFD-GEL-DSV-WNEKKVEIFLSVLNSQSD

GLQSCV---------------------T-AQKKN----------SKN------LQMYFKR

LHNQVLKRMAYSAHAWELVRKEVRTHLMRL-VLLGSA--TENS-------I-----

>Eslu_2

--------------------------------------------------M-------HS

ASSLGFLDIMGE----DIPKD----------S--VK----IFFPEDLYKQADC------S

PADD--QIWF-----I-L----QTLDEITKLFS-DKC-YSV-WGEKTVDNFLGVLSSQVD

GLQSCI---------------------T-SQKKR----------SKN------LHKYFKR

LNNDILKSMEYSPHAWEMVRKEVRTHLKRL-TLLGSA--PDNK-------LVQQ--

>IFN_SPAU

--------------M--LNRIFF-VC---LSL----SLYSAG-SSLSCRWMDHKF-RQHS

KNSLALLDTMAN----NSTNT----------TEDAEVEDTVAFPNLLYRQASK------A

SAED--QLAF-----T-V----QILDETAALFE-EDH-SSASWEENTVENFVNVVTQQAD

ELRSCI---------------------G-SHKKK----------NKK------LHMYFQR

LSSHVLKRMGHSAEAWELIRGEVKVHLMRA-NQLVTSATRTN--------------

>IFN_DILA

--------------M--LNRIFF-VC---LSL----SLYSAG-SSLSCRWMDHKF-RQHS

KNSLALLDTMAN----NSTNT----------TEDAEVEDTVAFPNLLYRQASK------A

SAED--QLAF-----T-V----QILDETAALFE-EDH-SSASWEENTVEDFVNVVTQQAD

NLRSCI---------------------G-SHKT-----------NKK------LQMYFMK

LSSHVLKKMGHSAEAWELIRKEIKTHLMRA-DQLVSSLLTTN--------------

>IFN1_OPFA

--------------M--LSRTFL-VC---LFL----SLCSAG-SSLSCRWLDHKF-RQHS

ENSLDLLDTMVN----NSTNT----------TEDAGVKDTVAFPNELYSQASK------A

AAED--KLGF-----T-V----QVLEETAALFE-EDH-SSASWEENTVENFVNVVTQQAD

GLRSCI---------------------G-SHKKK----------NKK------LHMYFKR

LSSHVLEQMGHSAEAWELIRNEIQTHLMRA-DLLVSSLLTTN--------------

>IFN_PAOL

--------------M--LNRIFF-VC---LCL----CLYSAG-SAMSCRWMDHKF-RQYS

KNSLDLLDMMAH----NSINT----------TEDVEVEETVALPEHLYSQVSN------A

SAED--RLGF-----T-V----QVLKEVAALFE-EDS-SFASWEEKQMDDFLNIVTQQAD

GLRSCI---------------------V-SHSHK----------NKK------LHMYFKR

LSRHVLKQMDYSVESWELIRKEIKNHLMRS-DVLISSLLTIN--------------

>IFN2_ORNI

--------------M--MNRILF-AC---LFL----GLFTVG-SSLSCKWMDDKF-KQHN

EETLNLLDTMGN----NSTNT----------T---EVEDTVAFPNHLYRQASK------A

SAED--KLAF-----T-V----QVLEEVAALFE-EDH-SSASWEDSTVRNFLNIVNKQAE

ELHSCI---------------------G-SHSHK----------KKK------TEMYFKR

LSDDVLKKKGHSAEAWEVIRKETKAHLMRL--TLIKSRGTTQS-------L-----

>IFN_ORLA

--------------M--LHRLVF-AC---ALV----SLAGAG-FSLRCRWLDHKF-KQFS

DTSLDLLEKMVN----NATNS----------TEGDATEDIVDFPHHLYRQASK------E

SAEN--QVAF-----T-V----QVLKEVSALFE-EDS-SSASWQQITVEKFLGVVNRQAD

ELHSCV-----------------------SESKK----------NRK------LRMYFKR

LLDHILKKQGYSAEAWETIRKETKAHLLRA-QRLLSPLISSK--------------

>IFN1_ORNI

--------------M--ISRIFI-AC---LFL----GMYSTG-SSLSCKWI-----VKHP

GNTLALLCIMFN----VITNT----------TKDAEIEHNVAFPNRLYRQTSK------A

TAED--KLAF-----T-V----QILKELLALFE-EDH-SSASWEENTVENFLNIVDKQTE

ELHSCI---------------------G-SHSNT----------QKR------REKYFKR

LLNKILKKNGYSAEAWEKIRNITQAHLRQC--EFLISLRTAH--------------

>IFN_TARU

--------------M--LP--LL-VC---LSL----CVYSQG-SPLGCRWLDDKF-RQYS

HKSLELLDTMVN----NSTNS----------S--VEPEEMVIFPQELYRQTFN------A

SAED--KLAL-----A-A----QIMNETVALLM-EDH-SGASWDEKQVENLVNVLTQQAD

NLQACM---------------------V-SPGHK----------SEE------VERYFNR

LSNHILKKMDYSAAAWELIREEIETLLMQT-HLLVSTLLSTP--------------

>IFN_TENI

--------------M--LT--VL-LC---LSL----CVCSQG-SPLGCRWLEEKF-TQYS

SLSLSLLDNMKS----NSTNS----------S--LEAEDTAIFPEVLYRQTFN------A

SAED--RLAF-----A-A----QILNETAALFE-EDY-SGASWEEKSVENFVNILTQQAD

NLGSCV---------------------A-SPGQS----------SKE------LHKYFTR

ISTHILRKTDHSAGAWELVREKIRSLLMRA-HLL----LTTH--------------

>IFN4_DARE

--------------M--KV--FA-AA---QFC----VLLSVG-FSLGCRWVKHRL-QHHH

GVSLDLLRKMGE----KVHDD----------N-----EDLNPIPYDLINNHRM------A

EPEK--QIQF-----V-I----QALVEITALFD-DAL---VPWDAKKMDDFLNIMHEEID

GLRSCG---------------------S-YKMKR----------NKK------LHLYFNR

-----LRRMTDGGRSWEMVRKRVIS-LMNQ-LHSFSF--HTH--------V-----

>IFNa1_gaac

--------------M--TSWTSM-LV---LLT----LLCSAGTPGLCCDWL-QHY-GHLS

NVSLTLVQTMGN----QLTDE----------E--SP----VSFPYRLYERIMN------D

KEDN--QLVF-----I-R----DSLELMAKLYR-HDNRSSVTWDANKMERFLMIIHRQIH

GLNLCV---------------------S-T--Q----------ITRR------LRRYYRR

LEKKTLYSTGGSPASWELIRKESKLHLDQL-NQLWGFMV-----------------

>Eslu_4

--------------MYSIR--LC-MS---LVL----MICSCN-ETMGCTWMRSMF-KSFI

SKSITVLQEKD-------DGE-------------PL----ISLPNKLYRQFDD------L

KADD--QIVF-----I-S----RTLKAIMHLYSSGKY-ES-TLETERIDTFIHYLSRQTM

ELDQCI---------------------K---AMNPTLSKSVKRANKK------MNSHFKF

LKN-YLKGEEFNGKAWIEIKRVVLAHLRRI-V--LT--------------------

>Opha_1

MISGCFQHIVL------------------FLLLSSGIRS------LDCNHI-VKQ-QSRT

VNIMKLLESMG---PWQCFNKIQDFAP-NDTG---------------------------S

IKED--ARAT-----F-G----LMLEQINRMFW-QNF-TKAEWNVTVTEHLQTSLDQQLV

QWEKCV----AEGK------KATK---DRIKL--------------K------LRKYFLR

LDT-FLKDEEYSSCAWEAVRHEIMGIQVFL-DQLLRT--LQR--------------

>Thsi_6

MISGCFQYIVL------------------LLLLSSGIRS------LNCNHI-VKQ-KDET

MNTIKLLESMG---PWQCFNQIQDFAP-NDTG---------------------------S

IKED--ARAT-----V-G----LMLEQINRIFS-QNF-TKAEWNMTITEHFQISLDQQLV

QWEKCV----AEGK------KATK---DRTKL--------------K------LRKYFLR

LDR-FLKDEEYSSCAWEAVRHEIMGIQFFL-DQLLRT--LQH--------------

>Vibe_7

MISGCFQYVVL------------------LFLLSSGVRS------LDCNHI-VKQ-KGAT

VNIMKLLEAMG---PLECFHKIPDFAP-NNIG---------------------------S

IKED--AKAT-----I-G----LMLEQISRIFS-QNF-TQTEWNMTVAEHFQIALDQQIV

QWEKCV----TEGN------KATK---ERTKL--------------K------LRSYFLR

LDT-FLKDEEYSSCAWEAVRQEIKGIPLFL-DRLLRT--LQS--------------

>Pybi_3

MVLRCLQHIVL------------------LLLFSSGIVS------LDCNHI-VKQ-KGAT

ADIRKLLEAMG---SLECFHKIPDFNP-KNIG---------------------------S

IKED--ARAT-----V-G----LMLEHIQRIFW-HNF-TKAEWNMTVTELFQIVLDQQVV

QWETCV----TVGE------KATK---DRTKL--------------K------LKKYFLR

LDT-FLKDEEYSPCAWDVVRQEVLGIHFFL-DQLLRT--LQN--------------

>IFN4_cami

--------MSLP-----------------VLLLCSPT-SGHG---SDC--M---Q-RRYW

RDLLESLN------PVACRGE--DVRE-PVML--------------------N------P

VKKD--KAAV-----T-L----QVLEEILRLFR-K---STVPWTNSK---FLNQVYQVIY

ELQNCM------TS--------S-----DLPVRS------------T------IKARFAN

LEG-FLNEKSI--CAWEIVHLETRKILQ----EVSRN--HARK-------------

>IFN5_cami

--------MSLP-----------------VLLLYSPT-SGHG---SDC--V---Q-RRYW

RDLLESLN------PEECRNE--DINP-APIS--------------------K------L

AKQN--KAAV-----I-V----QVLEEVLKLFS-K---PDAPWSNSKALTFLNKVSQILS

DLQSCM------QS--------P-----DSRVRS------------T------VIERFAK

LER-FLKAKSV--CAWEIVHAETRKIFQ----QVEQR--HARK-------------

>IFN9_anoli

--------MA-----------------------GQQ--S-------------SQ------

----------------ECLED--DFGPRKIL---------------------K-------

SQED--AKMA-----I-G----LILQQIQIVFQ-LNF-TQAQWSGKVTDLLSRALDQQHM

QWRRC-----A-----------TA---EAAKLR-------------S------LKRYFRK

LHT-FLRGRQYSFCAWKMVRYELLVIYPIL-NELMR---LEK--------------

>IFNB4_ONMY

--------MAVLK---------W-LSICLTLF----CQGTA--VSKPCRWTQFRL-GKLN

DVSIDLLSDMGGIFPLMCAEE----------N--VE----QMFPEDLY----K------N

TEGE--DVSV-----V-A---LEAMRYVDQLYN-NSL-TSVTWNKIKLNMFQNVIYRQVQ

NLELCV--------VGGVWE-------S-S----------------GDGGSVTLKTYFNK

LNT-VLKEKEHSACAWEIVRKEI---RENLKKFI-------DS-------RVKP--

>IFNB1_SASA

--------MAVLK---------W-LSICLTLF----CQGTV--ASKPCRWTQFRL-GKLN

DVSIGLLSDMGGLFPLMCAEE----------N--VE----QMFPEDLY----K------N

TEGE--DVSV-----V-A---LEAMRYVEQLYN-NSL-TSATWSKTKLNMFQNVIYRQVQ

NLELCV--------VGGVWE-------S-S----------------GDGWSVTLKTYFNK

LNT-VLKEKEYSACAWEIVRKEI---RENLKKFI-------DS-------RVKL--

>IFNC1_ONMY

--------MILQTV--------W-MS---AFL----CLAQV--CSVP---MPCQLQGQLV

RITHNLLRDMGGNFPLECLQE----------N--VF----VAFPATAF----T------I

SGES--QLSS-----SGAMAIYETLKNIDTLFG-ADD-LPTKWDQQKLENFQNIVYRQIE

ESK-CM--------MGSVDT-------S-DYLIR----------TEG------LKTYFGN

IAA-VLKEKNFSYCAWEVVRKEL---LYSL-QFILEH--NSDS-------LLRT--

>IFNC1_SASA

--------MALQTI--------W-MS---AFL----CVAHV--CSMP---MPCQLQGQLV

RITHNLLRDMGGNFPLECLQE----------N--VF----MAFPATAF----A------S

SGAP--QLGS-----SGAKAIYETLKNIDILFE-ADD-LPTQWDQQKLKNFQNIVYRQIE

ESK-CM--------MGSVDT-------S-DYLIR----------TEG------LNTYFGN

IAA-VLKEKNFSYCAWEVVRKEL---LYTL-QFILEH--NSDS-------LLRT--

>Eslu_1

--------MALTTI--------W-VS---ALL----CFVHV--WSMP---MTCHIQETLM

ESAKDLLRDMGGHFPLECLQG----------K--VN----ITFPAPAF----A------T

SSTP--TLSG-----SGAKAIYETLKNIELLFG-AED-LPTKWDQQKLDNFQNIIYTQID

HSK-CV--------SGSVET-------S-DYPVR----------AAA------LKTYFGN

IEA-ALKEKKFSYCAWEVVRKQV---LETL-IFILTK--NSDC-------LLRT--

>Anja_11

--------MAF-----------WFIF---AFL------AQA--WSMP---QTCQLDGNLI

RTSHNLLKDAGGHFPLQCIKE----------N--VL----IMFPSSAF----E------S

NGTI--EQET-----GVRMAIYETLRSLSLVLE-DGD-LPTKWDEKIMDDFQNIVYRQVD

----------------S-------------------------------------------

-------KCSFSFCAWEIVRKEL---VRTL-HFILDH--RSDM-------LCRL--

>clha_a13

--------MVLQSF--------W-VT---IIL----CFVHV--STVP---ITCGLQRRLV

EKSHSLLESMSGLFPVECLEH----------N--LP----IAFPSSAF----M------T

SEAA--E-SA-----GAEKVAYETLKLIDTLFA-NDS-MPTSWNN--LEDFQEIIYRQIE

ESE-CI--------M-TQSK-------D-DFPTR----------NAA------LKTYFDK

IAT-ILKEKESSDCAWEVVRKEI---LYTL-KFIL-Q--SSNY-------LI----

>IFN3_DARE

--------MDLHRV--------W-LC---TFF----CFVQV--WSLP---TNCNLQKNLM

KRTYTLLET-AGLFPMQCLDD----------R--VS----IPFPQNVF----E------Y

NE-D--QVTG-----V-EKAVYQTLQNIDALFE-NFG-DPDQWDAEKLDDFRGIVYRQIK

YST-CI--------MNKTEV-------A-DFPSR----------EAS------LKVYFET

ISS-TLKEKNSSYCAWEIVRSEI---LRTL-EFILKN--NSDI-------ML----

>IFN2_DARE

--------MEFWQV--------F-LC---PAL----FFAHI--TSKP---TNCFMRRKHV

KTAYSLLESMGGLFPRECLKE----------N--VR----ITFPKYAL----Q------S

NNSN--QKTG-----V-AKAVYKIMDHIDFLFA-NDS-YPEAWNKRKVDNFQNIVYRLTK

ENQ-CI--------MRMRAT-------V-DFPAR----------DDA------LKSYFNK

LAT-LLRNKDNSFCAWEVVRHEL---LGVL----------SDI-------IL----

>Anja_10

------------------------------------------------------------

------------------------------------------------------------

-------QSD-----IIRTVIYETLYSINSLFE-NDD-FPTDWDEIKLQDFQNIIYRQVD

KST-CV--------SS-------------------------------------LSPEM--

--------------------------------------------------------

>Leoc_1

---------------------------------------------MP---TRCAFREHLI

EVSLNLLKDMGGHFPRECIKD----------N--VL----ITFPANVL----F------S

SFAQ--TQNE-----NIQPVVYETLRSVNTLFQ-SEG-RPSTWDQRKLEDFQSVVFRQVS

DFKKCA--------LRRRET------------------------SSANNSTVQLKTYFKK

MGR-FLEEKNYSSC------------------------------------------

>Leoc_2

--------MALEGS--------W-LC---VVF----CLSQA--WAMP---TRCAFREHLI

EVSLNLLKDMGDGFPKECLSD----------N--VV----IAFPAEAY------------

EFAE--TQKE-----DFEIAIYKTLNSTDALFE-NEG-RPTSWGQRAVDEFQNLVFRQVQ

DFNTCV--------PGDVET-------TGN--------------SSAAYRITLLKTYFQK

MEN-VLQEKNYSSCAWEIIRKEL---LGIL-QVILDK--NAEI-------VV----

>Scma_1

--------MTP-------------LSVLLLQL----CSFQV--VAMP----TCKLQANVV

LKTHNLLRDLGAAFPVHCLQY----------N--VN----ISFPDSAF----P------D

ATAH--PQCR-----RALWVVYESLRGMQLILE-QND-SPVTWDEGILDNFQNLQHRLLE

DGS-CL--------ST---K-------GPDV----------------------FSSYFSN

VTD-VLQQQDSSVCGWMALRRDV---LSVLKTALREH--NSCF-------T-----

>anja_f

----------------------------------------G--KRLP---KDCQQEAMRI

NISHDERQKL-----LDLKEE----------N--IS------------------------

-------------------TLQQVFNLTWRIFR-KNM-TAVNWNMSKLKSFRKLLKRQSR

TFSTCA--------RL---T-------SSSRPAK----------NDG------KRVYFRK

LSR-FLSTEKYSRCAWEIVRAEI---LTLLHVPRIAH--NSGH-------KENAP-

>IFN1_frog

--------MS--------------VS---VLL----LIT------LGSSGQPTKG-KDVY

RTQLNIN------------REVRTLLGN--M-------------GAIP----Y------S

ECEDNWRLQI-------------TIHQFSVIFT-DNL-A----NSVEMSKMQTLLYWY--

----------------------STSLKE-LTLKE----------TKK------IRRYFRK

MLK-YLMKKGYSRCAWASVRDEMEKVLLLV-TWHTDI--LLKK-------HLRGHV

>IFNF2_ONMY

--------MATLN----VS--FV-VH---LLC----VIV------LKCSDQKEQM-YNLS

QTRQTLNDLAMERRPRGCIPEAEMIMVQ--R-------------PTLS----K------E

EVEKVWTLRL-------------AFQLASELFQ-QNL-TLVKWNSIKLRDLQDLLARQ--

----------------------YMTVRD-MRLRQ----------NLP------IKNYFKQ

LDD-FLSRESFSLCSWEVVRTEMGSIL-------RDF--YKKS-------KMRKHV

>IFNF1_ONMY

--------MATLN----VS--FV-VH---LLC----IIV------FKCSAPKVQK-YYLS

QTHQTLNDLAEERLPRGCIPEAERLRVQ--R-------------PALP----I------E

EGEKVWTLRL-------------AFQLASELFQ-QNL-TLVKWNSVKLRDLQDLLARQ--

----------------------NMTVRD-MSVHL----------NLP------IKNYFKE

LED-FLSHERFSACSWELVRAEMGSII-------SQA--IRNA-------K--KHV

>eslu_f

--------MAAHN----VT--FV-VH---LLF----ALA------LTCCD---ET-YYIF

KTRQVVNDLAMGRKPV-CVQEAARIRVH--R-------------PTLS----L------E

VGERFWTLSL-------------VFHLACELFQ-RNL-TLVKWNVNQLRELQELLARQ--

----------------------NRTVKD-IRLGQ----------SLP------NKKYFKQ

LDD-FLSRETFSLCAWEVVRFEMGRIL-------RDF--HKKS-------N-SKKT

>Eslu_6

--------MS--------------------------------------------------

-------------------ED---------------------------------------

------VAVV-----A-L----KTFGYVEQLF-DSKL-TPSTWNNGTFHLFKNCIFRQIQ

GLQEC------------------------------------------------V--NLRP

I-------------------------------------------------------

>Anja_7

---------V--------------------------------------------------

-------------------EE---------------------------------------

------RIIF-----I-H----EVINNIKDLYIKGKY-DTVTWDPKKLQMFQLNLHRQAS

ELKEC------------------------------------------------VRPNFAP

PQN-AGHDKEYR--------------------------------------------

>Opha_4

MAATSMGLLCL------------------VLLLAVPA-LG-----LHCNLLKWQQ-QRLN

QQSVELLKGMK---SPVCL--RKEVTP-QIL---------------------R------I

QRPRA-AKAI-----L------EMLHGFLHLFK-EDH---VAWDATLRKRFLPSFSAPV-

----CI----------------SI------------------------------------

---------------------QVVLDLQWF-T------------------------

>Thsi_4

MPSKRMGLLCL------------------VLLLAAPT-WG-----SNCNLLKLHQ-RRLN

RQSVELLRRVK---PAECL--RKVFSP-KIL---------------------G------I

REPRE-AKAV-----L------EVLQGFLHILK-DEH---VAWKATLQKRFLPMLHAQIQ

RIQGCL----------------GE-GRKEEKL--------------Q------LKKYFRS

IGN-FLEENGLDSCTREFVRHEIQLDFIYL-DRLTER--ME---------------

>Pybi_6

MSATPTELLCL------------------VLLLSAQV-TA-----LNCNFLKLQQ-QRFN

RHSVELLKGMS---PQECL--RKTSNP-TVL---------------------E------I

HQPQI-AKTI-----L------EMLHGFLNILS-DDC---NAWEAKLRNR----------

---KCV----------------SA------------------------------------

--------------TVQFPLTELRCPFTYF-QVEAEE--ND---------------

1. **IFN1 CHOM with IFN2 as outgroup:**

>Xetr_4

--------MLL------------------SLTSIVH--S------QSCKWLHPKQ-EYLN

TQILKAFNEMMPLK-------------------------------ETE----E------I

SQVE--AGAL-----A-L----NEYM---------KH-HESMGCKQQAERFQQLLYYQIH

QLEACV----------------TEENDL-LKE--------------S------ISEEFNL

LET-MVLEKN-------------SACVLRR-QRLLQR--PQ---------------

>Napa_2

---------------------------------MVS--A------QTCKWLHRNQ-EAWT

RQILHNFNQMVPAE-------------------------------KTG----Q------P

TQAE--SAAI-----A------DEHI------------------------------YQLA

RPSRCC----------------TAEGKY-VSY--------------T------VNQGREA

VKP-VLA--------------------VSR-YILLVK--PQL--------------

>IFN1_coela

--------MTL------KM--LLAFC---LLL----LVSNGT-FCQDCKKWVKQ-----Y

NKGLQYLEAMGGEFPLKCLAQ-----------------------LDVP----RKVVLRHS

KGER--RIEL-----V-H----DTLEHISKTYS-NN--TSTKWDEENLKKFQNVIHLESE

ELRACL--------------QERVSNKN-TQWRK----------KMT------LSRHFKE

TGN-FLQRQNYSSCAWETVRAITRMILQLI-Q------------------------

>IFN2_coela

--------MAL------KT--FVALC---LLL----FVPIGI-FCQECEELNSQQ-RLRI

RESLQELEGVGGKFPSQCLAQ-----------------------FNLH----KKVLLKHS

KGER--RITL-----V-Y----EILQQINRIYR-KN--PSATWDQNKLERFQNVLHSQTE

ELWKCL--------------EKKMSNMN-SQWNN----------AMK------LSKNFKE

MEK-FLKHQNYSSCAWELVRTITRRVLQQV-ER---------K-------A-----

>IFN6_cami

--------MAV------HY--QCGLS----LF----AMLCVS-LTLGCSTLRLQK--ILI

ATTLNTLDEMGGHVPRHCVAVGAELRIA--S-------------PDLR----L--LLQPL

QNND--RILL-----L-H----KTFQHLNKIFH-KNM-KSVTWDLTQVNHFRELLVTQRD

VVKDCI--------------QDSAS--D-SMLSA----------LST------IHTYFRK

LKK-FLKQQRYSACAWEVIRMETRARLQQI-LILTAR--MTKG-------N-----

>IFN1_cami

--------MAV------HY--QCGLS----LF----AMLCVS-LTLGCSTLRLQK--ILI

ATTLNTLDEMGGHVPRHCVAVGAEQGIA--S-------------PDLR----L--LLQPL

QNND--RILL-----L-H----KTFQHLNKIFH-KNM-KSVTWDLTQVNHFRELLVTQRE

VVKDCI--------------QDSAS--D-SMLSA----------LST------IHTYFRK

LKK-FLKQQRYSACAWEVIRMETRARLQQI-LILTAR--MTKG-------N-----

>IFN1_sqac

--------MVF------PS--VWRLW---ILL----VLLPGT-LSQDCQRLQLLD--NIN

NQALDALREMGGPIPLHCKTERTSLRAK--S-------------LDLH----Q--LSKRL

QTPD--RIQI-----V-H----QTLRHLTKIYS-MNL-GSVTWPRDKVENFRLLLDRQLG

ELEECV--------------RKPVP--E-SRPRR----------NAP------IHKYFRK

VEK-FLKQKRFSDCAWEIIRAETRARLQQI-LFITAK--IRRR-------S-----

>IFN2_sqac

--------MVF------PS--VWRLW---ILL----VLLPGT-LSQDCQRLQLLD--NIN

NQALDALREMGGPIPLQCKTERTSLRTK--S-------------LDLH----Q--LSKRL

QTPD--RIQI-----V-H----QTLRHLTKIYS-MNL-GSATWPRDKVENFRLLLDRQLG

ELEECV--------------RKPVP--E-SRPRR----------NAS------IHKYFRK

VEK-FLKQKRFSDCAWEIIRAETRARLQQI-LFITAK--IRRR-------S-----

>Leer_25

------------------------------------------------------------

------------------------------------------------------------

------TLLV-----V-D----QMLRQFRKIYS-MNL-ASVTWLQDKVENFRLLLDRQIR

ELENCV--------------RNTGS--E-TRPRR----------SAA------VHNYFRK

LGK-FLKRK-----------------------------------------------

>Leer_37

------------------------------------------------------------

------------------------------------------------------------

-TQE--RIHT-----A-H----QTLQQINNVYS-MNL-DSITWAQHKVENLRLLLDRQLR

TLEECV--------------KKPGS--K-STSKR----------NTR------ISNYFRK

LRK-FLKRNRFSDCAWEITRTE----------------------------------

>Scca_1

-------------------------------------VISGE-IVWGFNEILFYS--S--

---------QGGSFPRHCIKHRHALKTK--P-------------LNLV----K--LSKGL

EKED--QIQI-----L-H----QTLRHISKIYS-MNL-GSVTWDRDTVENLRLLLDRQLS

ELE---------------------------------------------------------

--------------------------------------------------------

>Leer_3

------------------------------------------------------------

------------------------------------------------------------

-DMD--TAVV-----V-Y----QVVSQFKEIYH-MDR-TSVTWPQDVMKRFGIKLDAQSS

ILENCV--------------RNAGS--D-AQTQK----------KAT------IQEYFKK

LSE-FLNRERFSACAWE---------------------------------------

>Leer_4

------------------------------------------------------------

------------------------------------------------------------

-APD--LAGI-----V-Y----QVVTQYNRIYN-MDR-TSVTWPQIIMTSLGFHLDVQTT

VLEDCL--------------THRGA--M-AQRQN----------RET------IHDYFRG

LSE-FLNRERFSACAWEAIREEMILWYQQV-FKFF---------------------

>IFN2_cami

--------MPL------RC--VWKLC---LCL----ALLATQ-TPSLACNLPLHN--LMC

QRSLNLLLWMRDSIPFHCVREMGST---------VDLR------LNLR----N--VTGPL

QPGD--RMQV-----Y-L----QTLHHLNEIYS-NNV-TS-TWDQEKILGFRFVLDEQQM

EMEKCA--------------KEPAS--D-AMVHT----------TSA------IRTYFTK

LGR-FLRQKRFSACAWEVIRAKTSRRLQEM-LTLAMK--EAKT-------R-----

>IFN3_coela

--------MAL------KC--LWTMF---LLL----LDFPVA-FPEQCNWVHLHQ-KY-S

TSKLPLLDEMGAGFSESCMIE-----------------------IVKD----KKLTLKFP

NSDH--MIETDIMPTV-C----EILNFIGNIYN-KNL-QLVPWDKKKIEHFQTVLYHEVE

EIKKCL--------------PGEKTNAN-SHSNS----------NMK------LQDYFST

LEN-FLEQKEYSPCAWEVVRAHIRTLLQFT-DRLTTV--ITKN-------E-----

>IFN4_coela

--------MAL------KC--LWTMF---LLL----LDFPVA-FPEQCNWVHLHQ-KY-S

ISKLQLLDEMGAGFPPHCINE-----------------------KGVD----EKVTLKFP

KQDH--MIQMEIMPTV-C----EVLNFTGNIYN-KNL-QFVPWNKRKVQHFQTVLHHEVE

ELKKCL--------------PEEKTNAN-SHSNS----------NMK------LQDYFSK

LEN-FLEQKEYSLCAWEIVRVHIRKLLQLT-DRLTTA--IRKT-------E-----

>anja_f

----------------------------------------G--KRLP---KDCQQEAMRI

NISHDERQKL-----LDLKEE----------N--IS------------------------

-------------------TLQQVFNLTWRIFR-KNM-TAVNWNMSKLKSFRKLLKRQSR

TFSTCA--------RL---T-------SSSRPAK----------NDG------KRVYFRK

LSR-FLSTEKYSRCAWEIVRAEI---LTLLHVPRIAH--NSGH-------KENAP-

>IFN5_coela

--------MAQ------KF--QIISL---ILF----IVSQVR-ADDQCTWSTAQQ-MHLN

KRNLNLTDDMGKFSPAECTTE-----------------------IQEV----QKMRLRFP

ENE-------DVIFIV-Y----TTLRHISKIYS-KNL-QPVSWNKTVLHEFQAAVHSQVE

ELEKCL--------------MEKMVDHY-LERKV----------ELK------LRNYFKL

LEK-MLAEKENNQCAWRFIRAQVRKFLYRI-DQLTAW--IGKM-------KNQSS-

>IFNB4_ONMY

--------MAVLK---------W-LSICLTLF----CQGTA--VSKPCRWTQFRL-GKLN

DVSIDLLSDMGGIFPLMCAEE----------N--VE----QMFPEDLY----K------N

TEGE--DVSV-----V-A---LEAMRYVDQLYN-NSL-TSVTWNKIKLNMFQNVIYRQVQ

NLELCV--------VGGVWE-------S-S----------------GDGGSVTLKTYFNK

LNT-VLKEKEHSACAWEIVRKEI---RENLKKFI-------DS-------RVKP--

>IFNB1_SASA

--------MAVLK---------W-LSICLTLF----CQGTV--ASKPCRWTQFRL-GKLN

DVSIGLLSDMGGLFPLMCAEE----------N--VE----QMFPEDLY----K------N

TEGE--DVSV-----V-A---LEAMRYVEQLYN-NSL-TSATWSKTKLNMFQNVIYRQVQ

NLELCV--------VGGVWE-------S-S----------------GDGWSVTLKTYFNK

LNT-VLKEKEYSACAWEIVRKEI---RENLKKFI-------DS-------RVKL--

>Eslu_6

--------MS--------------------------------------------------

-------------------ED---------------------------------------

------VAVV-----A-L----KTFGYVEQLF-DSKL-TPSTWNNGTFHLFKNCIFRQIQ

GLQEC------------------------------------------------V--NLRP

I-------------------------------------------------------

>IFNC1_ONMY

--------MILQTV--------W-MS---AFL----CLAQV--CSVP---MPCQLQGQLV

RITHNLLRDMGGNFPLECLQE----------N--VF----VAFPATAF----T------I

SGES--QLSS-----SGAMAIYETLKNIDTLFG-ADD-LPTKWDQQKLENFQNIVYRQIE

ESK-CM--------MGSVDT-------S-DYLIR----------TEG------LKTYFGN

IAA-VLKEKNFSYCAWEVVRKEL---LYSL-QFILEH--NSDS-------LLRT--

>IFNC1_SASA

--------MALQTI--------W-MS---AFL----CVAHV--CSMP---MPCQLQGQLV

RITHNLLRDMGGNFPLECLQE----------N--VF----MAFPATAF----A------S

SGAP--QLGS-----SGAKAIYETLKNIDILFE-ADD-LPTQWDQQKLKNFQNIVYRQIE

ESK-CM--------MGSVDT-------S-DYLIR----------TEG------LNTYFGN

IAA-VLKEKNFSYCAWEVVRKEL---LYTL-QFILEH--NSDS-------LLRT--

>Eslu_1

--------MALTTI--------W-VS---ALL----CFVHV--WSMP---MTCHIQETLM

ESAKDLLRDMGGHFPLECLQG----------K--VN----ITFPAPAF----A------T

SSTP--TLSG-----SGAKAIYETLKNIELLFG-AED-LPTKWDQQKLDNFQNIIYTQID

HSK-CV--------SGSVET-------S-DYPVR----------AAA------LKTYFGN

IEA-ALKEKKFSYCAWEVVRKQV---LETL-IFILTK--NSDC-------LLRT--

>clha_a13

--------MVLQSF--------W-VT---IIL----CFVHV--STVP---ITCGLQRRLV

EKSHSLLESMSGLFPVECLEH----------N--LP----IAFPSSAF----M------T

SEAA--E-SA-----GAEKVAYETLKLIDTLFA-NDS-MPTSWNN--LEDFQEIIYRQIE

ESE-CI--------M-TQSK-------D-DFPTR----------NAA------LKTYFDK

IAT-ILKEKESSDCAWEVVRKEI---LYTL-KFIL-Q--SSNY-------LI----

>Anja_11

--------MAF-----------WFIF---AFL------AQA--WSMP---QTCQLDGNLI

RTSHNLLKDAGGHFPLQCIKE----------N--VL----IMFPSSAF----E------S

NGTI--EQET-----GVRMAIYETLRSLSLVLE-DGD-LPTKWDEKIMDDFQNIVYRQVD

----------------S-------------------------------------------

-------KCSFSFCAWEIVRKEL---VRTL-HFILDH--RSDM-------LCRL--

>Anja_10

------------------------------------------------------------

------------------------------------------------------------

-------QSD-----IIRTVIYETLYSINSLFE-NDD-FPTDWDEIKLQDFQNIIYRQVD

KST-CV--------SS-------------------------------------LSPEM--

--------------------------------------------------------

>IFN3_DARE

--------MDLHRV--------W-LC---TFF----CFVQV--WSLP---TNCNLQKNLM

KRTYTLLET-AGLFPMQCLDD----------R--VS----IPFPQNVF----E------Y

NE-D--QVTG-----V-EKAVYQTLQNIDALFE-NFG-DPDQWDAEKLDDFRGIVYRQIK

YST-CI--------MNKTEV-------A-DFPSR----------EAS------LKVYFET

ISS-TLKEKNSSYCAWEIVRSEI---LRTL-EFILKN--NSDI-------ML----

>Leoc_1

---------------------------------------------MP---TRCAFREHLI

EVSLNLLKDMGGHFPRECIKD----------N--VL----ITFPANVL----F------S

SFAQ--TQNE-----NIQPVVYETLRSVNTLFQ-SEG-RPSTWDQRKLEDFQSVVFRQVS

DFKKCA--------LRRRET------------------------SSANNSTVQLKTYFKK

MGR-FLEEKNYSSC------------------------------------------

>Leoc_2

--------MALEGS--------W-LC---VVF----CLSQA--WAMP---TRCAFREHLI

EVSLNLLKDMGDGFPKECLSD----------N--VV----IAFPAEAY------------

EFAE--TQKE-----DFEIAIYKTLNSTDALFE-NEG-RPTSWGQRAVDEFQNLVFRQVQ

DFNTCV--------PGDVET-------TGN--------------SSAAYRITLLKTYFQK

MEN-VLQEKNYSSCAWEIIRKEL---LGIL-QVILDK--NAEI-------VV----

>IFN2_DARE

--------MEFWQV--------F-LC---PAL----FFAHI--TSKP---TNCFMRRKHV

KTAYSLLESMGGLFPRECLKE----------N--VR----ITFPKYAL----Q------S

NNSN--QKTG-----V-AKAVYKIMDHIDFLFA-NDS-YPEAWNKRKVDNFQNIVYRLTK

ENQ-CI--------MRMRAT-------V-DFPAR----------DDA------LKSYFNK

LAT-LLRNKDNSFCAWEVVRHEL---LGVL----------SDI-------IL----

>Scma_1

--------MTP-------------LSVLLLQL----CSFQV--VAMP----TCKLQANVV

LKTHNLLRDLGAAFPVHCLQY----------N--VN----ISFPDSAF----P------D

ATAH--PQCR-----RALWVVYESLRGMQLILE-QND-SPVTWDEGILDNFQNLQHRLLE

DGS-CL--------ST---K-------GPDV----------------------FSSYFSN

VTD-VLQQQDSSVCGWMALRRDV---LSVLKTALREH--NSCF-------T-----

>IFNA_human

MALTFALLVAL------------------LVLSCKSSCS------VGCDLPQTHL-GS--

RRTLMLLAQMRRISLFSCLKDRHDFGPQEEF--------------G-N----Q------F

QKAE--TIPV-----L-H----EMIQQIFNLFS-TKD-SSAAWDETLLDKFYTELYQQLN

DLEACVIQGVGVTE--------TPLMKEDSIL--------------A------VRKYFQR

ITL-YLKEKKYSPCAWEVVRAEIMRSFSLS-TNLQES--LRSKE------------

>Loaf_11

MAFSFLLLIAL------------------VVLSCNSTCS------LGCDLPQSHL-AN--

RRTMMLLGQMRRISPFSCLKDRNDFGPQEEL-------------DG-N----K------F

QKAQ--AISV-----H-H----EMIQQTFNLFS-LQA-SSAAWDKTLLDKLYTGLYQQLN

DLEVCLMQEMGVEE--------APVINEDSML--------------A------VRKYFQR

ITV-YLTEKKYSPCAWETVRAEVMSSFSAS-TNWKER--LRSKEGDLAP-------

>IFNA_horse

MALPVSLLMAL------------------VVLSCHSICS------LGCDLPHTHL-GN--

TRVLMLLGQMRRISPFSCLKDRNDFGPQEVF-------------DG-N----Q------F

RKPQ--AISA-----V-H----ETIQQIFHLFS-TDG-SSAAWDESLLDKLYTGLYQQLT

ELEACLSQEVGVEE--------TPLMNEDSLL--------------A------VRRYFQR

IAL-YLQEKKYSPCAWEIVRAEIMRSFSSS-TNLPQS-------------------

>IFNA_pig

MAPTSAFLTAL------------------VLLSCNAICS------LGCDLPQTHL-AH--

TRALRLLAQMRRISPFSCLDHRRDFGPHEAF-------------GG-N----Q------V

QKAQ--AMAL-----V-H----EMLQQTFQLFS-TEG-SAAAWNESLLHQFCTGLDQQLR

DLEACVMQEAGLEG--------TPLLEEDSIL--------------A------VRKYFHR

LTL-YLQEKSYSPCAWEIVRAEVMRSFSSS-RNLQDR--LRKKE------------

>Oror_10

MAPTVSLLLAL------------------VLLSCHSNCS------LGCDLPQTHL-AN--

TRALMLLQQMRRISPFSCLKDRNDFGPQEAF-------------GG-N----Q------F

QKAQ--AIAV-----V-H----EMIQQTFQLFS-TEG-SAAAWDETLLDKFCTALYQQLT

DLQACLMQEAGLEG--------TPLLKEDSIL--------------A------VRKYFHR

ITV-YLQEKKYSPCAWEIVRAEVMRSFSSS-TNL----------------------

>Ptva_8

MALLFSFLMAM------------------VVLSCQSICS------LGCDLPQTHL-VN--

RRALMLLGQMRRISPFSCLKDREDFGLQGAF-------------GG-N----Q------F

QEAQ--AIAV-----F-H----EMTQQTFLLFC-TEV-LSAAWDETLLGRFCNGLYQQLD

HLEACQTQELGAEE--------TPLLDEDSTL--------------A------VRKYFQR

INL-YLQEKKHSPCAWEIVRAEIMRSYSLS-THLKEK--SRSKD------------

>Ereu_6

MAPSSLFLKAL------------------LVLSCSYIFG------LGCDLPQSHP-VN--

RRPLLLLGQMRRLPPFSCLKDRHDFAPQEVF-------------DG-Q----Q------F

QKAH--ALSV-----L-H----EMLQQIFHLFS-TKH-SSADWDEGLLNSFCAELHQQLN

VLEGCQTQEVRVEQ--------TPRMK-DSIL--------------A------MKRYFQR

ITM-YLREKKYSPCAWEIVRVEIIRAFSLS-TKLQEK--LRSKD------------

>IFNA_mouse

MARLCAFLVML------------------IVMSYWSTCS------LGCDLPHTYL-RN--

KRALKVLAQMRRLPFLSCLKDRQDFGPLEKV-------------DN-Q----Q------I

QKAQ--AIPV-----L-R----DLTQQTLNLFT-SKA-SSAAWNTTLLDSFCNDLHQQLN

DLQTCLMQQVGVQE--------PPLTQEDALL--------------A------VRKYFHR

ITV-YLREKKHSPCAWEVVRAEVWRALSSS-VNLLPR--LSEKE------------

>Oror_9

MAFVLPLLTAL------------------VVFSYGPGGS------LGCDLSQNHR-IS--

RKNFMLLGQMRRISPRFCLKDRKDFGPQDMV-------------DG-S----Q------L

PKAQ--ATSV-----L-H----EMLQQVFCLFH-TER-STATWDTSLLDKLRTGLHQQLE

DLDACLVQAMGDEE--------TALGVTGPTL--------------A------VKRYFQG

IHL-YLKEKKYSDCAWEIVRVEIMRSLSSS-TNLQER--LRIMNGDLGSP------

>Loaf_4

MALLLSLLTAL------------------VVFSCGPAPS------LGCDLPQNHV-AS--

EKTVDLLDQMQRCPTFFCLDDRKDFRPQEMV-------------DG-S----Q------L

QKAQ--AIAF-----L-H----EMLQQIFDLFR-TMD-SFAAWNTTLLNQLLNGLPEQQE

DLETCFMQAMEEGK--------SALPIEGPAL--------------A------VKEYFEG

IRF-YLKEKEYSDCAWEFVRVEIRRSFSSS-TALQER--LRRKDGDMSSS------

>Loaf_5

MAFLLFLLTAL------------------VVFGCGPAPS------LGCDLSKKHL-TS--

KKTFVVLDQMRRLSPFSCLKERKDFRPQEMV-------------DG-S----Q------L

QKAQ--VISV-----L-H----EMLQQIFNLFH-TKD-SSAAWNTTLLDQLHSGLYLQLE

DLEACLVQAMEEEE--------SVLAIESSAL--------------A------VKRYFQG

IHS-YLKEKEYSDCAWEIVRVEIKRSFSSS-TNLQER--LRRKHGDMGSS------

>Dano_12

MALQLSLLMAL------------------VVFSCGPVPS------LSCDLPQSQL-VD--

RKTFVLLGQMGRISPFSCLKDRADFRPQEMV-------------DG-S----Q------V

QKSQ--AKFV-----L-H----EMFQQIFNLYH-TEG-SSAAWNMTLLDQLLSTLHEQLE

DLEACLLQEMGEEE--------TLLGIEGPVL--------------A------MRRYFQG

IRL-YLQEKKHSDCAWEVVRMELRRAFSSS-PNLKER--L----------------

>IFNO_human

MALLFPLLAAL------------------VMTSYSPVGS------LGCDLPQNHL-LS--

RNTLVLLHQMRRISPFLCLKDRRDFRPQEMV-------------KG-S----Q------L

QKAH--VMSV-----L-H----EMLQQIFSLFH-TER-SSAAWNMTLLDQLHTGLHQQLQ

HLETCLLQVVGEGE--------SAGAISSPAL--------------T------LRRYFQG

IRV-YLKEKKYSDCAWEVVRMEIMKSLFLS-TNMQER--LRSKDRDLGSS------

>IFNT_cow

MAFVLSLLMAL------------------VLVSYGPGRS------LGCYLSEDHL-GA--

RENLRLLARMNRLSPHPCLQDRKDFGPQEMV-------------EG-S----Q------L

QKDQ--AISV-----L-H----EMLQQCFNLFH-IEH-SSAAWNTTLLEQLCTGLQQQLE

DLDACLGPVMGEKD--------SDMGRMGPIL--------------T------VKRYFQD

IHV-YLKEKEYSDCAWEIIRVEMMRALSSS-TTLQKR--LRKMGGDLNSL------

>Ptva_4

MAPLLSLITAM------------------LVFSYGPSGS------LSCDLSQNHQ-VN--

KESIVLLHQMQRISSFRCRKDRKNFGPQEMV-------------DG-S----Q------V

QEAQ--AISV-----L-H----EMLQETSNVFG-SEH-SSAAWNTTVLHGLLSRLHWQLE

DLGTCLVLQMKEAE--------SALGMEAPTL--------------A------VKRYFQG

IRL-YLKEKQYSDCAWEIVRVEIKRAFSLS-TNLREM--LRNQDGDLRSP------

>Dano_2

MAFPVSSLVVL------------------MMIFSSPIGS------FSCGLPQSLV-RK--

QETFTVLSQMGTISLLSCLKDRTDFRPQEMM-------------DG-S----Q------V

QKTQ--AMSV-----L-H----EMLQQIFHLFH-TEG-SSAAWNTTLLDQLRSGLHRQLE

DLETCLLQEMG-ED--------SVLAMEGPTL--------------A------VRRYFQR

IRV-YLQKKKHSDCAWEVVRVEIRRCFLFI-NVLTRE--LRK--------------

>Ptva_16

MAFLVSSLMAL------------------VVIFSSPISS------MICDLPQSLL-GK--

QETSTALNQMQRISSFLCQKDRKDFRPRKMV-------------DG-S----Q------V

QKAQ--AISV-----L-H----EMLQQTFDVFG-TKQ-SSAAWNTTLLHGLLSGLHRQLE

DLGTCLVPEMKEVE--------SVLGTEDPTL--------------A------MKRYFQG

IHL-YLEEKQYSDCAWEVVRVEIRRYLFVV-NKFTRK--EI---------------

>Dano_10

MAPPVSVLKTL------------------LMLCSIPAC-------LGCDLPLIY--GH--

QEPFMLLHQMGRLSILSCLKDRTDFQPQELM-------------DG-I----Q------L

DKMH--ATTL-----L-H----EVVQQIFNLFS-TSG-SLATWDDTLLDRFLIGLHQQLD

NLETCLGKEKEEDQ--------THLGSENSRL--------------A------VKRYFQG

ISQ-YLTEKQDSPCAWEVVRVEIRKCFLFI-NKLQGK--LRK--------------

>Loaf_1

MALPISVLMAL------------------VMFCCRPACF------WCCDLPLSH---N--

QETFTLLNQMERISLLSCLKDRTDFRPQILM-------------DM-N----Q------L

EKTQ--AAVL-----L-Y----EMLQQTFNLFS-RSD-SLEAWDETFLDKFLLGLYQQLN

DLEICFEKERKVEQ--------IPLGTEN-----------------S------VKSYFQG

IGL-YLKEKEHSLCVGGCQSGN-QKMLSLH--------------------------

>Modo_6

MTSWSLLPVAL------------------ALLCSSTLCS------LDCDLTLGL------

QEDFSLLNQMSTSSLVPCLKDGINFNPKEAM-------------DR-S----Q------L

QKEN--ATVI-----V-L----EMVQQIFTLFS-QNT-TPATWNQTQVIQLLIRLDQQLE

QLERCLGQNVKWEE--------FSLRSEKTRF--------------A------LKSYFQG

ISQ-YLQGKEYSPCAWEIVRVEIRRLFLFM-SKLARK--LRD--------------

>Oran_7

MTNAGLIQIVL------------------VLLVSTSTVS------LSCSLLHT----VCM

EQSLKRLDRMQGKSLLSCLKDRKDFQPQELV-------------EA-G----P------F

KEGN--RAVA-----V-H----ELLQQIFTIFS-QNL-SQTGWDQSEVENFLHGLHRQLE

ELEVCQ--GTDTRW--------ASVGSDILRL--------------R------LKSYFRS

ISL-YLRDKDYSSCAWEIVRAQIRRC---I-FQFMRR--LRN--------------

>Oran_1

MTNRSSLPFVL------------------WLLLPTTIMA------QGYPKLYSHQ-WLSN

WQSLHLLDEMGGQFPLHCLKEKTNFKPAEMM-------------HP-H----Q------F

QQEN--ATEA-----I-H----DLLQNIFNIFG-RNH-SQTGWDEATVEKFLHGVHKEMM

RLELFE--EMGWEN--------STLRGDV-SL--------------H------IKSYFKG

MMD-YLKGRDYSSCAWEVTRMEAKRCFLVM-YRLTRK--LKK--------------

>IFNE_human

MIIKHFFGTVL------------------VLLASTTIFS------LDLKLIIFQQ-RQVN

QESLKLLNKLQTLSIQQCLPHRKNFLPQKSL-------------SP-Q----Q------Y

QKGH--TLAI-----L-H----EMLQQIFSLFR-ANI-SLDGWEENHTEKFLIQLHQQLE

YLEALL--EAEKLS--------GTLGSDNLRL--------------Q------VKMYFRR

IHD-YLENQDYSTCAWAIVQVEISRCLFFV-FSLTEK--LSK--------------

>IFNE_cow

MINKAFFEIVL------------------VLLASSTVCS------QELKLVLCQQ-RRVN

QESLKLLNKLQTSSVQQCLPHRKHFLPQKSV-------------NP-H----Q------Y

QKGQ--VLAI-----L-H----EMLQQIFSLFR-AIV-SLDGWEESHTEKFLVELHQQLE

YLEALL--QAKQKS--------DTLGSENLRL--------------Q------VKMYFQR

IHD-YLESQDYSSCAWTIVQVEINRCLFLV-FRLTRK--LSE--------------

>Oror_7

MINKPFFDIVL------------------VLLASSSVCS------RELKLVLFQQ-KRVN

RESLKLLNKLQTSSIQQCLPHRKNFLPQKSM-------------NP-H----Q------Y

QKGQ--ALTI-----L-H----EMLQQIFNLFR-AII-SLNGWEESHMEKLLIELHQQLK

YLEALR--QAEQKR--------DTLGSENLRL--------------Q------VKIYFQR

IRD-YLENQDYSTCAWTIVQVEINRCLFFV-FQLTGK--LSKQ---------ET--

>Ptva_15

MISKYFFEVVL------------------VLLASSTVFS------LELKLVLFQQ-RRVN

RENLKLLNKLQTPSIHQCLPHRKNFLPQKSL-------------NP-H----L------Y

QKGC--ALAI-----L-H----ETLQQIFSLFG-ANI-SLDGWEESHMEKFLIELHQQLE

YLETLQ--QAEQKS--------GILGSENLRL--------------Q------VKMYFQR

IRD-YLETQEYSRCAWTIVQVEINRCLFFV-FQLTGK--LSKQ---------DP--

>Dano_16

MINKHFFEIVL------------------VLLASSTLFS------LELKLVLFQQ-RQVN

RESLKLLNKLHT-SIQQCLPHRKNFLPQESM-------------NP-Y----Q------Y

QKGH--AVAI-----L-H----EMLQQIFNLFR-EKL-SLAIWEESQVEKFLIELHQQLE

HLEALQ--EPELKS--------DTLDSETFRL--------------Q------VKTYFRR

IRD-YLENQEYSSCAWTIVHVEINRCLFLF-TDSQES-------------------

>Loaf_2

MINKYFFETVV------------------VLLSSSMIFS------LELKLVHFQQ-R-MN

RESLKLLNTLWSSSIQQCLPHRANFAPQKSM-------------NP-H----Q------Y

HKGH--AVAI-----L-H----EMLQQIFNLFR-TNL-VLGSWEERHMEKFLIELYGQLE

HLEALL--EAEQKS--------GSLGTENLRL--------------Q------VKMYFQR

IHN-YLENQKYSSCAWTIVRVEIIRCLFFV-FRLTGK--LSQY---------DP--

>Ereu_10

MISKHIF--VL------------------ILLASSPIFS------LELKLFLVQL-RRLN

RESLKLLNIRQTSSIQRCLPHRKNFLPLKSP-------------SP-H----W------Y

QTEH--ALAI-----L-H----EMLQQIFNLFR-VNI-SLDDCEESYMEKFLMELHQQLE

LLEAFL--EAEQNS--------NTLSSENLRM--------------Q------VKMYFQR

IHN-YLGKQGYSNCAWTIVRVEINRCLLFM-LRLTTK--LSKQ---------DF--

>IFNE_mouse

MVHRQLPETVL------------------LLLVSSTIFS------LEPKRIPFQL-W-MN

RESLQLLKPLPSSSVQQCLAHRKNFLPQQPV-------------SP-H----Q------Y

QEGQ--VLAV-----V-H----EILQQIFTLLQ-THG-TMGIWEENHIEKVLAALHRQLE

YVESLL--NAAQKS--------GGSSAQNLRL--------------Q------IKAYFRR

IHD-YLENQRYSSCAWIIVQTEIHRCMFFV-FRFTTW--LSR--------------

>Dano_1

MDHM-YLLLAG------------------LMLCSSLDCS------LGCPLPRSQL-ES--

KEIFTLLRQMNRIPSHSCLNDRVDFKPWKAE-------------TV-T----Q------I

PKTQ--ATCF-----S-Y----EMFQQIFNLFQ-KEN-SRAAWDNSLLDELLSRLDHNLE

QV-----EQMKVE----------NLPCADLGT--------------L------VRDYLQG

TDG-YLNEKKYSSCAWEVVRGEPEMCFPLI--------------------------

>Oror_2

MAQI-YLLVAG------------------VLLCSIPAYS------LGWNLPRSHQ-EN--

KDVFQHLEQLQRIPSQWCLKDRTDFKPWKRE-------------NI-T----P------I

QVTQ--GTCH-----H-H----LMLQQIFNLFT-TED-SRAAWNNTLLDKLLSSLHLRLH

RL-----EQMKKD----------NLDCRDLGR--------------A------AREYFHG

IHV-YLKAKEYSPCAWEVVRVEIKRCLSLM--------------------------

>Ptva_17

MAQCSSWLAAG------------------EMLSFILICS------LGGDVPWIHL-EN--

RKIVSLLRELEVIPSHFCLKDRTDFKPWERG-------------SI-T----E------I

QKTQ--RTCF-----H-H----LILQQIFSLLN-AED-SHAAWNRTLLYQLLSRLHHSLE

EL-----DQTNEG----------NLVCPDLGI--------------L------VWNYFQG

IHN-YLKQKKYSTCAWEVVRVEITARLFLM--------------------------

>Ereu_8

MFQ--FLLMTG------------------VMLSSILACS------YGQD----HP-EK--

RVILMLLTQLKNTPSLSCLKDRTDFHPWSRG-------------EI-T----Q------I

HMAQ--GPCF-----Q-K----LMLQQVFRLFN-TEA-SRAAWNNSLLDRLLSSLYDSLE

QL-----EQMEV-----------SLACPSVGT--------------D------ALKYFQR

IKI-YLKAKKYSACAWEIVRSEIEARFFLI-LGTLRR--LGQRTESLGPP-LRAA-

>IFNB_human

MTNKCLLQIAL------------------LLCFSTTALS------MSYNLLGFLQ-RSSN

FQCQKLLWQL-NGRLEYCLKDRMNFDPEEIK-------------QL-Q----Q------F

QKED--AALT-----I-Y----EMLQNIFAIFR-QDS-SSTGWNETIVENLLANVYHQIN

HLKTVL--EELEKE--------DFTRGKMSSL--------------H------LKRYYGR

ILH-YLKAKEYSHCAWTIVRVEILRNFYFI-NRLTGY--LRN--------------

>IFNB_pig

MANKCILQIAL------------------LMCFSTTALS------MSYDVLRYQQ-RSSN

LACQKLLGQL-PGTPQYCLEDRMNFEPEEIM-------------QP-P----Q------F

QKED--AVLI-----I-H----EMLQQIFGILR-RNF-SSTGWNETVIKTILVELDGQMD

DLETIL--EEMEEE--------NFPRGDMTIL--------------H------LKKYYLS

ILQ-YLKSKEYRSCAWTVVQVEILRNFSFL-NRLTDY--LRN--------------

>Oror_6

MNHRCILQTAL------------------LLCFSTTALS------MSYRLLQFQQ-RSSN

LACQKLLQRL-PGMPQHCLEDRMDFKPEEIK-------------QP-Q----Q------F

RKED--AVLV-----T-Y----EMLQQIFGILR-RNF-SSTGWTETITENLLVEVYGQMD

RLETIL--EEMEKE--------NF-TSVVTIL--------------H------LKKYYLQ

IMQ-YLKSKEYSNCAWTVVRVEILRNFSFL-NRLTDY--LHN--------------

>Dano_11

MANRCAFQIAL------------------LLSFSTMALC------ISYNVLRFQQ-SSSN

LICQKLLKKL-NGSAEYCLQDRMDFKPEEIK-------------QP-Q----Q------F

QKEE--AALL-----I-Y----EMLQQIFGIFQ-RKF-SSTGWNETIVENLCVELYQQMD

RLETIL--EELEEE--------SFTWGDMTIL--------------H------LKNYYLR

ITQ-YLKAKEYSSCAWTVVRVEILRNFSFI-NRLTEY--LQN--------------

>Ptva_12

MTNRCILQFAL------------------LLCFSTTALS------MSYNWLRFQQ-RSSN

LACLKLLWQL-NGTPQYCHKDRMDFKPAEIK-------------QP-Q----Q------F

QKED--TVLI-----I-H----EMLRQIFDIFQ-RNF-SSTGWNETIIMNLYVTLSGQMD

RLETAM--EEMEEE--------NFTWESMTVL--------------H------LKNYYFR

IMR-YLETKLYSRCAWTVVKAEILRNFFFL-NGLTEY--LQN--------------

>Loaf_13

MTTRCILQVAL------------------LLSISTTALA------RSYKLLQFQQ-RSSN

LACQKLLWKL-NGAPESCLEDRMDFKPEEIK-------------QP-G----Q------L

QKED--AALV-----I-Y----EMLLQIFDIFL-GNF-SHTGWDETVIENLLAELSQQRD

RLVTIL--EEMEEE--------NPTSRNMTIL--------------H------LKNYYLG

IGQ-YLEAKDYSSCAWTVVQVEILRNFSFI-SGLTDY--LQN--------------

>IFNB_horse

MTYRWILPMAL------------------LLCFSTTALS------VNYDLLRSQL-RSSN

SACLMLLRQL-NGAPQRCPEDTMNFQPEEIE-------------QA-Q----Q------F

QKED--AALV-----I-Y----EMLQHTWRIFR-RNF-ASTGWNETIVKNLLVEVHLQMD

RLETNL--EEMEEE--------SSTWGNTTIL--------------R------LKKYYGR

ISQ-YLKAKKYSHCAWTVVQAEMLRNLAFL-NGLTDY--LQN--------------

>IFNB_cow

MTYRCLLQMVL------------------LLCFSTTALS------RSYSLLRFQQ-RQSL

KECQKLLGQL-PSTSQHCLEARMDFQPEEMK-------------QE-Q----Q------F

QKED--AILV-----M-Y----EVLQHIFGILT-RDF-SSTGWSETIIEDLLKELYWQMN

RLQPIQ--KEMQKQ--------NSTTEDTIVP--------------H------LGKYYFN

LMQ-YLESKEYDRCAWTVVQVQILTNVSFL-MRLTGY--VRD--------------

>Ereu_12

MANRYIFQIAL------------------LLCI-TTALA------ESYTLDQSQQ-KSSI

LVCQDLLNQL-NGSATDCLKQRMNSKPEEIK-------------NP-Q----L------L

QKED--LVLV-----T-Y----ELFQQIFGIFS-RNF-SRTSWNETIVEKLLMELYQQKN

QLKTTV--EEIKET--------NDIWGNKHIL--------------N------LKKYYFS

LMR-YLKANKYSSCAWIIIKTEIIRNFVYL-DKLISY--FSN--------------

>IFNB_mouse

MNNRWILHAAF------------------LLCFSTTALS------INYKQLQLQE-RTNI

RKCQELLEQL-NGKIN--LTYRADFKPMEM---------------T-E----K------M

QKSY--TAFA-----I-Q----EMLQNVFLVFR-NNF-SSTGWNETIVVRLLDELHQQTV

FLKTVL--EEKQEE--------RLTWESSTAL--------------H------LKSYYWR

VQR-YLKLMKYNSYAWMVVRAEIFRNFLII-RRLTRN--FQN--------------

>Modo_7

MVYRGILYLAL------------------LLLFSPSISS------KGYDSLRFHQ-RRTN

QRSLMFLNKMIGKLHPECLQERMDFQPREIV-------------QP-R----Q------C

QREN--ATMI-----I-H----EMLQQTLILFS-SKN-ACPDVNDTIIEPFLSGIYQQML

HLE----EEMDQAN--------SSWESLESIL--------------R------LKNYYQG

ITN-YLKNKEYSSCACKIVQVETRRNFSFL-YKLTEY--LKN--------------

>IFN1_anoli

-----LLPIAL------------------TMVLITEVSS------QDCGQLLARL-RQAN

KANLELLNSKMNSTPQQCIEGVFSFSLKNKL-------------TNRD----V------S

EEEN--AKVA-----I-Q----EVLQQTGHIFR-QNC-TEMLWDEDSLRAFHAGLDQQSE

NLKSCL----------------SASIQL-TSL--------------R------VKRYFRS

LND-FLKEKEYNRCAWEIIQIQVKQCFLWI-EKLIQE--IQSK---------MAH-

>Opha_2

-----FLQICL------------------VMF-FTNVSS------QHCDQLHSRL-QEDN

KGNLELLGSHMRATPLECIGDIADFS-EENV------------MSMNE----A------S

HEED--AKIA-----I-Q----EMLQQTDLIFK-KVH-AELFWDETSLRTFHTGLDQQIK

RLETCQ----------------NASLQL-TRL--------------R------VKRYFQG

LND-FLKDKQYSSCAWEIVQIQLRECFLLI-HQLIQR--IPTQ---------IKY-

>Vibe_2

------------------------------MF-FTKISS------QHCDQLHTRL-LEAN

KGNLELLGSHMRATPLQCIGDIVDFS-EEHL------------ISVDE----A------S

HEED--AKRA-----V-R----EMLQQTDLSFK-QAH-AELFWDENSLRQFHTGLDEQIK

KLETCQ----------------SASLQL-TRL--------------R------VKRYFQG

LNH-FLKEKKYSLCAWEIVQIQLRECFLLI-HQLIQR--IPIQ---------IMY-

>Pybi_2

-----LLQICL------------------AMF-FTKISS------QHCDQLHRRL-HKAS

KGNLKLLGSNIRATPLQCIGDIIDFS-EENL------------MSMDG----A------S

HEEN--AKIT-----I-Q----EMLQQIDLIFK-QVH-AELFWDENSLRQFHTGLYQQIK

ELEICQ----------------NASLQL-TRL--------------R------VKRYFQR

LSD-YLKDKKYSLCAWEIVQIQLRECFLLI-NELIQR--IPT-----------LY-

>Opha_6

-----CLFICL------------------GVF-FTEISP------QDCNQIRSRL-HEAN

LRNMNLPMRNMGSTPQQCIRDIIDFSLEENL------------TNMIN----E------L

QGET--AKVA-----I-K----ELLQQIDLIFK-ESH-SELAWDENSLREFHIGLHQEIK

NTKACW----------------NTSLQF-TRL--------------R------VKRYFQR

LRD-FLKNKEYNLCAWKIMQIQIRECFEWI-NHLNQR--IPSE---------T---

>Thsi_3

-----CLFICL------------------GI--FTEISS------QDCNQFRSRL-HEAN

LGNLNLLTRNMGSTPQQCIRDIIDFSLEENV------------MNMVN----E------L

QGEN--AKVA-----I-K----ELLQQIDLIFK-ESQ-SELAWDENSLREFHIGLDQEIK

KTAACW----------------NTSLKL-TRL--------------R------VKRYFQR

LRD-FLRNKDYNLCAWKIIQIQIRECFQWI-NQLNQR--IPNE---------T---

>Vibe_6

-----CLFICL------------------GIF-FTEISS------QDCNQLRSRL-HEAN

LGNLNLLTRNIGSTPQQCIRDIIDSSFEENL------------MNMVN----K------L

QGEN--AKVA-----I-K----ELLQQIDLIFK-ESH-SELVWDENSLREFHIELDQEIK

KAETCW----------------NTSLQF-TRL--------------R------VKRYFQR

LRH-FLKNKEYNLCAWKIIQIQVRECFEWI-NQLNQR--IPSE---------T---

>Opha_7

-----CLYICL------------------GIIFFGDISC------QNCNQLQRKL-LKAN

KDNSNLLSSNIRPTPLQCMRSFVELSLKKIM------------IDMND----E------C

QVDI--AKTA-----V-K----EILQQIDVIFR-QNH-TELVWHEGSLRDFHIGLDQQIK

MLETCG----------------NASLQL-TRL--------------R------IKRYFQR

LSD-FLKNKKYSLCAWEIVQIQIEACFQLI-NHYIQR--IRSK--------TMKK-

>IFN5_anoli

MAQQCLLAFCL------------------LMSF-REILS------QDCNDLRHEL-NGAN

KANLELLNVKMGSTPLQCVDDVINFSSKESL-------------PSIY----D------F

EEEN--ATVA-----I-D----EILQQISYLFN-QNH-TKLSWDENSIATFKLGVDNEIK

KLTPCL----------------SDSIDE-LRD--------------K------VRKYFER

INN-LLKEKEYNLCAWEIVQMEVRQCLIVV-DQLISR--IPKK---------KAV-

>Chmy_15

-----LPRVCL------------------VLLFFTEISS------RLCTMLHFQQ-KKMN

RESLEHL-KKMSGNPSQCINERAASKP-QDV-------------AQLP----V------S

QKEK--A--------------------IFSIFS-KNL-TQSAW---------NGLL----

------------------------------------------------------------

--------------------------------------------------------

>chpi_1

-------------------------------------------------MLHFQE-NKGN

KESVELL-KKMSENLSQFINEMKAFKP-QDV-------------VQLQ----L------S

QKEN--ADVA-----I-Q----EILQEIFTIFS-KNL-TQTAWDRSSIARFQNGLYQQIQ

PLEVCL----------------GA------------------------------------

--------------------------------------------------------

>Almi_3

-----LLHICL------------------VLLFSTEISS------QHCDLLSFQQ-KKLN

KDSLELL-EKMGGNPFQCFSERTDFKS-QDV-------------LKLQ----L------S

QKEN--AKLA-----I-Q----NILQEIFTVFS-KNL-TQTAWDEISIITFQNKLHQQIE

RLEACL----------------GFKLVL-TKL--------------K------IKRYFQG

IYN-FLEEKQYSLCAWEIIRMEMTRGFLLV-DQLTKS--I----------------

>Crpo_3

-MKMMLLHICL------------------ILLFSTEISS------QHCDLLSFQQ-KKLN

KDSLELL-EKMGGNPFQCFNEGIDFKS-QDV-------------LKRQ----S------S

QKEN--VKLA-----I-Q----NILQEIFTVFS-KNL-TQTAWDTISIITFQNKLHQQIE

RLEACL----------------GFKLVL-TKL--------------K------VRRYFQG

IYN-FLEEKQYSLCAWEIIRMEMTRCFLFV-DQLTKS--L----------------

>Gavga_8

FLSSFLLHVCL------------------VLLFSTEISS------LHCGLLNFQQ-KKLN

KDSLELL-DNMGGNPSQCSNERIDFKP-QDV-------------LMLR----S------S

LKEN--AKMA-----I-Q----EILQEVFTVFS-KNL-TQTAWDEASIVVFQNGLHWQTE

RLEACL----------------DLKALL-TRL--------------K------LKRYFQG

IRN-FLEGK-----------------------------------------------

>Crpo_4

FLSSFLLHVCL------------------VLLFSTEISS------LHCGLLNFQQ-KKLN

KDSLELL-DNMGGNPSQCSSERTDFKP-QDI-------------LMLR----S------S

LKEN--AKMA-----I-Q----EILQEVFTVFS-KNL-TQTAWDEASIVAFQNGLHWQTE

RLEACL----------------DLKVLL-TRL--------------K------LKRYFQG

IRN-FLEGKQYSLCAWEIIRLEMPRCFLLL-DLLTKW--LKI--------------

>Gavga_6

---------------------------------------------------------KIE

Q-SLELL-DKIGRKPSQ-SNEKIDFKP-QDV-------------LVLG----S------T

LKEN--AQMA-----I-Q----EALQGVFTVFG-KNL-MQTAWDETFIVMFQNGLHWQIK

KLEACL----------------GLKVLL-TRV--------------K------LKNYFQG

ICN-FLEGKQYSLCAWEIILLEMHRYFLLL-DQLTKW--LK---------------

>Almi_11

----------L------------------VNLYHAR---------------KQER-MKIN

K-------EKRSPHDISRKYKKIDFKP-QDV-------------LILH----S------T

LKEN--AKMA-----I-Q----EILQGVFTVFS-KNL-MQTAWDETSIVMFQNGLHWQIK

RLEACS----------------GWEVLL-TRL--------------K------LKSYFQG

I-------------------------------------------------------

>Chmy_2

-----LLHICL------------------VLLFSIEISS------LDCNMLHFQQ-NKMN

MESLELL-SKMGGQPLQCLNENRNFRF-QKA-------------LRPR----E------S

QEKN--AKVV-----I-Q----EILQQIFNIFS-KNL-TQAAWDRSSVETLQKGLHQQTE

QLETCL----------------YS-LLF-PML--------------K------LKKYFQR

IRD-FLKEKQYSLCAWETIRLEMGRCFFFV-DQLIIR--LQN--------------

>Chpi_17

-----LLHICL------------------IMLFSTEISS------LDCTILHFQQ-NKMN

MESLELL-SKMGGQPLQCLNENRNFRL-QKA-------------LRPR----E------S

QEKN--AKMV-----I-Q----EILQQIFNIFS-KNL-TQAAWDRSSVETLQNGLHQQTE

KLETCL----------------HLYLLF-PML--------------K------LKKYFQR

IRD-FLKEKQYSLCAWETIRLEMGRCLLFV-DQLIKR--L----------------

>Chmy_1

-----FLHICL------------------VLLFSTKTSS------VDSNMLHFQQ-NKVN

QASLQLL-EKMGGQPVQCLNENSNFIS-QNV-------------LSSR----E------F

QKES--VIVA-----L-Q----EILQQIFNIFS-KSQ-LQTAWDRSSMDAFQNGLHHQIE

LLKTWF----------------NEY--F-TIL--------------K------VKKYFHV

IDN-FLKEKQYSLCASEIIREEMRRCFLFI-DQLTKR--LKN--------------

>Chpi_2

-----FLHICL------------------VLLF-TENSS------VDCNMLHFQQ-NKVN

QASLQLL-EKMGGQPVQCLNENSNFIS-QNV-------------LSSR----E------F

QKEN--AMVA-----I-Q----EILQQIFNIFS-KSH-IQTAWDRSSIVAFQNGLHQQIE

LLKTWF----------------DGY--F-TRL--------------K------VKKYFHV

VDN-FLKEKQYSLCAWEIIREEMRKCFLIM-DQLTKR--LKN--------------

>Pesi_2

-----FLHICL------------------VLHISTKISF------VDCNMFLFQQ-NKVN

QDSLKLL-EKMGGQPVQCLNEKSNFIS-QNL-------------FSST----E------F

QKEN--AMMV-----I-Q----EILQQSFTIFR-KIQ-IQTDWDRSSIAAFQNGLYQQIE

LLKTWF----------------DGY--L-TRL--------------K------VKKYFHV

IDT-FLEKRQYSRCACEIIREEMRRCFLFI-DQLTKR--LKN--------------

>Chpi_8

----------------------------------------------------------MN

SESLEHL-EKMGGNPFQCLNERTAFKP-RDI-------------LKIR----L------S

QQEN--AKVA-----I-Q----QILQELFHIFN-NNL-TQAAWNGTSIKEFQNGLHQQIE

KLETCL----------------SAYLLL-TSL--------------K------LKRYFQT

IDD-FLKEKQYSQCAWEIIRVEISRCFPIL-NILTKR--LQDE--E------LKY-

>Pesi_3

----------------------------------------------------------MN

SKSLEHL-EKMGGPPFQCLNERSAFKA-TDI-------------LKVR----L------A

QQEN--AKAA-----I-Q----QILQELFQIFS-KNL-THAAWDGTSIKEFQNGIHQQIE

KLEVCL----------------SAYLLH-TSL--------------K------LRRYFQT

VRH-FLKEKQYSRCAWEIIRLEVSRCFLVL-NILTKR--IEN--------------

>Almi_2

-----LLKFFL------------------VLLLFKVSSS------LHCNSLASNQ-NKVN

KDGLDFL-DKMRRNSPQCLSERLDLKT-KDI-------------FKIE----L------S

QKDN--AKAA-----I-Q----ELLKAIFYVLS-NNL-TQTTWQESSIEKFKNGLHWQIE

NLETCL----------------DASPLV-TRL--------------K------LKRYFQA

IDN-FLKEKQYSQCAWEIISVELSRCFQFI-DKLTIK--LSTS-------------

>Crpo_5

-----LLKFFL------------------VLLFFKVSSS------LHCSSLASNQ-NKVN

KDGLDFL-DKMRRNSPQCLSERLDLKT-KDI-------------FKIE----P------S

QKHN--AKAA-----I-Q----ELLKAIFYVFS-KNV-TQMTWQESSIEKFKNGLHWQIE

NLETCL----------------DASPLV-TRL--------------K------LKRYFQA

IDN-FLKEKQYSQCAWVIISVELSRCFQFI-DKLTKK--L----------------

>Gavga_7

-------------------------------------SS------LHCSSLASNQ-NKVN

KDGLDFL-DKMRRNSPQCLSERLDLKT-KDI-------------FKIE----L------S

QKHN--AKAA-----I-Q----ELLKAIFYVFS-KNL-TQTTWQESSIEKFKNGLHWQIE

NLETCL----------------DAGPLV-TRL--------------K------LKRYFQA

IDN-FLKEKQYSQCAWEIISVELSRCFQFI-DKLTKK--LRYC-------------

>Almi_1

MMKNKLLHICL------------------VLLFSTEITS------MQCDMIHFQQ-KRLN

KDSLELL-EKMGGSPFQCSNEN--------------------------------------

--------------------------QIQDYPG-KNL-TQTTWDWSSIVTFQNGLHRQIQ

LLEACL----------------VATQLH-TRL--------------K------LNRYFQR

IHN-FLEEKHYNLCAWEIIRIEMPNCFLFV-DQLTKS--LKN--------------

>Chmy_5

-------------------------------------MS------LDCNLLRHQQ-SKFN

WYSLQLL-QNMGGKPLECLEDKTAFQF-EKI-------------LKPK----F------L

QQ----AQMS-----V-H----EILEQLFGIFS-RNL-SQTGWERRKVERFLNGLALQTE

RLEECL----------------HT-------L--------------R------LKKYFQR

IQD-FLKEKKYSTCAWEIVREEGQRCFQYI-HKLTVR--MKN--------------

>Chpi_4

-----LWQICL------------------VLLFSAGVMS------LDCNLLRHQQ-SKFN

GYSLQLL-QNMGGNPLKCLEDKTAFQF-EKV-------------LKPK----F------Q

QH----AKMA-----I-H----EILQQLFGIFS-RNL-TQTGWERTKVGSFLNGLTLQTE

RLETCL----------------PT-------L--------------R------LKKYFQR

IQD-FLNEKKYSTCAWEIVREEGQRCFQYI-DKLTVR--MKK--------------

>Pesi_4

-----LWQLCQ------------------LLLFSAGVMS------LDCNLLHHQQ-SKFN

RYSLQLL-QKTGRSPLECLGDLTAFQF-EKV-------------LKHK----F------P

QH----AQMA-----A-H----EILQQLFGIFS-RNL-LQTRWEKGDVELFRNGLHLQTK

HLEKCL----------------ST-------L--------------R------LKRYFQR

IKD-FLEKKKYSTCAWETVRLEAQRCFLYM-DKLTVM--MKN--------------

>Chmy_4

-----LLQISL------------------VLLCTTKIST------LDCNTLPLLH-NKVI

QGNLHLL-NKMGQQPEQCQSEKMHFKF-EQF-------------LKLR------------

QKEN--AKVE-----I-Q----EILQQTFYVFT-KNL-TLAAWDGRALERFQNRLNQQIE

HLEACL----------------TEY--I-IRL--------------K------LKKYFQK

IDN-FLKDKQYSLCSWEIIRLEMRRCLQFI-DKVIRR--LRN--------------

>Pesi_1

-----LLQISF------------------MLLCTTNISA------LDCNILPLLH-NKMI

QGHLHVL-NKMGQQPEQCQSEKMHFQF-EKF-------------LKLR------------

KKEN--AKVA-----I-H----EILLQIFYIFT-KHL-TLVAWDGRSLERFQNGVNQQIE

HLDACL----------------TEY--I-IRL--------------K------LKKHFQK

LDN-FLKDKHYSLCSWEIIRLEIRRYLHFI-VKVTRR--LRN--------------

>Coli_2

-----LIQIGL------------------IVLCITIISS------HQCNHLPLQQ-RKAI

ENSLQLL-DKMGEKPQRCLREKMSFKF-KQV-------------LKPT------------

QKEA--VEVA-----I-E----EIFQHIFYIFS-KNL-TLAAWDGTALEKFQNGLYHQIE

QLEACV------------------Y--V-NRL--------------K------LKKYFQK

IDC-FLKDKQHNLCSWEISRAEMRRCLQLI-DKVIRK--LYKV-----------H-

>Apfo_3

-----LTQIGL------------------ILLCTTTISS------LQCNHLHLQQ-RKVI

ENSLQLL-DKMGEKPQQCLKEKMSFRF-EQV-------------LKPR------------

QKET--VKVV-----I-E----EIFQHIFYIFS-KNL-TLAAWDGTALEQFQNGLYQQIE

QLEACV------------------Y--V-NRL--------------K------LKKYFQK

IDC-FLKDKKHNLCSWEISRAEMRRCLQLI-DKVIRK--LNN--------------

>Fape_1

-----LIQIGL------------------ILLCTTTISS------LQCNHLPLQQ-GKVV

ENSLKLL-DKMGKKPQQCLREKMSFRF-EQV-------------LKPR------------

QKEN--VEVV-----V-E----EIFQHIFYIFS-KNL-TLAAWDGTALEQFQNGLHQQIE

QLEACV------------------Y--V-NRL--------------K------LKKYFQK

LDC-FLKDKKHDLCSWEISRAELRRCLQLI-DKVIRK--LNN--------------

>Tyal_2

-----LIQIGL------------------ILLCTTTISS------LQCSHLPLQQ-RKVI

KNSLQLL-DKMGKKPRQCLREKMSFRF-KQV-------------LNPR------------

QKET--VKVA-----I-E----EIFQNIFYIFS-KNL-TLAAWDGTALEQFQNGLYQQIE

QLEACV------------------Y--V-NRL--------------K------LKKYFQK

IDC-FLKDKQHNLCSWEISRAEMRRCLQLT-DKVIRK--LNN--------------

>Gaga_3

-----FIQIGF------------------ILLCTITISS------LTCNHLPLQQ-RRVI

ESSLQLL-DKMGRRPQQCLREKMSFRF-EQV-------------LKPR------------

QKET--VKVA-----I-E----EILQHIFYIFS-KNL-TLAAWDGAALEQFQNGLYQQIE

KLEACI------------------Y--V-NRL--------------K------LKKYFQK

IDS-FLKEKQHNLCSWEISRAEMRRCLQLI-DKVIRK--LYK--------------

>Caan_3

-----LIPTGL------------------ILLCTTTISC------LWCNHLPLQQ-RKVI

QNSLQLL-DKMGNKPQQCLKEKMFFSF-EQV-------------LKPR------------

QKES--VKVA-----I-E----EIFQHIFYIFS-RNL-TLAAWDGAALEQFQNGLYQQIE

QLEVCV------------------S--V-NRL--------------K------LKKYFQK

IDC-FLRDKQHNLCSWEISRAEMRKCLQMI-DNVIWK--LNS--------------

>Stca_2

-----LLQIGL------------------ILSCTTNISS------LHCNHLSLQQ-SKVI

ESSLQLL-DKMGEKPQRCLRERMSFRF-EQV-------------LKPR------------

QKET--VKMA-----I-E----EILQHIFHIFS-KNL-TLAAWDGQALEQFQNGLYQQIE

QVEACV------------------Y--A-SRL--------------K------LKKYFQK

IDY-FLKDKQHSMCSWEISRAEMRRCLQFV-DKVIKR--LNN--------------

>Almi_5

--------MFF------------------ILLCTMQIST------PDCNIPSLQQ-SKAI

QSSLHLL-DKIGQAPLQCRREHVLFKF-HNI-------------LKLS------------

QKDN--VKVA-----V-Q----ETLQSIFYMFS-KNL-TLAAWDGRSLESFQNGLYQQIE

QLEACS----------------IKY--A-NRL--------------K------LKKYFQR

IDN-FLKGKQYSLCSWEIIREEVRKCLQLI-EKGLEG--LENK---------IKND

>Gavga_2

--------MFF------------------ILLCTMQIST------LDCNIPSLQQ-SKAI

QSSLHLL-DKIGQAPLQCRREHVPFNF-RNI-------------LKLR------------

QKDN--VKVA-----V-Q----EMLQSIFYMFS-KNL-TLAAWDGRSLESFQNGLYQQIE

QLEACS----------------LKY--D-NRL--------------K------LKKYFQR

IDN-FLKDKQYSLCSWEIIREEVRTCLQLI-ETVTKA-------------------

>Crpo_7

MEVSGLLQMFF------------------ILLCTMQIST------LDCNIPPLQQ-SKAI

QSSLHLL-DKIGQAPLQCRHEHVPFNF-RNI-------------LKLR------------

QKDN--VKVA-----V-Q----EMLQSIFYTFS-KNL-TLAAWDGRSLESFQNGLYQQIE

KLEACS----------------LKY--D-NRL--------------K------LKKYFQR

IDN-FLKDKQYSLCSWEIIREEVRTCLQLI-ETVTKA-------------------

>Chpi_15

-----LADESP------------------VTVLSVH---------------ASGQ-SSSG

RRLLAPL-MPLRSSPGSAW-----------------------------------------

RHQP--SKI---------------LQERFHIFN--NL-TQAPWNGTSIKEFQNGLHQQIE

KLETCL----------------SAYLLL-TSL--------------K------LKRYFQT

IDD-FLKEKQYSLYAWEIIRAEISRCLLIL-DIL----------------------

>Chmy_6

------------------------------------------------------------

----------------------------MAI-------------LKPR------------

EKVN--IVVT-----I-H----KILHETFNLFS-KNL--HAAWNTTCIEKFQNGLHWQIE

QLETCL----------------GANLQS-T-L--------------N------VKKYFQR

IKD-FLKEKHYSHCSWEQY-----FCLLSF-KKKSKN-------------------

>Almi_4

MEEPTFLHVCL------------------VLVFSIKISS------PDCS--RLQR-IKVN

-HSLYLL-CRMGGQPLSCLNDRTDFRI-REI-------------FIIR------------

KKEN--ALMI-----I-H----ELLHHIFQLFS-KNL-PQGAWNPSCIEKFQNGLHWQIE

QLEKCF----------------GGNLQN-NIL--------------K------AKKYFQR

ISH-FLNEKNYSRCSWETARMEMRRCFLFL-DHLLKN--LRN--------------

>Gavga_3

MEEPAFLHVCL------------------VLVFSIKISS------PDCS--RLRQ-IKVN

-QSLHLL-CRMGGEPLSCLNDRPHFRI-RQI-------------FTAR------------

NKEN--ALMI-----I-H----ELLHHIFQLFS-KNL-PQGLWNPSCIEKFQNGLHWQIE

QLQTCF----------------GGDLQN-NIL--------------K------VKKYFQR

ISH-FLNEKNYSRCSWETARMEMRICFLFL-DHLLKK--FRN--------------

>Crpo_1

MEEPTFLHVCL------------------VLVFSIKISS------PDCS--RLQQ-IKVN

-QSLHLL-CRMGGEPLSCLNDRTDFRI-REI-------------FTAR------------

KKED--ALMI-----I-H----ELLHHIFQLFS-KNL-PQGPWNPSCIEKFQNGLHWQIE

QLETCF----------------GGDLQN-NIL--------------K------VKKYFQR

ISH-FLNEKNYSRCSWETARMEMRRCFLFL-DHLLKK--FRK--------------

>Tyal_1

TDKTTLLRVCV------------------TLALYIKISH------PVC---LFQG-IKVN

YNNMNFL-WTMGGYSQQCLSEATDFRF-MEI-------------TKVT------------

QK-N--VTMI-----I-Y----EFLQQTFQLFS-KNL-PAGAWNTSKIQKFQNGIHQQIE

ELEVCL----------------LEILKS-TTF--------------S------VKKYFQR

ITD-FLKDKKYSHCSWEAVQMELRSCLIIF-DSLLKK--HTS--------------

>Apfo_2

-----LLRVCI------------------TLALYVKISH------PAC---LFQG-IRMN

YHNMNLL-CKMGGYSQQCPSETTDFRF-MEI-------------TKIT------------

QK-N--VTVI-----T-C----KFLQQIFQLFS-KNL-PVGAWNTSNIEKFQNGIHHQIE

ELETCL----------------SESLRS-TTL--------------S------MKKYFQR

ITN-FLKDKQXSHCSWEAVRMELRTCFIIF-DI-----------------------

>Stca_3

-----LLQVCI------------------TLALYTKISH------PVC---LFQG-SKVN

YQNMNFL-CKMGASPQQCLRERTDFKF-MEI-------------TKVR------------

QR-N--AIVM-----I-H----ELLRQIFHLFS-KNL-PESVWNASCIEKFQNGIHQQIE

ELETCL----------------VESLNS-TTL--------------R------VKKYFRR

ITS-FLENKQYSHCSWEAVRMEVRTCFIFI-DCLMRK--HMA--------------

>Chpi_6

------------------------------ML-----------------MDVFQE-----

--------QTSSEKP-----------P-RDA-------------F-IP----V------I

KRAN--YE-W-----L-H----EILQQIFNIFS-KNL-TQSAWDGTSIVRFQNGLYQQIQ

RLEACL----------------RANLQL-TSR--------------R------VKKYFQG

IDA-FLKEKQYSLCAWEIIRMEISRSFVLI-DKLTRS--LSN--------------

>Almi_7

-----LWKICL------------------VALLSAHVAA------LDCSNFRDLQ-KVLN

RNSMQLL-GQVAGAPEECLEDRPTFRF-DKV-------------LRSK------------

APHN--AWMA-----T-Y----EILQKLFSLFK-RTL-PETAWDTRSVERLLNAVHLQIK

RLETCP------------------Y----KAT--------------G------LKKYFRK

IDD-FLRAKNYSKCAWEVVRIEAKTWFYYL-DKLKNR--LN---------------

>Gavga_1

-----LWQICL------------------AALLLAHVTA------LDCSNLKDLQ-EVLN

RNSVQLL-GQVAGAPEECLEDRPTFRF-EKV-------------LRSK------------

APHN--AWMA-----T-Y----EILQQLFSLFK-RNL-PETAWDTRSMERFLNAVHVQIK

RLETCP------------------Y----KAM--------------K------LKKYFRN

IHD-FLQEKNYSKCAWEVVRIEAKTWFYYL-DKFRNR--L----------------

>Crpo_2

-----LWQICL------------------AALLLARVTA------LDCSNFKDLQ-EVLN

RNSMQLL-GQVAGAPEECLEDRPTFRF-EKV-------------LRSK------------

APHN--AWMA-----S-Y----EILQQLFSLFK-GNL-PETAWDMHSVERFLNAVHVQIK

RLETCL------------------Y----KAM--------------K------LKKYFRN

IHN-FLREKNYSKCAWEVVRIEAKTWFYYL-DKFGNR--L----------------

>Chmy_14

-----MAYQVP------------------TQP---------------------GM-TSIS

KSAFSAL-QK----PSSC------------------------------------------

------SK-----------------------------------NGSSIKVFQNGLHQQIE

KLETCL----------------SACLLL-TSL--------------K------LKRYFQT

IDD-FLKEKQYSRCAG---RSSVRKYPDVF-SFLTY--------------------

>Opha_5

-----TLKVCL------------------VLVLFAKSSV------FRCSIFHAQW-KQLI

QNNLPHL-CRTNEEPLQCIYELTDFNF-LEV-------------LEVT------------

NRDS--ADI------I-Y----ELLQQISYLLS-NA----HAWNSTCFENLKNGLHQQIK

NLETCL----------------NVNSYL-LTL--------------K------VKRYFQR

MNN-FLTVKQHSSCSWEMIHSEIKGCMLFI-THLLKK--L----------------

>Thsi_2

-----ILKVCL------------------VLVLLAKFLV------FRCSIFHAQW-KQLI

QNNLPHL-CRTNEEPLQCIYELTDFGF-LEV-------------LEVS------------

NRDN--AVI------I-Y----ELLQQISCLLS-NAP-SDHAWNSTCFENLKNALYEQMK

NLQACL----------------NAKSYL-LTL--------------K------VKRYFQR

MNN-FLTL------------------------------------------------

>Pybi_1

-----TLKICL------------------VLVLFAKLSV------FQCSIFHAQW-KQMI

QNNLHQL-CRANEEPLKCLPEVTDFRF-LEV-------------LKVS------------

NRGN--AII------I-Y----EILQQISCLLS-KGH-SYNVWNSTCFENLQNALHQQMK

TLATCL----------------NATSYF-LTL--------------K------VKKYFQR

MND-FLTVKQYSSCSWEMIHSEIKGCMLFI-IHLLNK--LKQ--------------

>Vibe_8

-----ILKFCL------------------VLVLFAKFSV------FRCSVFQAQW-MQLT

QNNLPHL-CR-NEIPPQCIYDLTDFRF-VEL-------------LQVN------------

NRDN--AVI------I-R----EFLNQIFYLLS-YAH-RYKVWNSTCFENVKIVLHQVIN

NLERCL----------------NANSYL-PKI--------------K------VKRYFKR

MIN-FLTLKQHSSCSWKIIHLEIEKCMMFI-TRLLEK--LKQ--------------

>IFNK_human

MIQKCLWLEIL------------------MGIFIAGTLS------LDCNLLNVHL-RRVT

WQNLRHLSSMSNSFPVECLRENIAFEPQEFL-------------QY-T----Q------P

MKRD--IKKA-----F-Y----EMSLQAFNIFS-QHT-FKY-WKERHLKQIQIGLDQQAE

YLNQCLEEDKNEED--------MKEMKENEML--------------E------LRRYFHR

IDN-FLKEKKYSDCAWEIVRVEIRRCLYYF-YKFTAL--FRRK-------------

>IFNK_dog

VIRKCLWPACL------------------VGLLITGVLS------LDCNLLHFHL-RKVT

WQNLRLLSSMSNSFPVECLREIKAFEPQEIL-------------SH-T----Q------P

VKRY--IVEA-----F-Y----EMSIQAFNIFS-QYT-FKSTWENDYLKQIQIGLDQQLQ

YAERCLEEEEKEDD--------SKEMEEDGIL--------------E------LRRYFNR

IDN-FLKEKKYSHCAWEIIRVEIRRCFYYY-FKFAPL--LRKK-------------

>Ptva_13

MIRKCLWPACL------------------MGLLITGILS------LDCNLLN-----RVT

WQNLKLLSSMRNSFPKDCLRENKAFEPQEIL-------------YS-T----Q------L

LKRD--IKEA-----F-Y----EISLQAFDIFS-QYT-FQSTWKKKYLKRIQIGLDRQLQ

YLEQCLEEEEKNED--------MKEMEEDESL--------------E------LKRYFHR

ISS-FLKDKKYSHCAWEIVRVEIRRCFYY----FTAL--LRKK-------------

>Dano_13

MIQKCLWPACL------------------MDLFITGILS------LDCDLLNVHL-SRVT

WQNLRVLRSMSNSFPLKCLRETEAFEPQEIL-------------SN-T----Q------P

VRRD--IKEV-----F-Y----EMSTQAFNIFS-QYT-FNSTWEEKHLKQIQIGLDRQIE

YVEQCLEDEEKNED--------MKQMEEDEML--------------E------LRRYFNR

INK-FLKDKKHSHCAWEIVLVEIRRCFCY--FKFTAL--L----------------

>Oror_5

VIRKCMWPVCL------------------MGLFVTGILS------LDCNLLNVHL-RRVT

WKNLSLLRRMSKSFPIECLRESKAFEPQEIL-------------SH-T----Q------P

LTRD--IKEA-----F-Y----EMSRQAFHIFI-QDT-FKSTWEEKHLRQVQIGLDQQLQ

YLEQCLEEEE-NED--------MREVAEDERL--------------E------LRRYFNR

IDR-FLKDKKYSHCAWEIVRVEIRRCFYF--FKFTAL--LRRK-------------

>Loaf_16

VIRKYFWPICL------------------VGLFLTSVLS------QSCDLLYVHL-NRVT

WQNLKLLSHMSNPFPVECLKEKKAFEPQEIL-------------SH-T----Q------P

VKRH--IEEA-----F-Y----EISSQVFNIFS-QHA-CKSAWDEKHLKQIQIGLHQQVE

YLERCLEEEEKSED--------MKQMEE-KIL--------------K------LRRYFNR

LGN-FLKDKKYSQCAWEIVLVEIRRCVFYY-FKFTTL--LRKK-------------

>Ereu_11

LIRKCLWSSCL------------------VYLFLTGIHS------LDCSFLNIQL-RRVT

GQNARLMSSMKGPLRQECLKDINNFEPEEIF-------------LC-N----Q------S

KKWN--IKVN-----F-Y----EIYANAFRIFS-QYT-VKYSWEEECMQQILMELNLQLE

SLEQCLKEEK----------------ENDEIL--------------K------LKRYFFR

IQS-YLRDKKYSDCAWKIVFVEIGRCFYHS-LKLTRL--SRKK-------------

>IFNK_mouse

MTPKFLWLVAL------------------VALYIPPIQS------LNC----VYL-DDSI

LENVKLLGSTMTGFPLRCLKDITDFKPKEIL-------------PY-I----Q------H

MKRE--INAV-----S-Y----RISSLALTIFN-LKG-SIPPVTEEHWERIRSGLFKQVR

QAQECFMDEEKE----------------REHL--------------E------LGKYFFR

IKK-FLINKKYSFCAWKIVTVEIRRCFIIF-SKSRKL--LKMK-------------

>Opha_1

MISGCFQHIVL------------------FLLLSSGIRS------LDCNHI-VKQ-QSRT

VNIMKLLESMG---PWQCFNKIQDFAP-NDTG---------------------------S

IKED--ARAT-----F-G----LMLEQINRMFW-QNF-TKAEWNVTVTEHLQTSLDQQLV

QWEKCV----AEGK------KATK---DRIKL--------------K------LRKYFLR

LDT-FLKDEEYSSCAWEAVRHEIMGIQVFL-DQLLRT--LQR--------------

>Thsi_6

MISGCFQYIVL------------------LLLLSSGIRS------LNCNHI-VKQ-KDET

MNTIKLLESMG---PWQCFNQIQDFAP-NDTG---------------------------S

IKED--ARAT-----V-G----LMLEQINRIFS-QNF-TKAEWNMTITEHFQISLDQQLV

QWEKCV----AEGK------KATK---DRTKL--------------K------LRKYFLR

LDR-FLKDEEYSSCAWEAVRHEIMGIQFFL-DQLLRT--LQH--------------

>Vibe_7

MISGCFQYVVL------------------LFLLSSGVRS------LDCNHI-VKQ-KGAT

VNIMKLLEAMG---PLECFHKIPDFAP-NNIG---------------------------S

IKED--AKAT-----I-G----LMLEQISRIFS-QNF-TQTEWNMTVAEHFQIALDQQIV

QWEKCV----TEGN------KATK---ERTKL--------------K------LRSYFLR

LDT-FLKDEEYSSCAWEAVRQEIKGIPLFL-DRLLRT--LQS--------------

>Pybi_3

MVLRCLQHIVL------------------LLLFSSGIVS------LDCNHI-VKQ-KGAT

ADIRKLLEAMG---SLECFHKIPDFNP-KNIG---------------------------S

IKED--ARAT-----V-G----LMLEHIQRIFW-HNF-TKAEWNMTVTELFQIVLDQQVV

QWETCV----TVGE------KATK---DRTKL--------------K------LKKYFLR

LDT-FLKDEEYSPCAWDVVRQEVLGIHFFL-DQLLRT--LQN--------------

>IFN9_anoli

--------MA-----------------------GQQ--S-------------SQ------

----------------ECLED--DFGPRKIL---------------------K-------

SQED--AKMA-----I-G----LILQQIQIVFQ-LNF-TQAQWSGKVTDLLSRALDQQHM

QWRRC-----A-----------TA---EAAKLR-------------S------LKRYFRK

LHT-FLRGRQYSFCAWKMVRYELLVIYPIL-NELMR---LEK--------------

>IFN1_ONMY

--------------MYTMQ--SW-SC---IFL----IICSMQSVCHCCDWIRHHY-GHLS

AEYLPLLDQMGG----DITKQ----------N--AP----VLFPTSLYRHIDD------A

EFED--KVIF-----L-K----ETIYQITKLFDG--NMKSATWDKKNLDDFLNILERQLE

NLNSCV---------------------S-PAMKP------ERRLKR----------YFKK

LSK-VLRKMNYSAQAWELIGKETKRNLQRL-DILAAQ--MY---------------

>IFNA1_SASA

--------------MYTVQ--SW-TC---ICL----IICSMQSVCHCCDWIRHHY-GHLS

SEYLSLLDQMGG----DITKQ----------D--AP----VFFPTSLYRHIDD------A

EVED--QVRF-----L-K----ETIYQITKLFDG--NMKSVTWDKKKLDDFLNILERQLE

NLKSCV---------------------S-PAMKP------EKRLKR----------YFKK

LKN-VLRKMNYSAQAWELIRKETKRHLQRL-DILAAQ--MY---------------

>Eslu_3

-----------------MQ--SW-IF---LFL----ILCRTQSSCSCCDWIRDHY-GTLS

REYLSLLDEMGG----NITKQ----------D--VP----VFFPESLYRLMED------A

QYEV--QVRF-----L-N----ETIHEIIKLFDE--NMDAVTWEEKKLDDFLILLHRQFQ

KLKSCV---------------------S-PAKKA------EGRLES----------FFKK

LKK-VLKEMNYSAQAWELIRKETKYVLEKL-YLLVAT--MHR--------------

>Anja_5

-------------------------------F----IVCSAQDFCDGCYWIQHGF-RRVS

GESLSLLSE---------------------------------------------------

MVDD--KVKF-----V-H----ASIDQIIKLFDE--NLDAVTWNRLKLEHFLIVLDRQSR

ELQKCV---------------------S-RCSII------RK------------------

--------------------------------------------------------

>IFN1_DARE

---------------------MW-TY---IFV----IYVILQSQSSACEWLG-RY-RIIT

TESLNLLKNMGG----KYA-D-------------LE----TPFPSRLYTLMDK------S

KVED--QVKF-----L-V----LTLDHIIHLMDAREHMNSVNWDQNTVEDFLNILHRKSS

DLKECV---------------------A-RYAKPAHKESYEIRIKR----------HFRT

LKK-ILKKKQYSAEAWEQIRRVVKSHLQRM-DIIASN--ARVN-------P-----

>IFN_CAAU

---------------MKTQ--MW-TY---MFV----MFLTLQGQCSACEWLG-RY-RMIS

NESLSLLKEMGG----KYP-E----------G--TK----VSFPGRLYNMIDN------A

KVED--QVKF-----L-V----LTLDHIIRLMDAREHMNSVQWNLQTVEHFLTVLNRQSS

DLKECV---------------------A-RY-QPSHKESYEKKINR----------HFKI

LKK-NLKKKEYSAQAWEQIRRAVKHHLQRM-DIIASN--RR---------------

>IFN_CTID

---------------MKTQ--MW-TY---MFV----MFLTLQGQCSACEWLG-RY-RMIS

NESLSLLKEMGG----KYP-E----------G--TK----VSFPGRLYNMIDN------A

KVED--QVKF-----L-V----LTLDHIIRLMDAREHMNSVQWNLQTVEHFLTVLNRQSS

DLKECV---------------------A-RY-QPSHKESYEKKINR----------HFKI

LKK-NLKKKEYSAQAWEQIRRAVKHHLQRM-DIIASN--RR---------------

>IFN_CYCA

--------------MNQTQ--MW-TC---IFV----IFLTLQSQCSACRWLG-RY-GTVS

ADSLNLLREMSG----QYP-E----------N--VK----MHFPGTLYNLIDK------A

EVED--QVRF-----L-V----LTLDHIINLMDASEHMNSAKWNLKKVEYFLEDLQRQSS

ELKECV---------------------A-QYQKPLQKESYEIRIKR----------HFRT

LKK-ILKKEKYSAQAWEQIRRAVRSHLQRM-DIIANN--AKKR-------V-----

>IFN2_ICPU

--------------MDIKL--SW-IC---LFL----LFFTVQERSEACNWMISQY-RAKN

DYCLSLLNEMGG----EIVPM----------T--GN----TSFPRRAYHEIEK------A

QAED--QVRF-----L-A----VATNEIIILFSAVSHVDDVKWDSRTLDNFLNILSRQLS

ELRNCT---------------------S-TYAERARRSSTEKKLRK----------HFKD

LRK-YLKNSNYSADSLEQIRSVVQRHLWRM-DTIAAI--VKQK-------LLRTN-

>IFN4_ICPU

--------------MDIKQ--SW-IC---LFL----LFFIVQERSEACNWMISQY-RAKN

NFCVSLLKEMGG----EIVQV--------------N----RPFPHKAYSEIDK------A

KAED--QVRF-----L-A----QATEQIISVFN-VSHVDEVKWDRSALDEFLNILNRQLT

ELTKCT---------------------S-TYAERAGHSPTERKLRK----------HFKK

LKK-FLNEANYSADSLERIRNVVQHHLWRM-DIIAAN--VKQK-------LLRTN-

>IFND1_ONMY

----------MHR-T--KS--LL-IC---LFL----TLCN-G-LSVGCRWMDHKF-IQHS

ETLMNVLNIMGG----EFTTD----------S--VD----VPFPEDLYEQAEY------L

PTDD--TIWF-----I-L----QTLDKIAELFD-GEL-NSV-WDEKKVEIFLNVLTSQSD

GLQSCV---------------------R-AQKKN----------SKN------LQMYFKR

LNNHVLKRMAYSAHAWELVRKEVRTHLRRL-VLLGSA--TENR-------I-----

>IFND1_SASA

----------MHR-T--KS--LL-IC---LFL----TMCD-G-FSMGCRWMDHKF-IQHS

ETLMNLLNIMGG----EFTTD----------S--VD----VPFPEDLYKQAEY------L

PTDD--TIWF-----I-L----QTLDKIAELFD-GEL-DSV-WNEKKVEIFLSVLNSQSD

GLQSCV---------------------T-AQKKN----------SKN------LQMYFKR

LHNQVLKRMAYSAHAWELVRKEVRTHLMRL-VLLGSA--TENS-------I-----

>Eslu_2

--------------------------------------------------M-------HS

ASSLGFLDIMGE----DIPKD----------S--VK----IFFPEDLYKQADC------S

PADD--QIWF-----I-L----QTLDEITKLFS-DKC-YSV-WGEKTVDNFLGVLSSQVD

GLQSCI---------------------T-SQKKR----------SKN------LHKYFKR

LNNDILKSMEYSPHAWEMVRKEVRTHLKRL-TLLGSA--PDNK-------LVQQ--

>IFN_SPAU

--------------M--LNRIFF-VC---LSL----SLYSAG-SSLSCRWMDHKF-RQHS

KNSLALLDTMAN----NSTNT----------TEDAEVEDTVAFPNLLYRQASK------A

SAED--QLAF-----T-V----QILDETAALFE-EDH-SSASWEENTVENFVNVVTQQAD

ELRSCI---------------------G-SHKKK----------NKK------LHMYFQR

LSSHVLKRMGHSAEAWELIRGEVKVHLMRA-NQLVTSATRTN--------------

>IFN_DILA

--------------M--LNRIFF-VC---LSL----SLYSAG-SSLSCRWMDHKF-RQHS

KNSLALLDTMAN----NSTNT----------TEDAEVEDTVAFPNLLYRQASK------A

SAED--QLAF-----T-V----QILDETAALFE-EDH-SSASWEENTVEDFVNVVTQQAD

NLRSCI---------------------G-SHKT-----------NKK------LQMYFMK

LSSHVLKKMGHSAEAWELIRKEIKTHLMRA-DQLVSSLLTTN--------------

>IFN1_OPFA

--------------M--LSRTFL-VC---LFL----SLCSAG-SSLSCRWLDHKF-RQHS

ENSLDLLDTMVN----NSTNT----------TEDAGVKDTVAFPNELYSQASK------A

AAED--KLGF-----T-V----QVLEETAALFE-EDH-SSASWEENTVENFVNVVTQQAD

GLRSCI---------------------G-SHKKK----------NKK------LHMYFKR

LSSHVLEQMGHSAEAWELIRNEIQTHLMRA-DLLVSSLLTTN--------------

>IFN_PAOL

--------------M--LNRIFF-VC---LCL----CLYSAG-SAMSCRWMDHKF-RQYS

KNSLDLLDMMAH----NSINT----------TEDVEVEETVALPEHLYSQVSN------A

SAED--RLGF-----T-V----QVLKEVAALFE-EDS-SFASWEEKQMDDFLNIVTQQAD

GLRSCI---------------------V-SHSHK----------NKK------LHMYFKR

LSRHVLKQMDYSVESWELIRKEIKNHLMRS-DVLISSLLTIN--------------

>IFN2_ORNI

--------------M--MNRILF-AC---LFL----GLFTVG-SSLSCKWMDDKF-KQHN

EETLNLLDTMGN----NSTNT----------T---EVEDTVAFPNHLYRQASK------A

SAED--KLAF-----T-V----QVLEEVAALFE-EDH-SSASWEDSTVRNFLNIVNKQAE

ELHSCI---------------------G-SHSHK----------KKK------TEMYFKR

LSDDVLKKKGHSAEAWEVIRKETKAHLMRL--TLIKSRGTTQS-------L-----

>IFN_ORLA

--------------M--LHRLVF-AC---ALV----SLAGAG-FSLRCRWLDHKF-KQFS

DTSLDLLEKMVN----NATNS----------TEGDATEDIVDFPHHLYRQASK------E

SAEN--QVAF-----T-V----QVLKEVSALFE-EDS-SSASWQQITVEKFLGVVNRQAD

ELHSCV-----------------------SESKK----------NRK------LRMYFKR

LLDHILKKQGYSAEAWETIRKETKAHLLRA-QRLLSPLISSK--------------

>IFN1_ORNI

--------------M--ISRIFI-AC---LFL----GMYSTG-SSLSCKWI-----VKHP

GNTLALLCIMFN----VITNT----------TKDAEIEHNVAFPNRLYRQTSK------A

TAED--KLAF-----T-V----QILKELLALFE-EDH-SSASWEENTVENFLNIVDKQTE

ELHSCI---------------------G-SHSNT----------QKR------REKYFKR

LLNKILKKNGYSAEAWEKIRNITQAHLRQC--EFLISLRTAH--------------

>IFN_TARU

--------------M--LP--LL-VC---LSL----CVYSQG-SPLGCRWLDDKF-RQYS

HKSLELLDTMVN----NSTNS----------S--VEPEEMVIFPQELYRQTFN------A

SAED--KLAL-----A-A----QIMNETVALLM-EDH-SGASWDEKQVENLVNVLTQQAD

NLQACM---------------------V-SPGHK----------SEE------VERYFNR

LSNHILKKMDYSAAAWELIREEIETLLMQT-HLLVSTLLSTP--------------

>IFN_TENI

--------------M--LT--VL-LC---LSL----CVCSQG-SPLGCRWLEEKF-TQYS

SLSLSLLDNMKS----NSTNS----------S--LEAEDTAIFPEVLYRQTFN------A

SAED--RLAF-----A-A----QILNETAALFE-EDY-SGASWEEKSVENFVNILTQQAD

NLGSCV---------------------A-SPGQS----------SKE------LHKYFTR

ISTHILRKTDHSAGAWELVREKIRSLLMRA-HLL----LTTH--------------

>IFNa1_gaac

--------------M--TSWTSM-LV---LLT----LLCSAGTPGLCCDWL-QHY-GHLS

NVSLTLVQTMGN----QLTDE----------E--SP----VSFPYRLYERIMN------D

KEDN--QLVF-----I-R----DSLELMAKLYR-HDNRSSVTWDANKMERFLMIIHRQIH

GLNLCV---------------------S-T--Q----------ITRR------LRRYYRR

LEKKTLYSTGGSPASWELIRKESKLHLDQL-NQLWGFMV-----------------

>IFN4_DARE

--------------M--KV--FA-AA---QFC----VLLSVG-FSLGCRWVKHRL-QHHH

GVSLDLLRKMGE----KVHDD----------N-----EDLNPIPYDLINNHRM------A

EPEK--QIQF-----V-I----QALVEITALFD-DAL---VPWDAKKMDDFLNIMHEEID

GLRSCG---------------------S-YKMKR----------NKK------LHLYFNR

-----LRRMTDGGRSWEMVRKRVIS-LMNQ-LHSFSF--HTH--------V-----

>Anja_7

---------V--------------------------------------------------

-------------------EE---------------------------------------

------RIIF-----I-H----EVINNIKDLYIKGKY-DTVTWDPKKLQMFQLNLHRQAS

ELKEC------------------------------------------------VRPNFAP

PQN-AGHDKEYR--------------------------------------------

>IFNF2_ONMY

--------MATLN----VS--FV-VH---LLC----VIV------LKCSDQKEQM-YNLS

QTRQTLNDLAMERRPRGCIPEAEMIMVQ--R-------------PTLS----K------E

EVEKVWTLRL-------------AFQLASELFQ-QNL-TLVKWNSIKLRDLQDLLARQ--

----------------------YMTVRD-MRLRQ----------NLP------IKNYFKQ

LDD-FLSRESFSLCSWEVVRTEMGSIL-------RDF--YKKS-------KMRKHV

>IFNF1_ONMY

--------MATLN----VS--FV-VH---LLC----IIV------FKCSAPKVQK-YYLS

QTHQTLNDLAEERLPRGCIPEAERLRVQ--R-------------PALP----I------E

EGEKVWTLRL-------------AFQLASELFQ-QNL-TLVKWNSVKLRDLQDLLARQ--

----------------------NMTVRD-MSVHL----------NLP------IKNYFKE

LED-FLSHERFSACSWELVRAEMGSII-------SQA--IRNA-------K--KHV

>eslu_f

--------MAAHN----VT--FV-VH---LLF----ALA------LTCCD---ET-YYIF

KTRQVVNDLAMGRKPV-CVQEAARIRVH--R-------------PTLS----L------E

VGERFWTLSL-------------VFHLACELFQ-RNL-TLVKWNVNQLRELQELLARQ--

----------------------NRTVKD-IRLGQ----------SLP------NKKYFKQ

LDD-FLSRETFSLCAWEVVRFEMGRIL-------RDF--HKKS-------N-SKKT

>Eslu_4

--------------MYSIR--LC-MS---LVL----MICSCN-ETMGCTWMRSMF-KSFI

SKSITVLQEKD-------DGE-------------PL----ISLPNKLYRQFDD------L

KADD--QIVF-----I-S----RTLKAIMHLYSSGKY-ES-TLETERIDTFIHYLSRQTM

ELDQCI---------------------K---AMNPTLSKSVKRANKK------MNSHFKF

LKN-YLKGEEFNGKAWIEIKRVVLAHLRRI-V--LT--------------------

>Opha_4

MAATSMGLLCL------------------VLLLAVPA-LG-----LHCNLLKWQQ-QRLN

QQSVELLKGMK---SPVCL--RKEVTP-QIL---------------------R------I

QRPRA-AKAI-----L------EMLHGFLHLFK-EDH---VAWDATLRKRFLPSFSAPV-

----CI----------------SI------------------------------------

---------------------QVVLDLQWF-T------------------------

>Thsi_4

MPSKRMGLLCL------------------VLLLAAPT-WG-----SNCNLLKLHQ-RRLN

RQSVELLRRVK---PAECL--RKVFSP-KIL---------------------G------I

REPRE-AKAV-----L------EVLQGFLHILK-DEH---VAWKATLQKRFLPMLHAQIQ

RIQGCL----------------GE-GRKEEKL--------------Q------LKKYFRS

IGN-FLEENGLDSCTREFVRHEIQLDFIYL-DRLTER--ME---------------

>Pybi_6

MSATPTELLCL------------------VLLLSAQV-TA-----LNCNFLKLQQ-QRFN

RHSVELLKGMS---PQECL--RKTSNP-TVL---------------------E------I

HQPQI-AKTI-----L------EMLHGFLNILS-DDC---NAWEAKLRNR----------

---KCV----------------SA------------------------------------

--------------TVQFPLTELRCPFTYF-QVEAEE--ND---------------

>IFN1_frog

--------MS--------------VS---VLL----LIT------LGSSGQPTKG-KDVY

RTQLNIN------------REVRTLLGN--M-------------GAIP----Y------S

ECEDNWRLQI-------------TIHQFSVIFT-DNL-A----NSVEMSKMQTLLYWY--

----------------------STSLKE-LTLKE----------TKK------IRRYFRK

MLK-YLMKKGYSRCAWASVRDEMEKVLLLV-TWHTDI--LLKK-------HLRGHV

>IFN4_cami

--------MSLP-----------------VLLLCSPT-SGHG---SDC--M---Q-RRYW

RDLLESLN------PVACRGE--DVRE-PVML--------------------N------P

VKKD--KAAV-----T-L----QVLEEILRLFR-K---STVPWTNSK---FLNQVYQVIY

ELQNCM------TS--------S-----DLPVRS------------T------IKARFAN

LEG-FLNEKSI--CAWEIVHLETRKILQ----EVSRN--HARK-------------

>IFN5_cami

--------MSLP-----------------VLLLYSPT-SGHG---SDC--V---Q-RRYW

RDLLESLN------PEECRNE--DINP-APIS--------------------K------L

AKQN--KAAV-----I-V----QVLEEVLKLFS-K---PDAPWSNSKALTFLNKVSQILS

DLQSCM------QS--------P-----DSRVRS------------T------VIERFAK

LER-FLKAKSV--CAWEIVHAETRKIFQ----QVEQR--HARK-------------

>New|Hosa_G

MKTSYILAFQL-----------------CIVL-----------GSLGC-YCQDEA-----

----------------ENLKKYFNAGHSDVAD------NGTLFLGILK----N------W

KEES--DRKM-----Q-S----QIVSFYFKLFKNFKD------DQSSVETIKEDMNVKFF

N--SNK----------------------------------------K------KRDDFEK

LTNLNVQRGRRASQ------------------------------------------

>New|gaga_G

MTQTYNL-FVL-----------------SVIM-----------SSLNV-QLQDDI-----

----------------DKLKADFNSSHSDVAD------GGPIIVEKLK----N------W

TERN--EKRI-----L-S----QIVSMYLEMLEN--T------DKSHIKHISEELYTLKN

NLPDGV----------------------------------------K------KVKDIMD

LAKLRIQRQRRCNC------------------------------------------

>New|cami_G

MKLCFIIGLVT-----------------CLLL-----------GSASCDRLQDEI-----

----------------NNLKSHFDTSSHEVAD------GGPLFLNMLE----K------W

KGSP--ESGV-----F-H----RILLWYENFFKNIKGAPGKELDISNIGNLISKWIVEDR

Y--KPV----------------------------------------E------LKYDLDK

LDNQLVQRRRRSRT------------------------------------------

1. **IFN1 EXT:**

>oran_L3

MR---GHLLTG------------------AQAADPP--K------KQCYL--GKF-KSLS

PQELEAFKKAKDML-------------------------------LTD----R------T

ERLL--FLEA-----E-L----KLLG---------KM-SKSNLEGYLGRPLWTLRYIS-Q

ELQRCI----------------AQES-R-HSS--------------R------LTHWLHK

LQE-AREK------------------------------------------------

>gaga_L

MV---GVTIVG------------------AFPQVTP--K------KSCSL--SKY-QFPA

PLELKAVWRMKEML-------------------------------LTN----R------K

DRIT--LVEA-----E-L----DLLT---------NP-TTQRLAETCQQPLAFLTQVQ-E

DLRDCL----------------EAPSHQ-PSG--------------K------LRHWLQK

LET-AKKKET-------------AGCLLRC-AAQRED--CT---------------

>pesi_L3

TM---GYRVAE------------------AFPKGAL--R------TKCHL--AKY-KSLP

PRELEAFKKTKDLL-------------------------------LSD----R------K

DRVI--LVEK-----E-L----DFLE---------DV-EDPSLSKLLPRPLEILSQIR-E

DLRRCT----------------QTPSHS-HSK--------------R------LNSWLQN

LQA-SKETET-------------PACLLRC-AAYTEL--CV---------------

>gavga_L

------------------------------------------------------------

----------------------------------------------------R------K

DRVI--LVQA-----E-L----NFLK---------NI-EDPNLSEQLPRPLEFLTHIG-E

DLKSCT----------------HHHSHK-KSE--------------K------LSSWLQK

FHE-AKNKET-------------RECLLAC-AALKDD--CIS-------P------

>chpi_L2

---------VD------------------AFAEGAP------------------------

------------LR-------------------------------DPA----A------N

QNLL--VLEK-----K-V----DVIQ---------NL-SDPELVRNASKPLEILASIQ-E

DLRSCP----------------QQPV---------------------------LARWLRN

AYA-GKSEGS-------------ARCLLQN-VAHNES--CH---------------

>oran_L4

---------LE------------------VSCSSFM-DG------KKCYL--AHY-GSLD

PQVLRDVKDLQN------------------------------------------------

GRLL--LLER-----E-L----AHLR---------NL-SGLDLGRNATRPLQLLAAIC-E

DLASC----------------------S-ARQ--------------R------EMSWLRS

KAK-AKKEVT-------------PRCLLRQ-ATYLKP--CD---------------

>New|ANQ43341.1_type_III_IFN_7_Xenopus_laevis

------------------------------------------------------------

------------------------------------------------------------

DRLI--LTLE-----R-V----SLLQ---------NV-TESPLTTLVSQPLTMFLSLE-D

DLKFCR--------------KSPKYSDP-PSP--------------K------LMPWLNH

LKN-FRERVP-------------TECVVMC-WANSE--------------------

>New|ANQ43340.1_type_III_IFN_6_Xenopus_laevis

MAMMVLLVTVT------------------AHP-----HR------RHCHM--SRY-RSLS

PSDIRAVRLLHNPF-------------------------------SDG----I------K

DKQI--LTLE-----R-V----TLLT---------NM-TESSLSEFVSQPLEFFRSLE-D

DLKHC-------------------------------------------------------

--------VS-------------VQCVVMC-WANKE--------------------

>New|ANQ43339.1_type_III_IFN_5_Xenopus_laevis

MAMMVLLVTVT------------------AHP-----HR------RHCHM--SRY-RSLS

PSDIRAVRLLHNPF-------------------------------SDG----I------K

DRHI--LTLE-----R-V----TLLT---------NM-TESPLSEFVSQPLEFFRSLE-D

DLKHCR--------------KSPFYSDS-PSQ--------------Q------LMPWLNH

LKH-FRERVS-------------AQCVVMC-WANKE--------------------

>New|ANQ43337.1_type_III_IFN_3_Xenopus_laevis

MAMMVLLVTVT------------------AHP-----HR------RHCHM--SRY-RSLS

PSDIRAVRLLHNPF-------------------------------SDG----I------K

DKQI--LTLE-----R-V----TLLT---------NM-TESPLSEFVSQPLEFFRSLE-D

DLKHCR--------------KSPYYSDS-PSQ--------------K------LMPWLNH

LKH-FRERVS-------------AQCVVMC-WANKE--------------------

>New|ANQ43336.1_type_III_IFN_2_Xenopus_laevis

MAMMVLLVTVT------------------AHP-----HR------RHCHM--SRY-RSLS

PSDIRAVRLLHNPF-------------------------------SDG----I------K

DKQI--LTLE-----R-V----TLLT---------NM-TESPLSEFVSQPLEFFLSLE-D

DLKHCR--------------KSPLYSDS-PSQ--------------K------LMPWLNH

LKH-FRERVS-------------SQCVVMC-WANKE--------------------

>New|ANQ43303.1_type_III_IFN_5_Xenopus_tropicalis

MAMMVLLVTVT------------------AHP-----HR------RHCHM--SRY-RSVS

PSDIRAVRRLHNSF-------------------------------IDG----I------K

DRII--LTLE-----R-V----TMLT---------NI-TESPLSEFVSQPLEFFRSLE-D

DLKHCR--------------KSPLYSDP-PSQ--------------Q------LMPWLNH

LKH-FRERVS-------------SQCVVMC-WANKE--------------------

>New|ANQ43299.1_type_III_IFN_1_Xenopus_tropicalis

MAMMVLLVTVT------------------AHP-----HR------RHCHM--SRY-RSVS

PSDIRAVRRLHNPF-------------------------------SDG----I------K

DRLI--LTLE-----R-V----TMLT---------NM-TESPLSEFVTQPLEFFHSLE-D

DLKHC-------------------------------------------------------

--------VS-------------SQCVVMC-WANKE--------------------

>New|ANQ43335.1_type_III_IFN_1_Xenopus_laevis

MAMMVLLVTVT------------------AHP-----HR------RHCHT--SRY-RSLS

PSDIRAVRLLHNST-------------------------------NDG----I------K

DRLI--LTLE-----R-V----TLLT---------NM-SESPLSEFISQPLEFFRSLE-D

DLKHCR--------------KSPLNSDP-PSQ--------------Q------LMPWLNH

LKH-FREKVS-------------SQCVVMC-WANKE--------------------

>New|ANQ43342.1_type_III_IFN_8_Xenopus_laevis

MAMMVLLVTVT------------------AHP-----HR------RHCHM--SRY-RSLS

PSDIRAIRLLHNPF-------------------------------SDG----I------K

DKQI--LTLE-----R-V----TLLT---------NM-TESPLSEFVSQPLEFFRSLE-D

DLKHCR--------------KSPLYSDS-PSQ--------------K------LMPWLNH

LKH-FRERVS-------------AQCVLLA-ESNPNP-------------------

>napa_LR1a

---------VS------------------GRL-----HK------RLCPM--SRY-LSVA

SSDITTLKQLQHMS-------------------------------SNA----M------R

DRLI--LTLE-----R-V----SLLT---------NM-SMSAQPDTIKQSLMVFLKLR-D

DLLVCR----------------PEYSEP-TSP--------------E------LKLWLHH

LQR-FKETAS-------------PDCVVTC-WALNQ--------------------

>New|ANQ43302.1_type_III_IFN_4_Xenopus_tropicalis

MAMMVLLVTVT------------------AHP-----HR------RHCHM--SRY-GSVS

PSDIRAVRRLHNVS-------------------------------SQC----V------Q

DPVL--LSLT-----Q-L----LIED---------VM-----------------------

------------------------------------------------------------

-----------------------------C-WANKE--------------------

>New|ANQ43344.1_type_III_IFN_1_Xenopus_laevis

-------MYLTAAVF----FALLTTLGA-GHP----THR------ERCPL--SSY-QTVL

PSDITAARQLQNQT-------------------------------RVS----L------K

GRLR--LTLH-----R-V----SLLK---------NM-TKS---KLVLRNLELFGALH-E

TLL-CN----------------TSHSDP-THP--------------V------LESCTDH

LVT-YTKDTS-------------AGCIILV-WLLIED-------------------

>New|ANQ43305.1_type_III_IFN_1_Xenopus_tropicalis

-------MYLTAAVV----FTLLPTLG--GHP----THR------ERCPL--SGY-ETVL

PSDIIAAKELQNQT-------------------------------RMS----L------K

GRLM--LTLH-----R-I----SLLK---------NM-TKS---KLVLRNLELFGALQ-E

TLL-CN----------------TSHSYP-NHP--------------V------LESCAGL

LAT-YTKETP-------------AECIVLV-WLLIED-------------------

>scca_L

-------MATF------------------LAPSLSP-GQ------DGCSL--SRY-ARLP

PSVFKLFGDFHRQE-----PG---------------------------------------

ERLL--LVEA-----E-F----RLMQ---------EL-ETSILQNLTKKVLEVLYQMC-W

NLGRCL----------------TQKDQK-YPK--------------Q------LKKFLRN

LKH-AGRLGR-------------T---LSC-VASGED--C----------------

>Xetr_4

--------MLL------------------SLTSIVH--S------QSCKWLHPKQ-EYLN

TQILKAFNEMMPLK-------------------------------ETE----E------I

SQVE--AGAL-----A-L----NEYM---------KH-HESMGCKQQAERFQQLLYYQIH

QLEACV----------------TEENDL-LKE--------------S------ISEEFNL

LET-MVLEKN-------------SACVLRR-QRLLQR--PQ---------------

>New|ANW82729.1_type_I_IFN_5_Xenopus_tropicalis

MMGQWSVLLLL------------------SLTSIVH--S------QSCKWLHPKQ-EYLN

SQILKAFNETFTVR-------------------------------ETD----E------I

SQVE--AGVL-----I-V----REYM---------KH-HESMGCKQQAERFQQLLYYQIH

QLEACI--------------PKASENHL-IKE--------------T------ISEEFNL

LEK-IVLGKN-------------SACVLRR-QRLLQR--PQ---------------

>New|ANQ43259.1_type_I_IFN_4_Xenopus_tropicalis

MMGQWSVLLLL------------------SLTSIVH--S------QSCKWLHPKQ-EYLN

TQILKAFNEMMPLK-------------------------------ETE----E------I

SQVE--AGAL-----A-V----REYM---------KH-HESMGCKQQAERFQQLLYYQIH

QLEACV--------------SQTEENDL-LKE--------------S------ISEEFNL

LET-MVLEKN-------------SACVLRR-QRLLQR--PQ---------------

>New|ANQ43261.1_type_I_IFN_6_Xenopus_tropicalis

MMGQWSVLLLL------------------SLTSIVH--S------QSCKWLHPKQ-EYLN

SQILKAFNEMIALE-------------------------------KND----K------I

SQVE--AGAL-----A-V----REYM---------KH-HESMGCKQQAERFQQLLYYQIH

QLEACV--------------SQTEENDL-LKE--------------S------ISEEFNL

LET-MVLEKN-------------SACVLRR-QRLLQR--PQ---------------

>New|ANQ43262.1_type_I_IFN_7_Xenopus_tropicalis

MMGQWSVLLLL------------------SLTSIVH--S------QSCKWLHPKQ-EYLN

TQILKAFHEMKCLE-------------------------------ENV----N------I

SQVE--A--------T-V----KEYM---------KH-HESMGCKQQAERFQQLLYYQIH

QLEACV--------------SQTEENDL-LKE--------------S------ISEEFNL

LET-MVLEKN-------------SACVLRR-QRLLQR--PQ---------------

>New|ANQ43312.1_type_I_IFN_7_Xenopus_laevis

MMGQWSVLLLL------------------SLTSIVH--S------QSCKWLHPKQ-EYLN

TQILKTFNETIPIR-------------------------------ETE----K------I

SQVE--AAAL-----A-V----REYM---------KH-HERMGCKQQAERFQHLLYYQIH

QLEGCI--------------SETAEDHL-IKE--------------S------VSEQFNL

LEK-TILEKS-------------SACVLRR-HHLIQR--TQ---------------

>New|ANQ43309.1_type_I_IFN_4_Xenopus_laevis

MMGQWSVLLLL------------------SLTSIVH--S------QSCKWLHPQQ-EYLN

SQILKAFNQTIPIR-------------------------------ETE----K------V

SQVE--AAAL-----A-V----REYR---------KH-HESMGCKQQAERFQQLLYYQIH

QLEGCI--------------PETAENHL-IKE--------------S------VSEHLNL

LEK-IVLEKN-------------SACVLRR-QHLLQR--TQ---------------

>New|ANQ43308.1_type_I_IFN_3_Xenopus_laevis

MMGQWSVLLLL------------------SLTSIVH--S------QSCKWLHPQQ-EYLN

SQILKAYNQMNNLE-------------------------------ESD----K------T

LQVE--TAAL-----A-V----REYR---------KH-HESMGCKQQAERFQQLLYYQIH

QLEGCI--------------PEMAENHL-IKE--------------A------ISEEFHL

LEK-MVLEKN-------------SACVFRR-QHLLQR--TQ---------------

>New|ANQ43311.1_type_I_IFN_6_Xenopus_laevis

MMGQWSVLLLL------------------SLTSIVH--S------QSCKWLHPKQ-EYLN

TQILKTFNQTISLR-------------------------------END----E------I

SQVE--AAAL-----T-V----REYM---------KH-HESMGCKNQAERFQQLLYYQIH

QLEGCI--------------PETAENHL-IKG--------------A------VFEEFNL

LEK-IVLEKG-------------VSIV-----------------------------

>New|ANQ43260.1_type_I_IFN_5_Xenopus_tropicalis

MMGQWSVLLLL------------------SLTSIVH--S------QSCKWLHPKQ-EYLN

SQILKAFNETFTVR-------------------------------ETD----E------I

SQVE--AGVL-----I-V----REYM---------KH-HESMGCKQQAERFQQLLYYQIH

QLEACI--------------PKASENHL-IKE--------------T------ISEEFNL

LEK-IVLGKR-------------NAEILGR-RDTGKN--PDGPQ------------

>New|ANQ43258.1_type_I_IFN_3_Xenopus_tropicalis

TMASIQTILLL------------------VLIPIVQ--S------QNCKWLQPKQ-EYLN

RQTLKTFEEMNPPE-------------------------------DYD----E------S

SQME--EMVL-----A-V----RGYM---------KH-HESMGCKQQAERFQQLLYYQIN

QLEACI--------------PETAENPV-FNQ--------------T------ISDQYQA

LEQ-ILQEKN-------------TACTARR-QRLLQR--TA---------------

>New|ANQ43307.1_type_I_IFN_2_Xenopus_laevis

MMLSIKTFLGL------------------VLISIVQ--P------KTCKWLRPKQ-EYLN

SQILKTFEEMSPFE-------------------------------DYD----E------T

SQVE--AAAL-----A-V----REYR---------KH-HESMGCKQQAERFQQLLYYQIH

QLEGCI--------------PETAANPV-FNQ--------------T------LSEQFAV

LEK-FIQEEN-------------TPCLARR-QHLLQG--TA---------------

>New|ANQ43310.1_type_I_IFN_5_Xenopus_laevis

IVAQSYLHCPL------------------PKLQMAS--P------QTGISQHPNS-PNLQ

PIEFDIVCHDYPLE-------------------------------RLY----N------I

TQVE--AAAL-----A-V----REYR---------KH-HESMGCKQQAERFQQLLYYQIH

QLEGCV----------------PEENDL-LKN--------------K------ISEQFQQ

WEK-NVAEQW-------------S--------------------------------

>Napa_2

---------------------------------MVS--A------QTCKWLHRNQ-EAWT

RQILHNFNQMVPAE-------------------------------KTG----Q------P

TQAE--SAAI-----A------DEHI------------------------------YQLA

RPSRCC----------------TAEGKY-VSY--------------T------VNQGREA

VKP-VLA--------------------VSR-YILLVK--PQL--------------

>New|ANW82732.1_type_I_IFN_3.1_Xenopus_tropicalis

-MNRTLMVPLL------------------LVLSSGG--S------HGYPLLKKGG-EHLL

NEMLMKNDRLMLEE---------------------------------I----H------E

YQVE--ASVA-----A------IQYN---------EN-FEALGLSQELHELLQILDRLIE

EWAPYV----------------TDVNED-VSQ--------------G------ISEFYRQ

LIK-LVPKPN-------------AACVCQK-HLLVNE--TSR--------------

>New|ANQ43273.1_type_I_IFN_11_Xenopus_tropicalis

MMNRTLMVPLL------------------LVLSSGG--S------HGYPLLKKGG-EHLL

NEMLMKNDRLMLEE---------------------------------I----H------E

YQVE--ASVA-----A------IQYN---------EN-FEALGLSQELHELLQILDRLIE

EWAPYV----------------TDVNED-VSQ--------------G------ISEFYRQ

LIK-LVPKPN-------------AACVCQK-HLLVNE--TSR--------------

>New|AWK27016.1_IFN_3.4_Xenopus_tropicalis

-MSRTLTVPLL------------------LVLSSGG--Y------HGYPLPKKGG-EHLL

NEMLMKNDRLVLEE---------------------------------I----H------E

YQVE--ASVA-----A------IQYK---------EN-FEALGLSQELHELLQILDRLIE

EWAPYV----------------TDVNED-VSQ--------------G------ISEFYRQ

LIK-LVPKPN-------------AACVVQK-HLLVNE--TSR--------------

>New|ANQ43269.1_type_I_IFN_7_Xenopus_tropicalis

-MSRTLTVPLL------------------LVLSSGG--Y------HGYPLPKKGG-EHLL

NEMLMKNDRLVLEE---------------------------------I----H------E

YQVE--ASVA-----A------IQYK---------EN-FEALGLSQELHELLQILDRLIE

EWAPYV----------------TDVNED-VSQ--------------G------ISEFYRQ

LIK-LVPKPN-------------AACVVQK-HLLVNE--TSR--------------

>New|AWK27017.1_IFN_3.5_Xenopus_tropicalis

-MSRTLTVPLL------------------LVLSSGG--Y------HGYPLPKKGG-EHLL

NEMLMKNDRLVLEE---------------------------------I----H------E

YQVE--AAVA-----A------IQFN---------EN-FEALGLSQELHELLQILDRLIE

EWAPYV----------------TDVNED-VSQ--------------G------ISEFYRQ

LIK-LVPKEN-------------AACVVRK-HLLVNE--TSR--------------

>New|ANQ43264.1_type_I_IFN_2_Xenopus_tropicalis

-MSRTLTVPLL------------------LVLSSGG--Y------HGYPLPKKGG-EHLL

NEMLMKNDRLVLEE---------------------------------I----H------E

YQVE--AAVA-----A------IQFN---------EN-FEALGLSQELHELLQILDRLIE

EWAPYV----------------TDVNED-VSQ--------------G------ISEFYRQ

LIK-LVPKEN-------------AACVVRK-HLLVNE--TSR--------------

>New|ANQ43292.1_type_I_IFN_30_Xenopus_tropicalis

------------------------------------------------------------

--MLMKIDRLVLEE---------------------------------I----H------E

YQVE--AAVA-----A------IQFN---------EN-FEALGLSQELHELLQILDRLIE

EWAPYV----------------TDVNED-VSQ--------------G------ISEFYRQ

LIK-LVPKEN-------------AACVVRK-HLLVNE--TSR--------------

>New|AWK27015.1_IFN_3.3_Xenopus_tropicalis

-MSRALMVPLL------------------LVLSSGG--S------HAYPLLKKGG-EHLL

NEMLMKNDCLAPEE---------------------------------K----H------E

YQVE--AAVA-----A------IQYN---------EN-FEALGLSQELHELLQLLDRLIE

EWAPYV----------------TDVNED-VSQ--------------G------ISEFYRQ

LIN-LVRKEN-------------AACVVQK-HLLVND--TSR--------------

>New|ANQ43270.1_type_I_IFN_8_Xenopus_tropicalis

MMSRALMVPLL------------------LVLSSGG--S------HAYPLLKKGG-EHLL

NEMLMKNDCLAPEE---------------------------------K----H------E

YQVE--AAVA-----A------IQYN---------EN-FEALGLSQELHELLQLLDRLIE

EWAPYV----------------TDVNED-VSQ--------------G------ISEFYRQ

LIN-LVRKEN-------------AACVVQK-HLLVND--TSR--------------

>New|AWK27014.1_IFN_3.2_Xenopus_tropicalis

-MSRTLTVPLL------------------LVLSSGG--S------HAYPLLKKGG-DHLL

NEMLMKNDRLAPEE---------------------------------I----H------E

YQVE--AAVA-----A------IQYN---------EN-FEALGLSQELHELLQLLDRLIE

EWAPYV----------------TDVNED-VSQ--------------G------ISEFYRQ

LIK-LVRKEN-------------AACVVQK-HLLVNE--TSR--------------

>New|ANQ43272.1_type_I_IFN_10_Xenopus_tropicalis

MMSRTLTVPLL------------------LVLSSGG--S------HAYPLLKKGG-DHLL

NEMLMKNDRLAPEE---------------------------------I----H------E

YQVE--AAVA-----A------IQYN---------EN-FEALGLSQELHELLQLLDRLIE

EWAPYV----------------TDVNED-VSQ--------------G------ISEFYRQ

LIK-LVRKEN-------------AACVVQK-HLLVNE--TSR--------------

>New|ANQ43291.1_type_I_IFN_29_Xenopus_tropicalis

-MSRTLMVPLL------------------LVLSSGG--S------HAYPLLKKGG-DHLL

NEMLMKNDCLAPEK---------------------------------I----H------E

YQVE--AAVA-----A------IQYN---------EN-FEALGLSQELHELLQLLDRLIE

EWAPYV----------------TDLNED-VSQ--------------G------ISEFYRQ

LIK-LVRKEN-------------AACV-----------------------------

>New|ANQ43271.1_type_I_IFN_9_Xenopus_tropicalis

MMSRTLMVPLL------------------LVLSSGG--S------HGCPLLKKGG-EHLL

NEMLLTNDRLVLEE---------------------------------I----H------E

YQVE--AAVA-----A------IQYN---------EN-FEALGLSQELHEQLQLLQRLIE

EWAPCV----------------SDANKD-VSR--------------G------ISRFYRR

LRK-LVRKQN-------------AACVVRK-HLLINE--KGHD-----TTILMPR-

>New|ANQ43267.1_type_I_IFN_5_Xenopus_tropicalis

MMSRTLMVPLL------------------LVLSSGG--S------HGCPLLKKGG-EHLL

NEMLSTNDRLVLEE---------------------------------I----H------E

YQVE--AAVA-----A------IQYN---------EN-FEALGLSQELHEQLQLLQRLIE

EWAPCV----------------TDANKD-VSR--------------G------ISRFYRR

LRK-LVRKQN-------------AACVVRK-HLLINE--KGHD-----TTILMPR-

>New|ANQ43266.1_type_I_IFN_4_Xenopus_tropicalis

MMSRTLMVPLL------------------LVLSSGG--S------HGCPLLKKGG-EHLL

NEMLSTNDRLVLEE---------------------------------I----H------E

YQVE--AAVA-----A------IQYN---------EN-FEALGLSQELHEQLQLLQRLIE

EWAPCV----------------TDANKD-VSR--------------G------ISRFYRR

LRK-LVRKQN-------------AACVVRK-HLLINE--KGHD-----RTILMPR-

>New|ANQ43265.1_type_I_IFN_3_Xenopus_tropicalis

MMSHTLMVPLL------------------LVLSSGG--S------HGCPLLKKGG-EHLL

NEMLSTNDRLVLEE---------------------------------I----H------E

YQVE--AAVA-----A------IQYN---------EN-FEALGLSQELHEQLQLLQRLIE

EWAPCV----------------TDANKD-VSR--------------G------ISRFYRR

LRK-LVRKQN-------------AACVVRK-HLLINE--KGHD-----RTILMPR-

>New|ANQ43297.1_type_I_IFN_35_Xenopus_tropicalis

------------------------------------------------------------

--MLMKNDCLAPEE---------------------------------K----H------E

YQVE--AAVA-----A------IQYN---------EN-FEALGLSQELHELLQLLDRLIE

EWAPYV----------------TDVNED-VSQ--------------G------ISEFYRQ

LIN-LVRKEN-------------AACVVQK-HLLVND--TSREE----TTILT---

>New|ANQ43328.1_type_I_IFN_15_Xenopus_laevis

-MSCTFIS--L------------------MPLSNAS--S------LRCPQLQVKG-QFLL

NKMLSNFENFEPKE-------------------------------NIP----H------Y

SPTE--AVPM-----A------IKYV---------EN-CKRLGLTRELWELCQILHQLIE

EFAPCM----------------TYAYKD-ETK--------------H------ISRHYRQ

LRK-LAQKRN-------------SSCVMQK-QLLMN--------------------

>New|ANQ43326.1_type_I_IFN_13_Xenopus_laevis

-MSCTFIS--L------------------MPLSNAS--S------LRCPQLQVKG-QFLL

NKMLSNFENLEPKE-------------------------------NIP----H------Y

SPTE--AVPM-----A------IKYV---------EN-CKRLGLTRELWELCQILHQLME

EFAPCM----------------TYAYKD-ETK--------------H------ISRHYRQ

LKK-LAQKRN-------------SSCVMQK-QLLMN--------------------

>New|ANQ43319.1_type_I_IFN_7_Xenopus_laevis

-MSCTFIS--L------------------MPLSNAS--S------LRCPQLHMKG-QFLL

NKMLSNFEHLEPIE-------------------------------NIP----H------Y

SPTE--AVPM-----A------IKYL---------EN-CKRLGLTRELWELFQILHQLIE

EFAPCM----------------TYAYKD-ETK--------------H------ISRHYRQ

IRK-LAQKRN-------------SSCVMQK-QLLMN--------------------

>New|ANQ43321.1_type_I_IFN_9_Xenopus_laevis

-MSCTFIS--L------------------MPLANAS--S------LRCPQLQVKG-QFLL

NKILSNVEHLETIE-------------------------------NIP----H------Y

SPTE--AVPM-----A------IKYA---------EN-CKRLGLTRELWELCQLLQQLIE

ESAPCM----------------TYAYKD-ETK--------------H------ISRHYRQ

FKK-LAQKRK-------------IYCVKTA-SHELNI--LTDL-------------

>New|ANW82731.1_type_I_IFN_2.1_Xenopus_tropicalis

-MAPGTILYLL------------------VQPSSAS--S------PECRWPDKKG-EFID

NEILKVFDNLQPKE-------------------------------QHQ----D------D

SRGE--AAAI-----V------FAYK---------KK-G-----PRK---LLKLLLRRLK

QLGRCV----------------TGQYKA-VTD--------------A------ICKEYRE

LRQ-KAREWN-------------TTCAMTK-MYLMSK--TS---------------

>New|ANQ43282.1_type_I_IFN_20_Xenopus_tropicalis

-MAPGTILYLL------------------VQPSSAS--S------PECRWPDKKG-EFID

NEILKVFDNLQPKE-------------------------------QHQ----D------D

SRGE--AAAI-----V------FAYK---------KK-G-----PRK---LLKLLLRRLK

QLGRCV----------------TGQYKA-VTD--------------A------ICKEYRE

LRQ-KAREWN-------------TTCAMRK-MYLMSK--TS---------------

>New|AWK27013.1_IFN_2.4_Xenopus_tropicalis

-MTPGTILYLL------------------VRPSSAS--S------PECRWPD-RS-EFID

NEILKAFDNLQPKE-------------------------------QHQ----D------Y

SRGE--AAAI-----V------FAYK---------KK-V-----PRK---LLKLLLRRLK

QLGRCV----------------TGQYKA-VTD--------------A------ICKEYRE

LRQ-KAREWN-------------TTCAMTK-MYLMSK--TS---------------

>New|ANQ43275.1_type_I_IFN_13_Xenopus_tropicalis

-MTPGTILYLL------------------VRPSSAS--S------PECRWPD-RS-EFID

NEILKAFDNLQPKE-------------------------------QHQ----D------Y

SRGE--AAAI-----V------FAYK---------KK-V-----PRK---LLKLLLRRLK

QLGRCV----------------TGQYKA-VTD--------------A------ICKEYRE

LRQ-KAREWN-------------TTCAMTK-MYLMSK--TS---------------

>New|AWK27012.1_IFN_2.3_Xenopus_tropicalis

-MAPGTILYLL------------------VRPSSAS--S------PECRWPD-RS-EFID

NEILKVFDNLQPKE-------------------------------QHQ----D------Y

SRGE--AAAI-----V------FAYK---------KK-V-----PRK---LLKLLLRRLK

QLGRCV----------------TGQYKA-VTD--------------A------ICKEYRE

LRQ-KAREWN-------------TTCAMRK-MYLMSK--TS---------------

>New|ANQ43287.1_type_I_IFN_25_Xenopus_tropicalis

------------------------------------------------------------

------------------------------------------------------------

SRGE--AAAI-----V------FAYK---------KK-V-----PRK---LLKLLLRRLK

QLGRCV----------------TGQYKA-VTD--------------A------ICKEYRE

LRQ-KAREWN-------------TTCAMRK-MYLMSK--TS---------------

>New|ANQ43276.1_type_I_IFN_14_Xenopus_tropicalis

-MAPGTILYLL------------------VRPSSAS--S------PECRWPD-RS-EFID

NEILKVFDNLQPKE-------------------------------QHQ----D------Y

SRGE--AAAI-----V------FAYK---------KK-V-----PRK---LLKLLLRRLK

QLGRCV----------------TGQYKA-VTD--------------A------ICKEYRE

LRQ-KAREWN-------------TTCAMRK-MYLMSK--TS---------------

>New|ANQ43288.1_type_I_IFN_26_Xenopus_tropicalis

-MAPGTILYLL------------------VRPSSAS--S------PECRWPD-RS-EFID

NEILKAFDNLQPKE-------------------------------QHQ----D------Y

SRGE--AAAI-----V------FTYK---------KK-V-----PRK---LLKLLLRRLK

QLGRCV----------------TGQYKA-VTD--------------A------ICKEYRE

LRQ-KAREWN-------------TTCAMTK-MYLMSK--TS---------------

>New|AWK27011.1_IFN_2.2_Xenopus_tropicalis

-MAPGTILYLL------------------VQPSSAS--S------PECRWPD-RS-EFID

NEILKAFDNLQPKE-------------------------------QHQ----D------D

SRGE--AAAI-----V------FAYK---------KK-G-----PRK---LLKLLLRRLK

QLGRCV----------------TGQYKA-VTD--------------A------ICKEYRE

LRQ-KAREWN-------------TTCAMTK-MYLMSK--TS---------------

>New|ANQ43281.1_type_I_IFN_19_Xenopus_tropicalis

-MAPGTILYLL------------------VQPSSAS--S------PECRWPD-RS-EFID

NEILKAFDNLQPKE-------------------------------QHQ----D------D

SRGE--AAAI-----V------FAYK---------KK-G-----PRK---LLKLLLRRLK

QLGRCV----------------TGQYKA-VTD--------------A------ICKEYRE

LRQ-KAREWN-------------TTCAMTK-MYLMSK--TS---------------

>New|ANQ43294.1_type_I_IFN_32_Xenopus_tropicalis

-MAPGTILYLL------------------VRPLSAT--S------PECRWPD-RS-EFID

NEILKAFDNLQPKE-------------------------------QHQ----D------D

SRGE--AAAI-----V------FAYK---------KK-G-----PRK---LLKLLLRRLK

QLGRCV----------------TGQYTA-VTD--------------A------ICKEYRE

LRQ-KAREWN-------------TTCAMRK-MYLMSK--TS---------------

>New|ANQ43289.1_type_I_IFN_27_Xenopus_tropicalis

-MAPGTILYLL------------------VRPSSAS--S------PECRWPD-RS-EFID

NEILKVFDNLQPKE-------------------------------QHQ----D------D

SRGE--AAAI-----V------FAYK---------KK-G-----PRK---LLKLLLRRLK

QLGRCV----------------TGQYTA-VTD--------------A------ICKEYRE

LRQ-KAREWN-------------TTCAMRK-MYLMSK--TS---------------

>New|ANQ43293.1_type_I_IFN_31_Xenopus_tropicalis

-MAPGTILYLL------------------VRPSSAS--S------PECRWPD-RS-EFID

NEILKAFDNLQPKE-------------------------------QHQ----D------D

SRGE--AAAI-----V------FTYK---------KK-G-----PRK---LLKLLLRRLK

QLGRWV----------------TGQYTA-VTD--------------A------ICKEYRE

LRQ-KAREWN-------------TTCAMRK-M---SH--E----------------

>New|ANQ43298.1_type_I_IFN_36_Xenopus_tropicalis

-MAPGTILYLL------------------VQPSR----------------------ETID

NEILKVFDNLQLYK-------------------------------EIE----D------D

SPGE--AAAM-----V------LTYQ---------KK-TQCFRLPEEDNWLLYLLTEEID

QLAPCV----------------TDQYKA-VTE--------------P------ISREYRE

LLK-KAWEWN-------------TACTRRK-LDLIRN--IS---------------

>New|ANQ43290.1_type_I_IFN_28_Xenopus_tropicalis

------------------------------------------------------------

------------------------------------------------------------

----------------------------------------------EDNWLLYLLTEEID

QLAPCV----------------TDQYKA-VTE--------------P------ISREYRE

LLK-KAWEWN-------------TACTRRK-LDLIRN--IS---------------

>New|ANQ43268.1_type_I_IFN_6_Xenopus_tropicalis

-MAPGTILYLL------------------VRPSSAT--S------QECRWPDKKG-ENID

NEILKVFDNLQLYK-------------------------------EIE----D------D

SPGE--AAAM-----V------LTYQ---------KK-TQRFRLPEEDNWLLYLLTEEID

QLAPCV----------------TDQYKA-VTE--------------P------ISREYRE

LLK-KAWEWN-------------TACTRRK-LDLIRN--IS---------------

>New|ANQ43263.1_type_I_IFN_1_Xenopus_tropicalis

-MAPGTILYLL------------------VRPSSAT--S------QECRWPDKKG-ENID

NEILKVFDNLQLYK-------------------------------EIQ----D------D

SPGE--AAAM-----V------LTYQ---------KK-TQRFRLPEEDNWLLYLLTEEIH

QLAPCV----------------TDQYKA-VTE--------------P------ISREYRE

LLK-KAWEWN-------------TACTRRK-LDLIRN--IS---------------

>New|ANQ43296.1_type_I_IFN_34_Xenopus_tropicalis

-----TILYLL------------------VQPSR----------------------ETID

NEILKVFDNLQLYK-------------------------------EIE----D------D

SPGE--AAAM-----V------LTYQ---------KK-TQCFRLPEEDNWLLYLLTEEID

QLAPCV----------------TDQYKA-VTE--------------P------ISREYRE

LLK-KAWEWN-------------TACTRRK-LDLIRN--ISKRCLYSYNTFMA---

>New|ANQ43295.1_type_I_IFN_33_Xenopus_tropicalis

-MAPGTILYLL------------------F----------------PCRWPDKKG-ETID

NEILKVFDNLQLYK-------------------------------EIE----D------D

SPGE--AAAM-----V------LTYQ---------KK-TQCFRLPEEDNWLLYLLTEEID

QLAPCV----------------TDQYKA-VTE--------------P------ISREYRE

LLK-KAWEWN-------------TACTGEN------------------SSVIA---

>New|ANQ43332.1_type_I_IFN_20_Xenopus_laevis

-MASAAILFLV------------------VQPSRFS--S------PECQWLQKKG-EHID

NKILIPHEQCQPKV-------------------------------ENR----D------H

HELK--AAVV-----V------HTYS---------NY-GKTLGLLENDDYLRSLLEDVIN

ELAQCV----------------TNQYKD-FTD--------------P------ISNQYRE

LEK-HVQKWN-------------SACAMRK-MNLMNK--TS---------------

>New|ANQ43324.1_type_I_IFN_12_Xenopus_laevis

-MAPATIL-QQ------------------VGPSSAS--S------PECQWLDKKG-EHIE

NEILIVLDQLQPKE-------------------------------EIP----G------D

HELK--AAAV-----V------HTYR---------NF-AKTLGLPEKDEKLLSLLRYVMN

NLTPCV----------------TNQYKN-VTE--------------R------TSNQYIE

FEN-YVQKWN-------------TACAMRK-IDLMNK--TS---------------

>New|ANQ43322.1_type_I_IFN_10_Xenopus_laevis

-MAPATIL-QQ------------------VRPSSAS--S------PECQWLDKKG-EHIE

NEILIVLDQLQPKE-------------------------------EIQ----D------D

----------------------------------------VLSSLQKDEKLLSLLRYVMN

NLTPCV----------------TNQYKN-VTE--------------R------TSNQYTE

FEN-YVQKWN-------------TACAMRK-INLMNK--TS---------------

>New|ANQ43317.1_type_I_IFN_5_Xenopus_laevis

-MAPAVIL-QQ------------------VRPSNSR--S------PECQWLDKKG-EHID

NEILIVLDHLQPKE-------------------------------KIQ----D------D

HELK--AAAV-----V------HTYS---------NF-AKTLGLSKKDEKLLSLLRYVMN

NLTPCV----------------TNQYKD-VTE--------------R------TSNQYTE

LEN-YAQKWN-------------TACAMRK-MLLMSK--TS---------------

>New|ANQ43316.1_type_I_IFN_4_Xenopus_laevis

-MAPAAIL-QQ------------------VRPSSAS--S------PECQWLDKKG-EHID

NEILMVLDHLQPKE-------------------------------EIQ----D------D

HELK--AAAV-----V------HTYS---------NF-AKTLGLSENDEKLLSLLRYVMN

NLTPCV----------------TNQYKN-VTE--------------P------ISNQYTE

LEN-YAQKWN-------------TACAMRK-MHLMSK--TS---------------

>New|ANQ43313.1_type_I_IFN_1_Xenopus_laevis

-MAPAAIL-QQ------------------VRPSNSS--S------PEY------------

-----------------------------------------------------------D

HELK--AAAV-----V------HTCS---------NF-VKTLGLSENDEKLLSLLRYVMN

NLTPCV----------------TNRYKD-VTE--------------S------ISNQYTE

LEN-NAHNLN-----------------MQK-MNLISK--TS---------------

>New|ANQ43333.1_type_I_IFN_21_Xenopus_laevis

-MAPGTILYLL------------------VQPSSAG--S------RECPWFDKRG-ELVD

NEILKVFDNLQPTE-------------------------------ENK----Y------N

SQEE--AAAI-----V------FVYK---------KN-CSRL---SVNNQLLHLLQQQID

KLHTCN----------------INQYKE-ATQ--------------A------ISKEYRK

LRK-KGQPFS-------------NDLMREN-VGVSVQ--SGTIQRQRDTEYIA---

>New|ANQ43331.1_type_I_IFN_19_Xenopus_laevis

--MPA----LP------------------SAHMRRS--T------KQCAYVDDRA-ELID

HKIQKAFDHFPPNK-------------------------------NHE----D------D

SQAE--VAAM-----V------SILH---------R-----FRLPVEKNELRLLLQKQIN

QLAPCI----------------KGQYKE-ATE--------------L------IYKQYKE

ISK-KVREWS-------------TECT-----------------------------

>New|ANW82730.1_type_I_IFN_1.2_Xenopus_tropicalis

-MSQ-VLLLLP------------------VLV---C--S------PECPWLDNKG-EFQV

QKILTVLDHMEPTE-------------------------------EIP----N------D

SLGA--AAAI-----V------DKYS---------KK-CHQLGHTQKDSELLHLLHQLSQ

WVAPCL----------------TDEYKE-VTE--------------Q------VTKQF--

-RQ-TAQNPN-------------TACAMIK-QRLMN--------------------

>New|ANQ43285.1_type_I_IFN_23_Xenopus_tropicalis

-MSQ-VLLLLP------------------VLV---C--S------PECPWLDNKG-EFQV

QKILTVLDHMEPTE-------------------------------EIP----N------D

SLGA--AAAI-----V------DKYS---------KK-CHQLGHTQKDSELLHLLHQLSQ

WVAPCL----------------TDEYKE-VTE--------------Q------VTKQF--

-RQ-TAQNPN-------------TACAMIK-QRLMN--------------------

>New|AWK27008.1_IFN_1.5_Xenopus_tropicalis

-MSQ-VLLLLP------------------VLV---C--S------PECPWLDNKG-EFQV

QKILTVLDHMEPTE-------------------------------EIP----D------D

SLGA--AAAI-----V------DKYS---------KK-CHQLGHTQKDSELLHLLHQLSQ

WVAPCL----------------TDEYKE-VTE--------------Q------VTKQF--

-RQ-MARNPN-------------AACAMVK-QRLMN--------------------

>New|ANQ43279.1_type_I_IFN_17_Xenopus_tropicalis

-MSQ-VLLLLP------------------VLV---C--S------PECPWLDNKG-EFQV

QKILTVLDHMEPTE-------------------------------EIP----D------D

SLGA--AAAI-----V------DKYS---------KK-CHQLGHTQKDSELLHLLHQLSQ

WVAPCL----------------TDEYKE-VTE--------------Q------VTKQF--

-RQ-MARNPN-------------AACAMVK-QRLMN--------------------

>New|AWK27006.1_IFN_1.3_Xenopus_tropicalis

-MSQ-VLLLLP------------------VLV---C--S------PECPWLDNKG-EFQV

QKILTVLDHMEPTE-------------------------------EIP----D------D

SLGA--AAAI-----V------DKYS---------KK-CHQLGHTQKDSELLHLLHQLSQ

WVAPCL----------------TDEYKE-VTE--------------Q------VTKQF--

-RQ-MARNPN-------------AACAMVK-QHLMN--------------------

>New|ANQ43284.1_type_I_IFN_22_Xenopus_tropicalis

-MSQ-VLLLLP------------------VLV---C--S------PECPWLDNKG-EFQV

QKILTVLDHMEPTE-------------------------------EIP----D------D

SLGA--AAAI-----V------DKYS---------KK-CHQLGHTQKDSELLHLLHQLSQ

WVAPCL----------------TDEYKE-VTE--------------Q------VTKQF--

-RQ-MARNPN-------------AACAMVK-QHLMN--------------------

>New|AWK27009.1_IFN_1.6_Xenopus_tropicalis

-MSQ-VLLLLP------------------VLV---C--S------PECPWLDNKG-EFQV

QKILTVLDRMEPTE-------------------------------EIP----Y------D

SLGA--AAAI-----V------DKYS---------KK-CHQLGHTQKDSELLHLLHQLSQ

WVAPCL----------------TDKYKE-VTE--------------Q------VTKQF--

-RQ-MAQNPN-------------AACAKVK-QRLMN--------------------

>New|ANQ43278.1_type_I_IFN_16_Xenopus_tropicalis

-MSQ-VLLLLP------------------VLV---C--S------PECPWLDNKG-EFQV

QKILTVLDRMEPTE-------------------------------EIP----Y------D

SLGA--AAAI-----V------DKYS---------KK-CHQLGHTQKDSELLHLLHQLSQ

WVAPCL----------------TDKYKE-VTE--------------Q------VTKQF--

-RQ-MAQNPN-------------AACAKVK-QRLMN--------------------

>New|AWK27005.1_IFN_1.1_Xenopus_tropicalis

-MSQ-VLLLLP------------------VLVSRGS--S------PECPWLDNKG-EFQV

QKILTVLGSMEPME-------------------------------EIP----Y------D

SLGA--AAAI-----V------DKYS---------KK-CHQLGHTQKDSELLHLLHQLSQ

WVAPCL----------------TDEYKE-VTE--------------Q------VTKQF--

-RQ-MARNPN-------------AACAMIK-QRLMN--------------------

>New|ANQ43286.1_type_I_IFN_24_Xenopus_tropicalis

-MSQ-VLLLLP------------------VLVSRGS--S------PECPWLDNKG-EFQV

QKILTVLGSMEPME-------------------------------EIP----Y------D

SLGA--AAAI-----V------DKYS---------KK-CHQLGHTQKDSELLHLLHQLSQ

WVAPCL----------------TDEYKE-VTE--------------Q------VTKQF--

-RQ-MARNPN-------------AACAMIK-QRLMN--------------------

>New|ANQ43280.1_type_I_IFN_18_Xenopus_tropicalis

-----LLLTLP------------------T----------------TCP-----------

------------------------------------------------------------

-LGA--AAAI-----V------DKYS---------KK-CHQLGHTQKDSELLHLLHQLSQ

WVAPYL----------------TDKYKE-VTE--------------Q------VTKQF--

-RQ-MAQNPN-------------TACAMVK-QRLMN--------------------

>New|AWK27010.1_IFN_1.7_Xenopus_tropicalis

-MSQTVLP------------------------SRGS--S------PECPWLDNKG-EFLV

HKILTVLDRMEPME-------------------------------EIP----D------D

SLGA--AAAI-----V------DKYS---------KK-CHQLGHTQKDSELLHLLHQLRQ

WVAPCL----------------TDEYKE-VTE--------------P------ILEQLRQ

QRQ-TAPNPN-------------TACAMFK-QHLMN--------------------

>New|ANQ43277.1_type_I_IFN_15_Xenopus_tropicalis

-MSQTVLP------------------------SRGS--S------PECPWLDNKG-EFLV

HKILTVLDRMEPME-------------------------------EIP----D------D

SLGA--AAAI-----V------DKYS---------KK-CHQLGHTQKDSELLHLLHQLRQ

WVAPCL----------------TDEYKE-VTE--------------P------ILEQLRQ

QRQ-TAPNPN-------------TACAMFK-QHLMN--------------------

>New|AWK27007.1_IFN_1.4_Xenopus_tropicalis

-MSQTVLP------------------------SRGS--S------PECPWLDNKG-EFLV

HKILTVLDCMEPTE-------------------------------EIP----D------D

SLGA--AAAI-----V------DKYS---------KK-CHQLGHTQKDSELLHLLHQLRQ

WVAPCL----------------TDEYKE-VTE--------------P------ILEQLRQ

QRQ-TAPNPN-------------TACAMVK-QHLMN--------------------

>New|ANQ43283.1_type_I_IFN_21_Xenopus_tropicalis

-MSQTVLP------------------------SRGS--S------PECPWLDNKG-EFLV

HKILTVLDCMEPTE-------------------------------EIP----D------D

SLGA--AAAI-----V------DKYS---------KK-CHQLGHTQKDSELLHLLHQLRQ

WVAPCL----------------TDEYKE-VTE--------------P------ILEQLRQ

QRQ-TAPNPN-------------TACAMVK-QHLMN--------------------

>New|ANQ43274.1_type_I_IFN_12_Xenopus_tropicalis

-MAQ-SLLIVS------------------VLPSSGT--F------PECLKLG-----FMV

QEVQTIFGRMEPME-------------------------------EIP----H------D

SLGA--AAAV-----M------DKYR---------KK-CRQLGGTRKDSELLHLLHQLRH

RVAPCL----------------TDEYKE-VME--------------L------ILEHFKQ

QRW-MARNRN-------------AACAMIK-QRLMN--------------------

>New|ANQ43330.1_type_I_IFN_17_Xenopus_laevis

-MSG-IVLGLL------------------LQGPITT--S------PQCPWPAQNG-EFLV

NQMLSIFDHLKPEK-------------------------------EFM----H------K

SQME--ALPV-----A------TRYT---------EN-CQILGHLQEQNKLYVLLHQLGE

MLAQYK----------------TNDYKE-AME--------------A------ISKKFRK

LRR-RAQKRH-------------TAHAIGT-QSLMNG--TF---------------

>IFN1_coela

--------MTL------KM--LLAFC---LLL----LVSNGT-FCQDCKKWVKQ-----Y

NKGLQYLEAMGGEFPLKCLAQ-----------------------LDVP----RKVVLRHS

KGER--RIEL-----V-H----DTLEHISKTYS-NN--TSTKWDEENLKKFQNVIHLESE

ELRACL--------------QERVSNKN-TQWRK----------KMT------LSRHFKE

TGN-FLQRQNYSSCAWETVRAITRMILQLI-Q------------------------

>IFN2_coela

--------MAL------KT--FVALC---LLL----FVPIGI-FCQECEELNSQQ-RLRI

RESLQELEGVGGKFPSQCLAQ-----------------------FNLH----KKVLLKHS

KGER--RITL-----V-Y----EILQQINRIYR-KN--PSATWDQNKLERFQNVLHSQTE

ELWKCL--------------EKKMSNMN-SQWNN----------AMK------LSKNFKE

MEK-FLKHQNYSSCAWELVRTITRRVLQQV-ER---------K-------A-----

>IFN6_cami

--------MAV------HY--QCGLS----LF----AMLCVS-LTLGCSTLRLQK--ILI

ATTLNTLDEMGGHVPRHCVAVGAELRIA--S-------------PDLR----L--LLQPL

QNND--RILL-----L-H----KTFQHLNKIFH-KNM-KSVTWDLTQVNHFRELLVTQRD

VVKDCI--------------QDSAS--D-SMLSA----------LST------IHTYFRK

LKK-FLKQQRYSACAWEVIRMETRARLQQI-LILTAR--MTKG-------N-----

>IFN1_cami

--------MAV------HY--QCGLS----LF----AMLCVS-LTLGCSTLRLQK--ILI

ATTLNTLDEMGGHVPRHCVAVGAEQGIA--S-------------PDLR----L--LLQPL

QNND--RILL-----L-H----KTFQHLNKIFH-KNM-KSVTWDLTQVNHFRELLVTQRE

VVKDCI--------------QDSAS--D-SMLSA----------LST------IHTYFRK

LKK-FLKQQRYSACAWEVIRMETRARLQQI-LILTAR--MTKG-------N-----

>IFN1_sqac

--------MVF------PS--VWRLW---ILL----VLLPGT-LSQDCQRLQLLD--NIN

NQALDALREMGGPIPLHCKTERTSLRAK--S-------------LDLH----Q--LSKRL

QTPD--RIQI-----V-H----QTLRHLTKIYS-MNL-GSVTWPRDKVENFRLLLDRQLG

ELEECV--------------RKPVP--E-SRPRR----------NAP------IHKYFRK

VEK-FLKQKRFSDCAWEIIRAETRARLQQI-LFITAK--IRRR-------S-----

>IFN2_sqac

--------MVF------PS--VWRLW---ILL----VLLPGT-LSQDCQRLQLLD--NIN

NQALDALREMGGPIPLQCKTERTSLRTK--S-------------LDLH----Q--LSKRL

QTPD--RIQI-----V-H----QTLRHLTKIYS-MNL-GSATWPRDKVENFRLLLDRQLG

ELEECV--------------RKPVP--E-SRPRR----------NAS------IHKYFRK

VEK-FLKQKRFSDCAWEIIRAETRARLQQI-LFITAK--IRRR-------S-----

>Leer_25

------------------------------------------------------------

------------------------------------------------------------

------TLLV-----V-D----QMLRQFRKIYS-MNL-ASVTWLQDKVENFRLLLDRQIR

ELENCV--------------RNTGS--E-TRPRR----------SAA------VHNYFRK

LGK-FLKRK-----------------------------------------------

>Leer_37

------------------------------------------------------------

------------------------------------------------------------

-TQE--RIHT-----A-H----QTLQQINNVYS-MNL-DSITWAQHKVENLRLLLDRQLR

TLEECV--------------KKPGS--K-STSKR----------NTR------ISNYFRK

LRK-FLKRNRFSDCAWEITRTE----------------------------------

>Scca_1

-------------------------------------VISGE-IVWGFNEILFYS--S--

---------QGGSFPRHCIKHRHALKTK--P-------------LNLV----K--LSKGL

EKED--QIQI-----L-H----QTLRHISKIYS-MNL-GSVTWDRDTVENLRLLLDRQLS

ELE---------------------------------------------------------

--------------------------------------------------------

>Leer_3

------------------------------------------------------------

------------------------------------------------------------

-DMD--TAVV-----V-Y----QVVSQFKEIYH-MDR-TSVTWPQDVMKRFGIKLDAQSS

ILENCV--------------RNAGS--D-AQTQK----------KAT------IQEYFKK

LSE-FLNRERFSACAWE---------------------------------------

>Leer_4

------------------------------------------------------------

------------------------------------------------------------

-APD--LAGI-----V-Y----QVVTQYNRIYN-MDR-TSVTWPQIIMTSLGFHLDVQTT

VLEDCL--------------THRGA--M-AQRQN----------RET------IHDYFRG

LSE-FLNRERFSACAWEAIREEMILWYQQV-FKFF---------------------

>IFN2_cami

--------MPL------RC--VWKLC---LCL----ALLATQ-TPSLACNLPLHN--LMC

QRSLNLLLWMRDSIPFHCVREMGST---------VDLR------LNLR----N--VTGPL

QPGD--RMQV-----Y-L----QTLHHLNEIYS-NNV-TS-TWDQEKILGFRFVLDEQQM

EMEKCA--------------KEPAS--D-AMVHT----------TSA------IRTYFTK

LGR-FLRQKRFSACAWEVIRAKTSRRLQEM-LTLAMK--EAKT-------R-----

>IFN3_coela

--------MAL------KC--LWTMF---LLL----LDFPVA-FPEQCNWVHLHQ-KY-S

TSKLPLLDEMGAGFSESCMIE-----------------------IVKD----KKLTLKFP

NSDH--MIETDIMPTV-C----EILNFIGNIYN-KNL-QLVPWDKKKIEHFQTVLYHEVE

EIKKCL--------------PGEKTNAN-SHSNS----------NMK------LQDYFST

LEN-FLEQKEYSPCAWEVVRAHIRTLLQFT-DRLTTV--ITKN-------E-----

>IFN4_coela

--------MAL------KC--LWTMF---LLL----LDFPVA-FPEQCNWVHLHQ-KY-S

ISKLQLLDEMGAGFPPHCINE-----------------------KGVD----EKVTLKFP

KQDH--MIQMEIMPTV-C----EVLNFTGNIYN-KNL-QFVPWNKRKVQHFQTVLHHEVE

ELKKCL--------------PEEKTNAN-SHSNS----------NMK------LQDYFSK

LEN-FLEQKEYSLCAWEIVRVHIRKLLQLT-DRLTTA--IRKT-------E-----

>anja_f

----------------------------------------G--KRLP---KDCQQEAMRI

NISHDERQKL-----LDLKEE----------N--IS------------------------

-------------------TLQQVFNLTWRIFR-KNM-TAVNWNMSKLKSFRKLLKRQSR

TFSTCA--------RL---T-------SSSRPAK----------NDG------KRVYFRK

LSR-FLSTEKYSRCAWEIVRAEI---LTLLHVPRIAH--NSGH-------KENAP-

>IFN5_coela

--------MAQ------KF--QIISL---ILF----IVSQVR-ADDQCTWSTAQQ-MHLN

KRNLNLTDDMGKFSPAECTTE-----------------------IQEV----QKMRLRFP

ENE-------DVIFIV-Y----TTLRHISKIYS-KNL-QPVSWNKTVLHEFQAAVHSQVE

ELEKCL--------------MEKMVDHY-LERKV----------ELK------LRNYFKL

LEK-MLAEKENNQCAWRFIRAQVRKFLYRI-DQLTAW--IGKM-------KNQSS-

>IFNB4_ONMY

--------MAVLK---------W-LSICLTLF----CQGTA--VSKPCRWTQFRL-GKLN

DVSIDLLSDMGGIFPLMCAEE----------N--VE----QMFPEDLY----K------N

TEGE--DVSV-----V-A---LEAMRYVDQLYN-NSL-TSVTWNKIKLNMFQNVIYRQVQ

NLELCV--------VGGVWE-------S-S----------------GDGGSVTLKTYFNK

LNT-VLKEKEHSACAWEIVRKEI---RENLKKFI-------DS-------RVKP--

>IFNB1_SASA

--------MAVLK---------W-LSICLTLF----CQGTV--ASKPCRWTQFRL-GKLN

DVSIGLLSDMGGLFPLMCAEE----------N--VE----QMFPEDLY----K------N

TEGE--DVSV-----V-A---LEAMRYVEQLYN-NSL-TSATWSKTKLNMFQNVIYRQVQ

NLELCV--------VGGVWE-------S-S----------------GDGWSVTLKTYFNK

LNT-VLKEKEYSACAWEIVRKEI---RENLKKFI-------DS-------RVKL--

>Eslu_6

--------MS--------------------------------------------------

-------------------ED---------------------------------------

------VAVV-----A-L----KTFGYVEQLF-DSKL-TPSTWNNGTFHLFKNCIFRQIQ

GLQEC------------------------------------------------V--NLRP

I-------------------------------------------------------

>IFNC1_ONMY

--------MILQTV--------W-MS---AFL----CLAQV--CSVP---MPCQLQGQLV

RITHNLLRDMGGNFPLECLQE----------N--VF----VAFPATAF----T------I

SGES--QLSS-----SGAMAIYETLKNIDTLFG-ADD-LPTKWDQQKLENFQNIVYRQIE

ESK-CM--------MGSVDT-------S-DYLIR----------TEG------LKTYFGN

IAA-VLKEKNFSYCAWEVVRKEL---LYSL-QFILEH--NSDS-------LLRT--

>IFNC1_SASA

--------MALQTI--------W-MS---AFL----CVAHV--CSMP---MPCQLQGQLV

RITHNLLRDMGGNFPLECLQE----------N--VF----MAFPATAF----A------S

SGAP--QLGS-----SGAKAIYETLKNIDILFE-ADD-LPTQWDQQKLKNFQNIVYRQIE

ESK-CM--------MGSVDT-------S-DYLIR----------TEG------LNTYFGN

IAA-VLKEKNFSYCAWEVVRKEL---LYTL-QFILEH--NSDS-------LLRT--

>Eslu_1

--------MALTTI--------W-VS---ALL----CFVHV--WSMP---MTCHIQETLM

ESAKDLLRDMGGHFPLECLQG----------K--VN----ITFPAPAF----A------T

SSTP--TLSG-----SGAKAIYETLKNIELLFG-AED-LPTKWDQQKLDNFQNIIYTQID

HSK-CV--------SGSVET-------S-DYPVR----------AAA------LKTYFGN

IEA-ALKEKKFSYCAWEVVRKQV---LETL-IFILTK--NSDC-------LLRT--

>clha_a13

--------MVLQSF--------W-VT---IIL----CFVHV--STVP---ITCGLQRRLV

EKSHSLLESMSGLFPVECLEH----------N--LP----IAFPSSAF----M------T

SEAA--E-SA-----GAEKVAYETLKLIDTLFA-NDS-MPTSWNN--LEDFQEIIYRQIE

ESE-CI--------M-TQSK-------D-DFPTR----------NAA------LKTYFDK

IAT-ILKEKESSDCAWEVVRKEI---LYTL-KFIL-Q--SSNY-------LI----

>Anja_11

--------MAF-----------WFIF---AFL------AQA--WSMP---QTCQLDGNLI

RTSHNLLKDAGGHFPLQCIKE----------N--VL----IMFPSSAF----E------S

NGTI--EQET-----GVRMAIYETLRSLSLVLE-DGD-LPTKWDEKIMDDFQNIVYRQVD

----------------S-------------------------------------------

-------KCSFSFCAWEIVRKEL---VRTL-HFILDH--RSDM-------LCRL--

>Anja_10

------------------------------------------------------------

------------------------------------------------------------

-------QSD-----IIRTVIYETLYSINSLFE-NDD-FPTDWDEIKLQDFQNIIYRQVD

KST-CV--------SS-------------------------------------LSPEM--

--------------------------------------------------------

>IFN3_DARE

--------MDLHRV--------W-LC---TFF----CFVQV--WSLP---TNCNLQKNLM

KRTYTLLET-AGLFPMQCLDD----------R--VS----IPFPQNVF----E------Y

NE-D--QVTG-----V-EKAVYQTLQNIDALFE-NFG-DPDQWDAEKLDDFRGIVYRQIK

YST-CI--------MNKTEV-------A-DFPSR----------EAS------LKVYFET

ISS-TLKEKNSSYCAWEIVRSEI---LRTL-EFILKN--NSDI-------ML----

>Leoc_1

---------------------------------------------MP---TRCAFREHLI

EVSLNLLKDMGGHFPRECIKD----------N--VL----ITFPANVL----F------S

SFAQ--TQNE-----NIQPVVYETLRSVNTLFQ-SEG-RPSTWDQRKLEDFQSVVFRQVS

DFKKCA--------LRRRET------------------------SSANNSTVQLKTYFKK

MGR-FLEEKNYSSC------------------------------------------

>Leoc_2

--------MALEGS--------W-LC---VVF----CLSQA--WAMP---TRCAFREHLI

EVSLNLLKDMGDGFPKECLSD----------N--VV----IAFPAEAY------------

EFAE--TQKE-----DFEIAIYKTLNSTDALFE-NEG-RPTSWGQRAVDEFQNLVFRQVQ

DFNTCV--------PGDVET-------TGN--------------SSAAYRITLLKTYFQK

MEN-VLQEKNYSSCAWEIIRKEL---LGIL-QVILDK--NAEI-------VV----

>IFN2_DARE

--------MEFWQV--------F-LC---PAL----FFAHI--TSKP---TNCFMRRKHV

KTAYSLLESMGGLFPRECLKE----------N--VR----ITFPKYAL----Q------S

NNSN--QKTG-----V-AKAVYKIMDHIDFLFA-NDS-YPEAWNKRKVDNFQNIVYRLTK

ENQ-CI--------MRMRAT-------V-DFPAR----------DDA------LKSYFNK

LAT-LLRNKDNSFCAWEVVRHEL---LGVL----------SDI-------IL----

>Scma_1

--------MTP-------------LSVLLLQL----CSFQV--VAMP----TCKLQANVV

LKTHNLLRDLGAAFPVHCLQY----------N--VN----ISFPDSAF----P------D

ATAH--PQCR-----RALWVVYESLRGMQLILE-QND-SPVTWDEGILDNFQNLQHRLLE

DGS-CL--------ST---K-------GPDV----------------------FSSYFSN

VTD-VLQQQDSSVCGWMALRRDV---LSVLKTALREH--NSCF-------T-----

>IFNA_human

MALTFALLVAL------------------LVLSCKSSCS------VGCDLPQTHL-GS--

RRTLMLLAQMRRISLFSCLKDRHDFGPQEEF--------------G-N----Q------F

QKAE--TIPV-----L-H----EMIQQIFNLFS-TKD-SSAAWDETLLDKFYTELYQQLN

DLEACVIQGVGVTE--------TPLMKEDSIL--------------A------VRKYFQR

ITL-YLKEKKYSPCAWEVVRAEIMRSFSLS-TNLQES--LRSKE------------

>Loaf_11

MAFSFLLLIAL------------------VVLSCNSTCS------LGCDLPQSHL-AN--

RRTMMLLGQMRRISPFSCLKDRNDFGPQEEL-------------DG-N----K------F

QKAQ--AISV-----H-H----EMIQQTFNLFS-LQA-SSAAWDKTLLDKLYTGLYQQLN

DLEVCLMQEMGVEE--------APVINEDSML--------------A------VRKYFQR

ITV-YLTEKKYSPCAWETVRAEVMSSFSAS-TNWKER--LRSKEGDLAP-------

>IFNA_horse

MALPVSLLMAL------------------VVLSCHSICS------LGCDLPHTHL-GN--

TRVLMLLGQMRRISPFSCLKDRNDFGPQEVF-------------DG-N----Q------F

RKPQ--AISA-----V-H----ETIQQIFHLFS-TDG-SSAAWDESLLDKLYTGLYQQLT

ELEACLSQEVGVEE--------TPLMNEDSLL--------------A------VRRYFQR

IAL-YLQEKKYSPCAWEIVRAEIMRSFSSS-TNLPQS-------------------

>IFNA_pig

MAPTSAFLTAL------------------VLLSCNAICS------LGCDLPQTHL-AH--

TRALRLLAQMRRISPFSCLDHRRDFGPHEAF-------------GG-N----Q------V

QKAQ--AMAL-----V-H----EMLQQTFQLFS-TEG-SAAAWNESLLHQFCTGLDQQLR

DLEACVMQEAGLEG--------TPLLEEDSIL--------------A------VRKYFHR

LTL-YLQEKSYSPCAWEIVRAEVMRSFSSS-RNLQDR--LRKKE------------

>Oror_10

MAPTVSLLLAL------------------VLLSCHSNCS------LGCDLPQTHL-AN--

TRALMLLQQMRRISPFSCLKDRNDFGPQEAF-------------GG-N----Q------F

QKAQ--AIAV-----V-H----EMIQQTFQLFS-TEG-SAAAWDETLLDKFCTALYQQLT

DLQACLMQEAGLEG--------TPLLKEDSIL--------------A------VRKYFHR

ITV-YLQEKKYSPCAWEIVRAEVMRSFSSS-TNL----------------------

>Ptva_8

MALLFSFLMAM------------------VVLSCQSICS------LGCDLPQTHL-VN--

RRALMLLGQMRRISPFSCLKDREDFGLQGAF-------------GG-N----Q------F

QEAQ--AIAV-----F-H----EMTQQTFLLFC-TEV-LSAAWDETLLGRFCNGLYQQLD

HLEACQTQELGAEE--------TPLLDEDSTL--------------A------VRKYFQR

INL-YLQEKKHSPCAWEIVRAEIMRSYSLS-THLKEK--SRSKD------------

>Ereu_6

MAPSSLFLKAL------------------LVLSCSYIFG------LGCDLPQSHP-VN--

RRPLLLLGQMRRLPPFSCLKDRHDFAPQEVF-------------DG-Q----Q------F

QKAH--ALSV-----L-H----EMLQQIFHLFS-TKH-SSADWDEGLLNSFCAELHQQLN

VLEGCQTQEVRVEQ--------TPRMK-DSIL--------------A------MKRYFQR

ITM-YLREKKYSPCAWEIVRVEIIRAFSLS-TKLQEK--LRSKD------------

>IFNA_mouse

MARLCAFLVML------------------IVMSYWSTCS------LGCDLPHTYL-RN--

KRALKVLAQMRRLPFLSCLKDRQDFGPLEKV-------------DN-Q----Q------I

QKAQ--AIPV-----L-R----DLTQQTLNLFT-SKA-SSAAWNTTLLDSFCNDLHQQLN

DLQTCLMQQVGVQE--------PPLTQEDALL--------------A------VRKYFHR

ITV-YLREKKHSPCAWEVVRAEVWRALSSS-VNLLPR--LSEKE------------

>Oror_9

MAFVLPLLTAL------------------VVFSYGPGGS------LGCDLSQNHR-IS--

RKNFMLLGQMRRISPRFCLKDRKDFGPQDMV-------------DG-S----Q------L

PKAQ--ATSV-----L-H----EMLQQVFCLFH-TER-STATWDTSLLDKLRTGLHQQLE

DLDACLVQAMGDEE--------TALGVTGPTL--------------A------VKRYFQG

IHL-YLKEKKYSDCAWEIVRVEIMRSLSSS-TNLQER--LRIMNGDLGSP------

>Loaf_4

MALLLSLLTAL------------------VVFSCGPAPS------LGCDLPQNHV-AS--

EKTVDLLDQMQRCPTFFCLDDRKDFRPQEMV-------------DG-S----Q------L

QKAQ--AIAF-----L-H----EMLQQIFDLFR-TMD-SFAAWNTTLLNQLLNGLPEQQE

DLETCFMQAMEEGK--------SALPIEGPAL--------------A------VKEYFEG

IRF-YLKEKEYSDCAWEFVRVEIRRSFSSS-TALQER--LRRKDGDMSSS------

>Loaf_5

MAFLLFLLTAL------------------VVFGCGPAPS------LGCDLSKKHL-TS--

KKTFVVLDQMRRLSPFSCLKERKDFRPQEMV-------------DG-S----Q------L

QKAQ--VISV-----L-H----EMLQQIFNLFH-TKD-SSAAWNTTLLDQLHSGLYLQLE

DLEACLVQAMEEEE--------SVLAIESSAL--------------A------VKRYFQG

IHS-YLKEKEYSDCAWEIVRVEIKRSFSSS-TNLQER--LRRKHGDMGSS------

>Dano_12

MALQLSLLMAL------------------VVFSCGPVPS------LSCDLPQSQL-VD--

RKTFVLLGQMGRISPFSCLKDRADFRPQEMV-------------DG-S----Q------V

QKSQ--AKFV-----L-H----EMFQQIFNLYH-TEG-SSAAWNMTLLDQLLSTLHEQLE

DLEACLLQEMGEEE--------TLLGIEGPVL--------------A------MRRYFQG

IRL-YLQEKKHSDCAWEVVRMELRRAFSSS-PNLKER--L----------------

>IFNO_human

MALLFPLLAAL------------------VMTSYSPVGS------LGCDLPQNHL-LS--

RNTLVLLHQMRRISPFLCLKDRRDFRPQEMV-------------KG-S----Q------L

QKAH--VMSV-----L-H----EMLQQIFSLFH-TER-SSAAWNMTLLDQLHTGLHQQLQ

HLETCLLQVVGEGE--------SAGAISSPAL--------------T------LRRYFQG

IRV-YLKEKKYSDCAWEVVRMEIMKSLFLS-TNMQER--LRSKDRDLGSS------

>IFNT_cow

MAFVLSLLMAL------------------VLVSYGPGRS------LGCYLSEDHL-GA--

RENLRLLARMNRLSPHPCLQDRKDFGPQEMV-------------EG-S----Q------L

QKDQ--AISV-----L-H----EMLQQCFNLFH-IEH-SSAAWNTTLLEQLCTGLQQQLE

DLDACLGPVMGEKD--------SDMGRMGPIL--------------T------VKRYFQD

IHV-YLKEKEYSDCAWEIIRVEMMRALSSS-TTLQKR--LRKMGGDLNSL------

>Ptva_4

MAPLLSLITAM------------------LVFSYGPSGS------LSCDLSQNHQ-VN--

KESIVLLHQMQRISSFRCRKDRKNFGPQEMV-------------DG-S----Q------V

QEAQ--AISV-----L-H----EMLQETSNVFG-SEH-SSAAWNTTVLHGLLSRLHWQLE

DLGTCLVLQMKEAE--------SALGMEAPTL--------------A------VKRYFQG

IRL-YLKEKQYSDCAWEIVRVEIKRAFSLS-TNLREM--LRNQDGDLRSP------

>Dano_2

MAFPVSSLVVL------------------MMIFSSPIGS------FSCGLPQSLV-RK--

QETFTVLSQMGTISLLSCLKDRTDFRPQEMM-------------DG-S----Q------V

QKTQ--AMSV-----L-H----EMLQQIFHLFH-TEG-SSAAWNTTLLDQLRSGLHRQLE

DLETCLLQEMG-ED--------SVLAMEGPTL--------------A------VRRYFQR

IRV-YLQKKKHSDCAWEVVRVEIRRCFLFI-NVLTRE--LRK--------------

>Ptva_16

MAFLVSSLMAL------------------VVIFSSPISS------MICDLPQSLL-GK--

QETSTALNQMQRISSFLCQKDRKDFRPRKMV-------------DG-S----Q------V

QKAQ--AISV-----L-H----EMLQQTFDVFG-TKQ-SSAAWNTTLLHGLLSGLHRQLE

DLGTCLVPEMKEVE--------SVLGTEDPTL--------------A------MKRYFQG

IHL-YLEEKQYSDCAWEVVRVEIRRYLFVV-NKFTRK--EI---------------

>Dano_10

MAPPVSVLKTL------------------LMLCSIPAC-------LGCDLPLIY--GH--

QEPFMLLHQMGRLSILSCLKDRTDFQPQELM-------------DG-I----Q------L

DKMH--ATTL-----L-H----EVVQQIFNLFS-TSG-SLATWDDTLLDRFLIGLHQQLD

NLETCLGKEKEEDQ--------THLGSENSRL--------------A------VKRYFQG

ISQ-YLTEKQDSPCAWEVVRVEIRKCFLFI-NKLQGK--LRK--------------

>Loaf_1

MALPISVLMAL------------------VMFCCRPACF------WCCDLPLSH---N--

QETFTLLNQMERISLLSCLKDRTDFRPQILM-------------DM-N----Q------L

EKTQ--AAVL-----L-Y----EMLQQTFNLFS-RSD-SLEAWDETFLDKFLLGLYQQLN

DLEICFEKERKVEQ--------IPLGTEN-----------------S------VKSYFQG

IGL-YLKEKEHSLCVGGCQSGN-QKMLSLH--------------------------

>Modo_6

MTSWSLLPVAL------------------ALLCSSTLCS------LDCDLTLGL------

QEDFSLLNQMSTSSLVPCLKDGINFNPKEAM-------------DR-S----Q------L

QKEN--ATVI-----V-L----EMVQQIFTLFS-QNT-TPATWNQTQVIQLLIRLDQQLE

QLERCLGQNVKWEE--------FSLRSEKTRF--------------A------LKSYFQG

ISQ-YLQGKEYSPCAWEIVRVEIRRLFLFM-SKLARK--LRD--------------

>Oran_7

MTNAGLIQIVL------------------VLLVSTSTVS------LSCSLLHT----VCM

EQSLKRLDRMQGKSLLSCLKDRKDFQPQELV-------------EA-G----P------F

KEGN--RAVA-----V-H----ELLQQIFTIFS-QNL-SQTGWDQSEVENFLHGLHRQLE

ELEVCQ--GTDTRW--------ASVGSDILRL--------------R------LKSYFRS

ISL-YLRDKDYSSCAWEIVRAQIRRC---I-FQFMRR--LRN--------------

>Oran_1

MTNRSSLPFVL------------------WLLLPTTIMA------QGYPKLYSHQ-WLSN

WQSLHLLDEMGGQFPLHCLKEKTNFKPAEMM-------------HP-H----Q------F

QQEN--ATEA-----I-H----DLLQNIFNIFG-RNH-SQTGWDEATVEKFLHGVHKEMM

RLELFE--EMGWEN--------STLRGDV-SL--------------H------IKSYFKG

MMD-YLKGRDYSSCAWEVTRMEAKRCFLVM-YRLTRK--LKK--------------

>IFNE_human

MIIKHFFGTVL------------------VLLASTTIFS------LDLKLIIFQQ-RQVN

QESLKLLNKLQTLSIQQCLPHRKNFLPQKSL-------------SP-Q----Q------Y

QKGH--TLAI-----L-H----EMLQQIFSLFR-ANI-SLDGWEENHTEKFLIQLHQQLE

YLEALL--EAEKLS--------GTLGSDNLRL--------------Q------VKMYFRR

IHD-YLENQDYSTCAWAIVQVEISRCLFFV-FSLTEK--LSK--------------

>IFNE_cow

MINKAFFEIVL------------------VLLASSTVCS------QELKLVLCQQ-RRVN

QESLKLLNKLQTSSVQQCLPHRKHFLPQKSV-------------NP-H----Q------Y

QKGQ--VLAI-----L-H----EMLQQIFSLFR-AIV-SLDGWEESHTEKFLVELHQQLE

YLEALL--QAKQKS--------DTLGSENLRL--------------Q------VKMYFQR

IHD-YLESQDYSSCAWTIVQVEINRCLFLV-FRLTRK--LSE--------------

>Oror_7

MINKPFFDIVL------------------VLLASSSVCS------RELKLVLFQQ-KRVN

RESLKLLNKLQTSSIQQCLPHRKNFLPQKSM-------------NP-H----Q------Y

QKGQ--ALTI-----L-H----EMLQQIFNLFR-AII-SLNGWEESHMEKLLIELHQQLK

YLEALR--QAEQKR--------DTLGSENLRL--------------Q------VKIYFQR

IRD-YLENQDYSTCAWTIVQVEINRCLFFV-FQLTGK--LSKQ---------ET--

>Ptva_15

MISKYFFEVVL------------------VLLASSTVFS------LELKLVLFQQ-RRVN

RENLKLLNKLQTPSIHQCLPHRKNFLPQKSL-------------NP-H----L------Y

QKGC--ALAI-----L-H----ETLQQIFSLFG-ANI-SLDGWEESHMEKFLIELHQQLE

YLETLQ--QAEQKS--------GILGSENLRL--------------Q------VKMYFQR

IRD-YLETQEYSRCAWTIVQVEINRCLFFV-FQLTGK--LSKQ---------DP--

>Dano_16

MINKHFFEIVL------------------VLLASSTLFS------LELKLVLFQQ-RQVN

RESLKLLNKLHT-SIQQCLPHRKNFLPQESM-------------NP-Y----Q------Y

QKGH--AVAI-----L-H----EMLQQIFNLFR-EKL-SLAIWEESQVEKFLIELHQQLE

HLEALQ--EPELKS--------DTLDSETFRL--------------Q------VKTYFRR

IRD-YLENQEYSSCAWTIVHVEINRCLFLF-TDSQES-------------------

>Loaf_2

MINKYFFETVV------------------VLLSSSMIFS------LELKLVHFQQ-R-MN

RESLKLLNTLWSSSIQQCLPHRANFAPQKSM-------------NP-H----Q------Y

HKGH--AVAI-----L-H----EMLQQIFNLFR-TNL-VLGSWEERHMEKFLIELYGQLE

HLEALL--EAEQKS--------GSLGTENLRL--------------Q------VKMYFQR

IHN-YLENQKYSSCAWTIVRVEIIRCLFFV-FRLTGK--LSQY---------DP--

>Ereu_10

MISKHIF--VL------------------ILLASSPIFS------LELKLFLVQL-RRLN

RESLKLLNIRQTSSIQRCLPHRKNFLPLKSP-------------SP-H----W------Y

QTEH--ALAI-----L-H----EMLQQIFNLFR-VNI-SLDDCEESYMEKFLMELHQQLE

LLEAFL--EAEQNS--------NTLSSENLRM--------------Q------VKMYFQR

IHN-YLGKQGYSNCAWTIVRVEINRCLLFM-LRLTTK--LSKQ---------DF--

>IFNE_mouse

MVHRQLPETVL------------------LLLVSSTIFS------LEPKRIPFQL-W-MN

RESLQLLKPLPSSSVQQCLAHRKNFLPQQPV-------------SP-H----Q------Y

QEGQ--VLAV-----V-H----EILQQIFTLLQ-THG-TMGIWEENHIEKVLAALHRQLE

YVESLL--NAAQKS--------GGSSAQNLRL--------------Q------IKAYFRR

IHD-YLENQRYSSCAWIIVQTEIHRCMFFV-FRFTTW--LSR--------------

>Dano_1

MDHM-YLLLAG------------------LMLCSSLDCS------LGCPLPRSQL-ES--

KEIFTLLRQMNRIPSHSCLNDRVDFKPWKAE-------------TV-T----Q------I

PKTQ--ATCF-----S-Y----EMFQQIFNLFQ-KEN-SRAAWDNSLLDELLSRLDHNLE

QV-----EQMKVE----------NLPCADLGT--------------L------VRDYLQG

TDG-YLNEKKYSSCAWEVVRGEPEMCFPLI--------------------------

>Oror_2

MAQI-YLLVAG------------------VLLCSIPAYS------LGWNLPRSHQ-EN--

KDVFQHLEQLQRIPSQWCLKDRTDFKPWKRE-------------NI-T----P------I

QVTQ--GTCH-----H-H----LMLQQIFNLFT-TED-SRAAWNNTLLDKLLSSLHLRLH

RL-----EQMKKD----------NLDCRDLGR--------------A------AREYFHG

IHV-YLKAKEYSPCAWEVVRVEIKRCLSLM--------------------------

>Ptva_17

MAQCSSWLAAG------------------EMLSFILICS------LGGDVPWIHL-EN--

RKIVSLLRELEVIPSHFCLKDRTDFKPWERG-------------SI-T----E------I

QKTQ--RTCF-----H-H----LILQQIFSLLN-AED-SHAAWNRTLLYQLLSRLHHSLE

EL-----DQTNEG----------NLVCPDLGI--------------L------VWNYFQG

IHN-YLKQKKYSTCAWEVVRVEITARLFLM--------------------------

>Ereu_8

MFQ--FLLMTG------------------VMLSSILACS------YGQD----HP-EK--

RVILMLLTQLKNTPSLSCLKDRTDFHPWSRG-------------EI-T----Q------I

HMAQ--GPCF-----Q-K----LMLQQVFRLFN-TEA-SRAAWNNSLLDRLLSSLYDSLE

QL-----EQMEV-----------SLACPSVGT--------------D------ALKYFQR

IKI-YLKAKKYSACAWEIVRSEIEARFFLI-LGTLRR--LGQRTESLGPP-LRAA-

>IFNB_human

MTNKCLLQIAL------------------LLCFSTTALS------MSYNLLGFLQ-RSSN

FQCQKLLWQL-NGRLEYCLKDRMNFDPEEIK-------------QL-Q----Q------F

QKED--AALT-----I-Y----EMLQNIFAIFR-QDS-SSTGWNETIVENLLANVYHQIN

HLKTVL--EELEKE--------DFTRGKMSSL--------------H------LKRYYGR

ILH-YLKAKEYSHCAWTIVRVEILRNFYFI-NRLTGY--LRN--------------

>IFNB_pig

MANKCILQIAL------------------LMCFSTTALS------MSYDVLRYQQ-RSSN

LACQKLLGQL-PGTPQYCLEDRMNFEPEEIM-------------QP-P----Q------F

QKED--AVLI-----I-H----EMLQQIFGILR-RNF-SSTGWNETVIKTILVELDGQMD

DLETIL--EEMEEE--------NFPRGDMTIL--------------H------LKKYYLS

ILQ-YLKSKEYRSCAWTVVQVEILRNFSFL-NRLTDY--LRN--------------

>Oror_6

MNHRCILQTAL------------------LLCFSTTALS------MSYRLLQFQQ-RSSN

LACQKLLQRL-PGMPQHCLEDRMDFKPEEIK-------------QP-Q----Q------F

RKED--AVLV-----T-Y----EMLQQIFGILR-RNF-SSTGWTETITENLLVEVYGQMD

RLETIL--EEMEKE--------NF-TSVVTIL--------------H------LKKYYLQ

IMQ-YLKSKEYSNCAWTVVRVEILRNFSFL-NRLTDY--LHN--------------

>Dano_11

MANRCAFQIAL------------------LLSFSTMALC------ISYNVLRFQQ-SSSN

LICQKLLKKL-NGSAEYCLQDRMDFKPEEIK-------------QP-Q----Q------F

QKEE--AALL-----I-Y----EMLQQIFGIFQ-RKF-SSTGWNETIVENLCVELYQQMD

RLETIL--EELEEE--------SFTWGDMTIL--------------H------LKNYYLR

ITQ-YLKAKEYSSCAWTVVRVEILRNFSFI-NRLTEY--LQN--------------

>Ptva_12

MTNRCILQFAL------------------LLCFSTTALS------MSYNWLRFQQ-RSSN

LACLKLLWQL-NGTPQYCHKDRMDFKPAEIK-------------QP-Q----Q------F

QKED--TVLI-----I-H----EMLRQIFDIFQ-RNF-SSTGWNETIIMNLYVTLSGQMD

RLETAM--EEMEEE--------NFTWESMTVL--------------H------LKNYYFR

IMR-YLETKLYSRCAWTVVKAEILRNFFFL-NGLTEY--LQN--------------

>Loaf_13

MTTRCILQVAL------------------LLSISTTALA------RSYKLLQFQQ-RSSN

LACQKLLWKL-NGAPESCLEDRMDFKPEEIK-------------QP-G----Q------L

QKED--AALV-----I-Y----EMLLQIFDIFL-GNF-SHTGWDETVIENLLAELSQQRD

RLVTIL--EEMEEE--------NPTSRNMTIL--------------H------LKNYYLG

IGQ-YLEAKDYSSCAWTVVQVEILRNFSFI-SGLTDY--LQN--------------

>IFNB_horse

MTYRWILPMAL------------------LLCFSTTALS------VNYDLLRSQL-RSSN

SACLMLLRQL-NGAPQRCPEDTMNFQPEEIE-------------QA-Q----Q------F

QKED--AALV-----I-Y----EMLQHTWRIFR-RNF-ASTGWNETIVKNLLVEVHLQMD

RLETNL--EEMEEE--------SSTWGNTTIL--------------R------LKKYYGR

ISQ-YLKAKKYSHCAWTVVQAEMLRNLAFL-NGLTDY--LQN--------------

>IFNB_cow

MTYRCLLQMVL------------------LLCFSTTALS------RSYSLLRFQQ-RQSL

KECQKLLGQL-PSTSQHCLEARMDFQPEEMK-------------QE-Q----Q------F

QKED--AILV-----M-Y----EVLQHIFGILT-RDF-SSTGWSETIIEDLLKELYWQMN

RLQPIQ--KEMQKQ--------NSTTEDTIVP--------------H------LGKYYFN

LMQ-YLESKEYDRCAWTVVQVQILTNVSFL-MRLTGY--VRD--------------

>Ereu_12

MANRYIFQIAL------------------LLCI-TTALA------ESYTLDQSQQ-KSSI

LVCQDLLNQL-NGSATDCLKQRMNSKPEEIK-------------NP-Q----L------L

QKED--LVLV-----T-Y----ELFQQIFGIFS-RNF-SRTSWNETIVEKLLMELYQQKN

QLKTTV--EEIKET--------NDIWGNKHIL--------------N------LKKYYFS

LMR-YLKANKYSSCAWIIIKTEIIRNFVYL-DKLISY--FSN--------------

>IFNB_mouse

MNNRWILHAAF------------------LLCFSTTALS------INYKQLQLQE-RTNI

RKCQELLEQL-NGKIN--LTYRADFKPMEM---------------T-E----K------M

QKSY--TAFA-----I-Q----EMLQNVFLVFR-NNF-SSTGWNETIVVRLLDELHQQTV

FLKTVL--EEKQEE--------RLTWESSTAL--------------H------LKSYYWR

VQR-YLKLMKYNSYAWMVVRAEIFRNFLII-RRLTRN--FQN--------------

>Modo_7

MVYRGILYLAL------------------LLLFSPSISS------KGYDSLRFHQ-RRTN

QRSLMFLNKMIGKLHPECLQERMDFQPREIV-------------QP-R----Q------C

QREN--ATMI-----I-H----EMLQQTLILFS-SKN-ACPDVNDTIIEPFLSGIYQQML

HLE----EEMDQAN--------SSWESLESIL--------------R------LKNYYQG

ITN-YLKNKEYSSCACKIVQVETRRNFSFL-YKLTEY--LKN--------------

>IFN1_anoli

-----LLPIAL------------------TMVLITEVSS------QDCGQLLARL-RQAN

KANLELLNSKMNSTPQQCIEGVFSFSLKNKL-------------TNRD----V------S

EEEN--AKVA-----I-Q----EVLQQTGHIFR-QNC-TEMLWDEDSLRAFHAGLDQQSE

NLKSCL----------------SASIQL-TSL--------------R------VKRYFRS

LND-FLKEKEYNRCAWEIIQIQVKQCFLWI-EKLIQE--IQSK---------MAH-

>Opha_2

-----FLQICL------------------VMF-FTNVSS------QHCDQLHSRL-QEDN

KGNLELLGSHMRATPLECIGDIADFS-EENV------------MSMNE----A------S

HEED--AKIA-----I-Q----EMLQQTDLIFK-KVH-AELFWDETSLRTFHTGLDQQIK

RLETCQ----------------NASLQL-TRL--------------R------VKRYFQG

LND-FLKDKQYSSCAWEIVQIQLRECFLLI-HQLIQR--IPTQ---------IKY-

>Vibe_2

------------------------------MF-FTKISS------QHCDQLHTRL-LEAN

KGNLELLGSHMRATPLQCIGDIVDFS-EEHL------------ISVDE----A------S

HEED--AKRA-----V-R----EMLQQTDLSFK-QAH-AELFWDENSLRQFHTGLDEQIK

KLETCQ----------------SASLQL-TRL--------------R------VKRYFQG

LNH-FLKEKKYSLCAWEIVQIQLRECFLLI-HQLIQR--IPIQ---------IMY-

>Pybi_2

-----LLQICL------------------AMF-FTKISS------QHCDQLHRRL-HKAS

KGNLKLLGSNIRATPLQCIGDIIDFS-EENL------------MSMDG----A------S

HEEN--AKIT-----I-Q----EMLQQIDLIFK-QVH-AELFWDENSLRQFHTGLYQQIK

ELEICQ----------------NASLQL-TRL--------------R------VKRYFQR

LSD-YLKDKKYSLCAWEIVQIQLRECFLLI-NELIQR--IPT-----------LY-

>Opha_6

-----CLFICL------------------GVF-FTEISP------QDCNQIRSRL-HEAN

LRNMNLPMRNMGSTPQQCIRDIIDFSLEENL------------TNMIN----E------L

QGET--AKVA-----I-K----ELLQQIDLIFK-ESH-SELAWDENSLREFHIGLHQEIK

NTKACW----------------NTSLQF-TRL--------------R------VKRYFQR

LRD-FLKNKEYNLCAWKIMQIQIRECFEWI-NHLNQR--IPSE---------T---

>Vibe_6

-----CLFICL------------------GIF-FTEISS------QDCNQLRSRL-HEAN

LGNLNLLTRNIGSTPQQCIRDIIDSSFEENL------------MNMVN----K------L

QGEN--AKVA-----I-K----ELLQQIDLIFK-ESH-SELVWDENSLREFHIELDQEIK

KAETCW----------------NTSLQF-TRL--------------R------VKRYFQR

LRH-FLKNKEYNLCAWKIIQIQVRECFEWI-NQLNQR--IPSE---------T---

>Opha_7

-----CLYICL------------------GIIFFGDISC------QNCNQLQRKL-LKAN

KDNSNLLSSNIRPTPLQCMRSFVELSLKKIM------------IDMND----E------C

QVDI--AKTA-----V-K----EILQQIDVIFR-QNH-TELVWHEGSLRDFHIGLDQQIK

MLETCG----------------NASLQL-TRL--------------R------IKRYFQR

LSD-FLKNKKYSLCAWEIVQIQIEACFQLI-NHYIQR--IRSK--------TMKK-

>IFN5_anoli

MAQQCLLAFCL------------------LMSF-REILS------QDCNDLRHEL-NGAN

KANLELLNVKMGSTPLQCVDDVINFSSKESL-------------PSIY----D------F

EEEN--ATVA-----I-D----EILQQISYLFN-QNH-TKLSWDENSIATFKLGVDNEIK

KLTPCL----------------SDSIDE-LRD--------------K------VRKYFER

INN-LLKEKEYNLCAWEIVQMEVRQCLIVV-DQLISR--IPKK---------KAV-

>Chmy_15

-----LPRVCL------------------VLLFFTEISS------RLCTMLHFQQ-KKMN

RESLEHL-KKMSGNPSQCINERAASKP-QDV-------------AQLP----V------S

QKEK--A--------------------IFSIFS-KNL-TQSAW---------NGLL----

------------------------------------------------------------

--------------------------------------------------------

>chpi_1

-------------------------------------------------MLHFQE-NKGN

KESVELL-KKMSENLSQFINEMKAFKP-QDV-------------VQLQ----L------S

QKEN--ADVA-----I-Q----EILQEIFTIFS-KNL-TQTAWDRSSIARFQNGLYQQIQ

PLEVCL----------------GA------------------------------------

--------------------------------------------------------

>Almi_3

-----LLHICL------------------VLLFSTEISS------QHCDLLSFQQ-KKLN

KDSLELL-EKMGGNPFQCFSERTDFKS-QDV-------------LKLQ----L------S

QKEN--AKLA-----I-Q----NILQEIFTVFS-KNL-TQTAWDEISIITFQNKLHQQIE

RLEACL----------------GFKLVL-TKL--------------K------IKRYFQG

IYN-FLEEKQYSLCAWEIIRMEMTRGFLLV-DQLTKS--I----------------

>Crpo_3

-MKMMLLHICL------------------ILLFSTEISS------QHCDLLSFQQ-KKLN

KDSLELL-EKMGGNPFQCFNEGIDFKS-QDV-------------LKRQ----S------S

QKEN--VKLA-----I-Q----NILQEIFTVFS-KNL-TQTAWDTISIITFQNKLHQQIE

RLEACL----------------GFKLVL-TKL--------------K------VRRYFQG

IYN-FLEEKQYSLCAWEIIRMEMTRCFLFV-DQLTKS--L----------------

>Gavga_8

FLSSFLLHVCL------------------VLLFSTEISS------LHCGLLNFQQ-KKLN

KDSLELL-DNMGGNPSQCSNERIDFKP-QDV-------------LMLR----S------S

LKEN--AKMA-----I-Q----EILQEVFTVFS-KNL-TQTAWDEASIVVFQNGLHWQTE

RLEACL----------------DLKALL-TRL--------------K------LKRYFQG

IRN-FLEGK-----------------------------------------------

>Crpo_4

FLSSFLLHVCL------------------VLLFSTEISS------LHCGLLNFQQ-KKLN

KDSLELL-DNMGGNPSQCSSERTDFKP-QDI-------------LMLR----S------S

LKEN--AKMA-----I-Q----EILQEVFTVFS-KNL-TQTAWDEASIVAFQNGLHWQTE

RLEACL----------------DLKVLL-TRL--------------K------LKRYFQG

IRN-FLEGKQYSLCAWEIIRLEMPRCFLLL-DLLTKW--LKI--------------

>Gavga_6

---------------------------------------------------------KIE

Q-SLELL-DKIGRKPSQ-SNEKIDFKP-QDV-------------LVLG----S------T

LKEN--AQMA-----I-Q----EALQGVFTVFG-KNL-MQTAWDETFIVMFQNGLHWQIK

KLEACL----------------GLKVLL-TRV--------------K------LKNYFQG

ICN-FLEGKQYSLCAWEIILLEMHRYFLLL-DQLTKW--LK---------------

>Almi_11

----------L------------------VNLYHAR---------------KQER-MKIN

K-------EKRSPHDISRKYKKIDFKP-QDV-------------LILH----S------T

LKEN--AKMA-----I-Q----EILQGVFTVFS-KNL-MQTAWDETSIVMFQNGLHWQIK

RLEACS----------------GWEVLL-TRL--------------K------LKSYFQG

I-------------------------------------------------------

>Chmy_2

-----LLHICL------------------VLLFSIEISS------LDCNMLHFQQ-NKMN

MESLELL-SKMGGQPLQCLNENRNFRF-QKA-------------LRPR----E------S

QEKN--AKVV-----I-Q----EILQQIFNIFS-KNL-TQAAWDRSSVETLQKGLHQQTE

QLETCL----------------YS-LLF-PML--------------K------LKKYFQR

IRD-FLKEKQYSLCAWETIRLEMGRCFFFV-DQLIIR--LQN--------------

>Chpi_17

-----LLHICL------------------IMLFSTEISS------LDCTILHFQQ-NKMN

MESLELL-SKMGGQPLQCLNENRNFRL-QKA-------------LRPR----E------S

QEKN--AKMV-----I-Q----EILQQIFNIFS-KNL-TQAAWDRSSVETLQNGLHQQTE

KLETCL----------------HLYLLF-PML--------------K------LKKYFQR

IRD-FLKEKQYSLCAWETIRLEMGRCLLFV-DQLIKR--L----------------

>Chmy_1

-----FLHICL------------------VLLFSTKTSS------VDSNMLHFQQ-NKVN

QASLQLL-EKMGGQPVQCLNENSNFIS-QNV-------------LSSR----E------F

QKES--VIVA-----L-Q----EILQQIFNIFS-KSQ-LQTAWDRSSMDAFQNGLHHQIE

LLKTWF----------------NEY--F-TIL--------------K------VKKYFHV

IDN-FLKEKQYSLCASEIIREEMRRCFLFI-DQLTKR--LKN--------------

>Chpi_2

-----FLHICL------------------VLLF-TENSS------VDCNMLHFQQ-NKVN

QASLQLL-EKMGGQPVQCLNENSNFIS-QNV-------------LSSR----E------F

QKEN--AMVA-----I-Q----EILQQIFNIFS-KSH-IQTAWDRSSIVAFQNGLHQQIE

LLKTWF----------------DGY--F-TRL--------------K------VKKYFHV

VDN-FLKEKQYSLCAWEIIREEMRKCFLIM-DQLTKR--LKN--------------

>Pesi_2

-----FLHICL------------------VLHISTKISF------VDCNMFLFQQ-NKVN

QDSLKLL-EKMGGQPVQCLNEKSNFIS-QNL-------------FSST----E------F

QKEN--AMMV-----I-Q----EILQQSFTIFR-KIQ-IQTDWDRSSIAAFQNGLYQQIE

LLKTWF----------------DGY--L-TRL--------------K------VKKYFHV

IDT-FLEKRQYSRCACEIIREEMRRCFLFI-DQLTKR--LKN--------------

>Chpi_8

----------------------------------------------------------MN

SESLEHL-EKMGGNPFQCLNERTAFKP-RDI-------------LKIR----L------S

QQEN--AKVA-----I-Q----QILQELFHIFN-NNL-TQAAWNGTSIKEFQNGLHQQIE

KLETCL----------------SAYLLL-TSL--------------K------LKRYFQT

IDD-FLKEKQYSQCAWEIIRVEISRCFPIL-NILTKR--LQDE--E------LKY-

>Pesi_3

----------------------------------------------------------MN

SKSLEHL-EKMGGPPFQCLNERSAFKA-TDI-------------LKVR----L------A

QQEN--AKAA-----I-Q----QILQELFQIFS-KNL-THAAWDGTSIKEFQNGIHQQIE

KLEVCL----------------SAYLLH-TSL--------------K------LRRYFQT

VRH-FLKEKQYSRCAWEIIRLEVSRCFLVL-NILTKR--IEN--------------

>Almi_2

-----LLKFFL------------------VLLLFKVSSS------LHCNSLASNQ-NKVN

KDGLDFL-DKMRRNSPQCLSERLDLKT-KDI-------------FKIE----L------S

QKDN--AKAA-----I-Q----ELLKAIFYVLS-NNL-TQTTWQESSIEKFKNGLHWQIE

NLETCL----------------DASPLV-TRL--------------K------LKRYFQA

IDN-FLKEKQYSQCAWEIISVELSRCFQFI-DKLTIK--LSTS-------------

>Crpo_5

-----LLKFFL------------------VLLFFKVSSS------LHCSSLASNQ-NKVN

KDGLDFL-DKMRRNSPQCLSERLDLKT-KDI-------------FKIE----P------S

QKHN--AKAA-----I-Q----ELLKAIFYVFS-KNV-TQMTWQESSIEKFKNGLHWQIE

NLETCL----------------DASPLV-TRL--------------K------LKRYFQA

IDN-FLKEKQYSQCAWVIISVELSRCFQFI-DKLTKK--L----------------

>Gavga_7

-------------------------------------SS------LHCSSLASNQ-NKVN

KDGLDFL-DKMRRNSPQCLSERLDLKT-KDI-------------FKIE----L------S

QKHN--AKAA-----I-Q----ELLKAIFYVFS-KNL-TQTTWQESSIEKFKNGLHWQIE

NLETCL----------------DAGPLV-TRL--------------K------LKRYFQA

IDN-FLKEKQYSQCAWEIISVELSRCFQFI-DKLTKK--LRYC-------------

>Almi_1

MMKNKLLHICL------------------VLLFSTEITS------MQCDMIHFQQ-KRLN

KDSLELL-EKMGGSPFQCSNEN--------------------------------------

--------------------------QIQDYPG-KNL-TQTTWDWSSIVTFQNGLHRQIQ

LLEACL----------------VATQLH-TRL--------------K------LNRYFQR

IHN-FLEEKHYNLCAWEIIRIEMPNCFLFV-DQLTKS--LKN--------------

>Chmy_5

-------------------------------------MS------LDCNLLRHQQ-SKFN

WYSLQLL-QNMGGKPLECLEDKTAFQF-EKI-------------LKPK----F------L

QQ----AQMS-----V-H----EILEQLFGIFS-RNL-SQTGWERRKVERFLNGLALQTE

RLEECL----------------HT-------L--------------R------LKKYFQR

IQD-FLKEKKYSTCAWEIVREEGQRCFQYI-HKLTVR--MKN--------------

>Chpi_4

-----LWQICL------------------VLLFSAGVMS------LDCNLLRHQQ-SKFN

GYSLQLL-QNMGGNPLKCLEDKTAFQF-EKV-------------LKPK----F------Q

QH----AKMA-----I-H----EILQQLFGIFS-RNL-TQTGWERTKVGSFLNGLTLQTE

RLETCL----------------PT-------L--------------R------LKKYFQR

IQD-FLNEKKYSTCAWEIVREEGQRCFQYI-DKLTVR--MKK--------------

>Pesi_4

-----LWQLCQ------------------LLLFSAGVMS------LDCNLLHHQQ-SKFN

RYSLQLL-QKTGRSPLECLGDLTAFQF-EKV-------------LKHK----F------P

QH----AQMA-----A-H----EILQQLFGIFS-RNL-LQTRWEKGDVELFRNGLHLQTK

HLEKCL----------------ST-------L--------------R------LKRYFQR

IKD-FLEKKKYSTCAWETVRLEAQRCFLYM-DKLTVM--MKN--------------

>Chmy_4

-----LLQISL------------------VLLCTTKIST------LDCNTLPLLH-NKVI

QGNLHLL-NKMGQQPEQCQSEKMHFKF-EQF-------------LKLR------------

QKEN--AKVE-----I-Q----EILQQTFYVFT-KNL-TLAAWDGRALERFQNRLNQQIE

HLEACL----------------TEY--I-IRL--------------K------LKKYFQK

IDN-FLKDKQYSLCSWEIIRLEMRRCLQFI-DKVIRR--LRN--------------

>Pesi_1

-----LLQISF------------------MLLCTTNISA------LDCNILPLLH-NKMI

QGHLHVL-NKMGQQPEQCQSEKMHFQF-EKF-------------LKLR------------

KKEN--AKVA-----I-H----EILLQIFYIFT-KHL-TLVAWDGRSLERFQNGVNQQIE

HLDACL----------------TEY--I-IRL--------------K------LKKHFQK

LDN-FLKDKHYSLCSWEIIRLEIRRYLHFI-VKVTRR--LRN--------------

>Coli_2

-----LIQIGL------------------IVLCITIISS------HQCNHLPLQQ-RKAI

ENSLQLL-DKMGEKPQRCLREKMSFKF-KQV-------------LKPT------------

QKEA--VEVA-----I-E----EIFQHIFYIFS-KNL-TLAAWDGTALEKFQNGLYHQIE

QLEACV------------------Y--V-NRL--------------K------LKKYFQK

IDC-FLKDKQHNLCSWEISRAEMRRCLQLI-DKVIRK--LYKV-----------H-

>Apfo_3

-----LTQIGL------------------ILLCTTTISS------LQCNHLHLQQ-RKVI

ENSLQLL-DKMGEKPQQCLKEKMSFRF-EQV-------------LKPR------------

QKET--VKVV-----I-E----EIFQHIFYIFS-KNL-TLAAWDGTALEQFQNGLYQQIE

QLEACV------------------Y--V-NRL--------------K------LKKYFQK

IDC-FLKDKKHNLCSWEISRAEMRRCLQLI-DKVIRK--LNN--------------

>Fape_1

-----LIQIGL------------------ILLCTTTISS------LQCNHLPLQQ-GKVV

ENSLKLL-DKMGKKPQQCLREKMSFRF-EQV-------------LKPR------------

QKEN--VEVV-----V-E----EIFQHIFYIFS-KNL-TLAAWDGTALEQFQNGLHQQIE

QLEACV------------------Y--V-NRL--------------K------LKKYFQK

LDC-FLKDKKHDLCSWEISRAELRRCLQLI-DKVIRK--LNN--------------

>Tyal_2

-----LIQIGL------------------ILLCTTTISS------LQCSHLPLQQ-RKVI

KNSLQLL-DKMGKKPRQCLREKMSFRF-KQV-------------LNPR------------

QKET--VKVA-----I-E----EIFQNIFYIFS-KNL-TLAAWDGTALEQFQNGLYQQIE

QLEACV------------------Y--V-NRL--------------K------LKKYFQK

IDC-FLKDKQHNLCSWEISRAEMRRCLQLT-DKVIRK--LNN--------------

>Gaga_3

-----FIQIGF------------------ILLCTITISS------LTCNHLPLQQ-RRVI

ESSLQLL-DKMGRRPQQCLREKMSFRF-EQV-------------LKPR------------

QKET--VKVA-----I-E----EILQHIFYIFS-KNL-TLAAWDGAALEQFQNGLYQQIE

KLEACI------------------Y--V-NRL--------------K------LKKYFQK

IDS-FLKEKQHNLCSWEISRAEMRRCLQLI-DKVIRK--LYK--------------

>Caan_3

-----LIPTGL------------------ILLCTTTISC------LWCNHLPLQQ-RKVI

QNSLQLL-DKMGNKPQQCLKEKMFFSF-EQV-------------LKPR------------

QKES--VKVA-----I-E----EIFQHIFYIFS-RNL-TLAAWDGAALEQFQNGLYQQIE

QLEVCV------------------S--V-NRL--------------K------LKKYFQK

IDC-FLRDKQHNLCSWEISRAEMRKCLQMI-DNVIWK--LNS--------------

>Stca_2

-----LLQIGL------------------ILSCTTNISS------LHCNHLSLQQ-SKVI

ESSLQLL-DKMGEKPQRCLRERMSFRF-EQV-------------LKPR------------

QKET--VKMA-----I-E----EILQHIFHIFS-KNL-TLAAWDGQALEQFQNGLYQQIE

QVEACV------------------Y--A-SRL--------------K------LKKYFQK

IDY-FLKDKQHSMCSWEISRAEMRRCLQFV-DKVIKR--LNN--------------

>Almi_5

--------MFF------------------ILLCTMQIST------PDCNIPSLQQ-SKAI

QSSLHLL-DKIGQAPLQCRREHVLFKF-HNI-------------LKLS------------

QKDN--VKVA-----V-Q----ETLQSIFYMFS-KNL-TLAAWDGRSLESFQNGLYQQIE

QLEACS----------------IKY--A-NRL--------------K------LKKYFQR

IDN-FLKGKQYSLCSWEIIREEVRKCLQLI-EKGLEG--LENK---------IKND

>Gavga_2

--------MFF------------------ILLCTMQIST------LDCNIPSLQQ-SKAI

QSSLHLL-DKIGQAPLQCRREHVPFNF-RNI-------------LKLR------------

QKDN--VKVA-----V-Q----EMLQSIFYMFS-KNL-TLAAWDGRSLESFQNGLYQQIE

QLEACS----------------LKY--D-NRL--------------K------LKKYFQR

IDN-FLKDKQYSLCSWEIIREEVRTCLQLI-ETVTKA-------------------

>Crpo_7

MEVSGLLQMFF------------------ILLCTMQIST------LDCNIPPLQQ-SKAI

QSSLHLL-DKIGQAPLQCRHEHVPFNF-RNI-------------LKLR------------

QKDN--VKVA-----V-Q----EMLQSIFYTFS-KNL-TLAAWDGRSLESFQNGLYQQIE

KLEACS----------------LKY--D-NRL--------------K------LKKYFQR

IDN-FLKDKQYSLCSWEIIREEVRTCLQLI-ETVTKA-------------------

>Chpi_15

-----LADESP------------------VTVLSVH---------------ASGQ-SSSG

RRLLAPL-MPLRSSPGSAW-----------------------------------------

RHQP--SKI---------------LQERFHIFN--NL-TQAPWNGTSIKEFQNGLHQQIE

KLETCL----------------SAYLLL-TSL--------------K------LKRYFQT

IDD-FLKEKQYSLYAWEIIRAEISRCLLIL-DIL----------------------

>Chmy_6

------------------------------------------------------------

----------------------------MAI-------------LKPR------------

EKVN--IVVT-----I-H----KILHETFNLFS-KNL--HAAWNTTCIEKFQNGLHWQIE

QLETCL----------------GANLQS-T-L--------------N------VKKYFQR

IKD-FLKEKHYSHCSWEQY-----FCLLSF-KKKSKN-------------------

>Almi_4

MEEPTFLHVCL------------------VLVFSIKISS------PDCS--RLQR-IKVN

-HSLYLL-CRMGGQPLSCLNDRTDFRI-REI-------------FIIR------------

KKEN--ALMI-----I-H----ELLHHIFQLFS-KNL-PQGAWNPSCIEKFQNGLHWQIE

QLEKCF----------------GGNLQN-NIL--------------K------AKKYFQR

ISH-FLNEKNYSRCSWETARMEMRRCFLFL-DHLLKN--LRN--------------

>Gavga_3

MEEPAFLHVCL------------------VLVFSIKISS------PDCS--RLRQ-IKVN

-QSLHLL-CRMGGEPLSCLNDRPHFRI-RQI-------------FTAR------------

NKEN--ALMI-----I-H----ELLHHIFQLFS-KNL-PQGLWNPSCIEKFQNGLHWQIE

QLQTCF----------------GGDLQN-NIL--------------K------VKKYFQR

ISH-FLNEKNYSRCSWETARMEMRICFLFL-DHLLKK--FRN--------------

>Crpo_1

MEEPTFLHVCL------------------VLVFSIKISS------PDCS--RLQQ-IKVN

-QSLHLL-CRMGGEPLSCLNDRTDFRI-REI-------------FTAR------------

KKED--ALMI-----I-H----ELLHHIFQLFS-KNL-PQGPWNPSCIEKFQNGLHWQIE

QLETCF----------------GGDLQN-NIL--------------K------VKKYFQR

ISH-FLNEKNYSRCSWETARMEMRRCFLFL-DHLLKK--FRK--------------

>Tyal_1

TDKTTLLRVCV------------------TLALYIKISH------PVC---LFQG-IKVN

YNNMNFL-WTMGGYSQQCLSEATDFRF-MEI-------------TKVT------------

QK-N--VTMI-----I-Y----EFLQQTFQLFS-KNL-PAGAWNTSKIQKFQNGIHQQIE

ELEVCL----------------LEILKS-TTF--------------S------VKKYFQR

ITD-FLKDKKYSHCSWEAVQMELRSCLIIF-DSLLKK--HTS--------------

>Apfo_2

-----LLRVCI------------------TLALYVKISH------PAC---LFQG-IRMN

YHNMNLL-CKMGGYSQQCPSETTDFRF-MEI-------------TKIT------------

QK-N--VTVI-----T-C----KFLQQIFQLFS-KNL-PVGAWNTSNIEKFQNGIHHQIE

ELETCL----------------SESLRS-TTL--------------S------MKKYFQR

ITN-FLKDKQXSHCSWEAVRMELRTCFIIF-DI-----------------------

>Stca_3

-----LLQVCI------------------TLALYTKISH------PVC---LFQG-SKVN

YQNMNFL-CKMGASPQQCLRERTDFKF-MEI-------------TKVR------------

QR-N--AIVM-----I-H----ELLRQIFHLFS-KNL-PESVWNASCIEKFQNGIHQQIE

ELETCL----------------VESLNS-TTL--------------R------VKKYFRR

ITS-FLENKQYSHCSWEAVRMEVRTCFIFI-DCLMRK--HMA--------------

>Chpi_6

------------------------------ML-----------------MDVFQE-----

--------QTSSEKP-----------P-RDA-------------F-IP----V------I

KRAN--YE-W-----L-H----EILQQIFNIFS-KNL-TQSAWDGTSIVRFQNGLYQQIQ

RLEACL----------------RANLQL-TSR--------------R------VKKYFQG

IDA-FLKEKQYSLCAWEIIRMEISRSFVLI-DKLTRS--LSN--------------

>Almi_7

-----LWKICL------------------VALLSAHVAA------LDCSNFRDLQ-KVLN

RNSMQLL-GQVAGAPEECLEDRPTFRF-DKV-------------LRSK------------

APHN--AWMA-----T-Y----EILQKLFSLFK-RTL-PETAWDTRSVERLLNAVHLQIK

RLETCP------------------Y----KAT--------------G------LKKYFRK

IDD-FLRAKNYSKCAWEVVRIEAKTWFYYL-DKLKNR--LN---------------

>Gavga_1

-----LWQICL------------------AALLLAHVTA------LDCSNLKDLQ-EVLN

RNSVQLL-GQVAGAPEECLEDRPTFRF-EKV-------------LRSK------------

APHN--AWMA-----T-Y----EILQQLFSLFK-RNL-PETAWDTRSMERFLNAVHVQIK

RLETCP------------------Y----KAM--------------K------LKKYFRN

IHD-FLQEKNYSKCAWEVVRIEAKTWFYYL-DKFRNR--L----------------

>Crpo_2

-----LWQICL------------------AALLLARVTA------LDCSNFKDLQ-EVLN

RNSMQLL-GQVAGAPEECLEDRPTFRF-EKV-------------LRSK------------

APHN--AWMA-----S-Y----EILQQLFSLFK-GNL-PETAWDMHSVERFLNAVHVQIK

RLETCL------------------Y----KAM--------------K------LKKYFRN

IHN-FLREKNYSKCAWEVVRIEAKTWFYYL-DKFGNR--L----------------

>Chmy_14

-----MAYQVP------------------TQP---------------------GM-TSIS

KSAFSAL-QK----PSSC------------------------------------------

------SK-----------------------------------NGSSIKVFQNGLHQQIE

KLETCL----------------SACLLL-TSL--------------K------LKRYFQT

IDD-FLKEKQYSRCAG---RSSVRKYPDVF-SFLTY--------------------

>Opha_5

-----TLKVCL------------------VLVLFAKSSV------FRCSIFHAQW-KQLI

QNNLPHL-CRTNEEPLQCIYELTDFNF-LEV-------------LEVT------------

NRDS--ADI------I-Y----ELLQQISYLLS-NA----HAWNSTCFENLKNGLHQQIK

NLETCL----------------NVNSYL-LTL--------------K------VKRYFQR

MNN-FLTVKQHSSCSWEMIHSEIKGCMLFI-THLLKK--L----------------

>Thsi_2

-----ILKVCL------------------VLVLLAKFLV------FRCSIFHAQW-KQLI

QNNLPHL-CRTNEEPLQCIYELTDFGF-LEV-------------LEVS------------

NRDN--AVI------I-Y----ELLQQISCLLS-NAP-SDHAWNSTCFENLKNALYEQMK

NLQACL----------------NAKSYL-LTL--------------K------VKRYFQR

MNN-FLTL------------------------------------------------

>Pybi_1

-----TLKICL------------------VLVLFAKLSV------FQCSIFHAQW-KQMI

QNNLHQL-CRANEEPLKCLPEVTDFRF-LEV-------------LKVS------------

NRGN--AII------I-Y----EILQQISCLLS-KGH-SYNVWNSTCFENLQNALHQQMK

TLATCL----------------NATSYF-LTL--------------K------VKKYFQR

MND-FLTVKQYSSCSWEMIHSEIKGCMLFI-IHLLNK--LKQ--------------

>IFNK_human

MIQKCLWLEIL------------------MGIFIAGTLS------LDCNLLNVHL-RRVT

WQNLRHLSSMSNSFPVECLRENIAFEPQEFL-------------QY-T----Q------P

MKRD--IKKA-----F-Y----EMSLQAFNIFS-QHT-FKY-WKERHLKQIQIGLDQQAE

YLNQCLEEDKNEED--------MKEMKENEML--------------E------LRRYFHR

IDN-FLKEKKYSDCAWEIVRVEIRRCLYYF-YKFTAL--FRRK-------------

>IFNK_dog

VIRKCLWPACL------------------VGLLITGVLS------LDCNLLHFHL-RKVT

WQNLRLLSSMSNSFPVECLREIKAFEPQEIL-------------SH-T----Q------P

VKRY--IVEA-----F-Y----EMSIQAFNIFS-QYT-FKSTWENDYLKQIQIGLDQQLQ

YAERCLEEEEKEDD--------SKEMEEDGIL--------------E------LRRYFNR

IDN-FLKEKKYSHCAWEIIRVEIRRCFYYY-FKFAPL--LRKK-------------

>Ptva_13

MIRKCLWPACL------------------MGLLITGILS------LDCNLLN-----RVT

WQNLKLLSSMRNSFPKDCLRENKAFEPQEIL-------------YS-T----Q------L

LKRD--IKEA-----F-Y----EISLQAFDIFS-QYT-FQSTWKKKYLKRIQIGLDRQLQ

YLEQCLEEEEKNED--------MKEMEEDESL--------------E------LKRYFHR

ISS-FLKDKKYSHCAWEIVRVEIRRCFYY----FTAL--LRKK-------------

>Dano_13

MIQKCLWPACL------------------MDLFITGILS------LDCDLLNVHL-SRVT

WQNLRVLRSMSNSFPLKCLRETEAFEPQEIL-------------SN-T----Q------P

VRRD--IKEV-----F-Y----EMSTQAFNIFS-QYT-FNSTWEEKHLKQIQIGLDRQIE

YVEQCLEDEEKNED--------MKQMEEDEML--------------E------LRRYFNR

INK-FLKDKKHSHCAWEIVLVEIRRCFCY--FKFTAL--L----------------

>Oror_5

VIRKCMWPVCL------------------MGLFVTGILS------LDCNLLNVHL-RRVT

WKNLSLLRRMSKSFPIECLRESKAFEPQEIL-------------SH-T----Q------P

LTRD--IKEA-----F-Y----EMSRQAFHIFI-QDT-FKSTWEEKHLRQVQIGLDQQLQ

YLEQCLEEEE-NED--------MREVAEDERL--------------E------LRRYFNR

IDR-FLKDKKYSHCAWEIVRVEIRRCFYF--FKFTAL--LRRK-------------

>Loaf_16

VIRKYFWPICL------------------VGLFLTSVLS------QSCDLLYVHL-NRVT

WQNLKLLSHMSNPFPVECLKEKKAFEPQEIL-------------SH-T----Q------P

VKRH--IEEA-----F-Y----EISSQVFNIFS-QHA-CKSAWDEKHLKQIQIGLHQQVE

YLERCLEEEEKSED--------MKQMEE-KIL--------------K------LRRYFNR

LGN-FLKDKKYSQCAWEIVLVEIRRCVFYY-FKFTTL--LRKK-------------

>Ereu_11

LIRKCLWSSCL------------------VYLFLTGIHS------LDCSFLNIQL-RRVT

GQNARLMSSMKGPLRQECLKDINNFEPEEIF-------------LC-N----Q------S

KKWN--IKVN-----F-Y----EIYANAFRIFS-QYT-VKYSWEEECMQQILMELNLQLE

SLEQCLKEEK----------------ENDEIL--------------K------LKRYFFR

IQS-YLRDKKYSDCAWKIVFVEIGRCFYHS-LKLTRL--SRKK-------------

>IFNK_mouse

MTPKFLWLVAL------------------VALYIPPIQS------LNC----VYL-DDSI

LENVKLLGSTMTGFPLRCLKDITDFKPKEIL-------------PY-I----Q------H

MKRE--INAV-----S-Y----RISSLALTIFN-LKG-SIPPVTEEHWERIRSGLFKQVR

QAQECFMDEEKE----------------REHL--------------E------LGKYFFR

IKK-FLINKKYSFCAWKIVTVEIRRCFIIF-SKSRKL--LKMK-------------

>Opha_1

MISGCFQHIVL------------------FLLLSSGIRS------LDCNHI-VKQ-QSRT

VNIMKLLESMG---PWQCFNKIQDFAP-NDTG---------------------------S

IKED--ARAT-----F-G----LMLEQINRMFW-QNF-TKAEWNVTVTEHLQTSLDQQLV

QWEKCV----AEGK------KATK---DRIKL--------------K------LRKYFLR

LDT-FLKDEEYSSCAWEAVRHEIMGIQVFL-DQLLRT--LQR--------------

>Thsi_6

MISGCFQYIVL------------------LLLLSSGIRS------LNCNHI-VKQ-KDET

MNTIKLLESMG---PWQCFNQIQDFAP-NDTG---------------------------S

IKED--ARAT-----V-G----LMLEQINRIFS-QNF-TKAEWNMTITEHFQISLDQQLV

QWEKCV----AEGK------KATK---DRTKL--------------K------LRKYFLR

LDR-FLKDEEYSSCAWEAVRHEIMGIQFFL-DQLLRT--LQH--------------

>Vibe_7

MISGCFQYVVL------------------LFLLSSGVRS------LDCNHI-VKQ-KGAT

VNIMKLLEAMG---PLECFHKIPDFAP-NNIG---------------------------S

IKED--AKAT-----I-G----LMLEQISRIFS-QNF-TQTEWNMTVAEHFQIALDQQIV

QWEKCV----TEGN------KATK---ERTKL--------------K------LRSYFLR

LDT-FLKDEEYSSCAWEAVRQEIKGIPLFL-DRLLRT--LQS--------------

>Pybi_3

MVLRCLQHIVL------------------LLLFSSGIVS------LDCNHI-VKQ-KGAT

ADIRKLLEAMG---SLECFHKIPDFNP-KNIG---------------------------S

IKED--ARAT-----V-G----LMLEHIQRIFW-HNF-TKAEWNMTVTELFQIVLDQQVV

QWETCV----TVGE------KATK---DRTKL--------------K------LKKYFLR

LDT-FLKDEEYSPCAWDVVRQEVLGIHFFL-DQLLRT--LQN--------------

>IFN9_anoli

--------MA-----------------------GQQ--S-------------SQ------

----------------ECLED--DFGPRKIL---------------------K-------

SQED--AKMA-----I-G----LILQQIQIVFQ-LNF-TQAQWSGKVTDLLSRALDQQHM

QWRRC-----A-----------TA---EAAKLR-------------S------LKRYFRK

LHT-FLRGRQYSFCAWKMVRYELLVIYPIL-NELMR---LEK--------------

>IFN1_ONMY

--------------MYTMQ--SW-SC---IFL----IICSMQSVCHCCDWIRHHY-GHLS

AEYLPLLDQMGG----DITKQ----------N--AP----VLFPTSLYRHIDD------A

EFED--KVIF-----L-K----ETIYQITKLFDG--NMKSATWDKKNLDDFLNILERQLE

NLNSCV---------------------S-PAMKP------ERRLKR----------YFKK

LSK-VLRKMNYSAQAWELIGKETKRNLQRL-DILAAQ--MY---------------

>IFNA1_SASA

--------------MYTVQ--SW-TC---ICL----IICSMQSVCHCCDWIRHHY-GHLS

SEYLSLLDQMGG----DITKQ----------D--AP----VFFPTSLYRHIDD------A

EVED--QVRF-----L-K----ETIYQITKLFDG--NMKSVTWDKKKLDDFLNILERQLE

NLKSCV---------------------S-PAMKP------EKRLKR----------YFKK

LKN-VLRKMNYSAQAWELIRKETKRHLQRL-DILAAQ--MY---------------

>Eslu_3

-----------------MQ--SW-IF---LFL----ILCRTQSSCSCCDWIRDHY-GTLS

REYLSLLDEMGG----NITKQ----------D--VP----VFFPESLYRLMED------A

QYEV--QVRF-----L-N----ETIHEIIKLFDE--NMDAVTWEEKKLDDFLILLHRQFQ

KLKSCV---------------------S-PAKKA------EGRLES----------FFKK

LKK-VLKEMNYSAQAWELIRKETKYVLEKL-YLLVAT--MHR--------------

>Anja_5

-------------------------------F----IVCSAQDFCDGCYWIQHGF-RRVS

GESLSLLSE---------------------------------------------------

MVDD--KVKF-----V-H----ASIDQIIKLFDE--NLDAVTWNRLKLEHFLIVLDRQSR

ELQKCV---------------------S-RCSII------RK------------------

--------------------------------------------------------

>IFN1_DARE

---------------------MW-TY---IFV----IYVILQSQSSACEWLG-RY-RIIT

TESLNLLKNMGG----KYA-D-------------LE----TPFPSRLYTLMDK------S

KVED--QVKF-----L-V----LTLDHIIHLMDAREHMNSVNWDQNTVEDFLNILHRKSS

DLKECV---------------------A-RYAKPAHKESYEIRIKR----------HFRT

LKK-ILKKKQYSAEAWEQIRRVVKSHLQRM-DIIASN--ARVN-------P-----

>IFN_CAAU

---------------MKTQ--MW-TY---MFV----MFLTLQGQCSACEWLG-RY-RMIS

NESLSLLKEMGG----KYP-E----------G--TK----VSFPGRLYNMIDN------A

KVED--QVKF-----L-V----LTLDHIIRLMDAREHMNSVQWNLQTVEHFLTVLNRQSS

DLKECV---------------------A-RY-QPSHKESYEKKINR----------HFKI

LKK-NLKKKEYSAQAWEQIRRAVKHHLQRM-DIIASN--RR---------------

>IFN_CTID

---------------MKTQ--MW-TY---MFV----MFLTLQGQCSACEWLG-RY-RMIS

NESLSLLKEMGG----KYP-E----------G--TK----VSFPGRLYNMIDN------A

KVED--QVKF-----L-V----LTLDHIIRLMDAREHMNSVQWNLQTVEHFLTVLNRQSS

DLKECV---------------------A-RY-QPSHKESYEKKINR----------HFKI

LKK-NLKKKEYSAQAWEQIRRAVKHHLQRM-DIIASN--RR---------------

>IFN_CYCA

--------------MNQTQ--MW-TC---IFV----IFLTLQSQCSACRWLG-RY-GTVS

ADSLNLLREMSG----QYP-E----------N--VK----MHFPGTLYNLIDK------A

EVED--QVRF-----L-V----LTLDHIINLMDASEHMNSAKWNLKKVEYFLEDLQRQSS

ELKECV---------------------A-QYQKPLQKESYEIRIKR----------HFRT

LKK-ILKKEKYSAQAWEQIRRAVRSHLQRM-DIIANN--AKKR-------V-----

>IFN2_ICPU

--------------MDIKL--SW-IC---LFL----LFFTVQERSEACNWMISQY-RAKN

DYCLSLLNEMGG----EIVPM----------T--GN----TSFPRRAYHEIEK------A

QAED--QVRF-----L-A----VATNEIIILFSAVSHVDDVKWDSRTLDNFLNILSRQLS

ELRNCT---------------------S-TYAERARRSSTEKKLRK----------HFKD

LRK-YLKNSNYSADSLEQIRSVVQRHLWRM-DTIAAI--VKQK-------LLRTN-

>IFN4_ICPU

--------------MDIKQ--SW-IC---LFL----LFFIVQERSEACNWMISQY-RAKN

NFCVSLLKEMGG----EIVQV--------------N----RPFPHKAYSEIDK------A

KAED--QVRF-----L-A----QATEQIISVFN-VSHVDEVKWDRSALDEFLNILNRQLT

ELTKCT---------------------S-TYAERAGHSPTERKLRK----------HFKK

LKK-FLNEANYSADSLERIRNVVQHHLWRM-DIIAAN--VKQK-------LLRTN-

>IFND1_ONMY

----------MHR-T--KS--LL-IC---LFL----TLCN-G-LSVGCRWMDHKF-IQHS

ETLMNVLNIMGG----EFTTD----------S--VD----VPFPEDLYEQAEY------L

PTDD--TIWF-----I-L----QTLDKIAELFD-GEL-NSV-WDEKKVEIFLNVLTSQSD

GLQSCV---------------------R-AQKKN----------SKN------LQMYFKR

LNNHVLKRMAYSAHAWELVRKEVRTHLRRL-VLLGSA--TENR-------I-----

>IFND1_SASA

----------MHR-T--KS--LL-IC---LFL----TMCD-G-FSMGCRWMDHKF-IQHS

ETLMNLLNIMGG----EFTTD----------S--VD----VPFPEDLYKQAEY------L

PTDD--TIWF-----I-L----QTLDKIAELFD-GEL-DSV-WNEKKVEIFLSVLNSQSD

GLQSCV---------------------T-AQKKN----------SKN------LQMYFKR

LHNQVLKRMAYSAHAWELVRKEVRTHLMRL-VLLGSA--TENS-------I-----

>Eslu_2

--------------------------------------------------M-------HS

ASSLGFLDIMGE----DIPKD----------S--VK----IFFPEDLYKQADC------S

PADD--QIWF-----I-L----QTLDEITKLFS-DKC-YSV-WGEKTVDNFLGVLSSQVD

GLQSCI---------------------T-SQKKR----------SKN------LHKYFKR

LNNDILKSMEYSPHAWEMVRKEVRTHLKRL-TLLGSA--PDNK-------LVQQ--

>IFN_SPAU

--------------M--LNRIFF-VC---LSL----SLYSAG-SSLSCRWMDHKF-RQHS

KNSLALLDTMAN----NSTNT----------TEDAEVEDTVAFPNLLYRQASK------A

SAED--QLAF-----T-V----QILDETAALFE-EDH-SSASWEENTVENFVNVVTQQAD

ELRSCI---------------------G-SHKKK----------NKK------LHMYFQR

LSSHVLKRMGHSAEAWELIRGEVKVHLMRA-NQLVTSATRTN--------------

>IFN_DILA

--------------M--LNRIFF-VC---LSL----SLYSAG-SSLSCRWMDHKF-RQHS

KNSLALLDTMAN----NSTNT----------TEDAEVEDTVAFPNLLYRQASK------A

SAED--QLAF-----T-V----QILDETAALFE-EDH-SSASWEENTVEDFVNVVTQQAD

NLRSCI---------------------G-SHKT-----------NKK------LQMYFMK

LSSHVLKKMGHSAEAWELIRKEIKTHLMRA-DQLVSSLLTTN--------------

>IFN1_OPFA

--------------M--LSRTFL-VC---LFL----SLCSAG-SSLSCRWLDHKF-RQHS

ENSLDLLDTMVN----NSTNT----------TEDAGVKDTVAFPNELYSQASK------A

AAED--KLGF-----T-V----QVLEETAALFE-EDH-SSASWEENTVENFVNVVTQQAD

GLRSCI---------------------G-SHKKK----------NKK------LHMYFKR

LSSHVLEQMGHSAEAWELIRNEIQTHLMRA-DLLVSSLLTTN--------------

>IFN_PAOL

--------------M--LNRIFF-VC---LCL----CLYSAG-SAMSCRWMDHKF-RQYS

KNSLDLLDMMAH----NSINT----------TEDVEVEETVALPEHLYSQVSN------A

SAED--RLGF-----T-V----QVLKEVAALFE-EDS-SFASWEEKQMDDFLNIVTQQAD

GLRSCI---------------------V-SHSHK----------NKK------LHMYFKR

LSRHVLKQMDYSVESWELIRKEIKNHLMRS-DVLISSLLTIN--------------

>IFN2_ORNI

--------------M--MNRILF-AC---LFL----GLFTVG-SSLSCKWMDDKF-KQHN

EETLNLLDTMGN----NSTNT----------T---EVEDTVAFPNHLYRQASK------A

SAED--KLAF-----T-V----QVLEEVAALFE-EDH-SSASWEDSTVRNFLNIVNKQAE

ELHSCI---------------------G-SHSHK----------KKK------TEMYFKR

LSDDVLKKKGHSAEAWEVIRKETKAHLMRL--TLIKSRGTTQS-------L-----

>IFN_ORLA

--------------M--LHRLVF-AC---ALV----SLAGAG-FSLRCRWLDHKF-KQFS

DTSLDLLEKMVN----NATNS----------TEGDATEDIVDFPHHLYRQASK------E

SAEN--QVAF-----T-V----QVLKEVSALFE-EDS-SSASWQQITVEKFLGVVNRQAD

ELHSCV-----------------------SESKK----------NRK------LRMYFKR

LLDHILKKQGYSAEAWETIRKETKAHLLRA-QRLLSPLISSK--------------

>IFN1_ORNI

--------------M--ISRIFI-AC---LFL----GMYSTG-SSLSCKWI-----VKHP

GNTLALLCIMFN----VITNT----------TKDAEIEHNVAFPNRLYRQTSK------A

TAED--KLAF-----T-V----QILKELLALFE-EDH-SSASWEENTVENFLNIVDKQTE

ELHSCI---------------------G-SHSNT----------QKR------REKYFKR

LLNKILKKNGYSAEAWEKIRNITQAHLRQC--EFLISLRTAH--------------

>IFN_TARU

--------------M--LP--LL-VC---LSL----CVYSQG-SPLGCRWLDDKF-RQYS

HKSLELLDTMVN----NSTNS----------S--VEPEEMVIFPQELYRQTFN------A

SAED--KLAL-----A-A----QIMNETVALLM-EDH-SGASWDEKQVENLVNVLTQQAD

NLQACM---------------------V-SPGHK----------SEE------VERYFNR

LSNHILKKMDYSAAAWELIREEIETLLMQT-HLLVSTLLSTP--------------

>IFN_TENI

--------------M--LT--VL-LC---LSL----CVCSQG-SPLGCRWLEEKF-TQYS

SLSLSLLDNMKS----NSTNS----------S--LEAEDTAIFPEVLYRQTFN------A

SAED--RLAF-----A-A----QILNETAALFE-EDY-SGASWEEKSVENFVNILTQQAD

NLGSCV---------------------A-SPGQS----------SKE------LHKYFTR

ISTHILRKTDHSAGAWELVREKIRSLLMRA-HLL----LTTH--------------

>IFNa1_gaac

--------------M--TSWTSM-LV---LLT----LLCSAGTPGLCCDWL-QHY-GHLS

NVSLTLVQTMGN----QLTDE----------E--SP----VSFPYRLYERIMN------D

KEDN--QLVF-----I-R----DSLELMAKLYR-HDNRSSVTWDANKMERFLMIIHRQIH

GLNLCV---------------------S-T--Q----------ITRR------LRRYYRR

LEKKTLYSTGGSPASWELIRKESKLHLDQL-NQLWGFMV-----------------

>New|AVD96638.1_type_1_IFN_h_Argyrosomus_regius

--------------M--VNWTG-------VLF----VLCALLTPALFCDWL-RYY-GQLS

NNSLTLLQQMGG----QFTEQ----------E--CP----VRFPTRIYRDIYK------A

EVQS--QLVF-----I-R----DSLNLISGLYR-HDNLTSVSWDTVKTEHFLINIHRQTE

ELNTCV---------------------S-T--NT---------SNSS------LTKYYRR

LAKSTLHRTGGSPASWELIRKQTKLHLDQL-DLLVECIKSSSAACRRRSAASRQH-

>New|API68650.1_IFN_h_Larimichthys_crocea

--------------M--VNWTG-------VLF----VLCTLLTPALCCDWL-RHY-GHLS

NDSLTLLQQMGG----QWTGQ----------E--CR----VPFPRKIYRDIYK------A

EVQS--QLVF-----I-R----DSLKLISGLYH-HDNLTSASWDTVKTEHFLISIHRQTE

ELNTCV---------------------L-A--NT---------SNSS------LRKYYRR

LARSTLHCTGGSPASWELIRKQTKLHLDQL-DLLVECIKSSSAACRRRSAASRQH-

>New|XP_018516925.1_IFN_a3-like_Lates_calcarifer

MSAHIFIFITF--IM--ISST--------VLF----LLCAALAPALCCDWL-THY-SRHS

NISRTLILQMGD----PLTTE----------E--SK----VSFPYRLYKDIRN------T

EVES--QLVF-----I-R----DSLELILNLYH-HDNHSSIAWDTDKMDHFLMSVDRQID

GLNTCV---------------------S-M--NR---------ADSR------LRKYYRR

LEKRTLYLTGGAPASWELIRKETKLHLDQL-ELLVASIR--AATRRRRSTPTHQH-

>New|XP_026180879.1_IFN_a3-like_Mastacembelus_armatus

--------------M--MSCIR-------LFF----ILCSVLTPALCCDW--RHY-RRYS

NNSLTFIRLMGG----PLTKE----------K--SP----VPFPEKLNKLIRN------A

EVET--KLVF-----I-R----DNLKEIAHLYH-HGNRSSVTWDTHTTTKFLTNIDRQIE

ELNTCV---------------------S-T--NT---------ADHR------LRKYYKR

LVTRILGHTGGSTSSWELLRKETKLRLDHL-DLLVAFIKDAASASRSRSTPTQQH-

>New|BBA46272.1_typeI_IFN_4_Paralichthys_olivaceus

------------------------MI---RCT----IILCALTSALCCDWL-RHY-NHYS

NVTLTLLRQMGG----QLTED----------E--SP----VSFPFKLYTQVRH------R

KVES--QLIF-----I-K----NSLLGISDLYR-HDNLSSPTWDTKVTHHFQVNLHRLKE

ELNTCV---------------------P-TSKQ----------LNRR------LTRYYTR

L-RRTLDRTGGSIASWELIRKETELHLHQL-QLLVTSILNSAASRRR---------

>New|XP_019966742.1_IFN_a3-like_Paralichthys_olivaceus

STEHVFVFI----II--IFFIFI-MI---RCT----IILCALTSALCCDWL-RHY-NHYS

NVTLTLLRQMGG----QLTED----------E--SP----VSFPFKLYTQVRH------R

KVES--QLIF-----I-K----NSLLGISDLYR-HDNLSSPTWDTKVTHHFQVNLHRLKE

ELNTCV---------------------P-TSKQ----------LNRR------LTRYYTR

L-RRTLDRTGGSIASWELIRKETELHLHQL-QLLVTSILNSAASRRR---------

>New|XP_026019408.1_IFN_a3-like_Astatotilapia_calliptera

--------------M--VTWTG-------LLF----VLCGALTPALCCDWL-THY-KQPS

KEARGLLTLMGD----QLTEQ----------H--SP----VRFPKHLYKQIKN------S

EVDS--KLVF-----I-R----DSLQLIFCLYR-HDNLSAAPWGAGKTEGFLTVIHRQIM

ELSACV---------------------S-T--NP---------ANSR------LRSYYRT

LANSTLSCSGCSTASWQLLRKETKLRLEQL-ELLVASIRVPAAASRRRSAATQQRN

>New|XP_005950669.1_IFN_a3-like_Haplochromis_burtoni

--------------M--VTWTG-------LLF----VLCGALTPALCCDWL-THY-KQPS

KEARGLLTLMGD----QLTEQ----------H--SP----VRFPKHLYKQIKN------S

EVDS--KLVF-----I-R----DSLQLIFCLYR-HDNLSAAPWGADKTEGFLTVIHRQIM

ELSACV---------------------S-T--NP---------ANSR------LRSYYRT

LANSTLSCSGCSTASWQLLRKETKLRLEQL-ELLVASIRVPAAANRRRSAATQQRN

>New|XP_014265825.1_IFN_a3_Maylandia_zebra

--------------M--VTW-G-------LLF----VLCGALTPALCCDWL-THY-KQPS

KEARGLLTLMGD----QLTEQ----------H--SP----VRFPKHLYKQIKN------S

EVDS--KLVF-----I-R----DSLQLIFCLYR-HDNLSAAPWGADKTEGFLTVIHRQIM

ELSACV---------------------S-T--NP---------ANSR------LRSYYRT

LANSTLSCSGCSTASWQLLRKETKLRLEQL-ELLVASIRVPAAASRRRSAATQQRN

>New|XP_024658104.1_IFN_a3-like_Maylandia_zebra

--------------M--VTWTG-------LLF----ILCGTLTSALCCDWL-THY-KAPM

EEARGFLTRMGD----QLTEQ----------H--SP----VRFPKHLYKQIKN------S

EVDS--KLVF-----I-R----DSLQLIFCLYR-HDNLSAAPWGADKTEGFLTVIHRQIM

ELSACV---------------------S-T--NP---------ANSR------LRSYYRT

LANSTLSCSGCSTASWQLLRKETKLRLEQL-ELLVASIRVPAAASRRRSAATQQRN

>New|XP_013771349.1_IFN_a3-like_Pundamilia_nyererei

EAPHS-IFIIF--IM--VTWTG-------LLF----ILCGTLTSALCCDWL-THY-KAPM

EEARGFLTRMGD----QLTEQ----------H--SP----VRFPKHLYKQIKN------S

EVDS--KLVF-----I-R----DSLQLIFCLYR-HDNLSAAPWGADKTEGFLTVIHRQIM

ELSACV---------------------S-T--NP---------ANSR------LRSYYRT

LANSTLSCSGCSTASWQLLRKETKLRLEQL-ELLVASIRVPAAASRRRSAATQQRN

>New|XP_005469255.2_IFN_a3_Oreochromis_niloticus

--------------M--VTWTG-------LLF----VLCGALTSALCCDWL-THY-KQPS

KEARDLLTLMGD----QLTEQ----------H--PP----VRFPKHLYKQMRN------S

EVES--KLVF-----I-R----DSLQLIFCLYR-HDNLSAAPWGADKTEGFLTVIHRQIT

ELSACV---------------------S-T--NP---------ANSR------LRSYYRT

LANSTLSCSGCSTASWQLLRKETKLRLDQL-ELLVASIRVPASASRRRSAATQQRD

>New|XP_020496686.1_IFN_a3-like_Labrus_bergylta

--------------M--NSMSRL-TC---LLF----VLCSVLTPTLGCDWISRRF-GHLS

GLSLDLIQHMGG----PLTKQ----------P--SP----VRFPDRLYSRVRK------A

EVES--QLVF-----I-R----DSLDLISGLYH-HDNLTSVTWNTKKLEDFQAIIHRQAE

ELSRCV---------------------S-STTN----------YKRQ------LRNYYRR

L-SCTLNRTGGSAASWELIRKETKLNLDHL-DVLVSFIKASGAASRRRNTPTHQHQ

>Anja_7

---------V--------------------------------------------------

-------------------EE---------------------------------------

------RIIF-----I-H----EVINNIKDLYIKGKY-DTVTWDPKKLQMFQLNLHRQAS

ELKEC------------------------------------------------VRPNFAP

PQN-AGHDKEYR--------------------------------------------

>IFN4_DARE

--------------M--KV--FA-AA---QFC----VLLSVG-FSLGCRWVKHRL-QHHH

GVSLDLLRKMGE----KVHDD----------N-----EDLNPIPYDLINNHRM------A

EPEK--QIQF-----V-I----QALVEITALFD-DAL---VPWDAKKMDDFLNIMHEEID

GLRSCG---------------------S-YKMKR----------NKK------LHLYFNR

-----LRRMTDGGRSWEMVRKRVIS-LMNQ-LHSFSF--HTH--------V-----

>IFNF2_ONMY

--------MATLN----VS--FV-VH---LLC----VIV------LKCSDQKEQM-YNLS

QTRQTLNDLAMERRPRGCIPEAEMIMVQ--R-------------PTLS----K------E

EVEKVWTLRL-------------AFQLASELFQ-QNL-TLVKWNSIKLRDLQDLLARQ--

----------------------YMTVRD-MRLRQ----------NLP------IKNYFKQ

LDD-FLSRESFSLCSWEVVRTEMGSIL-------RDF--YKKS-------KMRKHV

>IFNF1_ONMY

--------MATLN----VS--FV-VH---LLC----IIV------FKCSAPKVQK-YYLS

QTHQTLNDLAEERLPRGCIPEAERLRVQ--R-------------PALP----I------E

EGEKVWTLRL-------------AFQLASELFQ-QNL-TLVKWNSVKLRDLQDLLARQ--

----------------------NMTVRD-MSVHL----------NLP------IKNYFKE

LED-FLSHERFSACSWELVRAEMGSII-------SQA--IRNA-------K--KHV

>eslu_f

--------MAAHN----VT--FV-VH---LLF----ALA------LTCCD---ET-YYIF

KTRQVVNDLAMGRKPV-CVQEAARIRVH--R-------------PTLS----L------E

VGERFWTLSL-------------VFHLACELFQ-RNL-TLVKWNVNQLRELQELLARQ--

----------------------NRTVKD-IRLGQ----------SLP------NKKYFKQ

LDD-FLSRETFSLCAWEVVRFEMGRIL-------RDF--HKKS-------N-SKKT

>Eslu_4

--------------MYSIR--LC-MS---LVL----MICSCN-ETMGCTWMRSMF-KSFI

SKSITVLQEKD-------DGE-------------PL----ISLPNKLYRQFDD------L

KADD--QIVF-----I-S----RTLKAIMHLYSSGKY-ES-TLETERIDTFIHYLSRQTM

ELDQCI---------------------K---AMNPTLSKSVKRANKK------MNSHFKF

LKN-YLKGEEFNGKAWIEIKRVVLAHLRRI-V--LT--------------------

>Opha_4

MAATSMGLLCL------------------VLLLAVPA-LG-----LHCNLLKWQQ-QRLN

QQSVELLKGMK---SPVCL--RKEVTP-QIL---------------------R------I

QRPRA-AKAI-----L------EMLHGFLHLFK-EDH---VAWDATLRKRFLPSFSAPV-

----CI----------------SI------------------------------------

---------------------QVVLDLQWF-T------------------------

>Thsi_4

MPSKRMGLLCL------------------VLLLAAPT-WG-----SNCNLLKLHQ-RRLN

RQSVELLRRVK---PAECL--RKVFSP-KIL---------------------G------I

REPRE-AKAV-----L------EVLQGFLHILK-DEH---VAWKATLQKRFLPMLHAQIQ

RIQGCL----------------GE-GRKEEKL--------------Q------LKKYFRS

IGN-FLEENGLDSCTREFVRHEIQLDFIYL-DRLTER--ME---------------

>Pybi_6

MSATPTELLCL------------------VLLLSAQV-TA-----LNCNFLKLQQ-QRFN

RHSVELLKGMS---PQECL--RKTSNP-TVL---------------------E------I

HQPQI-AKTI-----L------EMLHGFLNILS-DDC---NAWEAKLRNR----------

---KCV----------------SA------------------------------------

--------------TVQFPLTELRCPFTYF-QVEAEE--ND---------------

>IFN1_frog

--------MS--------------VS---VLL----LIT------LGSSGQPTKG-KDVY

RTQLNIN------------REVRTLLGN--M-------------GAIP----Y------S

ECEDNWRLQI-------------TIHQFSVIFT-DNL-A----NSVEMSKMQTLLYWY--

----------------------STSLKE-LTLKE----------TKK------IRRYFRK

MLK-YLMKKGYSRCAWASVRDEMEKVLLLV-TWHTDI--LLKK-------HLRGHV

>New|ANQ43256.1_type_I_IFN_1_Xenopus_tropicalis

--------MS--------------VS---VLL----LIT------LGSSGQPTKG-KDVY

RTQLNIN------------REVRTLLGN--M-------------GAIP----Y------S

ECEDNWRLQI-------------TIHQFSVIFT-DNL-A----NSVEMSKMQTLLYWY--

----------------------STSLKE-LTLKE----------TKK------IRRYFRK

MLK-YLMKKGYSRCAWASVRDEMEKVLLLV-TWHTDI--LLKK-------HLRGHP

>New|ANQ43257.1_type_I_IFN_2_Xenopus_tropicalis

MGSWVLQTLS--------------LC---VLF----LTI------SIPRGQSLEC-NHIY

MSQHHCN------------KEALKHLVN--M-------------QKML----H------P

NCKDQWKLLE-------------IVHESSKLFS-SQL-V----MSTKISQLLMVLHKS--

----------------------STDWAQ-CVASK----------IRQ------IKKYFGR

MEA-YLKKKGYSHCALAAVINEVENLMKFV-ARHTDI--LLKK-------DL----

>New|ANQ43306.1_type_I_IFN_1_Xenopus_laevis

M-----QTCR--------------GC---ILF----LMC------TIPRFR---------

--------------------------------------------ELIL----H------P

HCKEQWSLLE-------------IVHESSKLFG-SQL-T----KTTKLSDLLMVLHKI--

----------------------STDWTQ-CVMSK----------TRQ------IKKYFGR

MEA-FLKKKDYSHCALATVTNEMESAMMFV-NRHTDI--LWKR-------EK----

>IFN4_cami

--------MSLP-----------------VLLLCSPT-SGHG---SDC--M---Q-RRYW

RDLLESLN------PVACRGE--DVRE-PVML--------------------N------P

VKKD--KAAV-----T-L----QVLEEILRLFR-K---STVPWTNSK---FLNQVYQVIY

ELQNCM------TS--------S-----DLPVRS------------T------IKARFAN

LEG-FLNEKSI--CAWEIVHLETRKILQ----EVSRN--HARK-------------

>IFN5_cami

--------MSLP-----------------VLLLYSPT-SGHG---SDC--V---Q-RRYW

RDLLESLN------PEECRNE--DINP-APIS--------------------K------L

AKQN--KAAV-----I-V----QVLEEVLKLFS-K---PDAPWSNSKALTFLNKVSQILS

DLQSCM------QS--------P-----DSRVRS------------T------VIERFAK

LER-FLKAKSV--CAWEIVHAETRKIFQ----QVEQR--HARK-------------

>New|ANQ43329.1_type_I_IFN_16_Xenopus_laevis

MAPTH-SALSL------------------LLL-----------LTLHCAAISLHR-----

------------FYKAEC----------------------------------E-------

QS----A--VGSQ----HGTGLHLLQESPNVMR--------------L------------

----CI---------------CAEQDKDTTSLT----------LT--------CISFLIQ

ISQL----HRIKLIQYNPFYHNAGFSF----SALAQY--CGK--------------

>New|ANQ43320.1_type_I_IFN_8_Xenopus_laevis

MAPTH-SALTL------------------LLL-----------LTLHCAAISLHR-----

------------FYKAEC----------------------------------E-------

QS----A--VGSQ----HGTGLYLLQESPNVMR--------------L------------

----CI---------------CAEQGKDTTSLT----------LT--------CISFLIS

LSCINITRHRIKLIQSKPFYHNAGFSF----SALAQY--CGK--------------

>New|ANQ43318.1_type_I_IFN_6_Xenopus_laevis

MAPTH-SALSL------------------LLL-----------FTLHCAAISLHR-----

------------FYKAEC----------------------------------E-------

QS----A--VGSQ----HGTGLHLLQESPNVMR--------------L------------

----CI---------------CAEQGKDTTSHT----------NI--------PSSN-KS

LSFINVTRHRIKRIQYNPFYHNAGFSF----SALAQY--CGK--------------

>New|ANQ43327.1_type_I_IFN_14_Xenopus_laevis

MAPTH-SALSL------------------LLL-----------LTLHCAAISLHR-----

------------FYKAEC----------------------------------E-------

QS----A--VGSQ----HGTGLYLLQESPNVMR--------------L------------

----CI---------------CAEQDKDTTSLT----------LT--------FLPH-TN

LSVVFPKTSSISFVQ------DTGSS------------------------------

>New|ANQ43325.1_type_I_IFN_18_Xenopus_laevis

MAPTH-SALSL------------------LLL-----------FTLHCAAISLHR-----

------------FYKAEC----------------------------------E-------

QS----A--VGSQ----HGTGLYLLQESPNVMR--------------L------------

----CI---------------CAEQGKDTTSLT----------LT--------FLPH-TN

LSVVFLKTSSISFVQ------DTGSS------------------------------

>New|ANQ43323.1_type_I_IFN_11_Xenopus_laevis

------------------------------------------------------------

-----MLSQESA----------------------V-------------------------

EQEHT-ATAA------------EV------------------------ARLLNPIEQAID

KSNDSF------------------S--TKSKLE----------FENK---ILIFQDYSVK

LSQ---KRKEFSNSCQQLVSLGINFALQFV-DHLGNRILLSHKE------II----

1. **IFN1 Amphibian sister group to all other IFN1s:**

>ANQ43258.1_type_I_interferon_3_Xenopus_tropicalis

MFKRNTFGSYCCINYAIYVSITVTTLHM--ASIQ------------TILLLVL-------

---------IPIVQSQNCKWLQ---------------PKQEYLNRQTLKTFEEMNP---P

EDYDESCQYDSIELPNIDEI-YSISQ---------------------------MEEMVLA

VRGVLNETMRFYMKHHESMGCKQQ-AWERFQ-------QLLYYQINQLEACIPETAENPV

FNQTISDQYQALEQIL---------QEK--NTACT---RDIIQSE-IR-GNLQLVGQLAS

RARRQRLLQRTA------------------------------------------------

---------------------------------------------

>ANQ43307.1_type_I_interferon_2_Xenopus_laevis

------------------------MIHM--LSIK------------TFLGLVL-------

---------ISIVQPKTCKWLR---------------PKQEYLNSQILKTFEEMSP---F

EDYDETCQY--DELPNIDGI-YSISQTCAPPAAGRTSGQKLHWGPVLTIC-PKVEAAALA

VREVLNETIRFYRKHHESMGCKQQ-AWERFQ-------QLLYYQIHQLEGCIPETAANPV

FNQTLSEQFAVLEKFI---------QEE-ENTPCL---RDIIQSE-IR-RNLQLAAQLSS

RARRQHLLQGTA------------------------------------------------

---------------------------------------------

>ANQ43310.1_type_I_interferon_5_Xenopus_laevis

--------------------------------MK------------IITGQGLNRTRRFT

SSWENESYDVSTIIAVAQSYLHCP------------LPKLQMASPQTGISQHPNSPNLQP

IEFDIVCHDYPTAQPNLERL-YNITQ---------------------------VEAAALA

VREVLNETIRFYRKHHESMGCKQQ-AWERFQ-------QLLYYQIHQLEGCVPETGENDL

LKNKISEQFQQWEKNV---------AEQ------A---WS--------------------

------------------------------------------------------------

---------------------------------------------

>ANQ43259.1_type_I_interferon_4_Xenopus_tropicalis

------------------------MLPM--GQWS------------VLLLLSL-------

---------TSIVHSQSCKWLH---------------PKQEYLNTQILKAFNEMMP---L

KETEEICEEHPTDLPNTEST-YSVSQ---------------------------VEAGALA

VREVLNETMRFYMKHHESMGCKQQ-AWERFQ-------QLLYYQIHQLEACVSQTEENDL

LKESISEEFNLLETMV---------LEK-DNSACV---WDFIHLE-TR-RNLQQVLQLSS

RLRRQRLLQRPQ------------------------------------------------

---------------------------------------------

>ANQ43261.1_type_I_interferon_6_Xenopus_tropicalis

------------------------MLQM--GQWS------------VLLLLSL-------

---------TSIVHSQSCKWLH---------------PKQEYLNSQILKAFNEMIA---L

EKNDKICQENPTDPSNEHRI-YSVSQ---------------------------VEAGALA

VREVLNETMRFYMKHHESMGCKQQ-AWERFQ-------QLLYYQIHQLEACVSQTEENDL

LKESISEEFNLLETMV---------LEK-DNSACV---WDFIHSE-TR-RNLQQVLELSS

RLRRQRLLQRPQ------------------------------------------------

---------------------------------------------

>ANQ43262.1_type_I_interferon_7_Xenopus_tropicalis

------------------------MLQM--GQWS------------VLLLLSL-------

---------TSIVHSQSCKWLH---------------PKQEYLNTQILKAFHEMKC---L

EENVNICQENPSELPNKERI-YSVSQ---------------------------VEA---T

VKEVLNETMRFYMKHHESMGCKQQ-AWERFQ-------QLLYYQIHQLEACVSQTEENDL

LKESISEEFNLLETMV---------LEK-DNSACV---WDFIHSE-TR-RNLQQVLQLSS

RLRRQRLLQRPQ------------------------------------------------

---------------------------------------------

>ANW82729.1_type_I_interferon_5_Xenopus_tropicalis

------------------------MLQM--GQWS------------VLLLLSL-------

---------TSIVHSQSCKWLH---------------PKQEYLNSQILKAFNETFT---V

RETDEICQEHRSDLSNTESL-YNVSQ---------------------------VEAGVLI

VREVLNETMRFYMKHHESMGCKQQ-AWERFQ-------QLLYYQIHQLEACIPKASENHL

IKETISEEFNLLEKIV---------LGK-DNSACV---WDFIHSE-TR-RNLQQVLQLSS

RLRRQRLLQRPQ------------------------------------------------

---------------------------------------------

>ANQ43260.1_type_I_interferon_5_Xenopus_tropicalis

------------------------MLQM--GQWS------------VLLLLSL-------

---------TSIVHSQSCKWLH---------------PKQEYLNSQILKAFNETFT---V

RETDEICQEHRSDLSNTESL-YNVSQ---------------------------VEAGVLI

VREVLNETMRFYMKHHESMGCKQQ-AWERFQ-------QLLYYQIHQLEACIPKASENHL

IKETISEEFNLLEKIV---------LGK-RNAEIR---CKSMPGEKVC-SSLMSIFMIRV

GLGRRDTGKNPDGPQ--------------------------------------P------

---------------------------------------------

>ANQ43311.1_type_I_interferon_6_Xenopus_laevis

------------------------MFHM--GQWS------------VLLLLSL-------

---------TSIVHSQSCKWLH---------------PKQEYLNTQILKTFNQTIS---L

RENDEICEEHPSEFPNTESI-YNVSQ---------------------------VEAAALT

VREVLNGTIRFYMKHHESMGCKNQ-AWERFQ-------QLLYYQIHQLEGCIPETAENHL

IKGAVFEEFNLLEKIV---------LEKIFGVSIV---YIF---------NVV-------

------------------------------------------------------------

---------------------------------------------

>ANQ43312.1_type_I_interferon_7_Xenopus_laevis

------------------------MFHM--GQWS------------VLLLLSL-------

---------TSIVHSQSCKWLH---------------PKQEYLNTQILKTFNETIP---I

RETEKICEEHPSDLPNTESI-YNVSQ---------------------------VEAAALA

VREVLNGTIRFYMKHHERMGCKQQ-AWERFQ-------HLLYYQIHQLEGCISETAEDHL

IKESVSEQFNLLEKTI---------LEK-GSSACV---WDFIHSE-IR-RNLQLVLQLSS

RLRRHHLIQRTQ------------------------------------------------

---------------------------------------------

>ANQ43309.1_type_I_interferon_4_Xenopus_laevis

------------------------MCHM--GQWS------------VLLLLSL-------

---------TSIVHSQSCKWLH---------------PQQEYLNSQILKAFNQTIP---I

RETEKVCEEQPSHLPNTESI-YNVSQS-------------------FCVCLEEVEAAALA

VREVLNETIRFYRKHHESMGCKQQ-AWERFQ-------QLLYYQIHQLEGCIPETAENHL

IKESVSEHLNLLEKIV---------LEK-DNSACV---WDFIRSE-IR-RNLQLVLQLSS

RLRRQHLLQRTQ------------------------------------------------

---------------------------------------------

>ANQ43308.1_type_I_interferon_3_Xenopus_laevis

------------------------MCHM--GQWS------------VLLLLSL-------

---------TSIVHSQSCKWLH---------------PQQEYLNSQILKAYNQMNN---L

EESDKTCQEYPTELPYEERI-KNVLQ---------------------------VETAALA

VREVLNETIRFYRKHHESMGCKQQ-AWERFQ-------QLLYYQIHQLEGCIPEMAENHL

IKEAISEEFHLLEKMV---------LEK-DNSACV---WVFISSE-IR-RNLQLVLQLSS

RFRRQHLLQRTQ------------------------------------------------

---------------------------------------------

>ANQ43324.1_type_I_interferon_12_Xenopus_laevis

------------------------MAPA---KFQ-LTKLFMVLTM-TILQQVG-------

---------PSSASSPECQWLD---------------KKGEHIENEILIVLDQLQP---K

EEIPGDCF---DEVPHYGFP-NNIHE---------------------------LKAAAMV

VHTVYNETVIFYRNFAKTLGLPEK-DYEKLL-------SLLRYVMNNLTPCVTNAEQYKN

VTERTSNQYIEFENYV---------QKW-GNTACA---QIIFWLI-S--KNVQEAVHLSS

QMRKIDLMNKTS------------------------------------------------

---------------------------------------------

>ANQ43322.1_type_I_interferon_10_Xenopus_laevis

------------------------MAPA---KFQ-LTKLFVVLAM-TILQQVR-------

---------PSSASSPECQWLD---------------KKGEHIENEILIVLDQLQP---K

EEIQDDCF---DEVPHYGFP-NNI------------------------------------

-----------------VLSSLQK-DYEKLL-------SLLRYVMNNLTPCVTNAEQYKN

VTERTSNQYTEFENYV---------QKW-GNTACA---QIIFWLI-S--KNVQEAVHLSS

QMRKINLMNKTS------------------------------------------------

---------------------------------------------

>ANQ43317.1_type_I_interferon_5_Xenopus_laevis

------------------------MAPA---KFQ-LTKLFVVL---VILQQVR-------

---------PSNSRSPECQWLD---------------KKGEHIDNEILIVLDHLQP---K

EKIQDDCF---DEVHHYGFP-NNIHE---------------------------LKAAAMV

VHTVYNETVIFYSNFAKTLGLSKK-DYEKLL-------SLLRYVMNNLTPCVTNAEQYKD

VTERTSNQYTELENYA---------QKW-GNTACA---QIIIWII-S--NNVQEAVHLSS

QMRKMLLMSKTS------------------------------------------------

---------------------------------------------

>ANQ43316.1_type_I_interferon_4_Xenopus_laevis

------------------------MAPA---KFQ-LTKLFVVL---AILQQVR-------

---------PSSASSPECQWLD---------------KKGEHIDNEILMVLDHLQP---K

EEIQDDCF---DEVSHYGFP-NNIHE---------------------------LKAAAMV

VHTVYNETVIFYSNFAKTLGLSEN-DYEKLL-------SLLRYVMNNLTPCVTNAEQYKN

VTEPISNQYTELENYA---------QKW-GNTACA---QIIIWVI-S--NNVQEAVHLSS

QMRKMHLMSKTS------------------------------------------------

---------------------------------------------

>ANQ43313.1_type_I_interferon_1_Xenopus_laevis

------------------------MAPA---KFQ-LTKLFVVL---AILQQVR-------

---------PSNSSSPEY------------------------------------------

-----DCF---DEVSHYGFPNNNIHE---------------------------LKAAAMV

VHTVYKETMIFCSNFVKTLGLSEN-DYEKLL-------SLLRYVMNNLTPCVTNAERYKD

VTESISNQYTELENNA---------HNL-GN-------------------NVQEAVHLSS

QMQKMNLISKTS------------------------------------------------

---------------------------------------------

>ANQ43332.1_type_I_interferon_20_Xenopus_laevis

------------------------MASA---KIH-LAKLSVVLAMAILFLVVQ-------

---------PSRFSSPECQWLQ---------------KKGEHIDNKILIPHEQCQP---K

VENRDHCF----DIPDNGFP-NNIHE---------------------------LKAAVMV

VHTVYNETMIFYSNYGKTLGLLEN-DYDYLR-------SLLEDVINELAQCVTNAGQYKD

FTDPISNQYRELEKHV---------QKW-GNSACA---QNIFWVI-S--ENLEKAVPLSS

QMRKMNLMNKTS------------------------------------------------

---------------------------------------------

>AWK27013.1_IFN_2.4_Xenopus_tropicalis

------------------------MTPG---EINCLAPLAVATT--ILYLLVR-------

---------PSSASSPECRWPD----------------RSEFIDNEILKAFDNLQP---K

EQHQDYCL---FEIPDSDFI-HNRSR---------------------------GEAAA-I

VFAVYNETLSFYKKK----------VPRKLL-------KLLLRRLKQLGRCVTGTAQYKA

VTDAICKEYRELRQKA---------REW-GNTTCA---RSIFWAL-G--NKLQQAVQLSS

LMTKMYLMSKTS------------------------------------------------

---------------------------------------------

>ANQ43275.1_type_I_interferon_13_Xenopus_tropicalis

------------------------MTPG---EINCLAPLAVATT--ILYLLVR-------

---------PSSASSPECRWPD----------------RSEFIDNEILKAFDNLQP---K

EQHQDYCL---FEIPDSDFI-HNRSR---------------------------GEAAA-I

VFAVYNETLSFYKKK----------VPRKLL-------KLLLRRLKQLGRCVTGTAQYKA

VTDAICKEYRELRQKA---------REW-GNTTCA---RSIFWAL-G--NKLQQAVQLSS

LMTKMYLMSKTS------------------------------------------------

---------------------------------------------

>ANQ43288.1_type_I_interferon_26_Xenopus_tropicalis

------------------------MAPG---EINCLALLAMATT--ILYLLVR-------

---------PSSASSPECRWPD----------------RSEFIDNEILKAFDNLQP---K

EQHQDYCL---FEIPDSDFI-HNRSR---------------------------GEAAA-I

VFTVYNETLSFYKKK----------VPRKLL-------KLLLRRLKQLGRCVTGTAQYKA

VTDAICKEYRELRQKA---------REW-GNTTCA---RSIFWAL-G--NKLQQAVQLSS

LMTKMYLMSKTS------------------------------------------------

---------------------------------------------

>AWK27012.1_IFN_2.3_Xenopus_tropicalis

------------------------MAPG---ERNCLAPLAVATT--ILYLLVR-------

---------PSSASSPECRWPD----------------RSEFIDNEILKVFDNLQP---K

EQHQDYCL---FEIPDSGFI-HNRSR---------------------------GEAAA-I

VFAVYNETLSFYKKK----------VPRKLL-------KLLLRRLKQLGRCVTGTAQYKA

VTDAICKEYRELRQKA---------REW-GNTTCA---RSIFWAL-G--NKLQQAVQLSS

LMRKMYLMSKTS------------------------------------------------

---------------------------------------------

>ANQ43276.1_type_I_interferon_14_Xenopus_tropicalis

------------------------MAPG---ERNCLAPLAVATT--ILYLLVR-------

---------PSSASSPECRWPD----------------RSEFIDNEILKVFDNLQP---K

EQHQDYCL---FEIPDSGFI-HNRSR---------------------------GEAAA-I

VFAVYNETLSFYKKK----------VPRKLL-------KLLLRRLKQLGRCVTGTAQYKA

VTDAICKEYRELRQKA---------REW-GNTTCA---RSIFWAL-G--NKLQQAVQLSS

LMRKMYLMSKTS------------------------------------------------

---------------------------------------------

>ANQ43287.1_type_I_interferon_25,_partial_Xenopus_tropicalis

------------------------------------------------------------

------------------------------------------------------------

-----------------------RSR---------------------------GEAAA-I

VFAVYNETLSFYKKK----------VPRKLL-------KLLLRRLKQLGRCVTGTAQYKA

VTDAICKEYRELRQKA---------REW-GNTTCA---RSIFWAL-G--NKLQQAVQLSS

LMRKMYLMSKTS------------------------------------------------

---------------------------------------------

>ANQ43294.1_type_I_interferon_32_Xenopus_tropicalis

------------------------MAPG---EINCLAPLAVATT--ILYLLVR-------

---------PLSATSPECRWPD----------------RSEFIDNEILKAFDNLQP---K

EQHQDDCL---FEIPDSDFI-HNRSR---------------------------GEAAA-I

VFAVYNETLSFYKKK----------GPRKLL-------KLLLRRLKQLGRCVTGTAQYTA

VTDAICKEYRELRQKA---------REW-GNTTCA---RSIFWAL-G--NKLQQAVQLSS

LMRKMYLMSKTS------------------------------------------------

---------------------------------------------

>ANQ43293.1_type_I_interferon_31_Xenopus_tropicalis

------------------------MAPG---EINCLAPLAVATT--ILYLLVR-------

---------PSSASSPECRWPD----------------RSEFIDNEILKAFDNLQP---K

EQHQDDCL---FEIPDSGFI-HNRSR---------------------------GEAAA-I

VFTVYKETLSFYKKK----------GPRKLL-------KLLLRRLKQLGRWVTGTAQYTA

VTDAICKEYRELRQKA---------REW-GNTTCA---RSIFWAL-G--NKLQQAVQLSS

LMRKM---SHE-------------------------------------------------

---------------------------------------------

>ANQ43289.1_type_I_interferon_27_Xenopus_tropicalis

------------------------MAPG---EINCLAPLAVATT--ILYLLVR-------

---------PSSASSPECRWPD----------------RSEFIDNEILKVFDNLQP---K

EQHQDDCL---FEIPDSGFI-HNRSR---------------------------GEAAA-I

VFAVYNETLSFYKKK----------GPRKLL-------KLLLRRLKQLGRCVTGTAQYTA

VTDAICKEYRELRQKA---------REW-GNTTCA---RSIFWAL-G--NKLQQAVQLSS

LMRKMYLMSKTS------------------------------------------------

---------------------------------------------

>AWK27011.1_IFN_2.2_Xenopus_tropicalis

------------------------MAPG---EINCLAPLAVATT--ILYLLVQ-------

---------PSSASSPECRWPD----------------RSEFIDNEILKAFDNLQP---K

EQHQDDCL---FEIPDSDFI-HNRSR---------------------------GEAAA-I

VFAVYNETLSFYKKK----------GPRKLL-------KLLLRRLKQLGRCVTGTAQYKA

VTDAICKEYRELRQKA---------REW-GNTTCA---RSIFWAL-G--NKLQQAVQLSS

LMTKMYLMSKTS------------------------------------------------

---------------------------------------------

>ANQ43281.1_type_I_interferon_19_Xenopus_tropicalis

------------------------MAPG---EINCLAPLAVATT--ILYLLVQ-------

---------PSSASSPECRWPD----------------RSEFIDNEILKAFDNLQP---K

EQHQDDCL---FEIPDSDFI-HNRSR---------------------------GEAAA-I

VFAVYNETLSFYKKK----------GPRKLL-------KLLLRRLKQLGRCVTGTAQYKA

VTDAICKEYRELRQKA---------REW-GNTTCA---RSIFWAL-G--NKLQQAVQLSS

LMTKMYLMSKTS------------------------------------------------

---------------------------------------------

>ANW82731.1_type_I_interferon_2.1_Xenopus_tropicalis

------------------------MAPG---EMNCLAPLAVATT--ILYLLVQ-------

---------PSSASSPECRWPD---------------KKGEFIDNEILKVFDNLQP---K

EQHQDDCL---FEIPDSDFI-HNRSR---------------------------GEAAA-I

VFAVYNETLSFYKKK----------GPRKLL-------KLLLRRLKQLGRCVTGTAQYKA

VTDAICKEYRELRQKA---------REW-GNTTCA---RSIFWAL-G--NKLQQAVQLSS

LMTKMYLMSKTS------------------------------------------------

---------------------------------------------

>ANQ43282.1_type_I_interferon_20_Xenopus_tropicalis

------------------------MAPG---EMNCLAPLAMVTT--ILYLLVQ-------

---------PSSASSPECRWPD---------------KKGEFIDNEILKVFDNLQP---K

EQHQDDCL---FEIPDSDFI-HNRSR---------------------------GEAAA-I

VFAVYNETLSFYKKK----------GPRKLL-------KLLLRRLKQLGRCVTGTAQYKA

VTDAICKEYRELRQKA---------REW-GNTTCA---RSIFWAL-G--NKLQQAVQLSS

LMRKMYLMSKTS------------------------------------------------

---------------------------------------------

>ANQ43298.1_type_I_interferon_36_Xenopus_tropicalis

------------------------MAPG---EINCLASLAVATT--ILYLLVQ-------

---------PSR----------------------------ETIDNEILKVFDNLQL---Y

KEIEDDCH---FEVHDTGFI-NVSSP---------------------------GEAAA-M

VLTIFWETKNFYQKKTQCFRLPEE-DRNWLL-------YLLTEEIDQLAPCVTDKAQYKA

VTEPISREYRELLKKA---------WEW-GNTACT---ETVNWAV-R--NIVQQGVTVSS

QRRKLDLIRNIS------------------------------------------------

---------------------------------------------

>ANQ43296.1_type_I_interferon_34_Xenopus_tropicalis

------------------------------------------MT--ILYLLVQ-------

---------PSR----------------------------ETIDNEILKVFDNLQL---Y

KEIEDDCH---FEVHDIGFI-NVSSP---------------------------GEAAA-M

VLTIFWETKNFYQKKTQCFRLPEE-DRNWLL-------YLLTEEIDQLAPCVTDKAQYKA

VTEPISREYRELLKKA---------WEW-GNTACT---ETVNWAV-R--NIVLQGGTVSL

QRRKLDLIRNISKRCLYSYCKTNTFMKRNSVTIA--------------------------

---------------------------------------------

>ANQ43290.1_type_I_interferon_28,_partial_Xenopus_tropicalis

------------------------------------------------------------

------------------------------------------------------------

------------------------------------------------------------

-----------------------E-DRNWLL-------YLLTEEIDQLAPCVTDKAQYKA

VTEPISREYRELLKKA---------WEW-GNTACT---ETVNWAV-R--NIVQQGVTVSS

QRRKLDLIRNIS------------------------------------------------

---------------------------------------------

>ANQ43268.1_type_I_interferon_6_Xenopus_tropicalis

------------------------MAPG---EINCLAPLAVATT--ILYLLVR-------

---------PSSATSQECRWPD---------------KKGENIDNEILKVFDNLQL---Y

KEIEDDCH---FEVHDIGLI-NVSSP---------------------------GEAAA-M

VLTIFRETKNFYQKKTQRFRLPEE-DRNWLL-------YLLTEEIDQLAPCVTDKAQYKA

VTEPISREYRELLKKA---------WEW-GNTACT---ETVNWAV-R--NIVQQGVTVSS

QRRKLDLIRNIS------------------------------------------------

---------------------------------------------

>ANQ43295.1_type_I_interferon_33_Xenopus_tropicalis

------------------------MAPG---EINCLASLAMATT--ILYLLFP-------

-----------------CRWPD---------------KKGETIDNEILKVFDNLQL---Y

KEIEDDCH---FEVHDIGFI-NVSSP---------------------------GEAAA-M

VLTIFWETKNFYQKKTQCFRLPEE-DRNWLL-------YLLTEEIDQLAPCVTDKAQYKA

VTEPISREYRELLKKA---------WEW-GNTACT---ETVNWAV-R--NIVQQGVTFLL

KGEN----------------STSSVISLNIVCIAT-------------------------

----------------------AKQIHSLKETL------------

>ANQ43263.1_type_I_interferon_1_Xenopus_tropicalis

------------------------MAPG---EINCLAPLAVATT--ILYLLVR-------

---------PSSATSQECRWPD---------------KKGENIDNEILKVFDNLQL---Y

KEIQDDCH---FEVHDIGFI-NVSSP---------------------------GEAAA-M

VLTIFWETKNFYQKKTQRFRLPEE-DRNWLL-------YLLTEEIHQLAPCVTDKVQYKA

VTEPISREYRELLKKA---------WEW-GNTACT---ETVNWAV-R--NIVQQGVTVSS

KRRKLDLIRNIS------------------------------------------------

---------------------------------------------

>ANQ43333.1_type_I_interferon_21_Xenopus_laevis

------------------------MAPGLIKQTNCLETLLMVTT--ILYLLVQ-------

---------PSSAGSRECPWFD---------------KRGELVDNEILKVFDNLQP---T

EENKYNCR---FKISNKGFF-NNRSQ---------------------------EEAAA-I

VFVVFRETKKFYKKNCSRLSV----NCNQLL-------HLLQQQIDKLHTCNINTVQYKE

ATQAISKEYRKLRKKGFGSNRLVWFQPF-GSNDLMVRFERFNRSF-RTISSFDRTVFVRS

QRENVGVSVQSGTIQRQRDCDGTEYIS-DSCYNAS----------------FGP------

-----Y--------------------SSLQR--------------

>ANQ43331.1_type_I_interferon_19_Xenopus_laevis

MPAR----------------MRGLMSAG------------------MCELPSA-------

---------HMRRSTKQCAYAQNSKKMGREGTGHGVDDRAELIDHKIQKAFDHFPP---N

KNHEDDCL---LEIPDNLFP-KNRSQ---------------------------AEVAA-M

VSIVHRRTM----KDLHRFRLPVE-KHNELR-------LLLQKQINQLAPCIKGKAQYKE

ATELIYKQYKEISKKV---------REW-ESTECT---ETINWAF-N--NILQQAVQ---

------------------------------------------------------------

------------------------------RLLK-----------

>AWK27008.1_IFN_1.5_Xenopus_tropicalis

------------------------MSQS-------PIILPVVLL--LLPVLVL-------

-------------CSPECPWLD---------------NKGEFQVQKILTVLDHMEP---T

EEIPDDCF---LPLPPIDFT-HNMSL---------------------------GAAAA-I

VDKVARETIRLYSKKCHQLGHTQK-DCSELL-------HLLHQLSQWVAPCLTDTGEYKE

VTEQVTKQFRQ---MA---------RNP-GNAACA---RHNIWAA-TT-HYYQQVARLTS

LMVKQRLMN---------------------------------------------------

---------------------------------------------

>ANQ43279.1_type_I_interferon_17_Xenopus_tropicalis

------------------------MSQS-------PIILPVVLL--LLPVLVL-------

-------------CSPECPWLD---------------NKGEFQVQKILTVLDHMEP---T

EEIPDDCF---LPLPPIDFT-HNMSL---------------------------GAAAA-I

VDKVARETIRLYSKKCHQLGHTQK-DCSELL-------HLLHQLSQWVAPCLTDTGEYKE

VTEQVTKQFRQ---MA---------RNP-GNAACA---RHNIWAA-TT-HYYQQVARLTS

LMVKQRLMN---------------------------------------------------

---------------------------------------------

>AWK27006.1_IFN_1.3_Xenopus_tropicalis

------------------------MSQS-------PIILPVVLL--LLPVLVL-------

-------------CSPECPWLD---------------NKGEFQVQKILTVLDHMEP---T

EEIPDDCF---LPLPPIDFT-HNMSL---------------------------GAAAA-I

VDKVARETIRLYSKKCHQLGHTQK-DCSELL-------HLLHQLSQWVAPCLTDTGEYKE

VTEQVTKQFRQ---MA---------RNP-GNAACA---RHNIWAA-TT-HYYQQVARLTS

LMVKQHLMN---------------------------------------------------

---------------------------------------------

>ANQ43284.1_type_I_interferon_22_Xenopus_tropicalis

------------------------MSQS-------PIILPVVLL--LLPVLVL-------

-------------CSPECPWLD---------------NKGEFQVQKILTVLDHMEP---T

EEIPDDCF---LPLPPIDFT-HNMSL---------------------------GAAAA-I

VDKVARETIRLYSKKCHQLGHTQK-DCSELL-------HLLHQLSQWVAPCLTDTGEYKE

VTEQVTKQFRQ---MA---------RNP-GNAACA---RHNIWAA-TT-HYYQQVARLTS

LMVKQHLMN---------------------------------------------------

---------------------------------------------

>AWK27010.1_IFN_1.7_Xenopus_tropicalis

------------------------MSQTPS-----PKLLPVVL-----------------

---------PSRGSSPECPWLD---------------NKGEFLVHKILTVLDRMEP---M

EEIPDDCF---LPLPPIDFT-HNMSL---------------------------GAAAA-I

VDKVARKTIRLYSKKCHQLGHTQK-DCSELL-------HLLHQLRQWVAPCLTDTWEYKE

VTEPILEQLRQQRQTA---------PNP-GNTACA---RHSIWAA-TT-HYYRQVARLTS

LMFKQHLMN---------------------------------------------------

---------------------------------------------

>ANQ43277.1_type_I_interferon_15_Xenopus_tropicalis

------------------------MSQTPS-----PKLLPVVL-----------------

---------PSRGSSPECPWLD---------------NKGEFLVHKILTVLDRMEP---M

EEIPDDCF---LPLPPIDFT-HNMSL---------------------------GAAAA-I

VDKVARKTIRLYSKKCHQLGHTQK-DCSELL-------HLLHQLRQWVAPCLTDTWEYKE

VTEPILEQLRQQRQTA---------PNP-GNTACA---RHSIWAA-TT-HYYRQVARLTS

LMFKQHLMN---------------------------------------------------

---------------------------------------------

>AWK27007.1_IFN_1.4_Xenopus_tropicalis

------------------------MSQTPS-----PKLLPVVL-----------------

---------PSRGSSPECPWLD---------------NKGEFLVHKILTVLDCMEP---T

EEIPDDCF---LTLPPIDFT-HNMSL---------------------------GAAAA-I

VDKVARKTIRLYSKKCHQLGHTQK-DCSELL-------HLLHQLRQWVAPCLTDTEEYKE

VTEPILEQLRQQRQTA---------PNP-GNTACA---RHSIWAA-TT-HYYRQVARLTS

LMVKQHLMN---------------------------------------------------

---------------------------------------------

>ANQ43283.1_type_I_interferon_21_Xenopus_tropicalis

------------------------MSQTPS-----PKLLPVVL-----------------

---------PSRGSSPECPWLD---------------NKGEFLVHKILTVLDCMEP---T

EEIPDDCF---LTLPPIDFT-HNMSL---------------------------GAAAA-I

VDKVARKTIRLYSKKCHQLGHTQK-DCSELL-------HLLHQLRQWVAPCLTDTEEYKE

VTEPILEQLRQQRQTA---------PNP-GNTACA---RHSIWAA-TT-HYYRQVARLTS

LMVKQHLMN---------------------------------------------------

---------------------------------------------

>ANQ43274.1_type_I_interferon_12_Xenopus_tropicalis

------------------------MAQS----------LSLSLL--IVSVL---------

---------PSSGTFPECLKLG-------------------FMVQEVQTIFGRMEP---M

EEIPHDCI---LTLPPIGFT-HNMSL---------------------------GAAAA-V

MDKVFSETIRLYRKKCRQLGGTRK-DCSELL-------HLLHQLRHRVAPCLTDTGEYKE

VMELILEHFKQQRWMA---------RNR-GNAACA---RHIIWMA-NA-QNYRQVARLTS

LMIKQRLMN---------------------------------------------------

---------------------------------------------

>ANQ43330.1_type_I_interferon_17_Xenopus_laevis

------------------------MSGI----------QSGILLHIVLGLLLQ-------

---------GPITTSPQCPWPA---------------QNGEFLVNQMLSIFDHLKP---E

KEFMH------KKLPPIGSV-FNISQ---------------------------MEALPMV

ATRVFVETISFYTENCQILGHLQE-QCNKLY-------VLLHQLGEMLAQYKTNTADYKE

AMEAISKKFRKLRRRA---------QKR-GHTAHA---QDLIWLT-VI-EDLRSVAAHLS

VIGTQSLMNGTF------------------------------------------------

---------------------------------------------

>AWK27009.1_IFN_1.6_Xenopus_tropicalis

------------------------MSQS-------PIILPVVLL--LLPVLVL-------

-------------CSPECPWLD---------------NKGEFQVQKILTVLDRMEP---T

EEIPYDCF---LPLPPIDFT-HNMSL---------------------------GAAAA-I

VDKVARKSIRLYSKKCHQLGHTQK-DCSELL-------HLLHQLSQWVAPCLTDKGKYKE

VTEQVTKQFRQ---MA---------QNP-GNAACA---RHNIWAA-TT-HYYQQVARLTS

LKVKQRLMN---------------------------------------------------

---------------------------------------------

>ANQ43278.1_type_I_interferon_16_Xenopus_tropicalis

------------------------MSQS-------PIILPVVLL--LLPVLVL-------

-------------CSPECPWLD---------------NKGEFQVQKILTVLDRMEP---T

EEIPYDCF---LPLPPIDFT-HNMSL---------------------------GAAAA-I

VDKVARKSIRLYSKKCHQLGHTQK-DCSELL-------HLLHQLSQWVAPCLTDKGKYKE

VTEQVTKQFRQ---MA---------QNP-GNAACA---RHNIWAA-TT-HYYQQVARLTS

LKVKQRLMN---------------------------------------------------

---------------------------------------------

>ANQ43280.1_type_I_interferon_18,_partial_Xenopus_tropicalis

------------------------------------------LL----------------

------------------------------------------------------------

-----------LTLP------TTCPL---------------------------GAAAA-I

VDKVARKSIRLYSKKCHQLGHTQK-DCSELL-------HLLHQLSQWVAPYLTDKGKYKE

VTEQVTKQFRQ---MA---------QNP-GNTACA---RHNIWAA-TT-HYYQQVARLTS

LMVKQRLMN---------------------------------------------------

---------------------------------------------

>ANW82730.1_type_I_interferon_1.2_Xenopus_tropicalis

------------------------MSQS-------PIILPVVLL--LLPVLVL-------

-------------CSPECPWLD---------------NKGEFQVQKILTVLDHMEP---T

EEIPNDCF---LPLPPIDFT-HNMSL---------------------------GAAAA-I

VDKVSRETIRLYSKKCHQLGHTQK-DCSELL-------HLLHQLSQWVAPCLTDTGEYKE

VTEQVTKQFRQ---TA---------QNP-GNTACA---RHNIWAA-TT-HYYQQVARLTS

LMIKQRLMN---------------------------------------------------

---------------------------------------------

>ANQ43285.1_type_I_interferon_23_Xenopus_tropicalis

------------------------MSQS-------PIILPVVLL--LLPVLVL-------

-------------CSPECPWLD---------------NKGEFQVQKILTVLDHMEP---T

EEIPNDCF---LPLPPIDFT-HNMSL---------------------------GAAAA-I

VDKVSRETIRLYSKKCHQLGHTQK-DCSELL-------HLLHQLSQWVAPCLTDTGEYKE

VTEQVTKQFRQ---TA---------QNP-GNTACA---RHNIWAA-TT-HYYQQVARLTS

LMIKQRLMN---------------------------------------------------

---------------------------------------------

>AWK27005.1_IFN_1.1_Xenopus_tropicalis

------------------------MSQS-------PIILPVVLL--LLPVLVL-------

---------PSRGSSPECPWLD---------------NKGEFQVQKILTVLGSMEP---M

EEIPYDCF---LPLPPIDFT-HNMSL---------------------------GAAAA-I

VDKVARETIRLYSKKCHQLGHTQK-DCSELL-------HLLHQLSQWVAPCLTDTGEYKE

VTEQVTKQFRQ---MA---------RNP-GNAACA---RHNIWAA-TT-HYYQQVARLTS

LMIKQRLMN---------------------------------------------------

---------------------------------------------

>ANQ43286.1_type_I_interferon_24_Xenopus_tropicalis

------------------------MSQS-------PIILPVVLL--LLPVLVL-------

---------PSRGSSPECPWLD---------------NKGEFQVQKILTVLGSMEP---M

EEIPYDCF---LPLPPIDFT-HNMSL---------------------------GAAAA-I

VDKVARETIRLYSKKCHQLGHTQK-DCSELL-------HLLHQLSQWVAPCLTDTGEYKE

VTEQVTKQFRQ---MA---------RNP-GNAACA---RHNIWAA-TT-HYYQQVARLTS

LMIKQRLMN---------------------------------------------------

---------------------------------------------

>ANQ43321.1_type_I_interferon_9_Xenopus_laevis

------------------------MSCT----------LLLILF--IS--LMP-------

---------LANASSLRCPQLQ---------------VKGQFLLNKILSNVEHLET---I

ENIPHYCF---TQFRLPTIT-FDKSP---------------------------TEAVPIM

AIKVFNETTDFYAENCKRLGLTRE-LCWELC-------QLLQQLIEESAPCMTYTVAYKD

ETKHISRHYRQFKKLA---------QKR-DKIYCV---RKFIWSV-NE-FTVGYSSLISH

AKTASHELNILTD------------------------------------L-LG-------

---------------------------------------------

>ANQ43319.1_type_I_interferon_7_Xenopus_laevis

------------------------MSCT----------LLLILF--IS--LMP-------

---------LSNASSLRCPQLH---------------MKGQFLLNKMLSNFEHLEP---I

ENIPHYCF---PRLHLHTLV-VDKSP---------------------------TEAVPFM

AIKVLNETNDFYLENCKRLGLTRE-LCWELF-------QILHQLIEEFAPCMTYTVAYKD

ETKHISRHYRQIRKLA---------QKR-GNSSCV---RKFIWSV-NE-KNLQWVTLLLS

RMQKQLLMN---------------------------------------------------

---------------------------------------------

>AWK27015.1_IFN_3.3_Xenopus_tropicalis

------------------------MSRA----------LSLILL--MVPLLLV-------

---------LSSGGSHAYPLLK---------------KGGEHLLNEMLMKNDCLAP---E

E--KHECF---PRLPRISWA-FDTYQ---------------------------VEAAVGA

AIQFLRETFRFYNENFEALGLSQE-LGHELL-------QLLDRLIEEWAPYVTDTTVNED

VSQGISEFYRQLINLV---------RKE-GNAACV-------LSV-IS-ENLYQVAHLLS

RVQKHLLVNDTSRVGYFK---DE-------------------------------------

---------------------------------------------

>ANQ43270.1_type_I_interferon_8_Xenopus_tropicalis

M---------------------DIMSRA----------LSLILL--MVPLLLV-------

---------LSSGGSHAYPLLK---------------KGGEHLLNEMLMKNDCLAP---E

E--KHECF---PRLPRISWA-FDTYQ---------------------------VEAAVGA

AIQFLRETFRFYNENFEALGLSQE-LGHELL-------QLLDRLIEEWAPYVTDTTVNED

VSQGISEFYRQLINLV---------RKE-GNAACV-------LSV-IS-ENLYQVAHLLS

RVQKHLLVNDTSRVGYFK---DE-------------------------------------

---------------------------------------------

>ANQ43297.1_type_I_interferon_35_Xenopus_tropicalis

------------------------------------------------------------

----------------------------------------------MLMKNDCLAP---E

E--KHECF---PRLPRISWA-FDTYQ---------------------------VEAAVGA

AIQFLRETFRFYNENFEALGLSQE-LGHELL-------QLLDRLIEEWAPYVTDTTVNED

VSQGISEFYRQLINLV---------RKE-GNAACV-------LSV-IS-ENLYQVAHLLS

RVQKHLLVNDTSREEH-----DTTILSLPHVASTPT--RKLPIGTMHWRLSIG-------

------RYNIPS--RALTLAPN----KALTRILQ-----------

>ANQ43291.1_type_I_interferon_29_Xenopus_tropicalis

------------------------MSRT----------LSLILL--MVPLLLV-------

---------LSSGGSHAYPLLK---------------KGGDHLLNEMLMKNDCLAP---E

K--IHECF---PRLPRISWA-FNTYQ---------------------------VEAAVGA

AIQFLRGTFRFYNENFEALGLSQE-LGHELL-------QLLDRLIEEWAPYVTDTTLNED

VSQGISEFYRQLIKLV---------RKE-GNAACV-------------------------

------------------------------------------------------------

---------------------------------------------

>AWK27014.1_IFN_3.2_Xenopus_tropicalis

------------------------MSRT----------LSLILL--TVPLLLV-------

---------LSSGGSHAYPLLK---------------KGGDHLLNEMLMKNDRLAP---E

E--IHECF---PRLPRISWA-FDTYQ---------------------------VEAAVGA

AIQFLRETFRFYNENFEALGLSQE-LGHELL-------QLLDRLIEEWAPYVTDTTVNED

VSQGISEFYRQLIKLV---------RKE-GNAACV-------LSV-IS-ENLYQVAHLLS

RVQKHLLVNETSRVGYFK---DE-------------------------------------

---------------------------------------------

>ANQ43272.1_type_I_interferon_10_Xenopus_tropicalis

M---------------------DIMSRT----------LSLILL--TVPLLLV-------

---------LSSGGSHAYPLLK---------------KGGDHLLNEMLMKNDRLAP---E

E--IHECF---PRLPRISWA-FDTYQ---------------------------VEAAVGA

AIQFLRETFRFYNENFEALGLSQE-LGHELL-------QLLDRLIEEWAPYVTDTTVNED

VSQGISEFYRQLIKLV---------RKE-GNAACV-------LSV-IS-ENLYQVAHLLS

RVQKHLLVNETSRVGYFK---DE-------------------------------------

---------------------------------------------

>ANQ43266.1_type_I_interferon_4_Xenopus_tropicalis

M---------------------DIMSRT----------LSLILL--MVPLLLV-------

---------LSSGGSHGCPLLK---------------KGGEHLLNEMLSTNDRLVL---E

E--IHECF---PRLPRISWA-FGTYQ---------------------------VEAAVGA

AIQFFNETFRFYNENFEALGLSQE-LCHEQL-------QLLQRLIEEWAPCVTDTKANKD

VSRGISRFYRRLRKLV---------RKQ-GNAACV-------LSV-IS-ENLYQVAHLLS

RVRKHLLINEKGH--------DRTILSLPPVASMPRPPHKLTRG-----LSIG-------

------RYRIPS--KSADSSTKHFS-KALTQILQ-----------

>ANQ43265.1_type_I_interferon_3_Xenopus_tropicalis

M---------------------DIMSHT----------LSLILL--MVPLLLV-------

---------LSSGGSHGCPLLK---------------KGGEHLLNEMLSTNDRLVL---E

E--IHECF---PRLPRISWA-FGTYQ---------------------------VEAAVGA

AIQFFNETFRFYNENFEALGLSQE-LCHEQL-------QLLQRLIEEWAPCVTDTKANKD

VSRGISRFYRRLRKLV---------RKQ-GNAACV-------LSV-IS-ENLYQVAHLLS

RVRKHLLINEKGH--------DRTILSLPPVASMPRPPHKLTRG-----LNANAQTPHRN

HALETFHWAVPHSFQSADSSTKQSS-YSNLTITKLSPLLKFLLKP

>ANQ43267.1_type_I_interferon_5_Xenopus_tropicalis

M---------------------DIMSRT----------LSLILL--MVPLLLV-------

---------LSSGGSHGCPLLK---------------KGGEHLLNEMLSTNDRLVL---E

E--IHECF---PRLPRISWA-FDTYQ---------------------------VEAAVGA

AIQFFNETFRFYNENFEALGLSQE-LCHEQL-------QLLQRLIEEWAPCVTDTKANKD

VSRGISRFYRRLRKLV---------RKQ-GNAACV-------LSV-IS-ENLYQVAHLLS

RVRKHLLINEKGH--------DTTILSLPPVASMPRPPHKLPIG-----ISIG-------

------RYRIPS--KSADSSTKHFS-KALTQILQ-----------

>ANQ43271.1_type_I_interferon_9_Xenopus_tropicalis

M---------------------DIMSRT----------LSLILL--MVPLLLV-------

---------LSSGGSHGCPLLK---------------KGGEHLLNEMLLTNDRLVL---E

E--IHECF---PRLPRISWA-FGTYQ---------------------------VEAAVGA

AIQFFIETFRFYNENFEALGLSQE-LCHEQL-------QLLQRLIEEWAPCVSDTKANKD

VSRGISRFYRRLRKLV---------RKQ-GNAACV-------LSV-IS-ENLYQVAHLLS

RVRKHLLINEKGH--------DTTILSLPPVALMPRPPHKLPRG-----LSIG-------

------RYRIPS--KSADSSTKHFS-KALTQILQ-----------

>ANQ43328.1_type_I_interferon_15_Xenopus_laevis

------------------------MSCT----------LLLILF--IS--LMP-------

---------LSNASSLRCPQLQ---------------VKGQFLLNKMLSNFENFEP---K

ENIPHYCF---PRLRLPTIV-VDRSP---------------------------TEAVPIM

AIKVFNETNDFYVENCKRLGLTRE-LCWELC-------QILHQLIEEFAPCMTYTVAYKD

ETKHISRHYRQLRKLA---------QKR-GNSSCV---RKFIWSV-NV-KNLQWVTLLLS

RMQKQLLMN---------------------------------------------------

---------------------------------------------

>ANQ43326.1_type_I_interferon_13_Xenopus_laevis

------------------------MSCT----------LLLILF--IS--LMP-------

---------LSNASSLRCPQLQ---------------VKGQFLLNKMLSNFENLEP---K

ENIPHYCF---PRLRLPTIV-VDKSP---------------------------TEAVPIM

AIKVFNETTDFYVENCKRLGLTRE-LCWELC-------QILHQLMEEFAPCMTYTVAYKD

ETKHISRHYRQLKKLA---------QKR-GNSSCV---RKFIWSV-NE-KNFQWVTLLLS

RMQKQLLMN---------------------------------------------------

---------------------------------------------

>AWK27017.1_IFN_3.5_Xenopus_tropicalis

------------------------MSRT----------LSLILL--TVPLLLV-------

---------LSSGGYHGYPLPK---------------KGGEHLLNEMLMKNDRLVL---E

E--IHECF---PRLPRISWA-FDTYQ---------------------------VEAAVGA

AIQFFNATIYFFNENFEALGLSQE-LCHELL-------QILDRLIEEWAPYVTDTTVNED

VSQGISEFYRQLIKLV---------PKE-GNAACV-------LSV-IS-ENLYQVAHLLS

RVRKHLLVNETSRVGYFK---DE-------------------------------------

---------------------------------------------

>ANQ43264.1_type_I_interferon_2_Xenopus_tropicalis

------------------------MSRT----------LSLILL--TVPLLLV-------

---------LSSGGYHGYPLPK---------------KGGEHLLNEMLMKNDRLVL---E

E--IHECF---PRLPRISWA-FDTYQ---------------------------VEAAVGA

AIQFFNATIYFFNENFEALGLSQE-LCHELL-------QILDRLIEEWAPYVTDTTVNED

VSQGISEFYRQLIKLV---------PKE-GNAACV-------LSV-IS-ENLYQVAHLLS

RVRKHLLVNETSRVGYFK---DE-------------------------------------

---------------------------------------------

>ANQ43292.1_type_I_interferon_30_Xenopus_tropicalis

------------------------------------------------------------

----------------------------------------------MLMKIDRLVL---E

E--IHECF---PRLPRISWA-FDTYQ---------------------------VEAAVGA

AIQFFNATIYFFNENFEALGLSQE-LSHELL-------QILDRLIEEWAPYVTDTTVNED

VSQGISEFYRQLIKLV---------PKE-GNAACV-------LSV-IS-ENLYQVAHLLS

RVRKHLLVNETSRVGYFK---DE-------------------------------------

---------------------------------------------

>AWK27016.1_IFN_3.4_Xenopus_tropicalis

------------------------MSRT----------LSLILL--TVPLLLV-------

---------LSSGGYHGYPLPK---------------KGGEHLLNEMLMKNDRLVL---E

E--IHECF---PRLPRISWA-FDTYQ---------------------------VEASVGA

AIQFFRETFRFYKENFEALGLSQE-LGHELL-------QILDRLIEEWAPYVTDTTVNED

VSQGISEFYRQLIKLV---------PKP-GNAACV-------LSV-IS-ENLYQAAHLLS

RVQKHLLVNETSRVGYFK---DE-------------------------------------

---------------------------------------------

>ANQ43269.1_type_I_interferon_7_Xenopus_tropicalis

------------------------MSRT----------LSLILL--TVPLLLV-------

---------LSSGGYHGYPLPK---------------KGGEHLLNEMLMKNDRLVL---E

E--IHECF---PRLPRISWA-FDTYQ---------------------------VEASVGA

AIQFFRETFRFYKENFEALGLSQE-LGHELL-------QILDRLIEEWAPYVTDTTVNED

VSQGISEFYRQLIKLV---------PKP-GNAACV-------LSV-IS-ENLYQAAHLLS

RVQKHLLVNETSRVGYFK---DE-------------------------------------

---------------------------------------------

>ANW82732.1_type_I_interferon_3.1_Xenopus_tropicalis

------------------------MNRT----------LSLILL--MVPLLLV-------

---------LSSGGSHGYPLLK---------------KGGEHLLNEMLMKNDRLML---E

E--IHECF---PRLPRINWA-FDTYQ---------------------------VEASVGA

AIQFFRETFRFYNENFEALGLSQE-LGHELL-------QILDRLIEEWAPYVTDTTVNED

VSQGISEFYRQLIKLV---------PKP-GNAACV-------LSV-IS-ENLYQVAHLLS

RCQKHLLVNETSRVGYFK---DE-------------------------------------

---------------------------------------------

>ANQ43273.1_type_I_interferon_11_Xenopus_tropicalis

M---------------------DIMNRT----------LSLILL--MVPLLLV-------

---------LSSGGSHGYPLLK---------------KGGEHLLNEMLMKNDRLML---E

E--IHECF---PRLPRINWA-FDTYQ---------------------------VEASVGA

AIQFFRETFRFYNENFEALGLSQE-LGHELL-------QILDRLIEEWAPYVTDTTVNED

VSQGISEFYRQLIKLV---------PKP-GNAACV-------LSV-IS-ENLYQVAHLLS

RCQKHLLVNETSRVGYFK---DE-------------------------------------

---------------------------------------------

>AWL54284.1_type_I_interferon_Nanorana_parkeri

------------------------MTST--SSWT------------VLLLLSL-------

---------SSMVSAQTCKWLH---------------RNQQEWTRQILHNFNQMVP---A

EETGQPCQDNSPHLPVIDSL-YSITQ---------------------------AESAAIA

VHEVANQIVQFYRRNQDRMDYHLP-AWEKLQ-------ELLNYQEQHLSDCIPEGAENQL

FIQGISQRFNTLQRIL---------EEQ-KDTACA---WRTVHTE-IG-RNLRLAAQLSA

RMRNQKSHD---------------------------------------------------

---------------------------------------------

>AWL54312.1_type_I_interferon_1_Nanorana_parkeri

------------------------MAAT--YSWT------------VVIFLSL-------

---------SSIISAQTCKWLH---------------PNQGVVTRQILQNFQQMIP---A

EDSPGVCLNSNFSLPDLRHL-YQITA---------------------------VQSAAIA

VREVGNQTTQFYRRYEERLDYHWP-AWERLQ-------ELLYYQEQQLSDCIPEGAENQL

FIQGISQQFTTLQRIL---------EEQ-EDEVCA---RRTAHAE-IR-RNLLLAGQLLS

RMRRQRLQRHLF------------------------------------------------

---------------------------------------------

>AWL54313.1_type_I_interferon_2_Nanorana_parkeri

------------------------MTST--SSWT------------VLLLLSL-------

---------SSVVSAQTCKWLH---------------RNQEAWTRQILHNFNHMVP---A

ERTGQPCQDNSPHLPVIDSL-YSITQ---------------------------AESAAIA

VREVTNQTLRFYRRNQDRMDYHRP-AWEKLQ-------ELLHYQEQHLSDCIPEGAENQL

FIQGITQRFNSLQRIL---------EEQ-KDNACA---WRTAHTE-IG-RNLRLAAQLSS

RMRKQKSHDQQSAQ----------------------------------------------

---------------------------------------------

>Napa_2

------------------------------------------------------------

-----------MVSAQTCKWLH---------------RNQEAWTRQILHNFNQMVP---A

EKTGQPCQDNSPHLPVIDSL-YSITQ---------------------------AESAAIA

-----DETREFHIYTRTQLARPSRCGWRREKCRKTAEGKYVSYTVNQIGKGSGEGRE---

----AVKPVLAVDGMR---------ALT-GSAAVR---WL--------------------

RVSRYILLVKPQCL----------------------------------------------

---------------------------------------------

>Xetr_4

------------------------MLPM--GQWS------------VLLLLSL-------

---------TSIVHSQSCKWLH---------------PKQEYLNTQILKAFNEMMP---L

KETEEICEEHPTDLPNTEST-YSVSQ---------------------------VEAGALA

VREVLNETMRFYMKHHESMGCKQQ-AWERFQ-------QLLYYQIHQLEACVSQTEENDL

LKESISEEFNLLETMV---------LEK-DNSACV---WDFIHLE-TR-RNLQQVLQLSS

RLRRQRLLQRPQ------------------------------------------------

---------------------------------------------

1. **IFN1 amniote only with closely related cartilaginous fish and amphibian:**

>IFNA_HUMAN

MALTFALLVALLVLSCKSSCSVGCDLPQTHLGS--RRTLMLLAQMRRISLFSCLKDRHDF

GPQEEF-G-NQFQKAETIPVLHEMIQQIFNLFSTKDSSAAWDETLLDKFYTELYQQLNDL

EACVIQGVGVTETPLMKEDSILAVRKYFQRITLYLKEKKYSPCAWEVVRAEIMRSFSLST

NLQESLRSK

>Loaf_11

MAFSFLLLIALVVLSCNSTCSLGCDLPQSHLAN--RRTMMLLGQMRRISPFSCLKDRNDF

GPQEELDG-NKFQKAQAISVHHEMIQQTFNLFSLQASSAAWDKTLLDKLYTGLYQQLNDL

EVCLMQEMGVEEAPVINEDSMLAVRKYFQRITVYLTEKKYSPCAWETVRAEVMSSFSAST

NWKERLRSK

>IFNA_horse

MALPVSLLMALVVLSCHSICSLGCDLPHTHLGN--TRVLMLLGQMRRISPFSCLKDRNDF

GPQEVFDG-NQFRKPQAISAVHETIQQIFHLFSTDGSSAAWDESLLDKLYTGLYQQLTEL

EACLSQEVGVEETPLMNEDSLLAVRRYFQRIALYLQEKKYSPCAWEIVRAEIMRSFSSST

NLPQS----

>IFNA_pig

MAPTSAFLTALVLLSCNAICSLGCDLPQTHLAH--TRALRLLAQMRRISPFSCLDHRRDF

GPHEAFGG-NQVQKAQAMALVHEMLQQTFQLFSTEGSAAAWNESLLHQFCTGLDQQLRDL

EACVMQEAGLEGTPLLEEDSILAVRKYFHRLTLYLQEKSYSPCAWEIVRAEVMRSFSSSR

NLQDRLRKK

>Oror_10

MAPTVSLLLALVLLSCHSNCSLGCDLPQTHLAN--TRALMLLQQMRRISPFSCLKDRNDF

GPQEAFGG-NQFQKAQAIAVVHEMIQQTFQLFSTEGSAAAWDETLLDKFCTALYQQLTDL

QACLMQEAGLEGTPLLKEDSILAVRKYFHRITVYLQEKKYSPCAWEIVRAEVMRSFSSST

NL-------

>IFNA_cow

MAPAWSFRLALLLLSCNAICSLGCHLPHTHLAN--RRVLMLLGQLRRVSPSSCLQDRNDF

APQEALGG-SQLQKAQAISVLHEVTQHTFQLFSTEGSATMWDESLLDKLRDALDQQLTDL

QFCLRQEEELQGAPLLKEDSSLAVRKYFHRLTLYLQEKRHSPCAWEVVRAQVMRAFSSST

NLQESFRRK

>Ptva_8

MALLFSFLMAMVVLSCQSICSLGCDLPQTHLVN--RRALMLLGQMRRISPFSCLKDREDF

GLQGAFGG-NQFQEAQAIAVFHEMTQQTFLLFCTEVLSAAWDETLLGRFCNGLYQQLDHL

EACQTQELGAEETPLLDEDSTLAVRKYFQRINLYLQEKKHSPCAWEIVRAEIMRSYSLST

HLKEKSRSK

>Ereu_6

MAPSSLFLKALLVLSCSYIFGLGCDLPQSHPVN--RRPLLLLGQMRRLPPFSCLKDRHDF

APQEVFDG-QQFQKAHALSVLHEMLQQIFHLFSTKHSSADWDEGLLNSFCAELHQQLNVL

EGCQTQEVRVEQTPRMK-DSILAMKRYFQRITMYLREKKYSPCAWEIVRVEIIRAFSLST

KLQEKLRSK

>IFNA_mouse

MARLCAFLVMLIVMSYWSTCSLGCDLPHTYLRN--KRALKVLAQMRRLPFLSCLKDRQDF

GPLEKVDN-QQIQKAQAIPVLRDLTQQTLNLFTSKASSAAWNTTLLDSFCNDLHQQLNDL

QTCLMQQVGVQEPPLTQEDALLAVRKYFHRITVYLREKKHSPCAWEVVRAEVWRALSSSV

NLLPRLSEK

>IFNA_sheep

MAFVLSLLMALVLVSYGPGGSLGCDLSQNHLVG--SQNLRLLGQMRRLSLRFCLQDRKDF

APQEMVEG-GQLQEAQAISVLHEMLQQSFNLFHTEHSSAAWDTTLLEHVRTGLHQQLDDL

DACLGEVTGEEDSALGRTGPTLAMKTYFQGIHVYLKEKGYSDCAWEIVRLEIMRSLSSST

SLHKRLRMM

>IFNO_cow

MAFVLSLLMALVLVSYGPGGSLGCDLSPNHLVG--RQNLRLLGQMRRLSPRFCLQDRKDF

APQEMVEV-SQFQEAQAISVLHEMLQQSFNLFHKERSSAAWDTTLLEQLLTGLHQQLDDL

DACLGLLTGEEDSALGRTGPTLAMKRYFQGIHVYLQEKGYSDCAWEIVRLEIMRSLSSST

SLQERLRMM

>IFND_human

MAFVLSLLMALVLVSYGPGGSLGCDLSQNHLVG--RKNLRLLDEMRRLSPHFCLQDRKDF

APQEMVEG-GQLQEAQAISVLHEMLQQSFNLFHTEHSSAAWDTTLLEPCRTGLHQQLDNL

DACLGQVMGEEDSALGRTGPTLALKRYFQGIHVYLKEKGYSDCAWETVRLEIMRSFSSLI

SLQERLRMM

>Oror_9

MAFVLPLLTALVVFSYGPGGSLGCDLSQNHRIS--RKNFMLLGQMRRISPRFCLKDRKDF

GPQDMVDG-SQLPKAQATSVLHEMLQQVFCLFHTERSTATWDTSLLDKLRTGLHQQLEDL

DACLVQAMGDEETALGVTGPTLAVKRYFQGIHLYLKEKKYSDCAWEIVRVEIMRSLSSST

NLQERLRIM

>IFNT_cow

MAFVLSLLMALVLVSYGPGRSLGCYLSEDHLGA--RENLRLLARMNRLSPHPCLQDRKDF

GPQEMVEG-SQLQKDQAISVLHEMLQQCFNLFHIEHSSAAWNTTLLEQLCTGLQQQLEDL

DACLGPVMGEKDSDMGRMGPILTVKRYFQDIHVYLKEKEYSDCAWEIIRVEMMRALSSST

TLQKRLRKM

>IFNT_sheep

MAFVLSLLMALVLVSYGPGGSLGCYLSQRLLDA--RENLRLLDRMNRLSPHSCLQDRKDF

GPQEMVEG-DQLQKDQAFPVLYEMLQQSFNLFYTEHSSAAWDTTLLEQLCTGLQQQLDHL

DTCRGQVMGEKDSELGNMDPIVTVKKYFQGIHDYLQEKGYSDCAWEIVRVEMMRALTSST

TLQKRLTKM

>IFNT_goat

MAFVLSLLMALVLVSYGPGGSLGCYLSRRLLDA--RENLRLLDRMNRLSPHSCQQDRKDF

GPQEMVEG-DQLQKDQASCVLYEMLQQSFNLFYTEHSSAAWDTTLLDQLCTGLQQQLDHL

DTCRGQVMGEKDSELGNMDPIVTVKKYFQGIYYYLQEKGYSDCAWETVRVEMMRALTAST

TLQKRLTKT

>Loaf_4

MALLLSLLTALVVFSCGPAPSLGCDLPQNHVAS--EKTVDLLDQMQRCPTFFCLDDRKDF

RPQEMVDG-SQLQKAQAIAFLHEMLQQIFDLFRTMDSFAAWNTTLLNQLLNGLPEQQEDL

ETCFMQAMEEGKSALPIEGPALAVKEYFEGIRFYLKEKEYSDCAWEFVRVEIRRSFSSST

ALQERLRRK

>Loaf_5

MAFLLFLLTALVVFGCGPAPSLGCDLSKKHLTS--KKTFVVLDQMRRLSPFSCLKERKDF

RPQEMVDG-SQLQKAQVISVLHEMLQQIFNLFHTKDSSAAWNTTLLDQLHSGLYLQLEDL

EACLVQAMEEEESVLAIESSALAVKRYFQGIHSYLKEKEYSDCAWEIVRVEIKRSFSSST

NLQERLRRK
[truncated: 94,395 more chars]
