# Supplementary material for: Discovery of All Three Types in Cartilaginous Fishes Enables Phylogenetic Resolution of the Origins and Evolution of Interferons
Source: Front Immunol. 2019 Jul 12;10:1558. doi: 10.3389/fimmu.2019.01558 (PMC6640115; doi:10.3389/fimmu.2019.01558)

Discovery of all three types in cartilaginous fishes enables phylogenetic resolution of the origins and evolution of interferons

Anthony K. Redmond, Jun Zou, Christopher J. Secombes, Daniel J. Macqueen, Helen Dooley

Supplementary Figures

Figure S1. Multiple sequence alignment of catshark IFN-λ to human interferons and IL-10. Key residues associated with IFN-λ3-receptor binding are highlighted in blue, while Phe158 which is deemed necessary for binding is shown in red. Cys pairs are in green.

Figure S2. Summary of top hits and structure predictions from Phyre2 with confidence >25% for catshark IFN-λ.

Figure S3. Full tree topology and posterior probabilities of the relaxed clock (uncorrelated lognormal) rooted class II α-helical cytokine family phylogeny under JTT+I+Γ in Fig 2B. Branch colours are as per Fig 2B.

Figure S4. Full tree topology and ultrafast bootstraps for the IFN3 tree in Fig 4. Branch colours, and stars are as per Fig 2B.

Figure S5. PhyloBayes CHOM tree from Fig 5A with all sequence titles and posterior probabilities. Branch colours are as per Fig 5A.

Figure S6. Full titles and posterior probabilities for the BEAST maximum clade credibility tree of the full IFN1 dataset, including compositionally biased sequences shown in Fig 5E. The analysis was performed under JTT+Γ with a relaxed clock (uncorrelated lognormal) model and a yule speciation prior. Colours as per Fig. 5E.

Figure S7. PhyloBayes tree of the CHOM dataset under JTT+Γ with the amphibian sister group to all other IFN1s, and the reptile IFN1s most distantly related mammal IFN1s removed. This was designed to detect if a branching artefact might be driving the placement of the cartilaginous fish genes sister to amniote IFN1s. Colours as per Fig. 5A.

Figure S8. PhyloBayes trees of the IFN1 CHOM dataset under the tested mixture models, all of which fit poorly compared to JTT+Γ according to 10-fold Bayesian cross-validation analyses, including full titles and posterior probabilities of the UL3+Γ tree shown in Fig 5E. Colours as per Fig. 5E.

Figure S9. Full titles and posterior probabilities for PhyloBayes tree of the IFN1 CHOM dataset using the more distantly related IFN2 as outgroup instead of IFN3 under JTT+Γ in Fig 5E.

Figure S10. Maximum likelihood IFN1 EXT tree under JTT+Γ from Fig 6A showing full tree topology, sequence titles and branch support. Branch colours as per Fig. 5A.

Figure S11. Full tree topology, ultrafast bootstrap supports, and sequence titles of the maximum likelihood consensus tree of the amphibian sister group to other IFN1s under JTT+Γ from Fig 6B.

Figure S12. Full tree topology, ultrafast bootstrap supports, and sequence titles of the maximum likelihood consensus trees from Fig 7A (A), and Fig 7B (B).

Figure S13. Full tree topology, ultrafast bootstrap supports, and sequence titles of the maximum likelihood consensus tree from Fig 8.

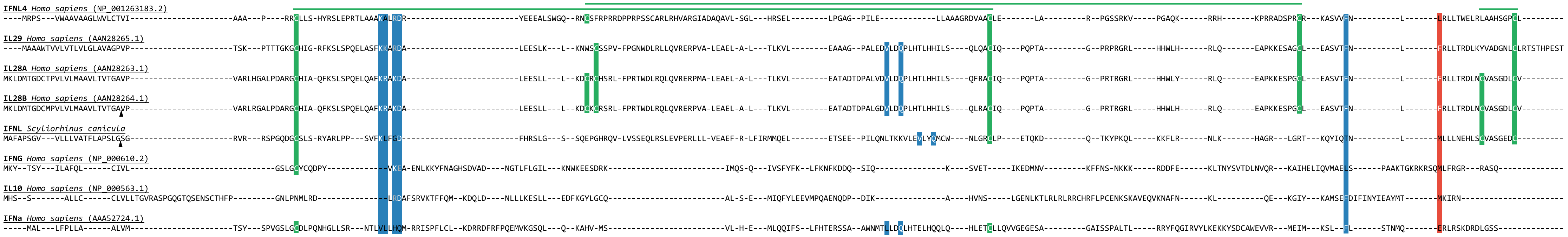
IFNL4 Homo sapiens (NP_001263183.2)
----MRPS---VWAAVAAGLWVLCTVI----------------------AAA---P----RRCLLS-HYRSLEPRTLAAAKALRDR----------------------YEEEALSWGQ--RNCSFRPRRDPPRPSSCARLRHVARGIADAQAVL-SGL---HRSEL--------LPGAG--PILE-----------LLAAAGRDVAACLE-------LA--------R--PGSSRKV-----PGAQK------RRH-------KPRRADSPRCR---KASVVFN----------L-------LRLLTWELRLAAHSGPCL---------
IL29 Homo sapiens (AAN28265.1)
----MAAAWTVVLVTLVLGLAVAGPVP---------------------TSK---PTTTGKGCHIG-RFKSLSPQELASFKKARDA----------------------LEESLK---L--KNWSCSSPV-FPGNWDLRLLQVRERPVA-LEAEL-A-L---TLKVL--------EAAAG--PALEDVLDQPLHTLHHILS----QLQACIQ----PQPTA--------G--PRPRGRL-----HHWLH------RLQ-------EAPKKESAGCL---EASVTFN----------L-------FRLLTRDLKYVADGNLCLRTSTHPEST
IL28A Homo sapiens (AAN28263.1)
MKLDMTGDCTPVLVLMAAVLTVTGAVP---------------------VARLHGALPDARGCHIA-QFKSLSPQELQAFKRAKDA----------------------LEESLL---L--KDCRCHSRL-FPRTWDLRQLQVRERPMA-LEAEL-A-L---TLKVL--------EATADTDPALVDVLDQPLHTLHHILS----QFRACIQ----PQPTA--------G--PRTRGRL-----HHWLY------RLQ-------EAPKKESPGCL---EASVTFN----------L-------FRLLTRDLNCVASGDLCV---------
IL28B Homo sapiens (AAN28264.1)
MKLDMTGDCMPVLVLMAAVLTVTGAVP---------------------VARLRGALPDARGCHIA-QFKSLSPQELQAFKRAKDA----------------------LEESLL---L--KDCKCRSRL-FPRTWDLRQLQVRERPVA-LEAEL-A-L---TLKVL--------EATADTDPALGDVLDQPLHTLHHILS----QLRACIQ----PQPTA--------G--PRTRGRL-----HHWLH------RLQ-------EAPKKESPGCL---EASVTFN----------L-------FRLLTRDLNCVASGDLCV---------
IFNL Scyliorhinus canicula
MAFAPSGV---VLLLVATFLAPSLGSG---------------------RVR---RSPGQDGCSLS-RYARLPP---SVFKLFGD----------------------FHRSLG---S--SQEPGHRQV-LVSSEQLRSLEVPERLLL-VEAEF-R-LFIRMMQEL--------ETSEE--PILQNLTKKVLEVLYQMCW----NLGRCLP----ETQKD--------Q--TKYPKQL-----KKFLR------NLK-------HAGR---LGRT---KQYIQTN----------L-------MLLLNEHLSCVASGEDC----------
IFNG Homo sapiens (NP_000610.2)
MKY--TSY---ILAFQL------CIVL-------------------------------GSLGCYCQDPY-------------VKEA-ENLKKYFNAGHSDVAD----NGTLFLGIL---KNWKEESDRK------------------------IMQS-Q---IVSFYFK--LFKNFKDDQ--SIQ--------------K----SVET-----IKEDMNV---------KFFNS-NKKK-----RDDFE------KLTNYSVTDLNVQR---KAIHELIQVMAELS------PAAKTGKRKRSQMLFRGR---RASQ-------------
IL10 Homo sapiens (NP_000563.1)
MHS--S-------ALLC------CLVLLTGVRASPGQGTQSENSCTHFP---------GNLPNMLRD--------------LRDAFSRVKTFFQM--KDQLD----NLLLKESLL---EDFKGYLGCQ------------------------AL-S-E---MIQFYLEEVMPQAENQDP--DIK--------------A----HVNS-----LGENLKTLRLRLRRCHRFLPCENKSKAVEQVKNAFN------KL----------QE---KGIY---KAMSEFDIFINYIEAYMT--------MKIRN--------------------
IFNa Homo sapiens (AAA52724.1)
-----MAL---LFPLLA------ALVM---------------------TSY---SPVGSLGCDLPQNHGLLSR---NTLVLLHQM-RRISPFLCL--KDRRDFRFPQEMVKGSQL---Q--KAHV-MS------------------------VL-H-E---MLQQIFS--LFHTERSSA--AWNMTLLDQLHTELHQQLQ----HLETCLLQVVGEGESA---------GAISSPALTL-----RRYFQGIRVYLKEKKYSDCAWEVVR---MEIM---KSL--FL------STNMQ--------ERLRSKDRDLGSS--------------

#	Template	Alignment Coverage	3D Model	Confidence	% i.d.	Template Information
1	c3hhcB	35-187(81%)	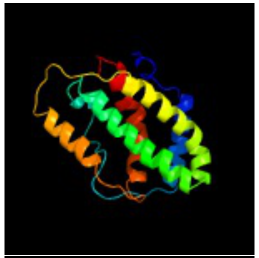	100.0	26	PDB header:cytokine<br>Chain: B: PDB Molecule:interleukin-28b;<br>PDBTitle: interferon-lambda is functionally an interferon but structurally2 related to the il-10 family
2	c3hhcD	70-177(70%)	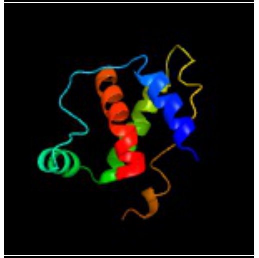	100.0	21	PDB header:cytokine<br>Chain: D: PDB Molecule:interleukin-28b;<br>PDBTitle: interferon-lambda is functionally an interferon but structurally2 related to the il-10 family
3	d1n1fa	33-177(77%)	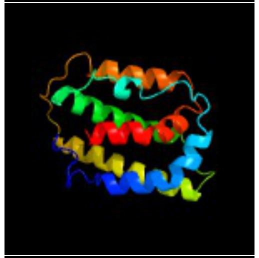	64.4	12	Fold:4-helical cytokines<br>Superfamily:4-helical cytokines<br>Family:Interferons/interleukin-10 (IL-10)
4	c4dohA	49-177(68%)	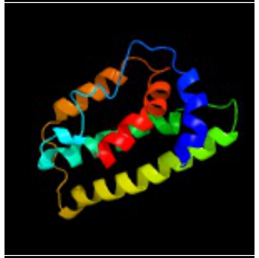	39.1	14	PDB header:signaling protein<br>Chain: A: PDB Molecule:interleukin-20;<br>PDBTitle: il20/il201/il20r2 ternary complex
5	c2n0pA	39-54(8%)	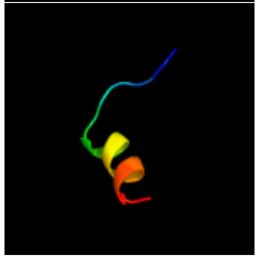	31.4	25	PDB header:structural genomics, unknown function<br>Chain: A: PDB Molecule:uncharacterized protein aq_1974;<br>PDBTitle: solution structure of aquifex aeolicus aq1974

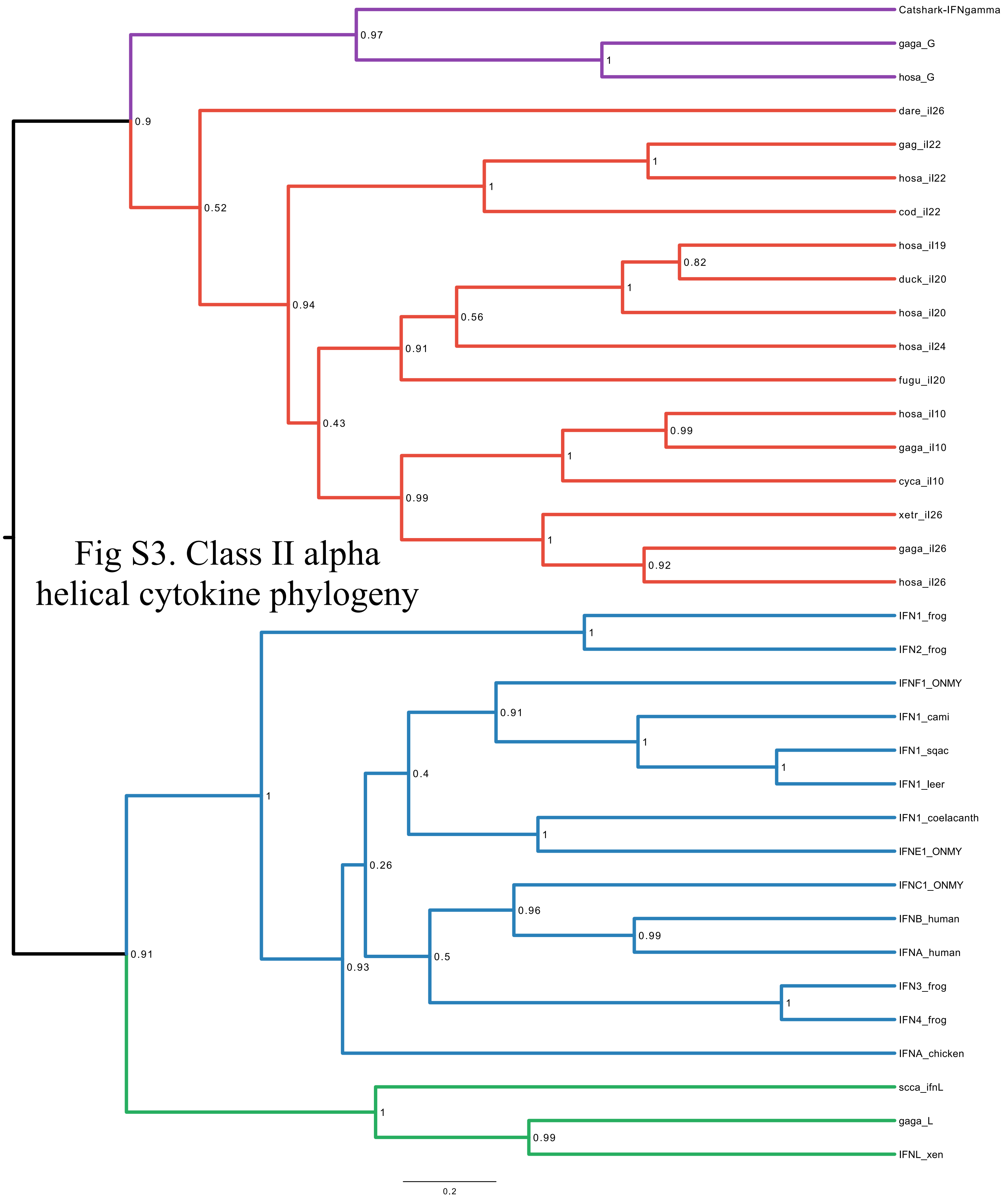

Fig S3. Class II alpha helical cytokine phylogeny

Fig S4. IFN3 phylogeny

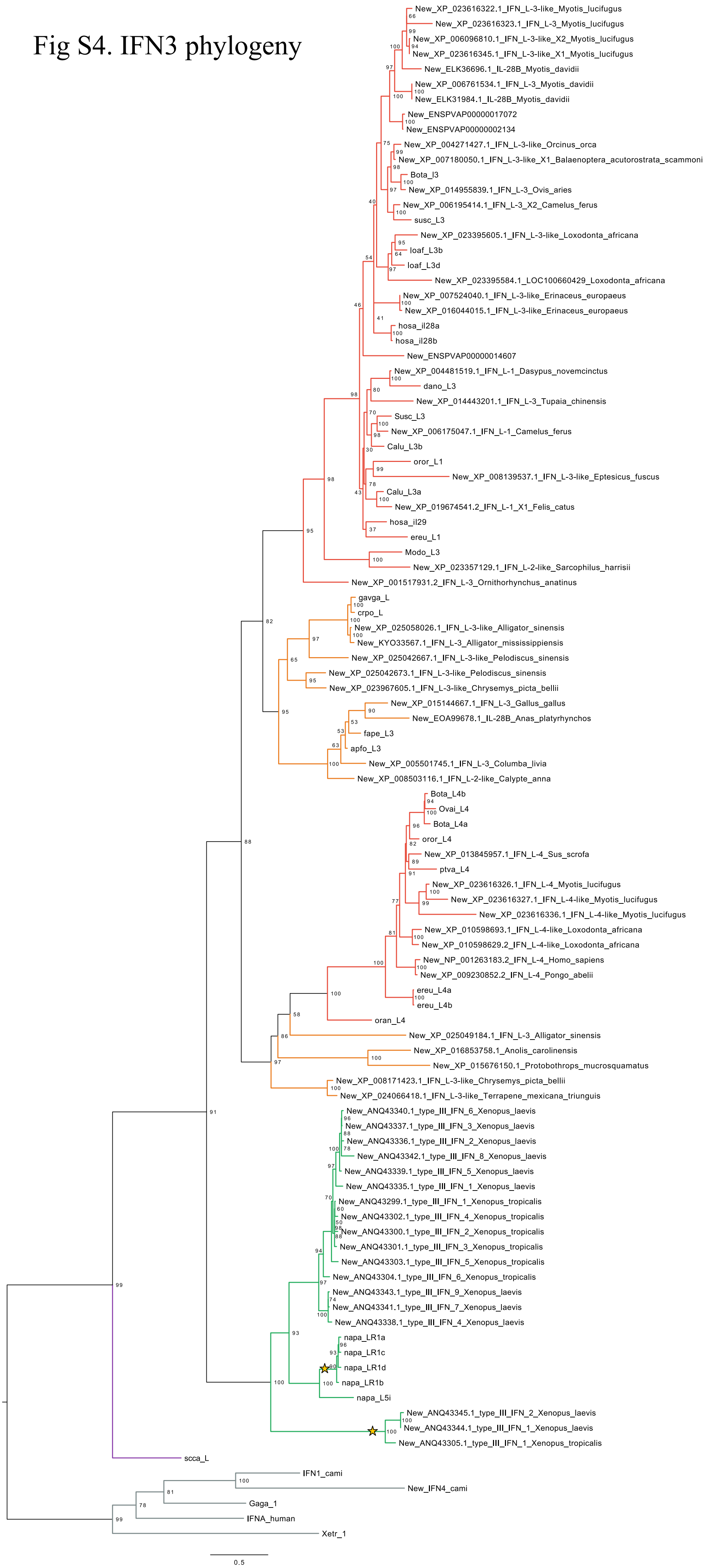

Fig S5. IFN1 CHOM Phylogeny

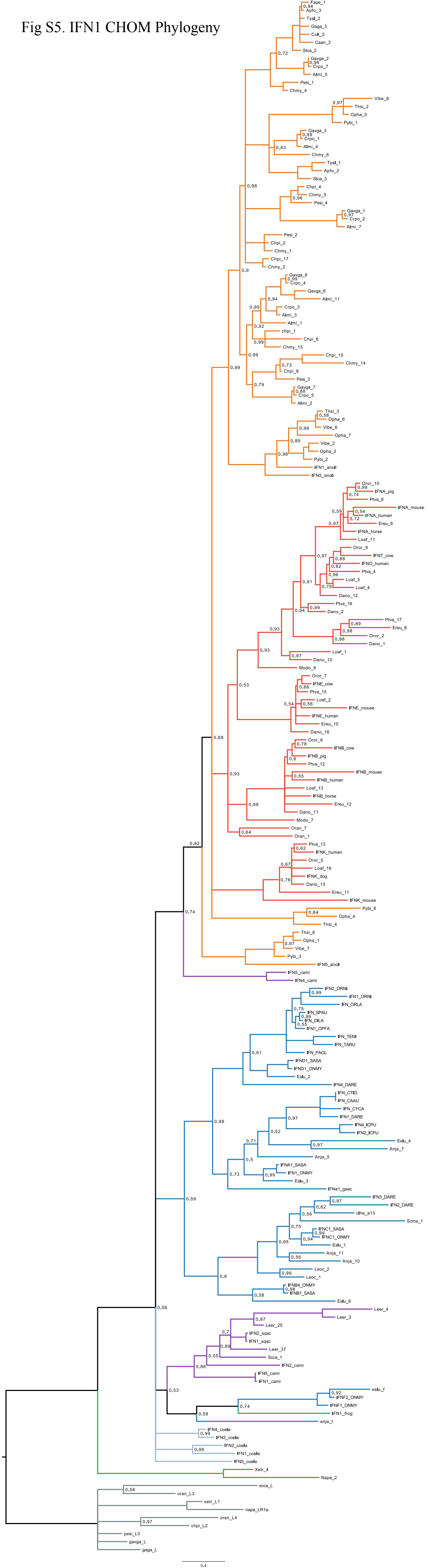

Fig S6. IFN1 FULL BEAST Phylogeny

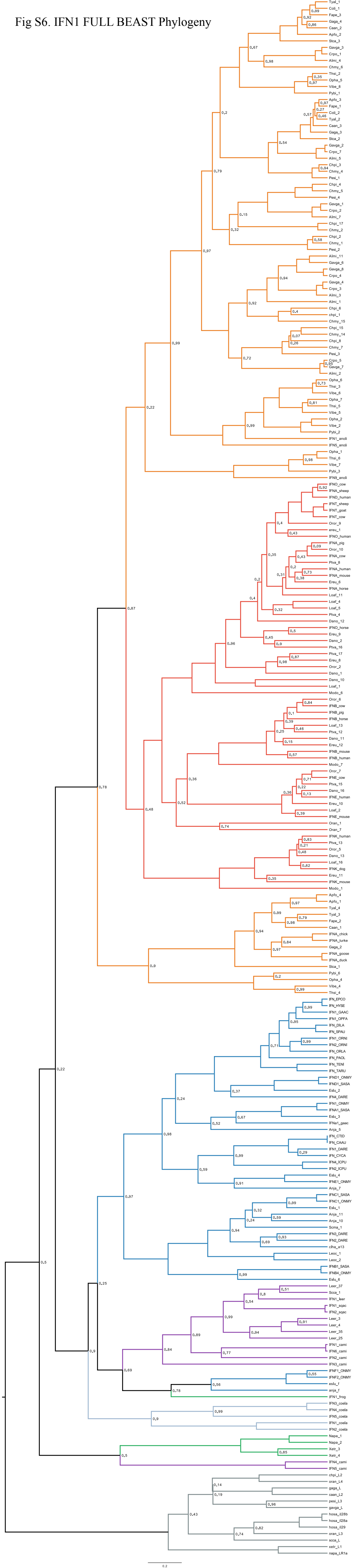

Fig S7. IFN1 CHOM
LBA test Phylogeny

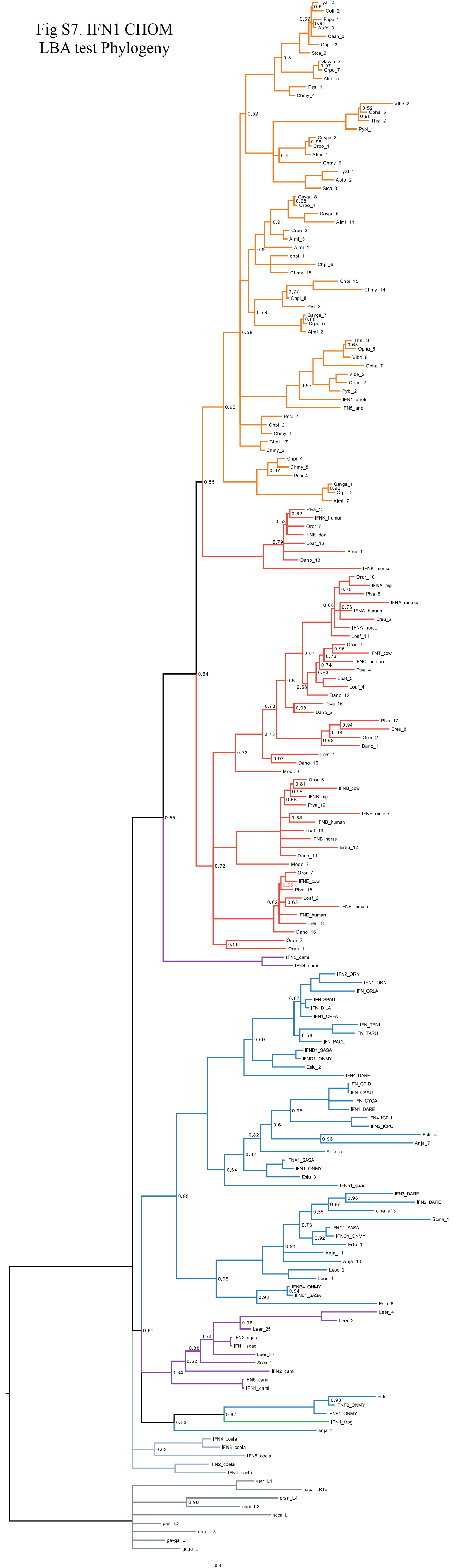

Fig S8. IFN1 CHOM poorer-fitting model Phylogenies

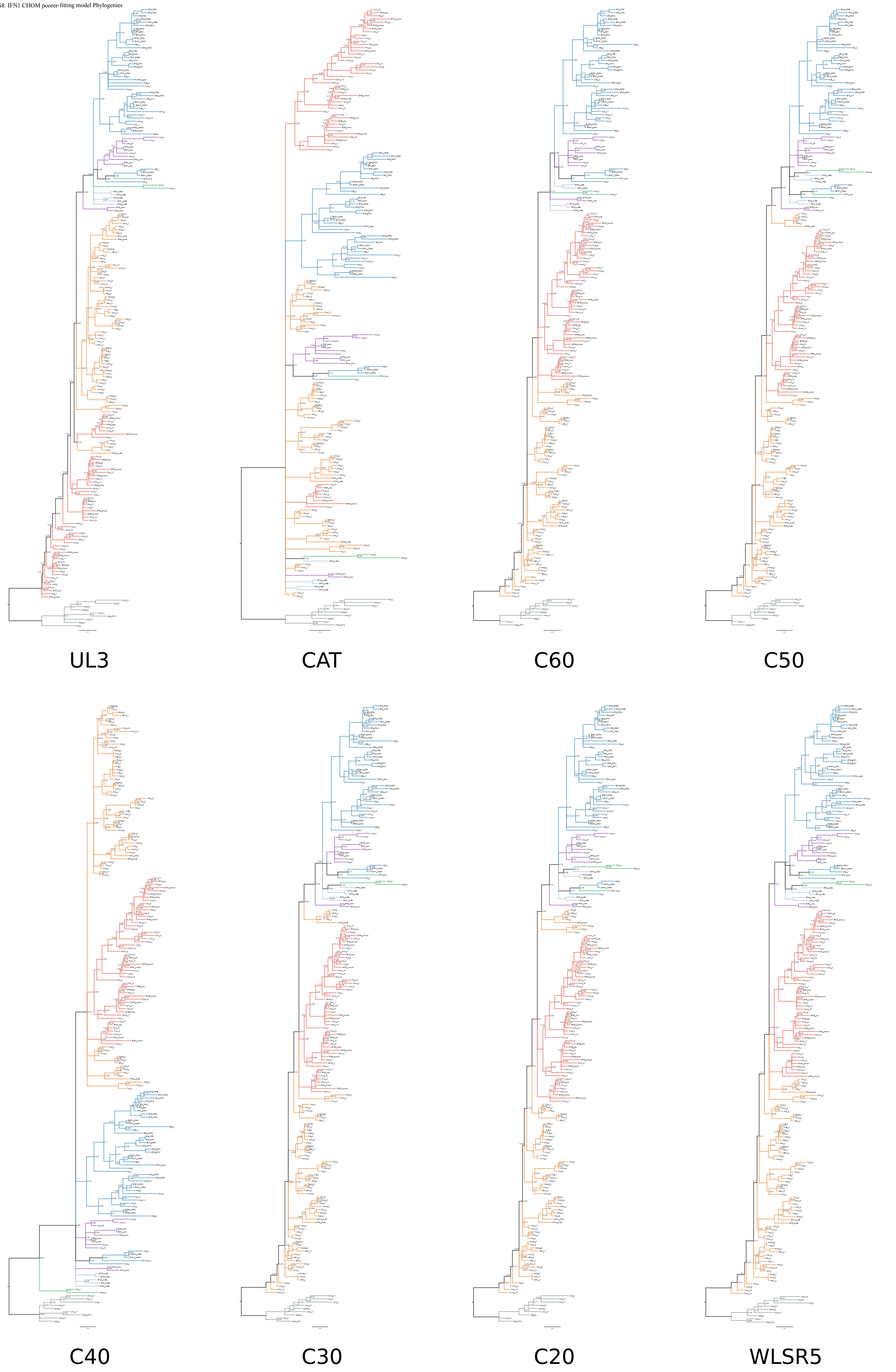

Fig S9. IFN1 CHOM IFN-gamma outgroup Phylogeny

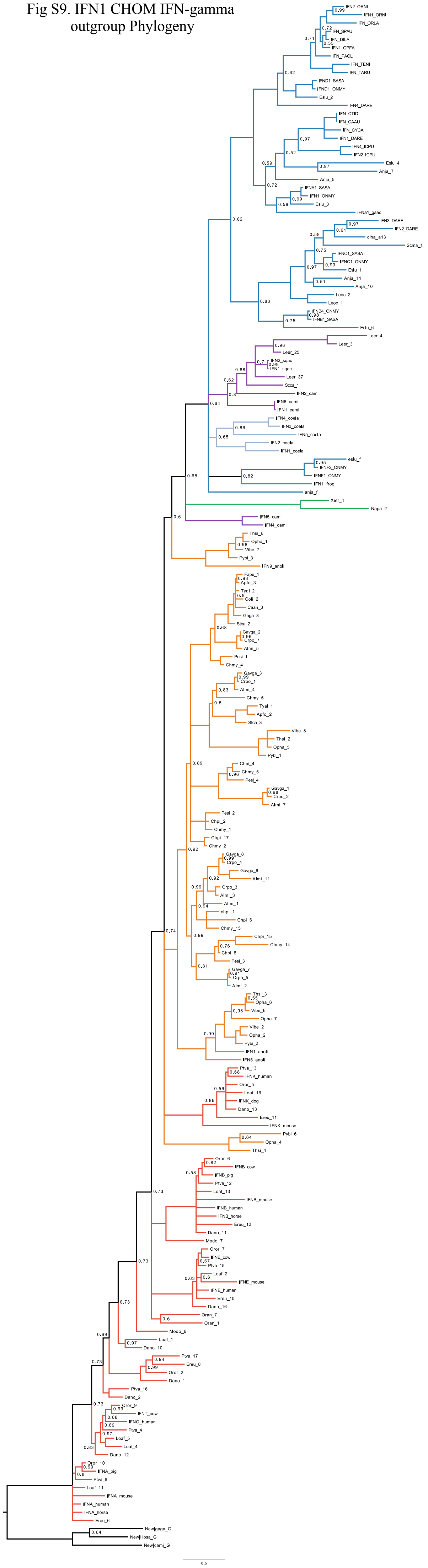

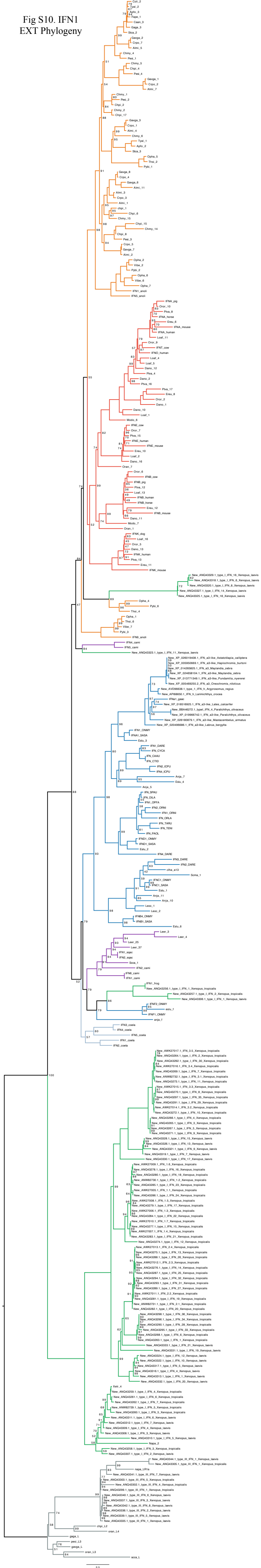

Fig S10. IFN1 EXT Phylogeny

Fig S11. IFN1 EXT Phylogeny of Amphibian-specific clade sister to other IFN1s

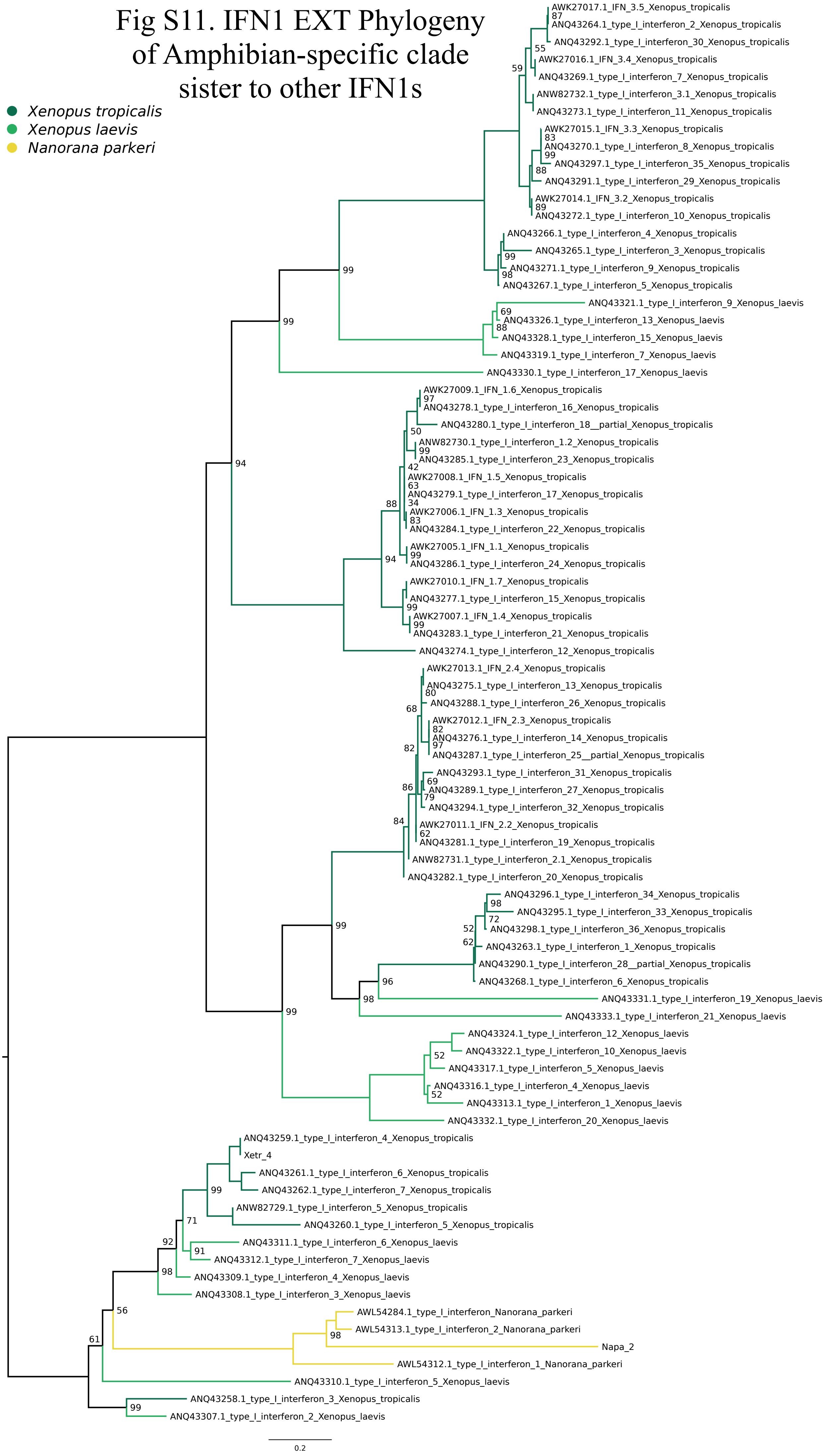

Fig S12. Amniote-specific IFN1 phylogenies

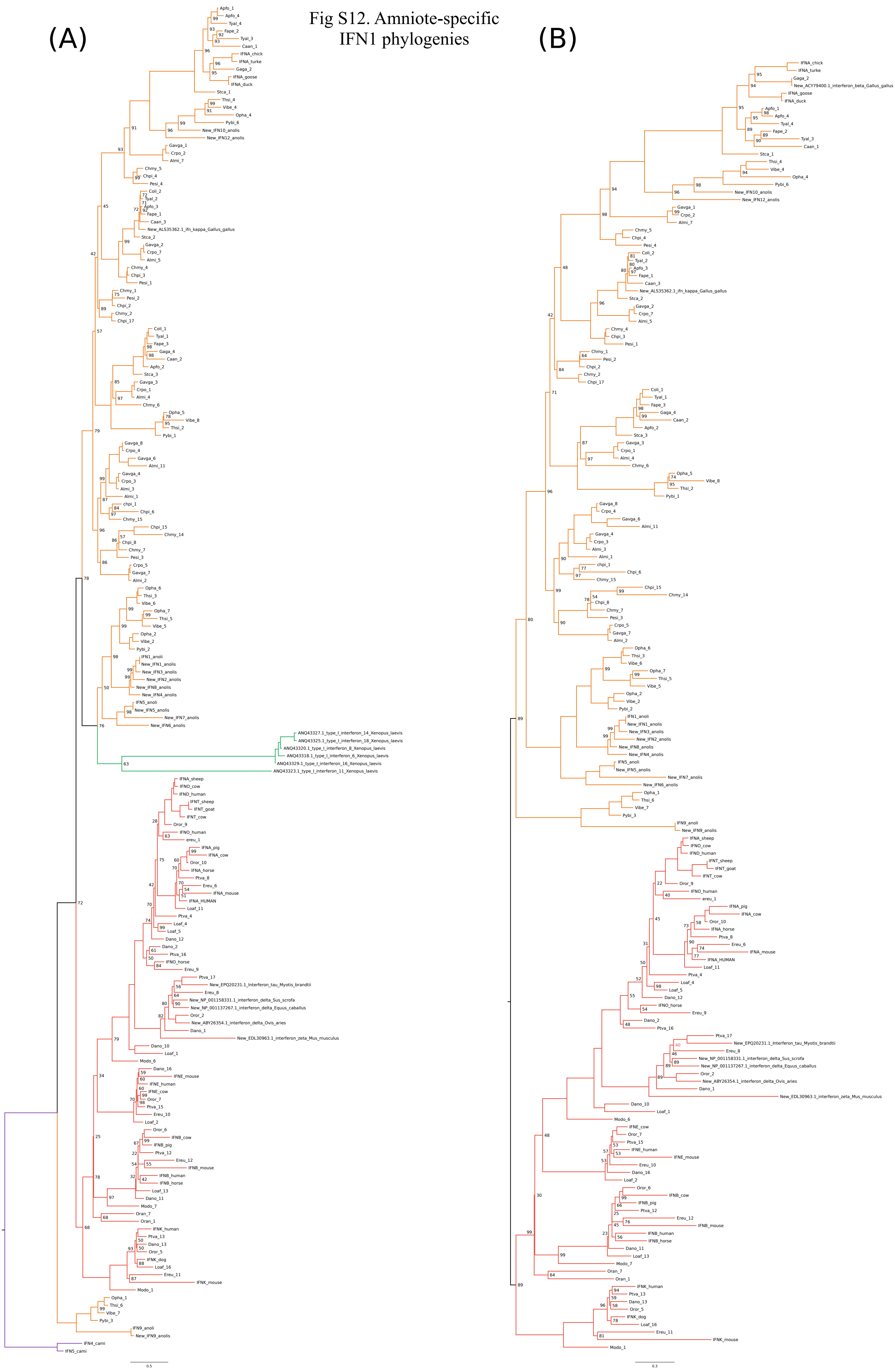

Fig S13. Non IFN-f Ray-finned fish IFN1 Phylogeny

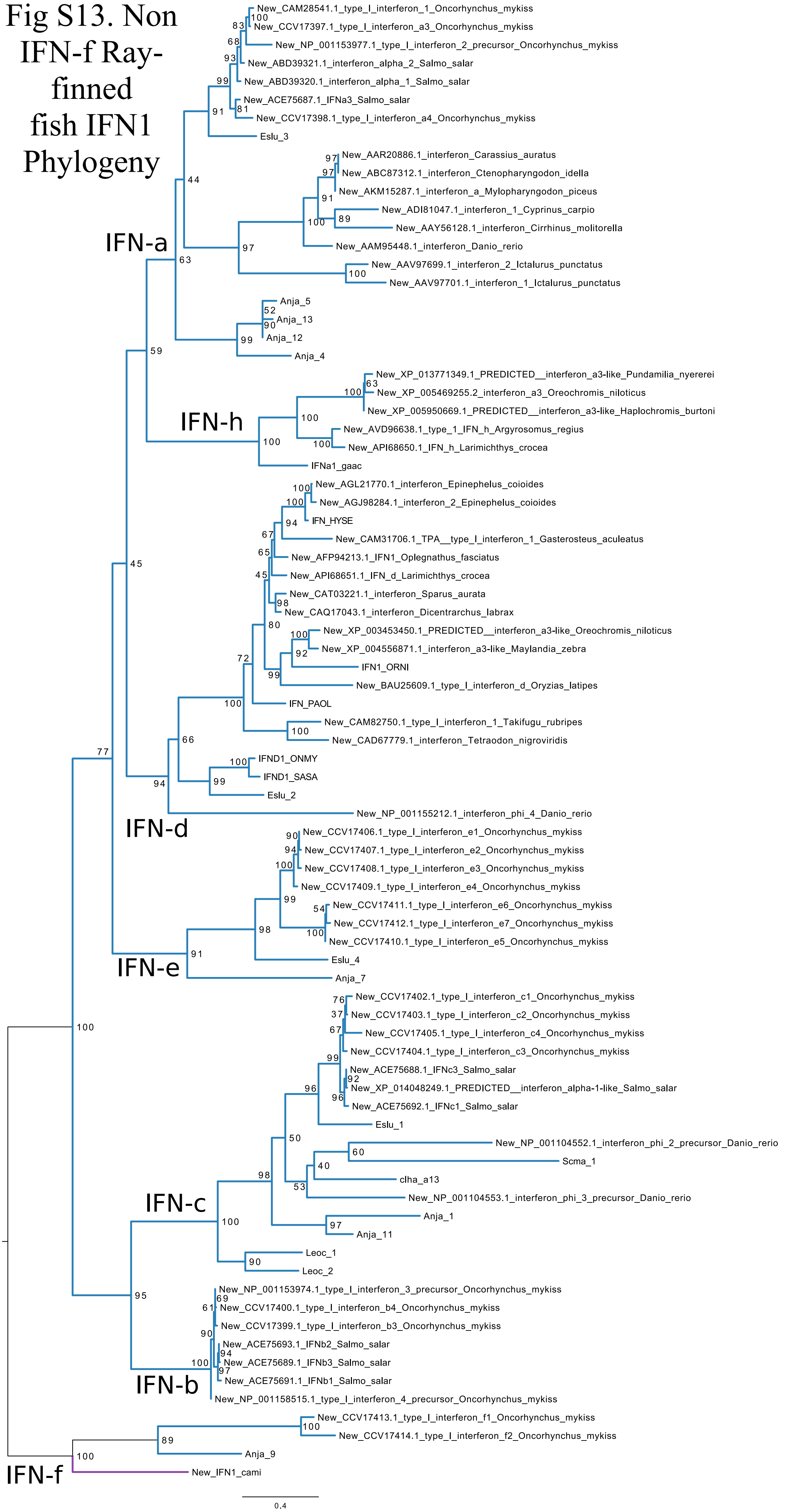

Supplement: Supplementary file 2 [file Data_Sheet_2.pdf]
