# Supplementary material for: Discovery of All Three Types in Cartilaginous Fishes Enables Phylogenetic Resolution of the Origins and Evolution of Interferons
Source: Front Immunol. 2019 Jul 12;10:1558. doi: 10.3389/fimmu.2019.01558 (PMC6640115; doi:10.3389/fimmu.2019.01558)
Supplement: Supplementary file 3 [file Table_1.docx]

Discovery of all three types in cartilaginous fishes enables phylogenetic resolution of the origins and evolution of interferons

Anthony K. Redmond, Jun Zou, Christopher J. Secombes, Daniel J. Macqueen, Helen Dooley

Supplementary Tables

**Table S1.** Genomes searched for interferon genes.

| **Species** | **Assembly** | **Searched for:** | | |
| --- | --- | --- | --- | --- |
|  |  | **IFN1** | **IFN2** | **IFN3** |
| *Branchiostoma lanceolatum* | GCA_900088365.1 | ✓ | ✓ | ✓ |
| *Ciona Intestinalis* | GCA_000224145.3 | ✓ | ✓ | ✓ |
| *Oikopleura dioica* | GCA_000209535.1 | ✓ | ✓ | ✓ |
| *Lethenteron camtschaticum* | GCA_000466285.1 | ✓ | ✓ | ✓ |
| *Petromyzon marinus* | GCA_002833325.1 | ✓ | ✓ | ✓ |
| *Callorhinchus milii* | <http://esharkgenome.imcb.a-star.edu.sg/>  & GCA_000165045.2 | ✓ |  | ✓ |
| *Scyliorhinus canicula* | Transcriptome (1) | ✓ | ✓ | ✓ |
| *Leucoraja erinacea* | <http://skatebase.org> | ✓ |  |  |
| *Squalus acanthias* | Unpublished transcriptome | ✓ |  |  |
| *Esox Lucius* | GCA_000721915.2 | ✓ |  |  |
| *Salmo salar* | GCA_000233375.4 |  |  | ✓ |
| *Anguilla japonica* | GCA_000470695.1 | ✓ |  | ✓ |
| *Danio rerio* | GCA_000002035.4 |  |  | ✓ |
| *Takifugu rubripes* | GCA_000180615.2 |  |  | ✓ |
| *Lepisosteus oculatus* | GCA_000242695.1 |  |  | ✓ |
| *Latimeria chalumnae* | GCA_000225785.1 | ✓ |  | ✓ |
| *Nanorana parkeri* | GCA_000935625.1 | ✓ |  | ✓ |
| *Xenopus siluriana tropicalis* | GCA_000004195.3 |  |  | ✓ |
| *Anolis carolinensis* | GCA_000090745.2 | ✓ |  |  |
| *Pelodiscus sinensis* | GCA_000230535.1 | ✓ |  | ✓ |
| *Chelonia mydas* | GCA_000344595.1 | ✓ | ✓ |  |
| *Chrysemys picta belii* | GCA_000241765.2 | ✓ | ✓ | ✓ |
| *Gavialus gangeticus* | GCA_000775435.1 | ✓ | ✓ | ✓ |
| *Crocodylus porosus* | GCA_000768395.1 | ✓ | ✓ | ✓ |
| *Alligator mississippiensis* | GCA_000281125.1 | ✓ | ✓ |  |
| *Python molurus bivittatus* | GCA_000186305.2 | ✓ |  | ✓ |
| *Vipera berus berus* | GCA_000800605.1 | ✓ |  |  |
| *Ophiophagus Hannah* | GCA_000516915.1 | ✓ |  |  |
| *Thamnophis sirtalis* | GCA_001077635.2 | ✓ |  |  |
| *Calypte anna* | GCA_000699085.1 | ✓ | ✓ | ✓ |
| *Gallus gallus* | GCA_000002315.2 | ✓ |  |  |
| *Falco peregrinus* | GCA_000337955.1 | ✓ | ✓ |  |
| *Struthio camelus australis* | GCA_000698965.1 | ✓ | ✓ |  |
| *Tyto alba* | GCA_000687205.1 | ✓ | ✓ |  |
| *Aptenodytes forsteri* | GCA_000699145.1 | ✓ | ✓ | ✓ |
| *Columba livia* | GCA_000337935.1 | ✓ | ✓ | ✓ |
| *Ornithorhynchus anatinus* | GCF_000002275.2 | ✓ | ✓ | ✓ |
| *Pteropus vampyrus* | GCA_000151845.2 | ✓ | ✓ | ✓ |
| *Loxodonta africana* | GCA_000001905.1 | ✓ | ✓ | ✓ |
| *Dasypus novemcinctus* | GCA_000208655.2 | ✓ | ✓ | ✓ |
| *Erinaceus europaeus* | GCA_000296755.1 | ✓ | ✓ | ✓ |
| *Orcinus orca* | GCA_000331955.2 | ✓ | ✓ | ✓ |
|  |  |  |  |  |

**References:**

1. Redmond AK, Macqueen DJ, Dooley H. Phylotranscriptomics suggests the jawed vertebrate ancestor could generate diverse helper and regulatory T cell subsets. *BMC Evol Biol* (2018) **18**:169. doi:10.1186/s12862-018-1290-2

**Table S2.** Accession numbers, new sequences, and titles in trees of interferon genes. (**see excel file**)

**Table S3.** Sequences removed from FULL dataset to form CHOM, as well as their Z-scores from the compositional heterogeneity PPS.

| **Removed Species** | **Chain 1 Z-scores** | **Chain 2 Z-scores** |
| --- | --- | --- |
| caan_L2 | 4.179 | 3.711 |
| hosa_il28a | 2.576 | 2.589 |
| hosa_il28b | 2.958 | 2.933 |
| hosa_il29 | 3.168 | 2.818 |
| Xetr_3 | 2.205 | 2.107 |
| Napa_1 | 3.113 | 3.053 |
| IFN1_leer | 2.015 | Not significant |
| Leer_35 | 2.385 | 2.397 |
| IFNA_cow | 2.033 | 2.081 |
| IFNA_sheep | 3.427 | 3.62 |
| IFNO_cow | 3.1 | 3.334 |
| IFND_human | 2.576 | 2.765 |
| IFNT_sheep | 2.047 | 2.208 |
| IFNT_goat | 2.553 | 2.715 |
| IFNO_horse | 2.261 | 2.15 |
| ereu_1 | 3.741 | 3.82 |
| Ereu_9 | 2.486 | 2.559 |
| Modo_1 | Not significant | 2.018 |
| Vibe_5 | 4.34 | 4.225 |
| Thsi_5 | 2.44 | 2.509 |
| Gavga_4 | 2.371 | 2.33 |
| Chmy_7 | 2.684 | 2.727 |
| Chpi_3 | 3.441 | 3.176 |
| Coli_1 | 2.375 | 2.326 |
| Fape_3 | 2.614 | 2.821 |
| Gaga_4 | 2.636 | 2.803 |
| Caan_2 | Not significant | 2.096 |
| IFN1_GAAC | 4.023 | 3.761 |
| IFN_EPCO | 5.384 | 5.43 |
| IFN_HYSE | 3.784 | 3.769 |
| IFNE1_ONMY | 2.49 | 2.595 |
| IFNA_chick | 4.728 | 4.952 |
| IFNA_turke | 5.71 | 5.925 |
| IFNA_goose | 8.027 | 7.801 |
| IFNA_duck | 8.211 | 7.705 |
| Gaga_2 | 2.822 | 2.774 |
| Caan_1 | 6.669 | 7.105 |
| Apfo_1 | 3.986 | 4.43 |
| Apfo_4 | 2.667 | 2.986 |
| Fape_2 | 8.105 | 9.736 |
| Tyal_4 | 8.484 | 9.611 |
| Tyal_3 | 6.689 | 6.417 |
| Stca_1 | 7.015 | 7.661 |
| IFN3_cami | 2.545 | 2.5 |
| Vibe_4 | Not significant | 2.039 |
